# Sonogashira diversification of unprotected halotryptophans, halotryptophan containing tripeptides; and generation of a new to nature bromo-natural product and its diversification in water†
†Electronic supplementary information (ESI) available: Experimental procedures; NMR characterisation; LC-MS characterisation. See DOI: 10.1039/c6sc04423a



**DOI:** 10.1039/c6sc04423a

**Published:** 2016-11-11

**Authors:** M. J. Corr, S. V. Sharma, C. Pubill-Ulldemolins, R. T. Bown, P. Poirot, D. R. M. Smith, C. Cartmell, A. Abou Fayad, R. J. M. Goss

**Affiliations:** a Department of Chemistry & BSRC , University of St Andrews , St Andrews , KY16 9ST , UK . Email: rjmg@st-andrews.ac.uk; b Ecole Nationale Supérieure de Chimie de Lille , France; c Helmholtz-Institute for Pharmaceutical Research Saarland (HIPS) , Microbial Natural Products (MINS) , Saarland University , E8.166123 Saarbrücken , Germany

## Abstract

The blending together of synthetic chemistry with natural product biosynthesis represents a potentially powerful approach to synthesis; to enable this, further synthetic tools and methodologies are needed. To this end, we have explored the first Sonogashira cross-coupling to halotryptophans in water. Broad reaction scope is demonstrated and we have explored the limits of the scope of the reaction. We have demonstrated this methodology to work excellently in the modification of model tripeptides. Furthermore, through precursor directed biosynthesis, we have generated for the first time a new to nature brominated natural product bromo-cystargamide, and demonstrated the applicability of our reaction conditions to modify this novel metabolite.

## Abstract 2

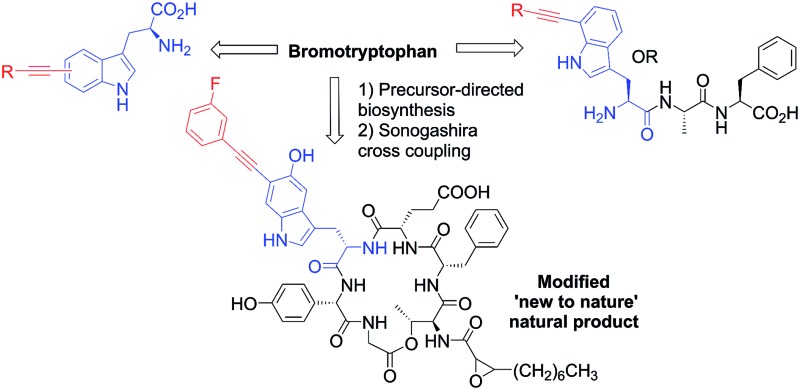
Aqueous Sonogashira cross-coupling of unprotected bromotryptophan, tripeptides and a new to nature natural product (accessed through biosynthetic manipulation) is reported.

## Introduction

**Scheme 1:**
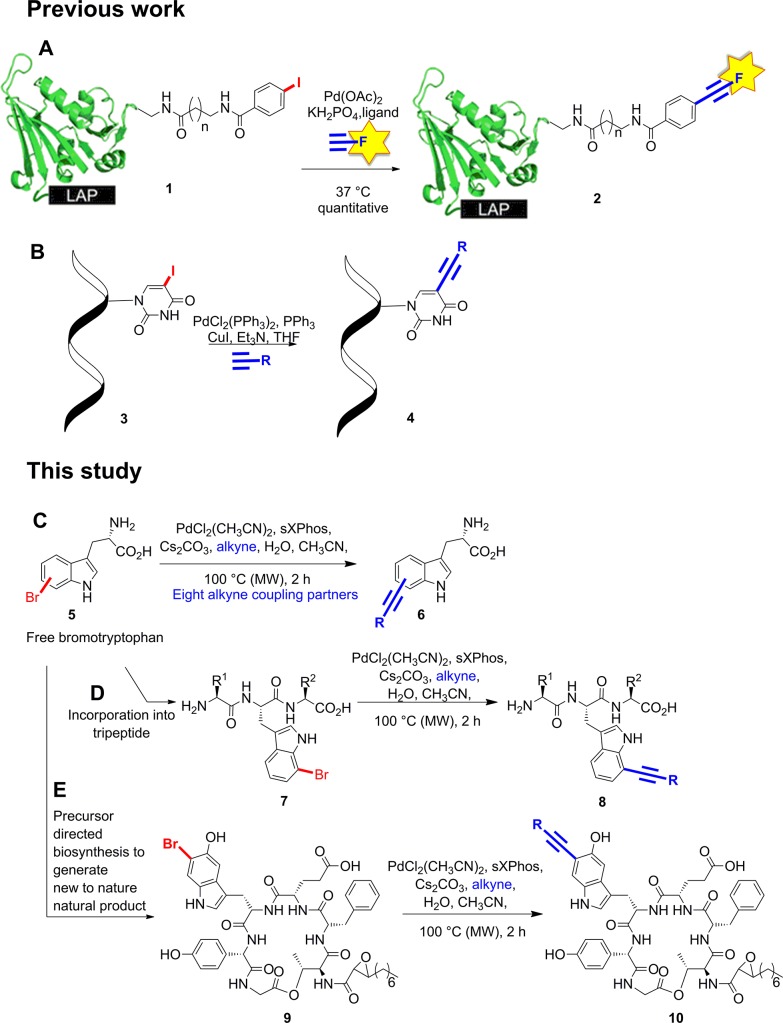
Sonogashira cross-coupling modification of natural product analogues. (A) Labelling of iodophenyl derivatives with fluorescent tags.^9^ (B) Cross-coupling of iodinated pyrimidine nucleoside.^7*a*^ (C) Sonogashira cross-coupling of unprotected bromotryptophans. (D) Sonogashira cross-coupling of 7-bromotryptophan in a tripeptide system. (E) New to nature bromo-cystargamide.

Natural products represent a valuable resource for medicinally relevant compounds, with many antimicrobial and antitumour agents entering clinical trials being based on natural products.^1^ New approaches to modify natural products and generate analogues are important, enabling, for example, structure activity relationships to be probed, and the natural product's bioactivity and bioavailability to be modulated, or enabling natural product tagging for fluorescence detection or immobilisation.^2a–d^ Palladium cross-coupling reactions are a powerful tool in synthesis and potentially for late-stage analogue generation of natural products.^3^ We previously reported the first utilisation of Suzuki–Miyaura cross-coupling for the diversification of free, unprotected halotryptophans.^2*e*^ Developing a new GenoChemetic approach to natural product analogue generation, we showed how, through biosynthetic pathway manipulation, a new to nature chlorinated antibiotic could be accessed and reported on the utility of palladium cross-coupling reactions for synthetic diversification of this chloro-uridyl peptide antibiotic.^2*f*^ In order to expand this GenoChemetic approach and our toolkit of compatible reactions, we set out to explore the potential applicability of the Sonogashira cross-coupling reaction.^4^ In this work, we wanted to examine, for the first time, whether the Sonogashira cross-coupling could be utilised to selectively diversify unprotected halotryptophans. Through biosynthetic pathway manipulation, we generated a new-to-nature bromo-cystargamide, and set out to explore whether the Sonogashira methodology that we had developed could be utilised in selectively modifying bromo-tryptophan residues as a component of a tripeptide or within our novel bromo natural product (Scheme 1).

The Sonogashira cross-coupling reaction of aryl amino acid derivatives has been shown to be important, enabling the modification of proteins and peptides through an alkynyl linker.^5d,5f,5g,5i,5l,8,9^ The reaction has also shown applicability in tagging the proteins with fluorescence labels (Scheme 1A),^5*a*^ in tagging for Fluorescence Resonance Energy Transfer (FRET)^5c,10^ and Positron Emission Tomography (PET) studies.^5*k*^ Such cross-coupling investigations within peptides, however, almost exclusively focused on either the diversification of highly reactive alkynyl derivatives embedded within peptides or iodo-phenyl derivatives such as iodophenylalanine^5^ and iodo-tyrosine.^5a,6^ Strategies to exploit the Sonogashira reaction to modify iodinated or alkynylated nucleic acids have also been successfully employed (Scheme 1B).^5a,7^ The reaction frequently requires the use of protected substrates, however, Sonogashira cross-coupling of unprotected amino acids, including iodo and bromo phenylalanine and tyrosine were also reported.^5l,6e^

The intrinsic fluorescence of tryptophan often determines, or modulates, the spectrophotometric properties of a given peptide or protein; and is a crucial residue for stabilising secondary and tertiary structure through intra and intermolecular interactions.^11^ It is also an important residue in many natural products. It is found in its halogenated form in a wide variety of metabolites including anticancer agents Rebeccamycin^12^ and diazonamide A.^13^ The possibility of selective chemical modification of such residues within natural products and peptides is attractive.^14^ However, though much research has been carried out on the Sonogashira modification of halophenylalanines and halotyrosines, to the best of our knowledge, there are no prior reports of the utilisation of this strategy for the Sonogashira diversification of more challenging halotryptophans, either as a discrete amino acid or as a component of a peptide or natural product.

Halotryptophans may be readily accessed through a simple one-step biotransformation using tryptophan synthase,^15,16^ or through a 4–5 step chemical synthesis.^17^ Challenges that need to be addressed to render Sonogashira cross-coupling of free halotryptophans useful are their poor solubility and their propensity to chelate to and deactivate the palladium catalyst.^18^

## Results and discussion

**Fig. 1:**
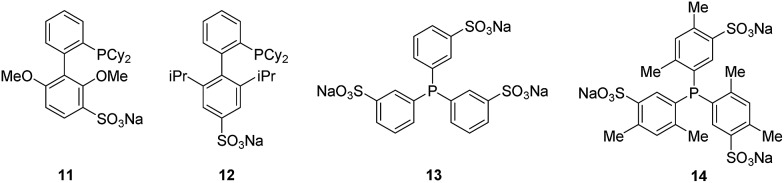
Water soluble ligands for Pd mediated cross-coupling. sSPhos **11**, sXPhos **12**, TPPTS **13** and TXPTS **14**.

We previously reported the Suzuki–Myaura cross-coupling of bromotryptophan-containing natural product analogues^2*e*^ using the Buchwald's water-soluble ligand sSPhos **11**.^19^ Anderson and Buchwald reported the use of water-soluble ligand sXPhos **12** for the Sonogashira coupling of aryl halides including chloropyridine.^19^ We decided to explore whether this ligand could be utilised to facilitate the Sonogashira coupling of halotryptophans in water. To start with a slightly less challenging substrate, we first investigated whether Sonogashira modification of unprotected 5-bromoindole **15** could be achieved (Fig. 1).

**Scheme 2:**
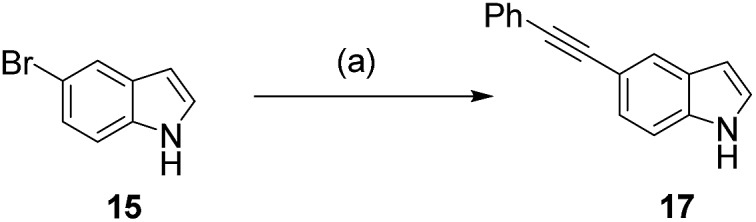
Sonogashira cross-coupling reaction of 5-bromoindole **15** with phenylacetylene **16**. (a) Reagents and conditions are described in Table 1.

Encouragingly, we were able to demonstrate the conversion of 5-bromoindole **15** with phenylacetylene **16**, in a 1 : 1 mixture of water and acetonitrile to its alkynylated derivative **17** in 76% conversion (Scheme 2, and Table 1, entry 1). This provided a good starting point, however, we observed that increasing the catalyst and ligand loading (from 2.5 to 5 mol% and from 7.5 to 15 mol%, respectively) improved the conversion to 93% (Table 1, entry 2). The reaction could be further improved by increasing the number of equivalents of alkyne coupling partner from 1.5 eq. to 3.0 eq., leading to complete conversion of 5-bromoindole **15** to the cross-coupling product **17** (Table 1, entry 3). With conditions in hand that enabled the cross-coupling of 5-bromoindole **15** to go to completion, we sought to investigate if the reaction time could be decreased. Switching from conventional heating to microwave heating at 100 °C proved to be successful, with full conversion being observed after 2 h heating in the microwave (Table 1, entry 4), rather than the 18 h previously required.

**Table 1:** Conditions^*a*^ for the Sonogashira cross-coupling reaction of 5-bromoindole **15** and 5-bromotryptophan **18** with phenylacetylene **16**

Entry	Substrate	Cat/mol%	Ligand/mol%	Alkyne loading/eq.	Heating method	Reaction time/h	NMR conv./%
1	**15**	2.5	7.5	1.5	Conventional	18	76^*b*^
2	**15**	5.0	15.0	1.5	Conventional	18	93^*b*^
3	**15**	5.0	15.0	3.0	Conventional	18	>99^*b*^
4	**15**	5.0	15.0	3.0	Microwave	2	>99^*b*^
5	**18**	5.0	15.0	3.0	Microwave	2	>99^*c*^

^*a*^Substrate (0.1 mmol), PdCl_2_(CH_3_CN)_2_ catalyst, sXPhos ligand **12**, Cs_2_CO_3_ (2.5 eq.), phenylacetylene **16**, CH_3_CN/H_2_O (1 : 1, 2 mL), sealed tube, 100 °C, solids and solvents purged with nitrogen.

^*b*^Based on ratio of starting material aromatic peak at *δ*_H_ 6.50 ppm compared to product aromatic peak at *δ*_H_ 6.56 ppm in CDCl_3_.

^*c*^Based on ratio of starting material aromatic peak at *δ*_H_ 7.89 ppm compared to product aromatic peak at *δ*_H_ 7.96 ppm in CD_3_OD.

Having established conditions to enable quantitative conversion of 5-bromoindole **15** within 2 h, we next explored the reaction with what we had perceived to be the more challenging unprotected 5-bromotryptophan **18**. Pleasingly, the reaction proceeded remarkably well with the tryptophan substrate and complete conversion to the product was observed after microwave heating for 2 h under the same conditions that we had developed for 5-bromoindole **15** (Table 1, entry 5).

With the successful quantitative conversion of unprotected 5-bromotryptophan **18** accomplished, we next investigated the limits of the reaction and specifically the impact of a series of water-soluble ligands. In order to determine the broader applicability we also explored the impact of air, the effect of substrate concentration on conversion, and the influence of temperature on the reaction (Table 2). To this end, we first investigated series of commercially available water-soluble ligands, sSPhos **11** (88%), TPPTS **13** (50%) and TXPTS **14** (83%) were all shown to mediate reasonable conversions of 5-bromotryptophan **18** to the expected product **19** (Table 2, entries 2–4), however, none of the ligands tested matched sXPhos **12** for performance (Table 2, entry 1). The reactions to this point had been carried out under inert conditions with de-gassed solvents, not always simple to achieve for biomaterials. In order to investigate the impact of air on the cross-coupling, the reaction was repeated in air using non-degassed solvents (Table 2, entry 5). The reaction proceeded well in the presence of oxygen with only a modest decrease in the observed conversion (86% with respect to >99%, Table 2, entries 5 and 1, respectively). Our results showed that an inert atmosphere was preferential for the reaction. However, the reaction still proceeded to give high conversion without the exclusion of oxygen from the reaction, showing that the conditions should work well for reactions where maintaining an inert atmosphere may prove difficult.

**Table 2:** Investigating the reaction of halo-tryptophans and phenylacetylene **16**. Conversions achieved under various conditions: exploring the impact of the ligand (entries 2–4), reaction in the presence of air (entry 5), concentration (entries 6 and 7), impact of temperature (entry 8), 5-chlorotryptophan **20** as substrate in place of 5-bromotryptophan **18** (entry 9)

Entry	Substrate	Substrate amount in mmol (concentration)	Ligand	*T*/°C	NMR conv.^*c*^/%
1	**18**	0.1 (50 mM)^*a*^	sXPhos	100	>99
2	**18**	0.1 (50 mM)^*a*^	sSPhos	100	88
3	**18**	0.1 (50 mM)^*a*^	TPPTS	100	50
4	**18**	0.1 (50 mM)^*a*^	TXPTS	100	83
5	**18**	0.1 (50 mM)^*b*^	sXPhos	100	86
6	**18**	0.05 (25 mM)^*a*^	sXPhos	100	67
7	**18**	0.025 (12.5 mM)^*a*^	sXPhos	100	34
8	**18**	0.1 (50 mM)^*a*^	sXPhos	80	35
9	**20**	0.1 (50 mM)^*a*^	sXPhos	100	<1

^*a*^Substrate (1 eq.), PdCl_2_(CH_3_CN)_2_ (5 mol%), ligand (15 mol%), Cs_2_CO_3_ (2.5 eq.), phenylacetylene (3.0 eq.), CH_3_CN/H_2_O (1 : 1, 2 mL), microwave heating, sealed tube, solids and solvents purged with nitrogen.

^*b*^As with^*a*^, except solids and solvents were not nitrogen purged.

^*c*^Based on ratio of starting material aromatic peak at *δ*_H_ 7.89 ppm compared to product aromatic peak at *δ*_H_ 7.96 ppm in CD_3_OD.

We next focused on studying the effect of concentration on the reaction. The reaction was carried out at 50 mM, 25 mM and 12.5 mM (Table 2, entries 1, 6 and 7, respectively). As expected, the conversion is not as efficient at lower concentrations, however, modest conversion, using the same catalyst loading, could be observed at a 4-fold dilution. There may be scope for improving these conversions, by increasing catalyst loading and coupling partner, when the halogenated substrate is at high dilution. Finally, the effects of lowering the temperature of the reaction were investigated. We observed that a slight reduction in reaction temperature from 100 °C to 80 °C (Table 2, entry 8) significantly decreased the observed conversion from >99% to 35%, indicating that 100 °C was likely to be close to optimal for this reaction.

Previous investigations of the Sonogashira cross-coupling have focused predominantly on aryl iodides; and so it is exciting to see that conditions can be developed to enable quantitative Sonogashira conversion of the less reactive bromotryptophans. The chloro analogues are even less reactive, but again in order to determine the limitations of the reaction conditions, the reaction of 5-chlorotryptophan **20** with phenylacetylene **16** was attempted (Table 2, entry 9). Unfortunately, this highly unreactive substrate showed no reaction after heating for 2 h in the microwave, with only starting material being observed.

With the optimal conditions for the Sonogashira coupling of halotryptophans determined, we then sought to examine the scope of the reaction by varying the halogen and its position, as well as the alkyne coupling partner (Scheme 4, Tables 3 and 4). The reactions were carried out with 5-bromotryptophan **18**, 6-bromotryptophan **21**, 7-bromotryptophan **22** and 7-iodotryptophan **23** with a range of commercially available terminal alkynes (Scheme 4, **16**, **24–33**). In each reaction, the conversion of the starting halo-tryptophan to the cross-coupled product was determined by ^1^H NMR. To obtain indication as to representative isolable yields for each alkynyl coupling partner, the product from reaction with the 5-bromotryptophan **18** was purified to determine the isolated yield (purified by reverse-phase HPLC and carried out in triplicate).

**Table 3:** Reaction of halo-tryptophans with commercially available phenylacetylenes **16**, **24**, **25** and ethynylthiophene **26** ^*a*^

Entry	Tryptophan	Alkyne	Product	Conversion^*b*^ (yield^*c*^)/%
1	5-Br **18**	**16**	**19**	>99 (80)
2	6-Br **21**	**16**	**34**	>99
3	7-Br **22**	**16**	**35**	81
4	7-I **23**	**16**	**35**	79
5	5-Br **18**	**24**	**36**	>99 (97)
6	6-Br **21**	**24**	**37**	>99
7	7-Br **22**	**24**	**38**	>99
8	7-I **23**	**24**	**38**	>99
9	5-Br **18**	**25**	**39**	>99 (79)
10	6-Br **21**	**25**	**40**	>99
11	7-Br **22**	**25**	**41**	>99
12	7-I **23**	**25**	**41**	>99
13	5-Br **18**	**26**	**42**	>99 (75)
14	6-Br **21**	**26**	**43**	>99
15	7-Br **22**	**26**	**44**	>99
16	7-I **23**	**26**	**44**	>99

^*a*^Substrate (0.1 mmol, 1.0 eq.), alkyne (0.3 mmol, 3.0 eq.), PdCl_2_(CH_3_CN)_2_ (5 mol%), sXPhos (15 mol%), Cs_2_CO_3_ (2.5 eq.), water/acetonitrile (1 : 1, 2 mL, degassed), microwave heating, 100 °C, 2 h.

^*b*^Based on NMR ratios of starting material to product in CD_3_OD.

^*c*^Average of three isolated yields.

**Table 4:** Reaction of halo-tryptophans with commercially-available alkynes **27–33** ^*a*^

Entry	Tryptophan	Alkyne	Product	Conversion^*b*^ (yield^*c*^)/%
1	5-Br **18**	**27**	**45**	>99 (81)
2	6-Br **21**	**27**	**46**	>99
3	7-Br **22**	**27**	**47**	81
4	7-I **23**	**27**	**47**	85
5	5-Br **18**	**28**	**48**	63 (26)
6	6-Br **21**	**28**	**49**	>99
7	7-Br **22**	**28**	**50**	74
8	7-I **23**	**28**	**50**	39
9	5-Br **18**	**29**	**51**	54 (51)
10	6-Br **21**	**29**	**52**	>99
11	7-Br **22**	**29**	**53**	>99
12	7-I **23**	**29**	**53**	56
13	5-Br **18**	**30**	**54**	16
14	5-Br **18**	**30**	**54**	50 (∼25^*d*^)
15	5-Br **18**	**31**	**55**	<1
16	5-Br **18**	**32**	**56**	<1
17	5-Br **18**	**33**	**57**	<1

^*a*^Substrate (0.1 mmol, 1.0 eq.), alkyne (0.3 mmol, 3.0 eq.), PdCl_2_(CH_3_CN)_2_ (5 mol%), sXPhos (15 mol%), Cs_2_CO_3_ (2.5 eq.), water/acetonitrile (1 : 1, 2 mL, degassed), microwave heating, 100 °C, 2 h.

^*b*^Based on NMR ratios of starting material to product in CD_3_OD.

^*c*^Average of three isolated yields.

^*d*^Product co-eluted with sXPhos **4** and could not be isolated pure, the approximate yield is based on a single isolation.

**Scheme 3:**
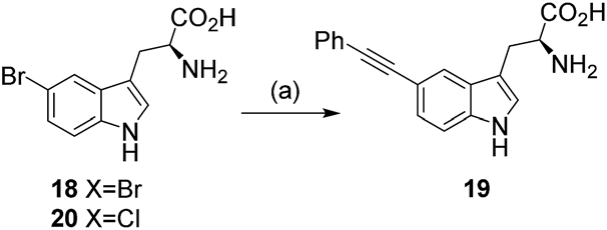
Investigating the reaction of halo-tryptophans and phenylacetylene **16**. (a) Reagents and conditions are described in Tables 1 and 2.

**Scheme 4:**
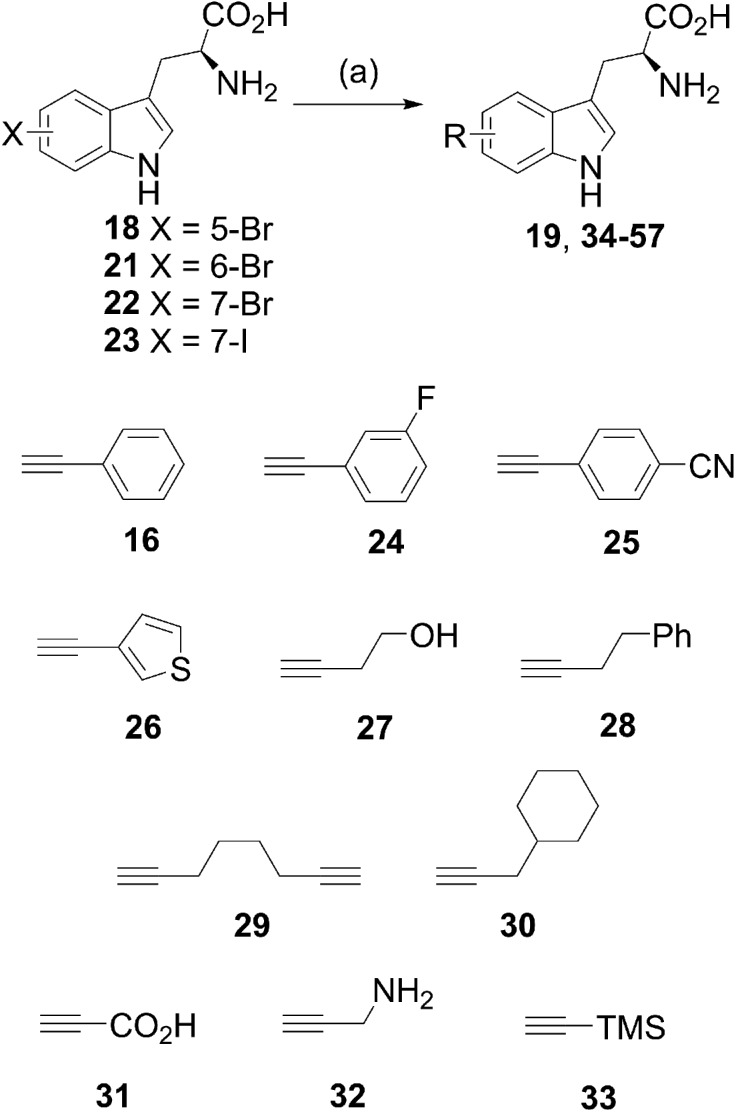
Exploring the substrate scope of the reaction.

We first examined the coupling of aromatic-substituted acetylenes. The three phenylacetylene-based alkynes (phenylacetylene **16**, 3-fluorophenylacetylene **24** and 4-cyanophenylacetylene **25**) all showed very good to excellent conversions for each tryptophan and high yields for the isolated products (Scheme 4 and Table 3). The high yield obtained for the 3-fluorophenylacetylene **24** was particularly pleasing as this enables an F label to be introduced into a molecule that can potentially be used to follow the fate of a molecule. Though current reaction times (2 h) are still rather long, there may be potential to develop this chemistry further to enable labelling for PET. Similarly, heterocyclic alkyne ethynylthiophene **26** showed excellent conversions for all the tryptophans and high yield for the isolated product (Table 3).

**Fig. 2:**
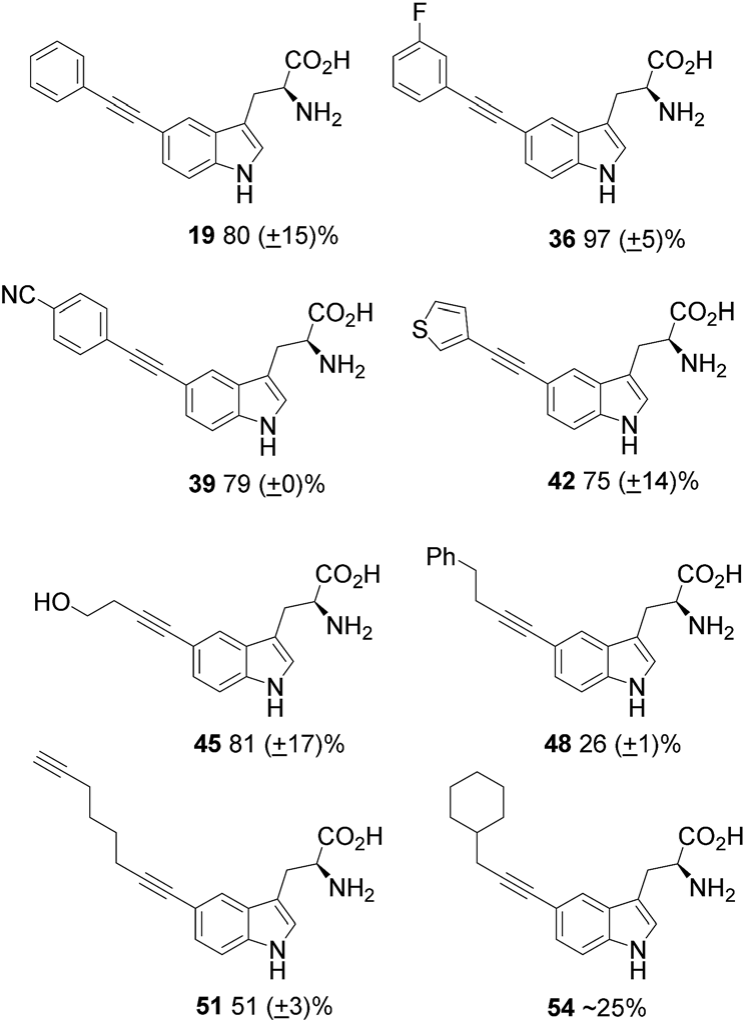
Isolated products from the coupling of 5-bromotryptophan **18**, along with isolated yields (from triplicate isolations).

Next, a range of aliphatic alkynes, containing useful functionality, and designed to introduce different levels of challenge, were investigated. Though the reaction, would not proceed with propiolic acid **31** (Table 4, entry 15), propargylamine **32** (Table 4, entry 16) or TMS acetylene (Table 4, entry 17), the cross-coupling reaction with 4-butyn-1-ol **27** proceeded with high conversion and high isolated yield for product **45** (Fig. 2) and provides an alcohol substituent for potential further modification. The reaction also proceeded with 4-phenyl-1-butyne **28** to give moderate to excellent conversions (Table 4, entries 5 to 8), but low isolated yields of product **48**. 1,7-Octadiyne **29** was chosen as a coupling partner in order to gain access to a terminal alkyne in the coupled product, in order to install an alkyne tag for subsequent alkyne–azide cycloaddition reactions.^20^ Pleasingly, the reaction proceeded to give diyne product **51** in 51% yield (average of three isolated yields), (Table 4, entry 9). 3-Cyclohexyl-1-propyne **30** was also investigated as a potential coupling partner. After the standard reaction conditions (100 °C in microwave, 2 h), ^1^H NMR showed approximately 16% conversion to the product **54** (Table 4, entry 13). Increasing the reaction time to 8 h (Table 4, entry 14) improved the conversion to 50%. The product was purified by reverse-phase HPLC, but co-eluted with the sXPhos **12** ligand, and it was challenging to obtain a definitive isolated yield in this case.

**Scheme 5:**
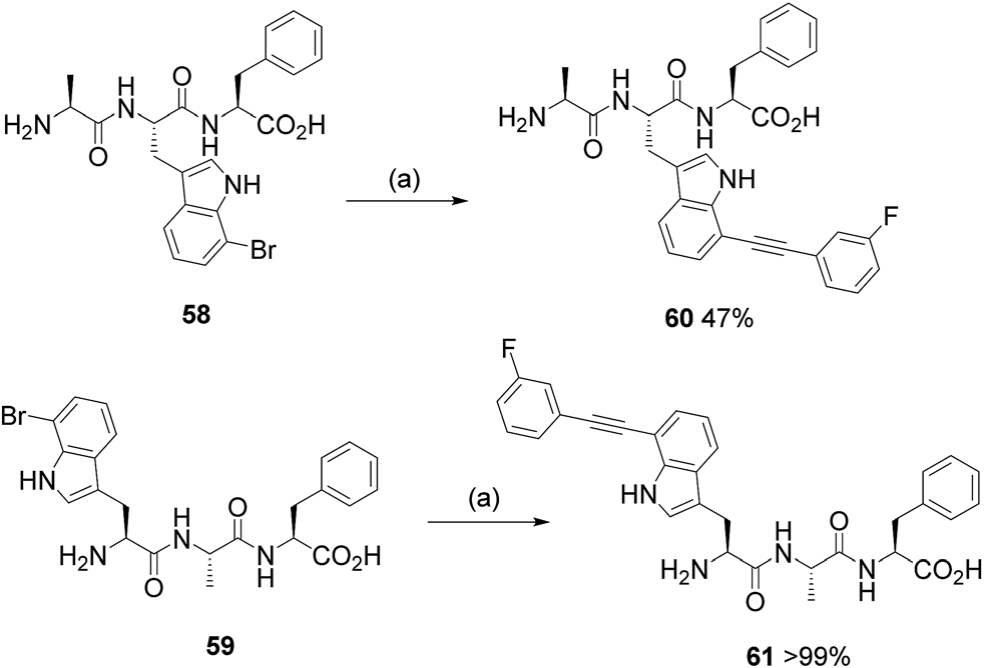
Investigating the Sonogashira modification of 7-bromo tryptophan, as a component of a tripeptide with 3-fluorophenylacetylene **24**. (a) Reagents and conditions: PdCl_2_(CH_3_CN)_2_ (5 mol%), sXPhos **12** (15 mol%), Cs_2_CO_3_ (2.5 eq.), H_2_O : CH_3_CN (1 : 1), microwave heating, 100 °C, 2 h.

Remarkably, our simple scoping reactions revealed that the developed Sonogashira cross-coupling reaction conditions could be applied to the modification of halotryptophans and could be carried out under aqueous conditions without the need for copper salts and without the need to protect the amino acid. We carried out analysis of the isolated products, as a representative set, using Marfey's reagent (see ESI†).^21^ Our analysis revealed no erosion of the enantiopurity of the tryptophans under the reaction conditions that we had employed (see ESI†). With robust conditions for tryptophan diversification *via* Sonogashira cross-coupling in hand, we next tested the reaction with more complex halo-tryptophan-containing substrates. We chose to design and synthesise two tripeptides containing an N-terminal and an internal 7-bromotryptophan. Tripeptides **58** and **59** were synthesised (see ESI†) and were subjected to the aqueous Sonogashira reaction conditions with 3-fluorophenylacetylene **24** (Scheme 5), chosen for its high efficiency as a coupling partner to free halotryptophans and also due to the benefit of being able to follow the reaction by ^19^F NMR. The reaction with Ala-(7-Br-Trp)-Phe **58** proceeded to give product **60** in 47% isolated yield. The reaction of (7-Br-Trp)-Ala-Phe **59** also gave expected product **61**, pleasingly in quantitative isolated yield. The lower isolated yield for product **60** could potentially be due to the steric hindrance and/or difficulties associated with purification due to potentially reduced aqueous solubility. The quantitative conversion of the 7-bromotryptophan residue at the N-terminus of this tripeptide is striking and opens up the possibility of using this methodology for the labelling, modification and modulation of peptides.

**Scheme 6:**
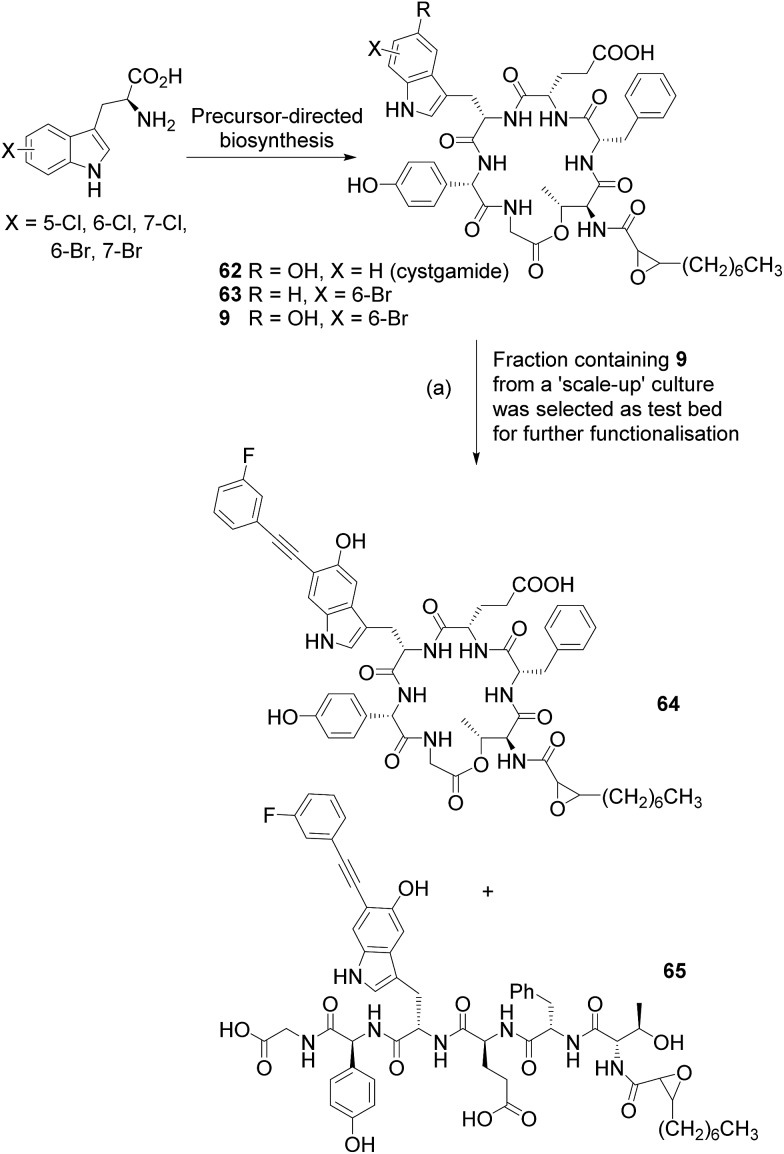
Precursor-directed biosynthesis with *K. cystarginea* to generate new-to-nature halo-analogues of cystargamide, a challenging test bed natural product system. Scale-up culture and Sonogashira reaction of semi-pure 6-bromo-cystargamide **9** with 3-fluorophenylacetylene **24** using the cross-coupling conditions that we had developed. (a) Reagents and conditions: PdCl_2_(CH_3_CN)_2_ sXPhos **12**, Cs_2_CO_3_, H_2_O : CH_3_CN (1 : 1), microwave heating, 100 °C, 2 h.

Having demonstrated the ability to apply this methodology to small peptides we next explored the reaction in the context of natural products as a potential tool for use in GenoChemetic analogue generation. The harnessing of biosynthetic pathways to force the incorporation of a halogen into a natural product provides a chemically orthogonal handle with the possibility for selective modification. We have previously demonstrated that the facile Suzuki–Miyaura cross coupling may be readily applied to such systems.^2*f*^ Cystargamide **62** (Scheme 6) is a 5-hydroxytryptophan containing cyclic lipopeptide isolated from *Kitasatospora cystarginea*.^22^ Though this compound has shown no notable biological activity to date, a very close structural analogue Q-6402 has been reported to inhibit phospholipase A_2_.^23^ Cystargamide provides an interesting and challenging test bed for GenoChemetic analogue generation: firstly because the naturally incorporated tryptophan shows hydroxylation and therefore the incorporation of other analogues of this amino acid may prove challenging, secondly because the molecule contains a number of functionalities that could potentially impede the reaction, and thirdly because it is produced in fairly low titre.^22^

We set out to explore whether halotryptophans could be incorporated, through precursor directed biosynthesis, to generate a halogenated analogue of this natural product (Scheme 6). To explore this possibility we carried out small scale feeding experiments with 7-, 6- and 5-chlorotryptophan and 7-, 6- and 5-bromotryptophan at 0.25 mM and 1.0 mM to both 4 mL cultures in 24 deep well plates and 50 mL cultures in 250 mL Erlenmeyer flasks. The cultures were extracted and analysed by LC-MSMS. Results demonstrated that all of the halotryptophans were incorporated into cystargamide, generating at low level, a series of new to nature halogenated analogues (see ESI† for details). As judged from the LC-absorbance peak area, there was no significant difference in the quantities of halo-cystargamide produced across the two tryptophan concentrations that we had carried out the precursor directed biosynthesis at, therefore, to reduce wastage of halotryptophan precursor we opted for a 0.25 mM concentration for future experiments. As we simply wanted to explore and hopefully demonstrate proof of principle by applying our Sonogashira methodology to the diversification of a novel “new to nature” natural product we decided to select just one halotryptophan for scaling up the fermentation with and feeding.

Our challenge would be to generate sufficient halogenated natural product on which to explore the cross-coupling. As we had shown the chlorotryptophans to be too unreactive for Sonogashira cross-coupling, and wanting to avoid placing a halogen on C-5 of the indole ring, which is naturally hydroxylated, and could provide a future chemically orthogonal handle for further functionalization, we set out to scale up production of 6-bromocystargamide. To this end we fed 8 L of culture (incubated in batches of 500 mL in 2 L flasks) with 0.26 mM 6-bromotryptophan. Extraction and purification resulted in an approximately 1.0 mg of a partially pure fraction containing brominated cystargamides along with other cystargamide derivatives. By comparing the LC-MS extracted ion peak areas, it could be estimated that the parent unhalogenated cystargamide **62**, a bromo-cystargamide analogue that lacked the 5-hydroxy on the tryptophan **63** and bromocystargamide **9**, were present in an approximate 10 : 0.2 : 1.4 ratio. This indicated the ability of the producing organism to process 6-bromo-tryptophan, though perhaps not quite as efficiently as unhalogenated tryptophan. Analysis of the extract also revealed quantities of linear unbrominated cystargamide in which the ester bond appeared to have been hydrolysed, and analogues of this and the brominated forms, which contained an alanine rather than glycine residue (see ESI† for more details).

A fraction containing predominantly the brominated analogue (>85%) **9** was subjected to the Sonogashira cross-coupling conditions developed within this study (Scheme 6). LC-MS analysis of the crude reaction mixture indicated that all brominated cystargamide had been consumed and revealed peaks at *m*/*z* 1072 [M + H]^+^ for product **64** and 1090 [M + H]^+^ for hydrolysed product **65** (further confirmed by MSMS), indicating successful cross-coupling with 3-fluorophenylacetylene **24** into analogue **9**. As no corresponding linear brominated cystargamide had been present within the starting material, for this reaction, it is likely that this second product arises from hydrolysis of cystargamide either during the reaction or work up. ^19^F{^1^H} NMR analysis showed two fluorine peaks at *δ*_F_(D_2_O) –114.05 and –114.43 ppm with a ddd coupling consistent with that observed for compounds **36**, **60** and **61**. Attempts to separate the very small amounts of the two compounds by HPLC proved to be unsuccessful, as was obtaining sufficient material for ^1^H NMR analysis. It was notable though that the conditions that we had developed could be used to enable the functionalization of a challenging “new to nature” natural product even within a chemically demanding test-bed system with the added challenge of being produced only at very low level.

## Conclusions

We have for the first time demonstrated the Sonogashira diversification of free unprotected 5-, 6- and 7-bromo tryptophan and 7-iodotryptophan, and shown the reaction to be very high yielding with phenylacetylene **16**, fluorophenylacetylene **24**, 4-cyanophenylacetylene **25** and ethynylthiophene **26**. The reaction products can be isolated by reverse-phase HPLC and show no loss of stereochemical integrity under the reaction conditions. The reaction can even be carried out in the presence of oxygen with only a small decrease in the observed conversion.

Tryptophan is an essential amino acid, it is a key component in many natural products and its presence in proteins and peptides is key to their structural integrity, furthermore the intrinsic fluorescence of tryptophan often determines, or modulates, the spectrophotometric properties of a given peptide or protein, it therefore represents a very exciting target for diversification. Sonogashira cross-coupling of other amino acid residues including iodophenylalanine has been shown to be useful to tagging or modulating the properties of these peptides. We have demonstrated the first Sonogashira modification of halotryptophan as a component of a peptide, this represents a starting point from which milder conditions must be developed in order to extend the applicability of this reaction to sensitive proteins.

Using a simple tripeptide containing a 7-bromotryptophan at the N-terminus we have been able achieve quantitative Sonogashira cross-coupling with 3-fluorophenylacetylene **24** within 2 h. This is exciting as it opens up the potential for development for ^19^F labelling of peptides, with the short reaction times making this a potentially attractive tool for PET imaging studies.

We have for the first time generated a series of “new to nature” halocystargamides including 6-bromocystargamide **9**. The enzymes that mediate the biosynthesis of these compounds are strikingly flexible in enabling the incorporation of a wide range of halogens at different positions around the indole ring. Full processing including hydroxylation of the bromotryptophan observed within the natural product, demonstrates how readily, even the sterically bulky C–Br, is tolerated by the biosynthetic enzymes. 6-Bromocystargamide **9** represented the demanding natural product test-bed desired with which to challenge the cross-coupling conditions that we had developed. Though the compound is functionally elaborate and can only be accessed in very small quantities, it was possible to selectively functionalise the molecule with 3-fluorophenylacetylene **24** using the conditions that we had developed. Previously we had exemplified the utility of the more forgiving Suzuki–Miyaura reaction in the selective modification of natural products; here we show that the Sonogashira reaction can be used as a tool for GenoChemetics and for the selective diversification of “new to nature” natural products. Such an approach hold the potential to be a useful tool for diversification for Structure Activity Relationship (SAR) studies, for modulating the electronics of tryptophan and for tagging natural products and proteins with labels for fluorescence tracking, immobilisation tagging and PET imaging.
