# Supplementary material for: Sonogashira diversification of unprotected halotryptophans, halotryptophan containing tripeptides; and generation of a new to nature bromo-natural product and its diversification in water
Source: Chem Sci. 2016 Nov 11;8(3):2039–46. doi: 10.1039/c6sc04423a (PMC5398305; doi:10.1039/c6sc04423a)
Supplement: Supplementary file 1 [file SC-008-C6SC04423A-s001.pdf]

Electronic Supplementary Material (ESI) for Chemical Science.
This journal is © The Royal Society of Chemistry 2016

Sonogashira diversification of unprotected halotryptophans, halotryptophan containing tripeptides; and generation of a new to nature bromo-natural product and its diversification in water

M. J. Corr,[a] S. V. Sharma,[a] C. Pubill-Ulldemolins,[a] R. T. Bown,[a] P. Poirot,[b] D. R. Smith,[a] C. Cartmell,[a] A. Abou Fayad,[c] R. J. M. Goss[a*]

Supporting Information

General Synthetic Experimental **Page number 2**

Experimental For the synthesis of:
Sodium 2'-(Dicyclohexylphosphanyl)-2,6-diisopropyl-(1,1-biphenyl)-4-sulfonate (sXPhos) **12**[2] 4
Standard Reaction Procedure of 5-bromoindole **15** under reflux: 5
Microwave Reaction Procedure of 5-bromoindole **15**: 5
Standard Microwave Reaction Procedure for Tryptophans: 5
(*S*)-2-Amino-3-(5-(phenylethynyl)-1*H*-indol-3-yl)propanoic acid **19** 6
(*S*)-2-Amino-3-5-((3-fluorophenyl)ethynyl)-1*H*-indol-3-yl)propanoic acid **36** 7
(*S*)-2-Amino-3-5-((4-cyanophenyl)ethynyl)-1*H*-indol-3-yl)propanoic acid **39** 7
(*S*)-2-Amino-3-(5-(thiophen-3-ylethynyl)-1*H*-indol-3-yl)propanoic acid **42** 8
(*S*)-2-Amino-3-(5-(4-hydroxybut-1-yn-1-yl)-1*H*-indol-3-yl)propanoic acid **45** 9
(*S*)-2-Amino-3-(5-(4-phenylbut-1-yn-1-yl)-1*H*-indol-3-yl)propanoic acid **48** 9
(*S*)-2-Amino-3-(5-(octa-1,7-diyn-1-yl)-1*H*-indol-3-yl)propanoic acid **51** 10
(*S*)-2-Amino-3-(3-cyclohexylprop-1-yn-1-yl)-1*H*-indol-3-yl)propanoic acid **54** 10
N-Boc-7-bromo-*S*-tryptophan **66** 11
7-Bromo-L-tryptophan methyl ester hydrochloride **67** 12
N-Boc-Ala-Phe-OMe **68** 12
H-Ala-Phe-OMe.TFA **69** 13
N-Boc-Trp-(7-Br)-Ala-Phe-OMe **70** 14
H-Trp-(7-Br)-Ala-Phe-OH **59** 15
N-Boc-Trp-(7-Br)-Phe-OMe **71** 16
N-Boc-Ala-Trp-(7-Br)-Phe-OMe **72** 17
H-Ala-Trp-(7-Br)-Phe-OH **58** 18
((*S*)-2-((*S*)-2-Aminopropanamido)-3-(7-((3-fluorophenyl)ethynyl)-1*H*-indol-3-yl)propanoyl)-L-phenylalanine **60** 19
((S)-2-Amino-3-(7-((3-fluorophenyl)ethynyl)-1H-indol-3-yl)propanoyl) -L-alanyl-L-phenylalanine **61** 20
Enantiopurity analysis 21

General Biological Experimental **21**
Kitasatospora cystargenia **culturing, and precursor directed biosynthesis of new to nature bromo-cystargamide** **22**
Upsacled feeding with selected 6-bromotryptophan at 0.25 mM to 8L culture 30
LC-MSMS ananysis of cystargamide and novel cystargamide species 32
Cross-coupling of bromo-cystargamide 85
LC-MSMS ananysis of cross-coupled cystargamide 86

NMR spectra for small tryptophan, peptide derivatives and ^{19}F NMR analysis of cross-coupled cystargamide

General Chemical Experimental

All reagents were purchased from commercial suppliers and were used without further purification unless otherwise stated. Dry dichloromethane was dried and deoxygenated with an MBraun SPS-800 solvent purification system and the moisture content of the solvent was analysed using a Karl Fischer coulometer (Metler Toledo DL32).

Proton NMR (^{1}H), carbon NMR (^{13}C), phosphorus NMR (^{31}P) and fluorine NMR (^{19}F) were recorded on a Bruker Ascend 500 (500 MHz), Bruker 500 UltraShield (500 MHz), Bruker Ascend 700 (700 MHz), Bruker 400 UltraShield (400 MHz) or a Bruker UltraShield (300 MHz) spectrometer. Fluorine NMR were also recorded as proton decoupled ($^{19}F\{^{1}H\}$). Phosphorus NMR ($^{31}P\{^{1}H\}$) was recorded as proton decoupled. Using an HSQC experiment with multiplicity editing, the ^{13}C NMR signals were assigned to CH_3, CH_2, CH and C. The NMR experiments were carried out in deuterochloroform ($CDCl_3$) deuterated water (D_2O), deuterated DMSO (d_6-DMSO) or deuterated methanol (d_4-MeOH). The chemical shifts (δ) are quoted in parts per million (ppm). Multiplicities are abbreviated as s, singlet; d, doublet; t, triplet; q, quartet; m, multiplet; b, broad for the ^{1}H NMR, ^{19}F NMR, $^{19}F\{^{1}H\}$ NMR and ^{13}C NMR spectra. Coupling constants are reported in Hertz (Hz).

Flash chromatography was performed using Davisil silica gel LC60A (40-63 micron). Thin layer chromatography (TLC) was performed using aluminium sheets of silica gel 60 F254 and was visualised under a Mineralight model UVGL-58 lamp (254 nm). The plates were developed with acidic methanolic vanillin solutions, ethanolic phosphomolybdic acid solutions or basic potassium permanganate solutions.

High and low resolution mass spectra were recorded at the University of St Andrews on a Waters Micromass LCT time of flight mass spectrometer coupled to a Waters 2975 HPLC system or on an Orbitrap ELOS pro. Optical rotation values were recorded in methanol on a Perkin Elmer Model 341 Polarimeter using a Na/Hal lamp (589 nm) at 20 °C in a 1 dm polarimeter cell and are given in 10^{-1} deg cm^{2} g^{-1}. Freeze drying was carried out on a Scanvac CoolSafe™ freeze dryer. Microwave reactions were carried out on a Biotage Initiator+.

5-Bromotryptophan **18**, 5-chlorotryptophan **20**, 6-bromotryptophan **21**, 7-bromotryptophan **22** and 7-iodotryptophan **23** were prepared as described previously.[1]

HPLC purification was carried out on an XBridge Prep Phenyl 5 μm column (10x250 mm) on a Gilson HPLC system (Gilson 322 pump, Gilson UV/Vis-151 detector, Gilson 402 syringe

pump). UPLC analysis was carried out on a Waters Acquity H-Class UPLC system fitted with an Acquity UPLC BEH C18 column (1.7 µm, 2.1x50 mm).

For purification of the cross coupling products from 5-bromotryptophan **18**, the following program was used: 10% acetonitrile/water for 5 min, 10% acetonitrile/water to 60% acetonitrile/water over 21 minutes, 60% acetonitrile/water to 90% acetonitrile/water over 5 minutes, 90% acetonitrile/water to 10% acetonitrile/water over 5 min, 10% acetonitrile/water for 4 min. Total run time 40 minutes. Flowrate 5 mL/min, detection wavelength 254 nm.

For purification of the cross coupling products from tripeptides **58** and **59**, the following program was used: 10% acetonitrile/water for 5 min, 10% acetonitrile/water to 60% acetonitrile/water over 30 minutes, 60% acetonitrile/water to 90% acetonitrile/water over 5 minutes, 90% acetonitrile/water to 10% acetonitrile/water over 5 min, 10% acetonitrile/water for 4 min. Total run time 50 minutes. Flowrate 5 mL/min, detection wavelength 330 nm.

Purification of selected peptides was carried out on a Biotage Isolera Four using reverse-phase SNAP C18 12 g column cartridges. The purification was carried out using water/methanol on the following gradient: 12 mL/min elution, 2% methanol/water for 1.15 min, 2→15% methanol/water for 1.15 min, 15% methanol/water for 5.15 min, 15→95% methanol/water for 15 min, 95% methanol/water for 3.45 min. The collection wavelength was set a 254 nm.

UPLC analysis was carried out on a Waters Acquity H-Class UPLC system.

A 1:1 mixture of water:acetonitrile was freshly prepared before each set of cross coupling reactions. The solvent was degassed by bubbling nitrogen through the solutions for at least 15 minutes, then storing under nitrogen.

The IUPAC names of some compounds were obtained using ChemBioDraw Ultra (version 13.0.2.3012).

Sodium 2'-(Dicyclohexylphosphanyl)-2,6-diisopropyl-(1,1-biphenyl)-4-sulfonate (sXPhos) 12[2]

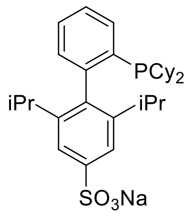

12

Sulfuric acid (1.0 mL) and fuming sulfuric acid (3.0 mL) were added to a suspension of XPhos (433 mg, 0.9 mmol, 1.0 eq) in dry DCM (3 mL) at 0 °C under nitrogen. The reaction was allowed to warm to r.t. and stirred for 24 h. The reaction mixture was cooled to 0 °C and crushed ice (10 g) was added. The reaction mixture was neutralised to pH 7 using sodium hydroxide solution (6 N, ~25 mL). The reaction mixture was extracted with DCM (3 x 50 mL) and the solvent was removed *in vacuo*. The residue was dissolved in cold methanol (20 mL), filtered, the filtrate collected and the solvent removed *in vacuo* to give sodium 2'-(dicyclohexylphosphanyl)-2,6-diisopropyl-(1,1-biphenyl)-4-sulfonate **12** (0.49 g, 100%) as a tan solid; ^{1}H NMR (500 MHz, d_4-MeOH) δ = 0.98 (6H, d, J(H,H)= 6.7 Hz, CH_3), 1.08-1.31 (16H, m, CH_3, CH_2), 1.55-1.89 (12H, m, CH, CH_2), 2.45-2.50 (2H, m, CH), 7.11-7.13 (1H, m, ArH), 7.38-7.47 (2H, m, ArH), 7.65-7.69 (3H, m, ArH); ^{13}C NMR (125 MHz, d_4-MeOH) δ = 23.1 (CH_3), 25.9 (CH_3), 27.5 (CH_2), 28.3 (d, J(C,P)= 8.9 Hz, CH_2), 28.5 (d, J(C,P)= 11.4 Hz, CH_2), 30.6 (d, J(C,P)= 12.0 Hz, CH_2), 31.9 (CH), 32.3 (d, J(C,P)= 16.1 Hz, CH_2), 35.6 (d, J(C,P)= 15.4 Hz, CH), 121.2 (CH), 128.0 (CH), 129.1 (CH), 132.3 (d, J(C,P)= 6.1 Hz, CH), 133.8 (CH), 137.2 (d, J(C,P)= 17.6 Hz, C), 142.7 (C), 145.5 (C), 147.5 (C), 147.7 (C), 148.3 (C); ^{31}P{^{1}H} NMR (162 MHz, d_4-MeOH) δ = -11.91 (1P, s); MS (ESI) 513 (100) [M-Na]$^-$; HRMS: *m/z* calcd for $C_{30}H_{42}O_3P_1S_1$ [M-Na]$^-$: 513.2598; found: 513.2596.

Standard Reaction Procedure of 5-bromoindole 15 under reflux:

5-Bromoindole **15** (20 mg, 0.1 mmol, 1.0 eq), *bis*(acetonitrile)dichloropalladium (II) (variable, see Table 1 in main text), sXPhos **12** (variable, see Table 1 in main text) and cesium carbonate (81 mg, 0.26 mmol, 2.5 eq) were added to a flask fitted with a reflux condenser under nitrogen. Degassed water/acetonitrile (1:1, 2 mL) was added to the flask, followed by the liquid alkyne substrate (variable, see Table 1 in main text). The reaction mixture was heated at 100 °C for 18 h. The reaction mixture was then cooled to r.t.

For analysis of the reaction conversions, 0.2 mL of reaction mixture was removed from the well-stirred reaction mixture and the solvent was removed *in vacuo*. The residue was dissolved in d_4-MeOH (0.7 mL) and analysed by ^{1}H NMR.

Microwave Reaction Procedure of 5-bromoindole 15:

5-Bromoindole **15** (20 mg, 0.1 mmol, 1.0 eq), *bis*(acetonitrile)dichloropalladium (II) (1.3 mg, 10 μmmol, 5 mol%), sXPhos **12** (8 mg, 30 μmmol, 15 mol%) and cesium carbonate (81 mg, 0.26 mmol, 2.5 eq) were added to a microwave tube, which was then fitted with a suba seal. The flask was evacuated and flushed with nitrogen three times. Degassed water/acetonitrile (1:1, 2 mL) and acetonitrile (1.0 mL) were added to the microwave tube, followed by the liquid alkyne substrate (33 *μ*L, 0.3 mmol, 3.0 eq). The tube was sealed and heated at 100 °C for 2 h using a microwave.

For analysis of the reaction conversion, 0.2 mL of reaction mixture was removed from the well-stirred microwave tube and the solvent was removed *in vacuo*. The residue was dissolved in d_4-MeOH (0.7 mL) and analysed by ^{1}H NMR.

Standard Microwave Reaction Procedure for Tryptophans:

The halo-tryptophan obtained from our previously reported procedure[1] (28 mg, 0.1 mmol, 1.0 eq for bromotryptophans, 33 mg 0.1 mmol, 1.0 eq for 7-iodotryptophan), *bis*(acetonitrile)dichloropalladium (II) (1.3 mg, 10 μmol, 5 mol%), sXPhos **12** (8 mg, 30 μmol, 15 mol%), (in the reaction with 4-cyanophenylacetylene **25**, 3.0 eq of the solid alkyne was also added at this stage) and cesium carbonate (81 mg, 0.26 mmol, 2.5 eq) were added to a microwave tube, which was then fitted with a suba seal. The flask was evacuated and flushed with nitrogen three times. Degassed water:acetonitrile (1:1, 2.0 mL) were added to the

microwave tube, followed by the liquid alkyne substrate (0.3 mmol, 3.0 eq). The tube was sealed and heated at 100 °C for 2 h using a microwave.

For analysis of the reaction conversions, 0.2 mL of reaction mixture was removed from the well-stirred microwave tube and the solvent was removed *in vacuo*. The residue was dissolved in d_4-MeOH (0.7 mL) and analysed by ^{1}H NMR.

For purification of specified reactions, the reaction mixture was cooled to r.t. and diluted with water (6.0 mL). The reaction mixture was centrifuged (13,000 rpm, 16060 g, for 5 min). The ultrafiltrate was collected and purified by HPLC purification, as described above, in 1 mL injections. The appropriate HPLC fractions (generally eluted at 18-20 minutes) were collected and the solvent removed *in vacuo*. The residue was re-suspended in water (50-100 mL) and lyophilised to give the product.

(*S*)-2-Amino-3-(5-(phenylethynyl)-1*H*-indol-3-yl)propanoic acid 19

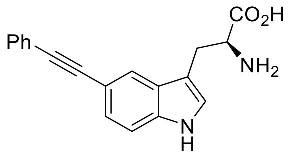

Using the standard procedure for tryptophans, 5-bromotryptophan **18** (28 mg, 0.1 mmol, 1.0 eq) and phenylacetylene **16** (33 μL, 0.3 mmol, 3.0 eq) gave (*S*)-2-amino-3-(5-(phenylethynyl)-1*H*-indol-3-yl)propanoic acid **19** (29.4 mg, 97%) as a fluffy, white solid; $[\alpha]_D$ -41.1° (c 0.27, MeOH); ^{1}H NMR (500 MHz, d_4-MeOH) δ = 3.03 (1H, dd, *J*(H,H)= 14.1, 8.4 Hz, CH_AH_B), 3.37 (1H, d, *J*(H,H)= 14.4 Hz, CH_AH_B), 3.68 (1H, bs, CH), 7.22 (1H, s, ArH), 7.26 (1H, dd, *J*(H,H)= 8.4, 1.5 Hz, ArH), 7.29-7.37 (4H, m, ArH), 7.48-7.51 (2H, m, ArH), 7.96 (1H, d, *J*(H,H)= 0.7 Hz, ArH); ^{13}C NMR (125 MHz, d_4-MeOH) δ = 31.0 (CH_2), 57.5 (CH), 87.4 (C), 92.5 (C), 111.9 (C), 112.5 (CH), 114.6 (C), 123.8 (CH), 125.6 (C), 125.96 (CH), 126.02 (CH), 128.7 (CH), 128.9 (C), 129.4 (CH), 132.2 (CH), 138.0 (C), 179.3 (CO); MS (ESI) 305 (100) $[M+H]^+$, 288 (80); HRMS: *m/z* calcd for $C_{19}H_{17}N_2O_2$ $[M+H]^+$: 305.1285; found: 305.1273.

The reaction was repeated to give 22.0 mg (73%) and 21.4 mg (70%) of the expected product **19** to give an average yield of 80% over three reactions.

(*S*)-2-Amino-3-5-((3-fluorophenyl)ethynyl)-1*H*-indol-3-yl)propanoic acid 36

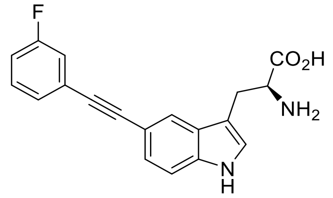

Using the standard procedure for tryptophans, 5-bromotryptophan **18** (28 mg, 0.1 mmol, 1.0 eq) and 3-fluorophenylacetylene **24** (34 μL, 0.3 mmol, 3.0 eq) gave (*S*)-2-amino-3-5-((3-fluorophenyl)ethynyl)-1*H*-indol-3-yl)propanoic acid **36** (32.2 mg, 100%) as a white solid; $[\alpha]_D$ -37.7° (c 0.26, MeOH); ^{1}H NMR (500 MHz, d_4-MeOH) δ = 3.14 (1H, dd, *J*(H,H)= 15.0, 8.9 Hz, CH_AH$_B$), 3.45-3.49 (1H, m, CH$_A$$H_B$), 3.81 (1H, dd, *J*(H,H)= 8.9, 3.8 Hz, CH), 7.07 (1H, ddd, *J*(H,H)= 9.0, 2.7 Hz, *J*(H,F)= 0.6 Hz, ArH), 7.22 (1H, ddd, *J*(H,H)= 2.7, 1.0 Hz, *J*(H,F)= 9.6 Hz, ArH), 7.25 (1H, s, ArH), 7.28-7.33 (2H, m, ArH), 7.35-7.40 (2H, m, ArH), 8.00 (1H, d, *J*(H,H)= 0.6 Hz, ArH); ^{13}C NMR (125 MHz, d_4-MeOH) δ = 29.0 (CH_2), 56.9 (CH), 86.3 (C), 93.5 (C), 110.6 (C), 112.7 (CH), 114.3 (C), 115.8 (d, *J*(C,F)= 21.4 Hz, CH), 118.6 (d, *J*(C,F)= 22.9 Hz, CH), 123.8 (C), 126.2 (CH), 126.4 (CH), 127.6 (d, *J*(C,F)= 9.5 Hz, C), 128.3 (d, *J*(C,F)= 2.7 Hz, CH), 128.7 (C), 131.3 (d, *J*(C,F)= 8.8 Hz, C), 138.2 (C) 164.0 (d, *J*(C,F)= 244.8 Hz, CF), 175.7 (CO); ^{19}F{^{1}H} NMR (470 MHz, d_4-MeOH) δ = -115.34 (1F, s); ^{19}F NMR (376 MHz, d_4-MeOH) δ = -115.31 (1F, dddd, *J*(H,F)= 9.6, 8.5, 5.8, 0.6 Hz); MS (ESI) 323 (60) $[M+H]^+$, 306 (50), 196 (80), 118 (100); HRMS: *m/z* calcd for $C_{19}H_{16}F_1N_2O_2$ $[M+H]^+$: 323.1190; found: 323.1190.

The reaction was repeated to give 32.2 mg (100%) and 28.9 mg (90%) of the expected product **36** to give an average yield of 97% over three reactions.

(*S*)-2-Amino-3-5-((4-cyanophenyl)ethynyl)-1*H*-indol-3-yl)propanoic acid 39

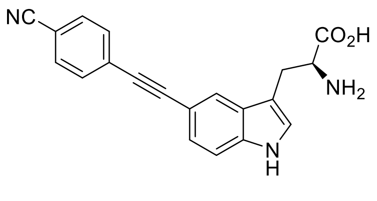

Using the standard procedure for tryptophans, 5-bromotryptophan **1**8 (28 mg, 0.1 mmol, 1.0 eq) and 4-cyanophenylacetylene **25** (38 mg, 0.3 mmol, 3.0 eq) gave (*S*)-2-amino-3-5-((3-cyanophenyl)ethynyl)-1*H*-indol-3-yl)propanoic acid **39** (26.1 mg, 79%) as a brown solid; $[\alpha]_D$ -37.7° (c 0.30, MeOH); ^{1}H NMR (500 MHz, d_4-MeOH) δ = 3.19 (1H, dd, *J*(H,H)= 15.2, 8.7 Hz, CH_AH$_B$), 3.50 (1H, dd, *J*(H,H)= 15.2, 4.0 Hz, CH$_A$$H_B$), 3.81 (1H, dd, *J*(H,H)= 8.7, 4.0 Hz, CH), 7.28 (1H, s, ArH), 7.32 (1H, dd, *J*(H,H)= 8.4, 1.3 Hz, ArH), 7.40 (1H, d, *J*(H,H)= 8.4 Hz,

ArH), 7.65-7.66 (2H, m, ArH), 7.72-7.74 (2H, m, ArH), 8.03 (1H, s, ArH); ^{13}C NMR (125 MHz, d_4-MeOH) δ = 28.4 (CH_2), 56.7 (CH), 86.4 (C), 97.3 (C), 110.4 (C), 111.8 (C), 112.8 (CH), 113.8 (C), 119.5 (C), 124.2 (CH), 126.3 (CH), 126.7 (C), 128.6 (C), 130.6 (C), 132.9 (CH), 133.4 (CH), 138.5 (C), 174.6 (CO); MS (ESI) 330 (100) $[M+H]^+$, 313 (90), 196 (90), 118 (100); HRMS: *m/z* calcd for $C_{20}H_{16}N_3O_2$ $[M+H]^+$: 330.1237; found: 330.1237.

The reaction was repeated to give 25.9 mg (79%) and 26.0 mg (79%) of the expected product **39** to give an average yield of 79% over three reactions.

(*S*)-2-Amino-3-(5-(thiophen-3-ylethynyl)-1*H*-indol-3-yl)propanoic acid 42

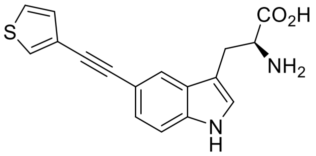

Using the standard procedure for tryptophans, 5-bromotryptophan **18** (28 mg, 0.1 mmol, 1.0 eq) and 3-ethynylthiophene **26** (30 μL, 0.3 mmol, 3.0 eq) gave (*S*)-2-amino-3-(5-(thiophen-3-ylethynyl)-1*H*-indol-3-yl)propanoic acid **42** (28.1 mg, 91%) as a fluffy, white solid; $[\alpha]_D$ -47.8° (c 0.23, MeOH); ^{1}H NMR (500 MHz, d_4-MeOH) δ = 3.03 (1H, dd, *J*(H,H)= 12.4, 7.9 Hz, C$H_A$$H_B$), 3.37 (1H, d, *J*(H,H)= 14.3 Hz, C$H_A$$H_B$), 3.68 (1H, bs, CH), 7.18 (1H, dd, *J*(H,H)= 5.0, 1.1 Hz, ArH), 7.21 (1H, s, ArH), 7.23 (1H, dd, *J*(H,H)= 8.4, 1.3 Hz, ArH), 7.33 (1H, d, *J*(H,H)= 8.4 Hz, ArH), 7.42 (1H, dd, *J*(H,H)= 5.0, 3.0 Hz, ArH), 7.54 (1H, dd, *J*(H,H)= 3.0, 1.1 Hz, ArH), 7.94 (1H, s, ArH); ^{13}C NMR (125 MHz, d_4-MeOH) δ = 30.9 (CH_2), 57.5 (CH), 82.6 (C), 91.7 (C), 111.8 (C), 112.5 (CH), 114.6 (C), 123.6 (CH), 124.5 (C), 125.9 (CH), 126.0 (CH), 1264 (CH), 128.54 (CH), 128.9 (C), 130.8 (CH), 137.9 (C), 179.5 (CO); MS (ESI) 311 (100) $[M+H]^+$, 294 (90); HRMS: *m/z* calcd for $C_{17}H_{15}N_2O_2S_1$ $[M+H]^+$: 311.0849; found: 311.0837.

The reaction was repeated to give 19.8 mg (64%) and 22.1 mg (71%) of the expected product **42** to give an average yield of 75% over three reactions.

(*S*)-2-Amino-3-(5-(4-hydroxybut-1-yn-1-yl)-1*H*-indol-3-yl)propanoic acid 45

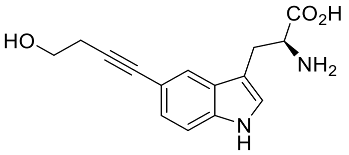

Using the standard procedure for tryptophans, 5-bromotryptophan **18** (28 mg, 0.1 mmol, 1.0 eq) and 3-butyn-1-ol **27** (23 μL, 0.3 mmol, 3.0 eq) gave (*S*)-2-amino-3-(5-(4-hydroxybut-1-yn-1-yl)-1*H*-indol-3-yl)propanoic acid **45** (21.4 mg, 79%) as a fluffy, white solid; $[\alpha]_D$ -30.0° (c 0.23, MeOH); ^{1}H NMR (500 MHz, d_4-MeOH) δ = 2.61 (2H, t, *J*(H,H)= 6.9 Hz, CH_2), 2.93 (1H, dd, *J*(H,H)= 14.3, 8.2 Hz, CH_AH_B), 3.26 (1H, dd, *J*(H,H)= 14.3, 4.3 Hz, CH_AH_B), 3.53-3.56 (1H, m, CH), 3.73 (2H, t, *J*(H,H)= 7.0 Hz, CH_2), 7.12 (1H, dd, *J*(H,H)= 8.4, 1.4 Hz, ArH), 7.16 (1H, s, ArH), 7.25 (1H, d, *J*(H,H)= 8.4 Hz, ArH), 7.80 (1H, s, ArH); ^{13}C NMR (125 MHz, d_4-MeOH) δ = 24.4 (CH_2), 32.1 (CH_2), 57.9 (CH), 62.1 (CH_2), 84.2 (C), 84.3 (C), 112.2 (CH), 112.5 (C), 115.1 (C), 123.6 (CH), 125.6 (CH), 126.0 (CH), 128.9 (C), 137.6 (C), 181.3 (CO); MS (ESI) 273 (100) $[M+H]^+$, 256 (30), 198 (50), 196 (100), 158 (70); HRMS: *m*/*z* calcd for $C_{15}H_{17}N_2O_3$ $[M+H]^+$: 273.1234; found: 273.1233.

The reaction was repeated to give 17.3 mg (64%) and 27.0 mg (99%) of the expected product **45** to give an average yield of 81% over three reactions.

(*S*)-2-Amino-3-(5-(4-phenylbut-1-yn-1-yl)-1*H*-indol-3-yl)propanoic acid 48

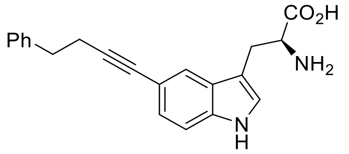

Using the standard procedure for tryptophans, 5-bromotryptophan **18** (28 mg, 0.1 mmol, 1.0 eq) and 4-phenyl-1-butyne **28** (42 μL, 0.3 mmol, 3.0 eq) gave (*S*)-2-amino-3-(5-(4-phenylbut-1-yn-1-yl)-1*H*-indol-3-yl)propanoic acid **48** (8.9 mg, 27%) as a light brown solid; $[\alpha]_D$ -24.8° (c 0.27, MeOH); ^{1}H NMR (500 MHz, d_4-MeOH) δ = 2.67 (2H, t, *J*(H,H)= 7.5 Hz, CH_2), 2.90 (2H, t, *J*(H,H)= 7.5 Hz, CH_2), 3.00 (1H, dd, *J*(H,H)= 14.3, 8.6 Hz, CH_AH_B), 3.33-3.37 (1H, m, CH_AH_B), 3.67 (1H, bs, CH), 7.08 (1H, dd, *J*(H,H)= 8.3, 1.3 Hz, ArH), 7.17-7.21 (2H, m, ArH), 7.25-7.31 (5H, m, ArH), 7.75 (1H, s, ArH); ^{13}C NMR (125 MHz, d_4-MeOH) δ = 23.1 (CH_2), 31.2 (CH_2), 37.1 (CH_2), 57.9 (CH), 84.4 (C), 87.4 (C), 112.0 (C), 112.7 (CH), 116.0 (CH), 123.7 (CH), 126.2 (CH), 126.6 (CH), 127.7 (CH), 129.2 (C), 129.8 (CH), 130.1 (CH), 138.1 (C) 142.9 (C), 179.5 (CO); MS (ESI) 333 (100) $[M+H]^+$, 316 (60); HRMS: *m*/*z* calcd for $C_{21}H_{21}N_2O_2$ $[M+H]^+$: 333.1598; found: 333.1591.

The reaction was repeated to give 8.3 mg (25%) and 8.7 mg (26%) of the expected product **48** to give an average yield of 26% over three reactions.

(*S*)-2-Amino-3-(5-(octa-1,7-diyn-1-yl)-1*H*-indol-3-yl)propanoic acid 51

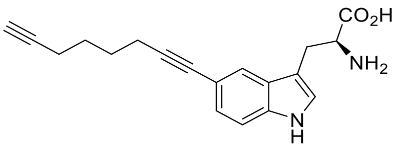

Using the standard procedure for tryptophans, 5-bromotryptophan **18** (28 mg, 0.1 mmol, 1.0 eq) and 1,7-octadiyne **29** (40 μL, 0.3 mmol, 3.0 eq) gave (*S*)-2-amino-3-(5-(octa-1,7-diyn-1-yl)-1*H*-indol-3-yl)propanoic acid **51** (17.2 mg, 56%) as a tan solid; $[\alpha]_D$ -23.1° (c 0.29, MeOH); ^{1}H NMR (500 MHz, d_4-MeOH) δ= 1.68-1.75 (4H, m, CH_2), 2.22-2.27 (3H, CH, CH_2), 2.42-2.45 (2H, m, CH_2), 2.98 (1H, dd, *J*(H,H)= 14.6, 8.5 Hz, CH_AH_B), 3.33-3.35 (1H, m, CH_AH_B), 3.65 (1H, bs, CH), 7.11 (1H, dd, *J*(H,H)= 8.4, 1.4 Hz, ArH), 7.17 (1H, s, ArH), 7.26 (1H, d, *J*(H,H)= 8.4 Hz, ArH), 7.28 (1H, s, ArH); ^{13}C NMR (125 MHz, d_4-MeOH) δ= 18.6 (CH_2), 19.6 (CH_2), 28.9 (CH_2), 29.2 (CH_2), 31.0 (CH_2), 57.5 (CH), 69.7 (CH), 83.5 (C), 84.8 (C), 86.9 (C), 111.6 (C), 112.2 (CH), 115.5 (CH), 123.2 (CH), 125.7 (CH), 126.1 (CH), 128.7 (C) 137.5 (C), 179.4 (CO); MS (ESI) 309 (100) $[M+H]^+$, 292 (70); HRMS: *m*/*z* calcd for $C_{19}H_{21}N_2O_2$ $[M+H]^+$: 309.1598; found: 309.1586.

The reaction was repeated to give 15.0 mg (49%) and 16.0 mg (52%) of the expected product **51** to give an average yield of 52% over three reactions.

(*S*)-2-Amino-3-(3-cyclohexylprop-1-yn-1-yl)-1*H*-indol-3-yl)propanoic acid 54

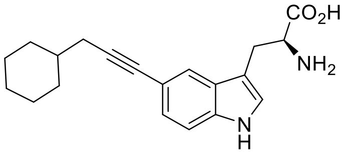

Using the standard procedure for tryptophans, 5-bromotryptophan **18** (28 mg, 0.1 mmol, 1.0 eq) and 3-cyclohexyl-1-propyne **30** (43 μL, 0.3 mmol, 3.0 eq) at 100 °C for 8 h gave (*S*)-2-amino-3-(3-cyclohexylprop-1-yn-1-yl)propanoic acid **54** (8 mg, ~25%) as a fluffy, white solid that contained the sXPhos **12** ligand as an inseparable impurity; ^{1}H NMR (500 MHz, d_4-MeOH) δ= 1.10 (2H, dtt, *J*(H,H)= 12.5, 12.4, 3.1 Hz, CH_2), 1.17-1.43 (2H, m, CH_2), 1.50-1.58 (1H,

m, CH), 1.68-1.84 (2H, m, CH_2), 1.90-1.95 (2H, m, CH_2), 2.29 (2H, d, J(H,H)= 6.7 Hz, CH_2), 2.93 (1H, dd, J(H,H)= 14.2, 8.4 Hz, CH_AH_B), 3.328-3.30 (1H, m, CH_AH_B), 3.59 (1H, s, CH), 7.09 (1H, dd, J(H,H)= 8.4, 1.4 Hz, ArH), 7.16 (1H, s, ArH), 7.25 (1H, d, J(H,H)= 8.4 Hz, ArH), 7.76 (1H, s, ArH); ^{13}C NMR (125 MHz, d_4-MeOH) δ = 27.0 (CH_2), 27.4 (CH_2), 27.5 (CH_2), 31.7 (CH_2), 33.9 (CH_2), 39.2 (CH), 57.8 (CH), 84.2 (C), 86.1 (C), 112.1 (CH), 115.6 (C), 123.2 (CH), 125.6 (CH), 126.0 (CH), 128.8 (C), 137.4 (C), 147.9 (C), 180.7 (CO); MS (ESI) 325 (100) $[M+H]^+$, 308 (60); HRMS: m/z calcd for $C_{20}H_{25}N_2O_2$ $[M+H]^+$: 325.1911; found: 325.1890.

N-Boc-7-bromo-S-tryptophan 66

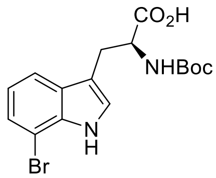

A suspension of 7-bromo-S-tryptophan **22** (100 mg, 0.35 mmol, 1.0 eq) and di-*t*-butyl dicarbonate (92 mg, 0.42 mmol, 1.2 eq) in 1,4-dioxane-water (1:1, 2 mL) was cooled to 0 °C. Aqueous KOH (1 M, 0.45 mL, 0.45 mmol, 1.25 eq) was added dropwise. The mixture was stirred for 4 h while warming to room temperature. The reaction was diluted with water (10 mL) and extracted with diethyl ether (2 x 10 mL). The aqueous layer was cooled in an ice-bath and the pH was adjusted to 2 using 1 M HCl. The resultant white suspension was extracted using ethyl acetate (3 x 10 mL). The combined organic layers were dried over anhydrous Na_2SO_4 and the solvent was removed *in vacuo* to give *N*-Boc-7-bromo-*S*-tryptophan **66** (120 mg, 89%) as a white, waxy solid that was used without further purification; ^{1}H NMR (300 MHz, d_4-MeOH) δ = 1.37 (9H, s, CH_3), 3.11 (1H, dd, J(H,H)= 14.6, 7.8 Hz, CH_AH_B), 3.31 (1H, dd, J(H,H)= 14.9, 5.1 Hz, CH_AH_B), 4.43 (1H, dd, J(H,H)= 7.8, 5.1 Hz, CH), 6.94 (1H, d, J(H,H)= 7.7 Hz, ArH), 7.17 (1H, s, ArH), 7.26 (1H, d, J(H,H)= 7.5 Hz, ArH), 7.56 (1H, dd, J(H,H)= 7.9, 0.7 Hz, ArH), 10.55 (1H, bs, NH); ^{13}C NMR (125 MHz, d_4-MeOH) δ = 27.3 (CH_3), 24.7 (CH_2), 54.4 (CH), 79.2 (C), 104.1 (C), 111.18 (C), 111.23 (C), 117.5 (CH), 119.6 (CH), 123.5 (CH), 124.2 (CH), 124.3 (CH), 129.1 (C), 156.3 (CO), 174.3 (CO); MS (ESI) 407 (100) $[M(^{81}Br)+Na]^+$, 405 (100) $[M(^{79}Br)+Na]^+$; HRMS: m/z calcd for $C_{16}H_{19}Br_1N_2Na_1O_4$ $[M(^{79}Br)+Na]^+$: 405.0420; found: 405.0415.

7-Bromo-S-tryptophan methyl ester hydrochloride 67

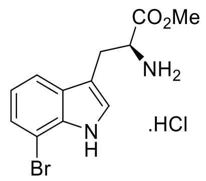

Thionyl chloride (290 µL, 4 mmol, 4.0 eq) was added to a flask containing dry methanol (10 mL) at 0 °C under argon. After stirring for 10 min, 7-bromo-*S*-tryptophan **22** (285 mg, 1 mmol, 1.0 eq) was added. The reaction was stirred overnight while allowing it to warm to r.t. The solvent was removed *in vacuo* to give 7-bromo-*S*-tryptophan methyl ester hydrochloride **67** (330 mg, 100%) as an off white hydrochloride salt that was used without further purification.

NMR on the HCl salt (d_6-DMSO) revealed broad signals. Hence, an analytical sample was obtained by desalting with dilute $NaHCO_3$ solution and extraction with ethyl acetate. Drying ($MgSO_4$) and evaporation of the solvent afforded the free base suitable for NMR in $CDCl_3$; ^1H NMR (400 MHz, $CDCl_3$) δ = 3.08 (1H, dd, *J*(H,H)= 14.4, 7.4 Hz, CH_AH_B), 3.27 (1H, ddd, *J*(H,H)= 14.4, 5.0, 0.7 Hz, CH_AH_B), 3.73 (3H, s, CH_3), 3.85 (1H, dd, *J*(H,H)= 7.4, 5.0 Hz, CH), 7.03 (1H, d, *J*(H,H)= 7.8 Hz, ArH), 7.15 (1H, d, *J*(H,H)= 2.2 Hz, ArH), 7.37 (1H, dd, *J*(H,H)= 7.7, 0.7 Hz, ArH), 7.58 (1H, d, *J*(H,H)= 7.9 Hz, ArH), 8.37 (1H, bs, NH); ^{13}C NMR (100 MHz, $CDCl_3$) δ = 30.9 (CH_2), 52.2 (CH_3), 55.1 (CH), 105.0 (C), 112.7 (C), 118.2 (CH), 120.9 (CH), 123.6 (CH), 124.7 (CH), 128.9 (C), 135.1 (C), 175.8 (CO); MS (ESI) 299 (100) $[M(^{81}Br)+H]^+$, 297 (100) $[M(^{79}Br)+H]^+$, 282 (60); HRMS: *m/z* calcd for $C_{12}H_{14}Br_1N_2O_2$ $[M(^{79}Br)+H]^+$: 297.0233; found: 297.0228.

***N*-Boc-Ala-Phe-OMe 68**

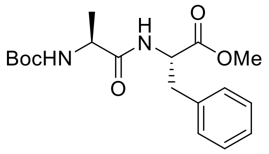

EDC.HCl (536 mg, 2.8 mmol, 1.2 eq) and HOBt (375mg, 2.8 mmol, 1.2 eq) were added to a microwave tube containing Boc-Ala-OH (440 mg, 2.3 mmol, 1.0 eq) in DMF (4.6 mL). The mixture was stirred for 5 min, then DIPEA (1 mL, 5.8 mmol, 2.5 eq) was added and the reaction mixture was stirred for 10 min. H-Phe-OMe.HCl (500 mg, 2.3 mmol, 1.0 eq) was added in one portion and reaction vial was sealed. The reaction was heated in a microwave reactor at 60 °C for 20 min. After cooling r.t., the reaction mixture was diluted with ethyl acetate (25 mL) and washed with water (3 x 10 mL), dilute sodium bicarbonate solution (10 mL) and brine (10 mL), dried over anhydrous $MgSO_4$ and the solvent removed *in vauco* to give *N*-Boc-Ala-Phe-OMe

68 (750 mg, 90%) as a white, waxy solid that was used without further purification; ^{1}H NMR (300 MHz, CDCl$_3$) δ= 1.28 (3H, d, J(H,H)= 7.0 Hz, CH$_3$), 1.42 (9H, s, CH$_3$), 3.05 (1H, dd, J(H,H)= 13.8, 6.3 Hz, CH_AH$_B$), 3.13 (1H, dd, J(H,H)= 13.8, 5.9 Hz, CH$_A$$H_B$), 3.67 (3H, s, CH$_3$), 4.18 (1H, bs, CH), 4.83 (1H, q, J(H,H)= 6.2 Hz, CH), 5.27 (1H, bs, NH), 6.83 (1H, bs, NH), 7.03-7.15 (2H, m, ArH), 7.16-7.37 (3H, m, ArH); ^{13}C NMR (75 MHz, CDCl$_3$) δ= 18.4 (CH$_3$), 28.8 (CH$_3$), 37.9 (CH$_2$), 50.0 (CH), 52.3 (CH$_3$), 53.3 (CH), 79.9 (C), 127.0 (CH), 128.5 (CH), 129.3 (CH), 135.9 (C), 155.4 (CO), 171.8 (CO), 172.5 (CO).

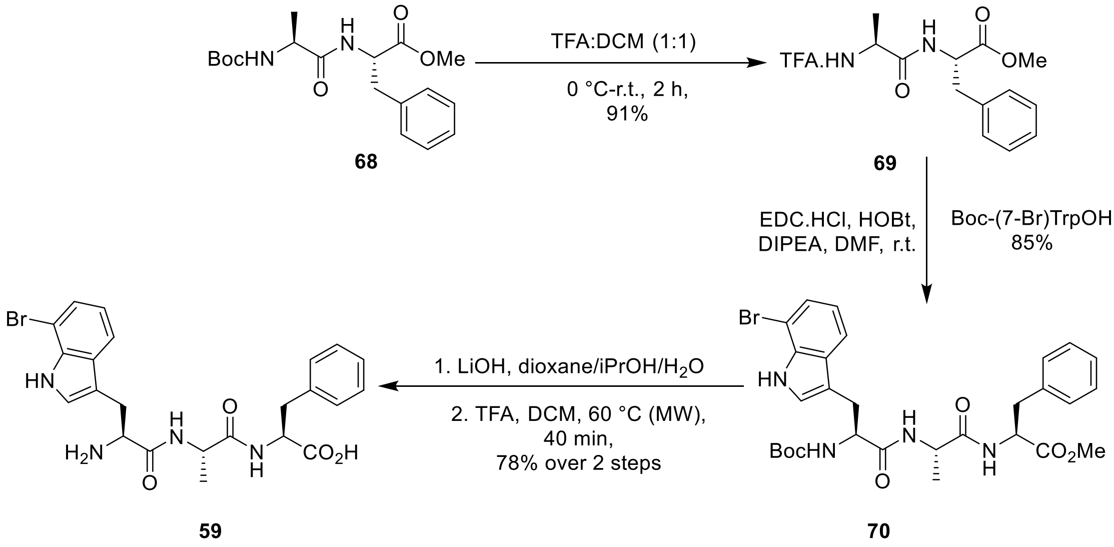

H-Ala-Phe-OMe.TFA 69

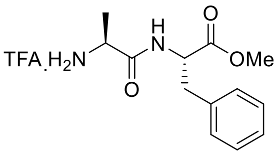

Water (0.2 mL) and TFA (2 mL) were added to a solution of *N*-Boc-Ala-Phe-OMe **68** (300 mg, 0.86 mmol, 1.0 eq) in DCM (3 mL) at 0 °C and stirred for 2 h, while warming to r.t. The solvent was removed *in vacuo*. The resulting pale, yellow oil was titurated with ice cold diethyl ether (3 x 5 mL) to give H-Ala-Phe-OMe.TFA **69** (310 mg, 91%) as an off white solid that was used without further purification; ^{1}H NMR (400 MHz, d_4-MeOH) δ= 1.50 (3H, d, J(H,H)= 7.1 Hz, CH$_3$), 3.02 (1H, dd, J(H,H)= 14.0, 9.2 Hz, CH_AH$_B$), 3.23 (1H, dd, J(H,H)= 14.0, 5.4 Hz, CH$_A$$H_B$), 3.73 (3H, s, CH$_3$), 3.88 (1H, q, J(H,H)= 7.1 Hz, CH), 4.73 (1H, dd, J(H,H)= 9.2, 5.4 Hz, CH), 7.24 (3H, dt, J(H,H)= 9.4, 3.2 Hz, ArH), 7.36-7.28 (2H, m, ArH).

N-Boc-Trp-(7-Br)-Ala-Phe-OMe 70

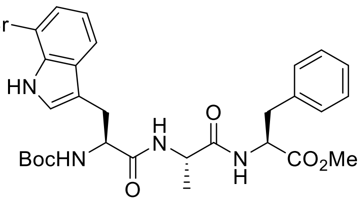

A solution of *N*-Boc-Trp-(7-Br)-OH **66** (220 mg, 0.57 mmol, 1.0 eq), H-Ala-Phe-OMe.TFA **69** (210 mg, 0.57 mmol, 1.0 eql) in DMF (4 mL) was cooled to 0 °C. Solid EDC.HCl (132 mg, 0.7 mmol, 1.2 eq) and HOBt (95 mg, 0.7 mmol, 1.2 eq) were added. The reaction mixture was stirred for 5 min, then DIPEA (0.28 mL, 1.6 mmol, 2.8 eq) was added dropwise. The reaction mixture was stirred for 20 h, while warming to r.t. The reaction mixture was diluted with ethyl acetate (20 mL) and washed successively with water (3 x 10 mL) and brine (10 mL). The combined organic layers were dried over anhydrous $MgSO_4$ and the solvent was removed *in vacuo*. Purification by column chromatography using silica gel (ethyl acetate:hexanes, 10-90%) gave *N*-Boc-Trp-(7-Br)-Ala-Phe-OMe **70** (300 mg, 85%) as a waxy, white solid; ^{1}H NMR (300 MHz, d_4-MeOH) δ= 1.25 (3H, d, J(H,H)= 6.9 Hz, CH_3), 1.35 (9H, s, CH_3), 2.91-3.27 (4H, m, CH_2), 3.66 (3H, s, CH_3), 4.26-4.44 (2H, m, CH, CH), 4.61 (1H, d, J(H,H)= 6.0 Hz, CH), 6.92 (1H, t, J(H,H)= 7.7 Hz, ArH), 7.13-7.21 (4H, m, ArH), 7.22-7.28 (3H, m, ArH), 7.59 (1H, d, J(H,H)= 7.8 Hz, ArH); ^{13}C NMR (75 MHz, d_4-MeOH) δ= 18.4 (CH_3), 28.4 (CH_3), 28.5 (CH_2), 37.9 (CH_2), 49.0 (CH), 52.5 (CH_3), 53.6 (CH), 55.0 (CH), 80.2 (C), 104.9 (C), 111.8 (C), 118.2 (CH), 128.8 (CH), 124.0 (CH), 124.6 (CH), 127.2 (CH), 128.7 (CH), 128.8 (CH), 129.3 (CH), 134.9 (C), 136.0 (C), 155.6 (CO), 171.6 (CO), 171.8 (CO), 171.9 (CO); MS (ESI) 639 (100) [M(^{81}Br)+Na]$^+$, 637 (100) [M(^{79}Br)+Na]$^+$, 617 (15) [M(^{81}Br)+H]$^+$, 615 (15) [M(^{79}Br)+H]$^+$; HRMS: *m/z* calcd for $C_{29}H_{36}Br_1N_4O_6$ [M(^{79}Br)+H]$^+$: 615.1813; found: 615.1799.

H-Trp-(7-Br)-Ala-Phe-OH 59

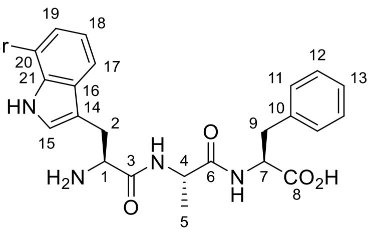

A solution of lithium hydroxide (14 mg, 0.6 mmol, 1.5 eq) in water (1 mL) was added to a solution of *N*-Boc-Trp-(7-Br)-Ala-Phe-OMe **70** (250 mg, 0.4 mmol, 10 eq) in 1,4-dioxane-iPrOH-water (1:1:0.5, 4 mL) at 0 °C. The reaction mixture was stirred vigorously until all the starting material has been consumed (as determined by TLC analysis). The reaction mixture was diluted with water (2 mL) and quenched by the addition of Amberlyst-120-H+ (to pH ~5-6). The resin was filtered and the solvent removed *in vacuo* to give the crude free acid (*N*-Boc-Ala-Phe-Trp-(7-Br)-OH). This intermediate was dissolved in DCM (3 mL) and transferred to a microwave vial. TFA (65 μL, 2 mmol, 5.0 eq) was added and reaction vial was sealed with an aluminium crimp cap. Reaction mixture was heated in a microwave reactor at 60 °C for 40 min. After cooling, the solvent was removed *in vacuo*. The residue was purified using gradient reversed phase chromatography (C-18, 12 g, water-MeOH, 5-95% gradient) to give H-Trp-(7-Br)-Ala-Phe-OH.TFA **59** (190 mg, 78% over 2 steps) as a white solid; ^{1}H NMR (500 MHz, d_4-MeOH) δ= 1.37 (3H, d, *J*(H,H)= 7.1 Hz, CH_3-C5), 3.00-3.03 (1H, dd, *J*(H,H)= 14.0, 8.1 Hz, CH_AH_B-C9), 3.12 (1H, dd, *J*(H,H)= 15.2, 9.0 Hz, CH_AH_B-C2), 3.21 (1H, dd, *J*(H,H)= 14.0, 5.2 Hz, CH_AH_B-C9), 3.36 (1H, dd, *J*(H,H)= 15.2, 5.2 Hz, CH_AH_B-C2), 4.11 (1H, dd, *J*(H,H)= 9.0, 5.2 Hz, CH-C1), 4.41 (1H, q, *J*(H,H)= 7.1 Hz, CH-C4), 4.63 (1H, dd, *J*(H,H)= 8.1, 5.2 Hz, CH-C7), 6.94 (1H, dd, *J*(H,H)= 7.8, 7.7 Hz, ArH-C18), 7.17 (1H, ddd, *J*(H,H)= 4.6, 4.1, 4.1 Hz, ArH-C15), 7.26-7.27 (5H, m, ArH-C11,C12,13), 7.30 (1H, d, *J*(H,H)= 7.5 Hz, ArH-C19), 7.66 (1H, d, *J*(H,H)= 7.9 Hz, ArH-C17); ^{13}C NMR (125 MHz, d_4-MeOH) δ= 18.3 (CH_3, C5), 28.9 (CH_2, C2), 38.3 (CH_2, C9), 50.5 (CH, C4), 54.6 (CH, C1), 55.3 (CH, C7), 105.8 (C, C20), 109.4 (C, C14), 118.6 (C, C17), 121.5 (CH, C18), 125.5 (CH, C19), 126.9 (CH, C13), 127.8 (CH, C15), 129.5 (CH, C10), 129.9 (C, C16), 130.5 (C, C11), 136.8 (C, C21), 138.4 (C, C10), 169.6 (CO, C3), 174.1 (CO, C6), 174.6 (CO, C8); HRMS: *m*/*z* calcd for $C_{23}H_{26}Br_1N_4O_4$ [M(^{79}Br)+H]$^+$: 501.1132; found: 501.1132.

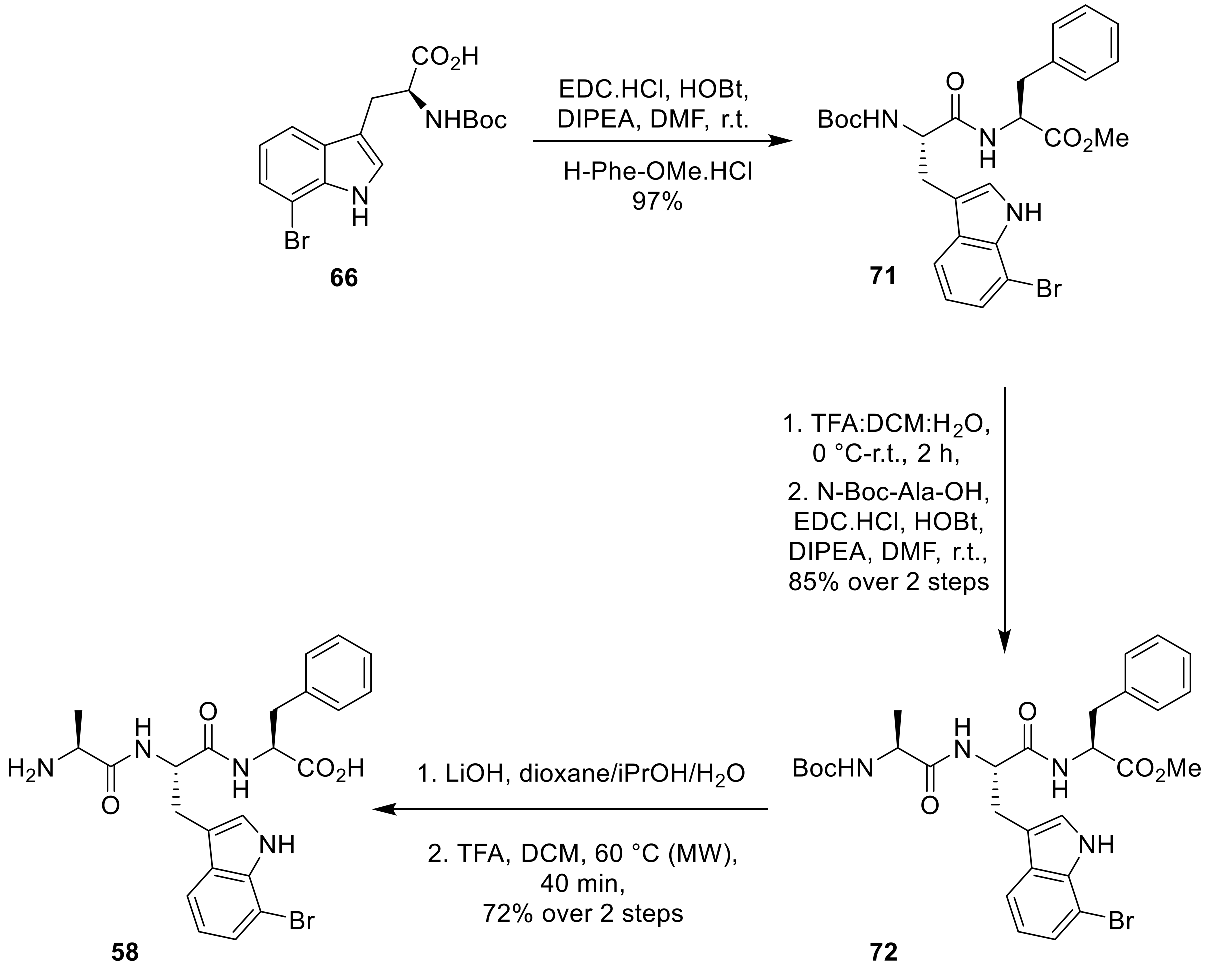

_N_-Boc-Trp-(7-Br)-Phe-OMe 71

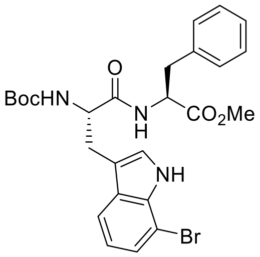

A solution of *N*-Boc-Trp-(7-Br)-OH **66** (135 mg, 0.37 mmol, 1.0 eq), H-Phe-OMe.HCl (85 mg, 0.4 mmol, 1.1 eq) in DMF (2 mL) was cooled to 0 °C. Solid EDC.HCl (83 mg, 0.43 mmol, 1.2 eq) and HOBt (58 mg, 0.43 mmol, 1.2 eq) were added. The reaction mixture was stirred for 5 min, then DIPEA (0.19 mL, 1.1 mmol, 3.0 eq) was added dropwise. The reaction mixture was stirred for 16 h, while warming to r.t. The reaction mixture was diluted with ethyl acetate (20 mL) and washed successively with water (3 x 10 mL) and brine (10 mL). The combined organic layers were dried over anhydrous $MgSO_4$ and the solvent was removed *in vacuo*. Purification by column chromatography using silica gel (MeOH:DCM, 0-10%) gave *N*-Boc-Trp-(7-Br)-

Phe-OMe **71** (194 mg, 97%) as a waxy, white solid; ^{1}H NMR (300 MHz, d_4-MeOH) δ= 1.36 (9H, s, CH_3), 2.86-3.06 (3H, m, CH, C$H_A$$H_B$), 3.15 (1H, dd, J(H,H)= 14.6, 5.9 Hz, C$H_A$$H_B$), 3.60 (3H, s, CH_3), 4.35 (1H, t, J(H,H)= 6.7 Hz, CH), 4.62 (1H, t, J(H,H)= 6.7 Hz, CH), 6.93 (1H, t, J(H,H)= 7.7 Hz, ArH), 7.06-7.17 (3H, m, ArH), 7.18-7.28 (4H, m, ArH), 7.57 (1H, d, J(H,H)= 7.8 Hz, ArH); ^{13}C NMR (75 MHz, d_4-MeOH) δ= 27.3 (CH_3), 27.9 (CH_2), 37.1 (CH_2), 51.3 (CH_3), 53.7 (CH), 55.2 (CH), 79.3 (C), 104.1 (C), 111.0 (C), 117.5 (CH), 119.6 (CH), 123.5 (C), 124.4 (CH), 126.5 (CH), 128.1 (CH), 128.9 (CH), 129.1 (CH), 134.9 (C), 136.4 (C), 156.0 (CO), 171.6 (CO), 172.8 (CO); MS (ESI) 568 (100) [M(^{81}Br)+Na]$^+$, 566 (100) [M(^{79}Br)+Na]$^+$, 546 (14), 544 (14); HRMS: m/z calcd for $C_{29}H_{36}Br_1N_4O_6$ [M(^{79}Br)+H]$^+$: 544.1442; found: 544.1431.

_N_-Boc-Ala-Trp-(7-Br)-Phe-OMe 72

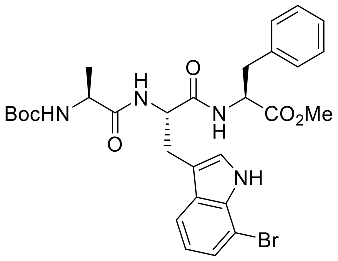

Water (0.1 mL) and TFA (1 mL) were added to a solution of *N*-Boc-Try-(7-Br)-Phe-OMe **71** (190 mg, 0.34 mmol, 1.0 eq) in DCM (2 mL) at 0 °C. The reaction mixture was stirred for 2 h, while warming to r.t. The solvent was removed *in vacuo*. The resultant pale yellow oil was triturated with cold diethyl ether (3 x 5 mL) to afford H-Trp-(7-Br)-Phe-OMe.TFA as a white foam.

A solution of H-Trp-(7-Br)-Phe-OMe.TFA (190 mg, 0.34 mmol, 1.0 eq) and *N*-Boc-Ala-OH (70 mg, 0.37 mmol, 1.1 eq) in DCM:DMF (1:1, 4 mL) was cooled to 0 °C. EDC.HCl (78 mg, 0.4 mmol, 1.2 eq) and HOBt (54 mg, 0.4 mmol, 1.2 eq) were added to the reaction mixture. DIPEA (0.2 mL, 1.2 mmol, 3.5 eq) was added dropwise and the reaction mixture was warmed to r.t. and stirred for 16 h. The reaction mixture was diluted with ethyl acetate (20 mL) and washed successively with water (3 x 10 mL) and brine (10 mL). The combined organic layers were dried over anhydrous $MgSO_4$ and the solvent was removed *in vacuo* to give *N*-Boc-Ala-Trp-(7-Br)-Phe-OMe **72** (190 mg, 85% over 2 steps) as a waxy, white solid that was used without further purification; ^{1}H NMR (500 MHz, d_6-DMSO) δ= 1.07 (3H, d, J(H,H)= 7.1 Hz, CH_3), 1.35 (9H, s, CH_3), 2.88-2.97 (2H, m, C$H_A$$H_B$, C$H_A$$H_B$), 3.00 (1H, dd, J(H,H)= 13.7, 6.1 Hz, C$H_A$$H_B$), 3.07 (1H, dd, J(H,H)= 14.7, 5.6 Hz, C$H_A$$H_B$), 3.56 (3H, s, CH_3), 3.86-4.00 (1H,

m, CH), 4.46 (1H, t, *J*(H,H)= 7.2 Hz, CH), 4.52-4.62 (1H, m, CH), 6.89 (1H, d, *J*(H,H)= 7.6 Hz, ArH), 6.93 (1H, t, *J*(H,H)= 7.7 Hz, ArH), 7.13-7.22 (4H, m, ArH), 7.23-7.31 (3H, m, ArH), 7.58 (1H, d, *J*(H,H)= 7.8 Hz, ArH); ^{13}C NMR (125 MHz, d_6-DMSO) δ = 18.3 (CH_3), 28.1 (CH_2), 28.5 (CH_3), 37.0 (CH_2), 50.0 (CH), 52.2 (CH_3), 53.0 (CH), 54.0 (CH), 78.7 (C), 104.3 (C), 111.3 (C), 118.4 (CH), 120.1 (CH), 123.8 (CH), 125.3 (CH), 127.0 (CH), 128.7 (C), 129.3 (CH), 129.5 (CH), 134.4 (C), 137.2 (C), 155.4 (CO), 171.5 (CO), 172.0 (CO), 172.8 (CO); MS (ESI) 639 (100) [M(^{81}Br)+Na]$^+$, 637 (100) [M(^{79}Br)+Na]$^+$, 617 (15) [M(^{81}Br)+H]$^+$, 615 (15) [M(^{79}Br)+H]$^+$; HRMS: *m*/*z* calcd for $C_{29}H_{36}Br_1N_4O_6$ [M(^{79}Br)+H]$^+$: 615.1813; found: 615.1800.

H-Ala-Trp-(7-Br)-Phe-OH 58

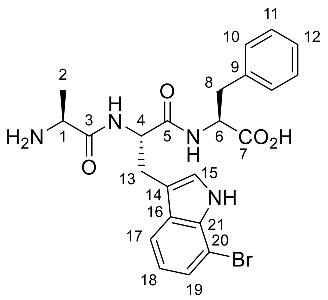

Following the procedure reported for **59**, *N*-Boc-Ala-Trp-(7-Br)-Phe-OMe **72** (170 mg, 0.27 mmol, 1.0 eq) gave H-Ala-Trp-(7-Br)-Phe-OH **58** (120 mg, 72% over 2 steps) as a white solid; ^{1}H NMR (500 MHz, d_4-MeOH) δ= 1.37 (3H, d, *J*(H,H)= 7.1 Hz, CH_3-C5), 3.00-3.03 (1H, dd, *J*(H,H)= 14.0, 8.1 Hz, CH_AH_B-C9), 3.12 (1H, dd, *J*(H,H)= 15.2, 9.0 Hz, CH_AH_B-C2), 3.21 (1H, dd, *J*(H,H)= 14.0, 5.2 Hz, CH_AH_B-C9), 3.36 (1H, dd, *J*(H,H)= 15.2, 5.2 Hz, CH_AH_B-C2), 4.11 (1H, dd, *J*(H,H)= 9.0, 5.2 Hz, CH-C1), 4.41 (1H, q, *J*(H,H)= 7.1 Hz, CH-C4), 4.63 (1H, dd, *J*(H,H)= 8.1, 5.2 Hz, CH-C7), 6.94 (1H, dd, *J*(H,H)= 7.8, 7.7 Hz, ArH-C18), 7.17 (1H, ddd, *J*(H,H)= 4.6, 4.1, 4.1 Hz, ArH-C15), 7.26-7.27 (5H, m, ArH-C11,C12,13), 7.30 (1H, d, *J*(H,H)= 7.5 Hz, ArH-C19), 7.66 (1H, d, *J*(H,H)= 7.9 Hz, ArH-C17); ^{13}C NMR (125 MHz, d_4-MeOH) δ= 18.3 (CH_3, C5), 28.9 (CH_2, C2), 38.3 (CH_2, C9), 50.5 (CH, C4), 54.6 (CH, C1), 55.3 (CH, C7), 105.8 (C, C20), 109.4 (C, C14), 118.6 (C, C17), 121.5 (CH, C18), 125.5 (CH, C19), 126.9 (CH, C13), 127.8 (CH, C15), 129.5 (CH, C10), 129.9 (C, C16), 130.5 (C, C11), 136.8 (C, C21), 138.4 (C, C10), 169.6 (CO, C3), 174.1 (CO, C6), 174.6 (CO, C8); HRMS: *m*/*z* calcd for $C_{23}H_{26}Br_1N_4O_4$ [M(^{79}Br)+H]$^+$: 501.1132; found: 501.1128.

((*S*)-2-((*S*)-2-Aminopropanamido)-3-(7-((3-fluorophenyl)ethynyl)-1*H*-indol-3-yl)propanoyl)-L-phenylalanine 60

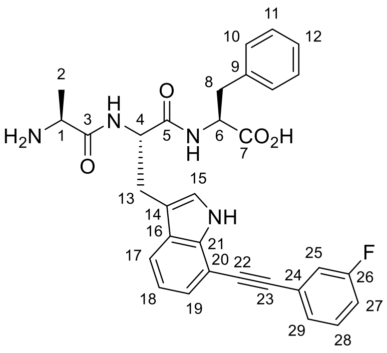

((S)-2-((S)-2-Aminopropanamido)-3-(7-bromo-1H-indol-3-yl)propanoyl)-L-phenylalanine **58** (4.0 mg, 8 μmol, 1.0 eq), sXPhos **12** (0.6 mg, 1.2 μmol, 15 mol%) and $CsCO_3$ (6.0 mg, 20 μmol, 2.5 eq) were added to a microwave tube and flushed with nitrogen. The microwave tube was sealed. A stock solution of *bis*(acetonitrile)dichloropalladium (II) (0.8 mg, 4 μmol, 0.5 eq) in degassed H_2O:CH_3CN (1:1, 1.0 mL) was prepared. The catalyst stock solution (0.1 mL, 0.4 μmol, 5 mol%) was injected into the microwave tube, followed by degassed H_2O:CH_3CN (1:1, 0.1 mL). 3-Fluorophenylacetylene **24** (10 μL, 80 μmol, 10.0 eq) was then injected into the microwave tube. The reaction mixture was well stirred, then heated at 100 °C for 2 h in a microwave. The reaction mixture was cooled to r.t. and diluted to 3 mL with H_2O:CH_3CN (9:1). The reaction mixture was centrifuged (13,000 rpm, 16060 g, 5 min) and the ultrafiltrate was collected and purified by HPLC purification as described above and lyophilised to give ((S)-2-((S)-2-aminopropanamido)-3-(7-((3-fluorophenyl)ethynyl)-1H-indol-3-yl)propanoyl)-L-phenylalanine **60** (2.0 mg, 47%) as a white solid; ^{1}H NMR (500 MHz, d_4-MeOH) δ = 1.24 (3H, d, J(H,H)= 6.9 Hz, CH_3-C2), 3.04 (1H, dd, J(H,H)= 13.5, 6.1 Hz, C$H_A$$H_B$-C8), 3.08 (1H, dd, J(H,H)= 14.6, 9.0 Hz, C$H_A$$H_B$-C13), 3.20 (1H, dd, J(H,H)= 13.5, 4.9 Hz, C$H_A$$H_B$-C8), 3.30-3.38 (1H, dd, J(H,H)= 14.6, 4.9 Hz, C$H_A$$H_B$-C13), 3.48 (1H, q, J(H,H)= 6.9 Hz, CH-C1), 4.45 (1H, dd, J(H,H)= 6.1, 4.9 Hz, CH-C6), 4.66 (1H, dd, J(H,H)= 9.0, 4.9 Hz, CH-C4), 7.06 (1H, dd, J(H,H)= 7.7, 7.6 Hz, ArH-C18), 7.10-7.21 (8H, m, ArH-C10,C11,C12,C15,C25), 7.30 (1H, dd, J(H,H)= 7.4, 0.7 Hz, ArH-C19), 7.38-7.44 (2H, m, ArH-C27,C28), 7.43-7.46 (1H, m, ArH-C29), 7.71 (1H, dd, J(H,H)= 8.0, 0.9 Hz, ArH-C17)- NOTE- the proton signal at 3.30-3.38 was obscured by the residual d_4-MeOH solvent peak, the position of the signal was determined by the 2D NMR experiments, with the coupling constants determined from the coupling constants of the protons on C4 and C13; ^{13}C NMR (125 MHz, d_4-MeOH) δ = 19.9 (CH_3, C2), 28.8 (CH_2, C13), 39.0 (CH_2, C8), 51.0 (CH, C1), 55.7 (CH, C4), 57.2 (CH, C6), 88.0 (C, C22), 92.4 (C, d, J(C,F)= 3.2 Hz, C23), 106.9 (C, C20), 112.1 (C, C14, C16), 116.3 (CH, d, J(C,F)= 21.8 Hz, C25), 119.0 (CH, d, J(C,F)= 23.1 Hz, C27), 121.0 (CH, C17), 125.5

(CH, C15), 126.3 (CH, C19), 127.0 (C, C24), 127.3 (CH, C12), 128.6 (CH, d, *J*(C,F)= 2.8 Hz, C29), 129.1 (CH, C10), 129.3 (C, C16), 130.8 (CH, C11), 131.4 (CH, d, *J*(C,F)= 8.8 Hz, C28), 138.2 (C, C21), 139.2 (C, C9), 164.0 (CF, d, *J*(C,F)= 244.4 Hz, C26), 172.6 (CO, C5), 176.7 (CO, C3), 177.3 (CO, C7); $^{19}F\{^1H\}$ NMR (376 MHz, d_4-MeOH) δ = -115.2 (1F, s); ^{19}F NMR (470 MHz, d_4-MeOH) δ = -115.2 (1F, ddd, *J*(H,F)= 9.5, 9.1, 5.6 Hz); MS (ESI) 541 (100) $[M+H]^+$; HRMS: *m*/*z* calcd for $C_{31}H_{30}F_1N_4O_4$ $[M+H]^+$: 541.2246; found: 541.2221.

((S)-2-Amino-3-(7-((3-fluorophenyl)ethynyl)-1H-indol-3-yl)propanoyl)-L-alanyl-L-phenylalanine 61

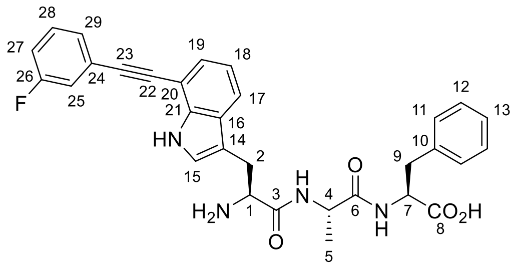

Using the same procedure as **60**, ((*S*)-2-Amino-3-(7-bromo-1*H*-indol-3-yl)propanoyl)-L-alanyl-L-phenylalanine **59** (4 mg, 8 μmol, 1.0 eq) gave ((*S*)-2-amino-3-(7-((3-fluorophenyl)ethynyl)-1*H*-indol-3-yl)propanoyl)-L-alanyl-L-phenylalanine **61** (4.3 mg, 100%) as a white solid; ^1H NMR (500 MHz, d_4-MeOH) δ = 1.15 (3H, d, *J*(H,H)= 7.1 Hz, CH_3-C5), 3.00 (1H, dd, *J*(H,H)= 13.6, 6.8 Hz, CH_AH_B-C9), 3.02 (1H, dd, *J*(H,H)= 14.7, 7.3 Hz, CH_AH_B-C2), 3.17-3.22 (2H, m, CH_AH_B-C2,C9), 3.68 (1H, dd, *J*(H,H)= 7.3, 5.2 Hz, CH-C1), 4.29 (1H, q, *J*(H,H)= 7.1 Hz, CH-C4), 4.45 (1H, dd, *J*(H,H)= 6.8, 4.9 Hz, CH-C7), 7.04 (1H, dd, *J*(H,H)= 7.7, 7.5 Hz, ArH-C18), 7.09-7.13 (2H, m, ArH-C15,C25), 7.17-7.20 (5H, m, ArH-C11,C12,C13), 7.31 (1H, d, *J*(H,H)= 7.2 Hz, ArH-C19), 7.38-7.41 (2H, m, ArH-C27,28), 7.43-7.46 (1H, m, ArH-C29), 7.69 (1H, d, *J*(H,H)= 7.9 Hz, ArH-C17); ^{13}C NMR (125 MHz, d_4-MeOH) δ = 18.0 (CH_3, C5), 31.4 (CH_2, C2), 39.1 (CH_2, C9), 50.6 (CH, C4), 56.5 (CH, C1), 57.2 (CH, C7), 88.0 (C, C22), 92.4 (C, d, *J*(C,F)= 3.0 Hz, C23), 107.0 (C, C20), 111.8 (C, C14, C16), 116.3 (CH, d, *J*(C,F)= 21.5 Hz, C25), 119.0 (CH, d, *J*(C,F)= 23.1 Hz, C27), 119.9 (CH, C18), 121.1 (CH, C17), 125.9 (CH, C13), 126.3 (CH, C19), 127.0 (C, d, *J*(C,F)= 9.3 Hz, C24), 127.3 (CH, C15), 128.6 (CH, d, *J*(C,F)= 2.9 Hz, C29), 129.1 (CH, C11), 130.8 (CH, C12), 131.3 (CH, d, *J*(C,F)= 8.8 Hz, C28), 138.3 (C, C21), 139.3 (C, C10), 163.9 (CF, d, *J*(C,F)= 244.8 Hz, C26), 173.7 (CO, C6), 176.8 (CO, C3), 177.6 (CO, C8); $^{19}F\{^1H\}$ NMR (470 MHz, d_4-MeOH) δ = -115.2 (1F, s); ^{19}F NMR (470 MHz, d_4-MeOH) δ = -115.2 (1F, ddd, *J*(H,F)=

9.4, 9.1, 5.8 Hz); MS (ESI) 541 (100) $[M+H]^+$; HRMS: *m*/*z* calcd for $C_{31}H_{30}F_1N_4O_4$ $[M+H]^+$: 541.2246; found: 541.2224.

Enantiopurity analysis

The enantiopurity of the cross coupled products was analysed using Marfey's Reagent (1-fluoro-2,4-dinitrophenyl-5-L-alanine amide, FDAA).

The tryptophan was dissolved in HCl solution (1 M, 10 mg/mL). An Eppendorf tube containing the tryptophan (50 µL), FDAA (1% *w*/*v* in actone, 100 µL) and $NaHCO_3$ solution (1M, 70 µL) was mixed thoroughly and incubated at 37 °C for 1 h. The reaction mixture was diluted (10 µL in 490 µL water) and centrifuged (13,000 rpm, 16060 g, 5 min) before UPLC analysis.

The UPLC analysis was carried out in acetonitrile/0.1% TFA in water at a gradient of 20% acetonitrile to 90% acetonitrile over 4.7 minutes. A 3:1 mixture of L-tryptophan to D-tryptophan was used as a standard. Two blank runs using acetone were conducted between each tryptophan sample. In all of the tryptophans analysed, only a single peak for the L-enantiomer was observed.

General Biological Experimental

Biological reagents and components for media, buffers and stock solutions were purchased from commercial suppliers, used without further purification and stored according to the supplier's instructions. Microorganisms were stored at -80 °C for long and short-term storage. Microorganisms were cultured under sterile conditions using a Faster BH-EN class II vertical laminar airflow cabinet and fermentation media was sterilised at 121 °C for 20 minutes at 1.3 bar in a Boxer Benchtop Denley autoclave prior to use. Alternatively, aqueous solutions of heat labile components were sterilised by passage through a 0.2 µm membrane. Culturing apparatus was sterilised by autoclaving as described above or alternatively, disposable pre-sterilised apparatus was used.

General apparatus: Pipetting of solutions and samples was done using LABNET Biopette autovclavable pipettes. Microbial cultures were incubated in a New Brunswick Scientific Innova 4300 incubator shaker, a New Brunswick Scientific I26 incubator shaker series, or Genlab incubator (static). pH measurements were taken using a Fisherbrand Hydrus 300 pH meter. Centrifugation was carried out using a Thermo Scientific IEC CL30R centrifuge.

***Kitasatospora cystargenia* culturing, and precursor directed biosynthesis of new to nature bromo-cystargamide**

Kitasatospora cystargenia NRRL-B16505 was obtained from the USDA agriculture research service culture collection. The strain was fermented in GY medium (10 g/L glucose, 10 g/L yeast extract) for 48 h at 28 °C (as previously reported by Kerr and coworkers)[3] and 200 rpm, in an incubator with a 2.54 cm throw. The mycelium from this culture was stored in 20% glycerol at -80 °C.

Small scale feeding experiments

In order to explore whether the halotryptophans could be incorporated too, we carried out small scale feeding experiments with 7, 6 and 5-chlorotryptophan and 7, 6 and5 -bromotryptophan at 0.25 and 1mM to both 4mL cultures in 24 deep well plates and 50mL cultures in 250mL Erlenmeyer flasks. The cultures were extracted and analysed by LCMSMS. Results demonstrated that all of the halotryptophans incorporated into cystargamide, generating new to nature halogenated analogues.

6Br-tryptophan 21 - Feeding at 0.25 mM scale

Detected Cystargamide Species	[M+H]+	Absorbance peak area[c]	Ratio compared to Cystargamide (WT)[d]
Cystargamide (WT)	954.4244	5923338	10.0
Linear cystargamide[a]	972.4349	3036620	5.1
Cystargamide no hydroxylated[b]	938.4294	607443	1.0
(6BrW) Brominated cystargamide no hydroxylated[b]	1016.3400	122182	0.2
(6Brw) Brominated cystargamide	1032.3349	834562	1.4

[a] The ester bond appeared to have been hydrolysed [b] That lacked the 5-hydroxy on the tryptophan [c] Average area from the reaction performed in triplicate [d] Calculated as: (Absorbance peak area species/Absorbance peak area cystargamide wild type (WT))*10

<u>**Ratios of the detected cystargamide species**</u>:

Cyclic cystargamide species : Linear cystargamide species = **3:1**

Brominated cystargamide species : Brominated linear cystargamide species = **1:0**

Cyclic cystargamide species : cyclic cystargamide species no hydroxylated= **9:1**

Brominated cyclic cystargamide species : Brominated cystargamide no hydroxylated= **7:1**

Analysis of the extract by LC-MS also revealed quantities of analogues of cystargamide containing alanine [ALA] instead of glycine. In this case, only linear forms in which the ester bond appeared to have been hydrolysed were detected.

Detected Cystargamide[ALA] species	[M+H]+	Absorbance peak area[c]	Ratio compared to Cystargamide (WT)[d]
Linear cystargamide[ALA][a]	986.4500	9574644	16.2
(6BrW) Brominated linear cystargamide[ALA] no hydroxylated[a],[b]	1048.3662	272892	0.5
(6BrW) Brominated linear cystargamide[ALA][a]	1064.3611	1312663	2.2

[a] The ester bond appeared to have been hydrolysed [b] That lacked the 5-hydroxy on the tryptophan [c] Average area from the reaction performed in triplicate [d] Calculated as: (Absorbance peak area species/Absorbance peak area cystargamide wild type (WT))*10

6Br-tryptophan 21 -Feeding at 1 mM scale

Detected Cystargamide species	[M+H]+	Absorbance peak area[c]	Ratio compared to Cystargamide (WT)[d]
Cystargamide (WT)	954.4244	9135391	10.0
Linear cystargamide[a]	972.4349	4175252	4.6
Cystargamide no hydroxylated[b]	938.4294	54982	0.1
(6BrW) Brominated cystargamide no hydroxylated[b]	1016.3400	1035165	1.1
(6BrW) Brominated cystargamide	1032.3349	1928082	2.1

[a] The ester bond appeared to have been hydrolysed [b] That lacked the 5-hydroxy on the tryptophan [c] Average area from the reaction performed in triplicate [d] Calculated as: (Absorbance peak area species/Absorbance peak area cystargamide wild type (WT))*10

<u>Ratios of the detected cystargamide species</u>:

Cyclic cystargamide species : Linear cystargamide species = **3:1**

Brominated cystargamide species : Brominated linear cystargamide species = **1:0**

Cyclic cystargamide species : cyclic cystargamide species no hydroxylated= **10.4:1**

Brominated cyclic cystargamide species : Brominated cystargamide no hydroxylated= **2:1**

Analysis of the extract by LC-MS also revealed quantities of analogues of cystargamide containing alanine [ALA] instead of glycine. In this case, only linear forms in which the ester bond appeared to have been hydrolysed were detected.

Detected Cystargamide[ALA] species	[M+H]+	Absorbance peak area[c]	Ratio compared to Cystargamide (WT)[d]
Linear cystargamide[ALA][a]	986.4500	15397915	16.9
Linear cystargamide[ALA] no hydroxylated[a],[b]	970.4557	60678	0.1
(6Br) Brominated linear cystargamide[ALA] no hydroxylated[a],[b]	1048.3662	1907165	2.1
(6Br) Brominated linear cystargamide[ALA][a]	1064.3611	2939735	3.2

[a] The ester bond appeared to have been hydrolysed [b] That lacked the 5-hydroxy on the tryptophan [c] Average area from the reaction performed in triplicate [d] Calculated as: (Absorbance peak area species/Absorbance peak area cystargamide wild type (WT))*10

5Br-tryptophan- Feeding at 1 mM scale

Detected Cystargamide species	[M+H]$^+$	Absorbance peak area[c]	Ratio compared to Cystargamide (WT)[d]
Cystargamide (WT)	954.4244	17929225	10.0
Linear cystargamide[a]	972.4349	600331	3.8
Cystargamide no hydroxylated[b]	938.4294	70645	0.3
(5BrW) Brominated cystargamide no hydroxylated[b]	1016.3400	312674	0.1

[a] The ester bond appeared to have been hydrolysed [b] That lacked the 5-hydroxy on the tryptophan [c] Average area from the reaction performed in triplicate [d] Calculated as: (Absorbance peak area species/Absorbance peak area cystargamide wild type (WT))*10

Ratios of the detected cystargamide species:

Cyclic cystargamide species : Linear cystargamide species = **3:1**

Brominated cystargamide species : Brominated linear cystargamide species = **1:0**

Cyclic cystargamide species : cyclic cystargamide species no hydroxylated= **21:1**

Brominated cyclic cystargamide species : Brominated cystargamide no hydroxylated= 0**:1**

Analysis of the extract by LC-MS also revealed quantities of analogues of cystargamide containing alanine [ALA] instead of glycine. In this case, only linear forms in which the ester bond appeared to have been hydrolysed were detected.

Detected Cystargamide[ALA] species	[M+H]$^+$	Absorbance peak area[c]	Ratio compared to Cystargamide (WT)[d]
Linear cystargamide[ALA][a]	986.4500	9536071	10.8
(5BrW) Brominated linear cystargamide[ALA] no hydroxylated[a],[b],[e]	1048.3662	266591	0.3

[a] The ester bond appeared to have been hydrolysed [b] That lacked the 5-hydroxy on the tryptophan [c] Average area from the reaction performed in triplicate [d] Calculated as: (Absorbance peak area species/Absorbance peak area cystargamide wild type (WT))*10 [e] MS2 data was not recorded due to interfering masses

7Br-tryptophan-Feeding at 1 mM scale

Detected Cystargamide species	[M+H]+	Absorbance peak area[c]	Ratio compared to Cystargamide (WT)[d]
Cystargamide (WT)	954.4244	3227118	10.0
Linear cystargamide[a]	972.4349	11589642	35.9
Cystargamide no hydroxylated[b]	938.4294	225555	0.7
(7BrW) Brominated cystargamide	1032.3349	348435	1.1

[a] The ester bond appeared to have been hydrolysed [b] That lacked the 5-hydroxy on the tryptophan [c] Average area from the reaction performed in triplicate [d] Calculated as: (Absorbance peak area species/Absorbance peak area cystargamide wild type (WT))*10

<u>**Ratios of the detected cystargamide species**</u>:

Cyclic cystargamide species : Linear cystargamide species = **0.3:1**

Brominated cystargamide species : Brominated linear cystargamide species = **1:0**

Cyclic cystargamide species : cyclic cystargamide species no hydroxylated= **16:1**

Brominated cyclic cystargamide species : Brominated cystargamide no hydroxylated= **1:0**

Analysis of the extract by LC-MS also revealed quantities of analogues of cystargamide containing alanine [ALA] instead of glycine. In this case, only linear forms in which the ester bond appeared to have been hydrolysed were detected.

Detected Cystargamide[ALA] species	[M+H]+	Absorbance peak area[c]	Ratio compared to Cystargamide (WT)[d]
Linear cystargamide[ALA][a]	986.4500	26865004	23.2
Linear cystargamide[ALA] no hydroxylated[a],[b]	970.4557	1320235	1.1
(7BrW) Brominated linear cystargamide[ALA] no hydroxylated[a],[b],[e]	1048.3662	576202	0.5
(7BrW) Brominated linear cystargamide[ALA][a],[d]	1064.3611	2078672	1.8

[a] The ester bond appeared to have been hydrolysed [b] That lacked the 5-hydroxy on the tryptophan [c] Average area from the reaction performed in triplicate [d] Calculated as: (Absorbance peak area species/Absorbance peak area cystargamide wild type (WT))*10 [e] MS2 data was not recorded due to interfering masses

5Cl-tryptophan-Feeding at 1 mM scale

Detected Cystargamide species	$[M+H]^+$	Absorbance peak area[c]	Ratio compared to Cystargamide (WT)[d]
Cystargamide (WT)	954.4244	1590311	10.0
Linear cystargamide[a]	972.4349	714633	4.5
Cystargamide no hydroxylated[b]	938.4294	155629	1.0
(5ClW) Chlorinated linear cystargamide no hydroxylated[a],[b]	990.401	191149	1.2

[a] The ester bond appeared to have been hydrolysed [b] That lacked the 5-hydroxy on the tryptophan [c] Average area from the reaction performed in triplicate [d] Calculated as: (Absorbance peak area species/Absorbance peak area cystargamide wild type (WT))*10

Ratios of the detected cystargamide species:

Cyclic cystargamide species : Linear cystargamide species = **1.93:1**

Chlorinated cystargamide species : Chlorinated linear cystargamide species = **0:1**

Cyclic cystargamide species : cyclic cystargamide species no hydroxylated= **10:1**

Chlorinated cyclic cystargamide species : Chlorinated cystargamide no hydroxylated= **0:1**

Analysis of the extract by LC-MS also revealed quantities of analogues of cystargamide containing alanine [ALA] instead of glycine. In this case, only linear forms in which the ester bond appeared to have been hydrolysed were detected.

Detected Cystargamide[ALA] species	$[M+H]^+$	Absorbance peak area[c]	Ratio compared to Cystargamide (WT)[d]
Linear cystargamide[ALA][a]	986.4500	2719153	17.1
Linear cystargamide[ALA] no hydroxylated[a],[b]	970.4557	13496	0.1
(5ClW) Chlorinated linear cystargamide[ALA] no hydroxylated[a],[b]	1004.4167	806781	5.1

[a] The ester bond appeared to have been hydrolysed [b] That lacked the 5-hydroxy on the tryptophan [c] Average area from the reaction performed in triplicate [d] Calculated as: (Absorbance peak area species/Absorbance peak area cystargamide wild type (WT))*10

6Cl-tryptophan-Feeding at 1 mM scale

Detected Cystargamide species	[M+H]+	Absorbance peak area[c]	Ratio compared to Cystargamide (WT)[d]
Cystargamide (WT)	954.4244	699261	10.0
Linear cystargamide[a]	972.4349	1326172	19.0
Cystargamide no hydroxylated[b]	938.4294	23596	0.3
(6ClW) Chlorinated cystargamide no hydroxylated[b]	972.3905	1277854	18.3
(6ClW) Chlorinated linear cystargamide no hydroxylated[a],[b]	990.401	1440729	20.6
(6ClW) Chlorinated cystargamide	988.3854	4530292	64.8
(6ClW) Chlorinated linear cystargamide[a]	1006.3960	7364948	100.5

[a] The ester bond appeared to have been hydrolysed [b] That lacked the 5-hydroxy on the tryptophan [c] Average area from the reaction performed in triplicate [d] Calculated as: (Absorbance peak area species/Absorbance peak area cystargamide wild type (WT))*10

<u>**Ratios of the detected cystargamide species**</u>:

Cyclic cystargamide species : Linear cystargamide species = **0.6:1**

Chlorinated cystargamide species : Chlorinated linear cystargamide species = **0.1:1**

Cyclic cystargamide species : cyclic cystargamide species no hydroxylated= **4:1**

Chlorinated cyclic cystargamide species : Chlorinated cystargamide no hydroxylated= **4:1**

Analysis of the extract by LC-MS also revealed quantities of analogues of cystargamide containing alanine [ALA] instead of glycine. In this case, only linear forms in which the ester bond appeared to have been hydrolysed were detected.

Detected Cystargamide[ALA] species	[M+H]+	Absorbance peak area[c]	Ratio compared to Cystargamide (WT)[d]
Linear cystargamide[ALA][a]	986.4500	4926993	70.5
Linear cystargamide[ALA] no hydroxylated[a],[b]	970.4557	102532	1.5
(6ClW) Chlorinated linear cystargamide[ALA] no hydroxylated[a],[b]	1004.4167	7641792	109.3
(6ClW) Chlorinated linear cystargamide[ALA][a]	1020.4116	24151097	345.4

[a] The ester bond appeared to have been hydrolysed [b] That lacked the 5-hydroxy on the tryptophan [c] Average area from the reaction performed in triplicate [d] Calculated as: (Absorbance peak area species/Absorbance peak area cystargamide wild type (WT))*10

7Cl-tryptophan-Feeding at 1 mM scale

Detected Cystargamide species	[M+H]+	Absorbance peak area[c]	Ratio compared to Cystargamide (WT)[d]
Cystargamide (WT)	954.4244	2106653	10.0
Linear cystargamide[a]	972.4349	1291560	6.1
Cystargamide no hydroxylated[b]	938.4294	300104	1.4
(7ClW) Chlorinated cystargamide no hydroxylated[b]	972.3905	668116	3.2
(7ClW) Chlorinated linear cystargamide no hydroxylated[a],[b]	990.401	94561	0.4
(7ClW) Chlorinated cystargamide[e]	988.3854	5313075	25.2
(7ClW) Chlorinated linear cystargamide[a]	1006.3960	2059653	9.8

[a] The ester bond appeared to have been hydrolysed [b] That lacked the 5-hydroxy on the tryptophan [c] Average area from the reaction performed in triplicate [d] Calculated as: (Absorbance peak area species/Absorbance peak area cystargamide wild type (WT))*10 [e] MS2 data was not recorded due to interfering masses

Ratios of the detected cystargamide species:

Cyclic cystargamide species : Linear cystargamide species = **2:1**

Chlorinated cystargamide species : Chlorinated linear cystargamide species = **3:1**

Cyclic cystargamide species : cyclic cystargamide species no hydroxylated= **8:1**

Chlorinated cyclic cystargamide species : Chlorinated cystargamide no hydroxylated= **8:1**

Analysis of the extract by LC-MS also revealed quantities of analogues of cystargamide containing alanine [ALA] instead of glycine. In this case, only linear forms in which the ester bond appeared to have been hydrolysed were detected.

Detected Cystargamide[ALA] species	[M+H]+	Absorbance peak area[c]	Ratio compared to Cystargamide (WT)[d]
Linear cystargamide[ALA][a]	986.4500	5187247	24.6
Linear cystargamide[ALA] no hydroxylated[a],[b]	970.4557	654399	3.1
(7ClW) Chlorinated linear cystargamide[ALA] no hydroxylated[a],[b]	1004.4167	956515	4.5
(7ClW) Chlorinated linear cystargamide[ALA][a]	1020.4116	7761149	36.8

[a] The ester bond appeared to have been hydrolysed [b] That lacked the 5-hydroxy on the tryptophan [c] Average area from the reaction performed in triplicate [d] Calculated as: (Absorbance peak area species/Absorbance peak area cystargamide wild type (WT))*10

Results demonstrated that all of the halotryptophans incorporated into cystargamide, generating new to nature halogenated analogues. As the concentration of tryptophan had no major impacted the amount of halo–cystargamide produced (see tables below) , we selected to scale up of the feeding of 6-bromotryptophan at 0.25 mM .

Scaled up feeding experiment with 6-bromotryptophan

A starter culture was prepared by inoculation of 50 mL GY medium in a 250 mL baffled conical flask with 2 mL glycerol mycelium stock, and fermented for 48 h at 28 °C and 200 rpm in an incubator with a 2.54 cm throw.
Fermentation cultures (8 L) were prepared by inoculating the production medium (0.4 g/L glucose, 0.8 g/L galactose, 0.8 g/L maltose, 1.6 g/L dextrin, 0.8 g/L soya peptone and 0.3 g/L ammonium sulfate, supplemented with 73 mg/L 6-bromotryptophan [0.26 mM] prepared as previously described[1] with starter culture (10% inoculum), and fermented for 72 h at 28 °C and 200 rpm.

The fermentation cultures were centrifuged (6000 rpm, 8980g, 30 min) and the supernatant was extracted with XAD7-HP resin overnight at 4 °C (20 mL resin / L supernatant). The resin was washed liberally with water, after which bound metabolites were eluted in 400 mL volumes each of 100% aqueous methanol. The presence of cystargamide and bromocystargamide was confirmed by UPLC and the extract was fractionated by gel permeation chromatography, using a 200 mL column of Sephadex LH-20 resin in methanol under gravity flow (25-30 cm/h). Cystargamide and bromocystargamide, both in their linear and cyclised forms, eluted between 120 – 180 mL. These fractions were further fractionated by RP-HPLC, using a Waters XBridge Prep Phenyl 10 x 250 mm 5 µm column with an isocratic gradient of 70% aqueous methanol and 0.1% formic acid and a flow rate of 6 mL/min, monitoring UV absorbance at 280 nm. Cystargamide was found to elute at 10.5 minutes, followed by bromocystargamide at 11 minutes. Solvent was removed from fractions using a Genevac centrifugal vacuum concentrator.

Throughout the purification, fractions were analysed by UPLC (95% acetonitrile and 0.1% trifluoroacetic acid with a flow rate of 0.6 ml/min, monitoring between 220 and 400 nm) or by LC-HRMS (XBridge C18 2.1 x 150 mm column, 5-95% 0.1% formic acid/water; acetonitrile, monitoring at 254 and 280 nm, HRMS: *m*/*z* calcd for $C_{49}H_{59}BrN_7O_{13}$ $[M+H]^+$: 1034.3349; found: 1032.3321.

The cystargamide and bromocystargamide were produced in linear and cyclic forms.

Extraction and purification resulted in a pure fraction containing 1.0 mg 6-bromocystargamide **9**. By comparison of the absorbance peak areas it could be estimated that the parent unhalogenated cystargamide **62**, a bromo-cystargamide analogue that lacked the 5-hydroxy on the tryptophan **63** and bromocystargamide **9**, were present in an approximate 10:0.2:1.4 ratio.

LC-MSMS data for cystargamide and its derivatives

Natural Cyclic Cystargamide (62)

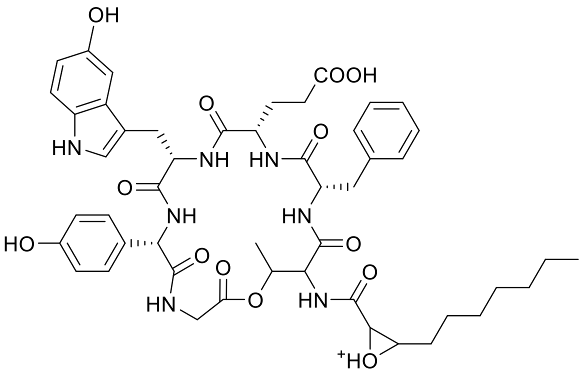

Chemical Formula: $C_{49}H_{60}N_7O_{13}^+$
Exact Mass: 954.4244

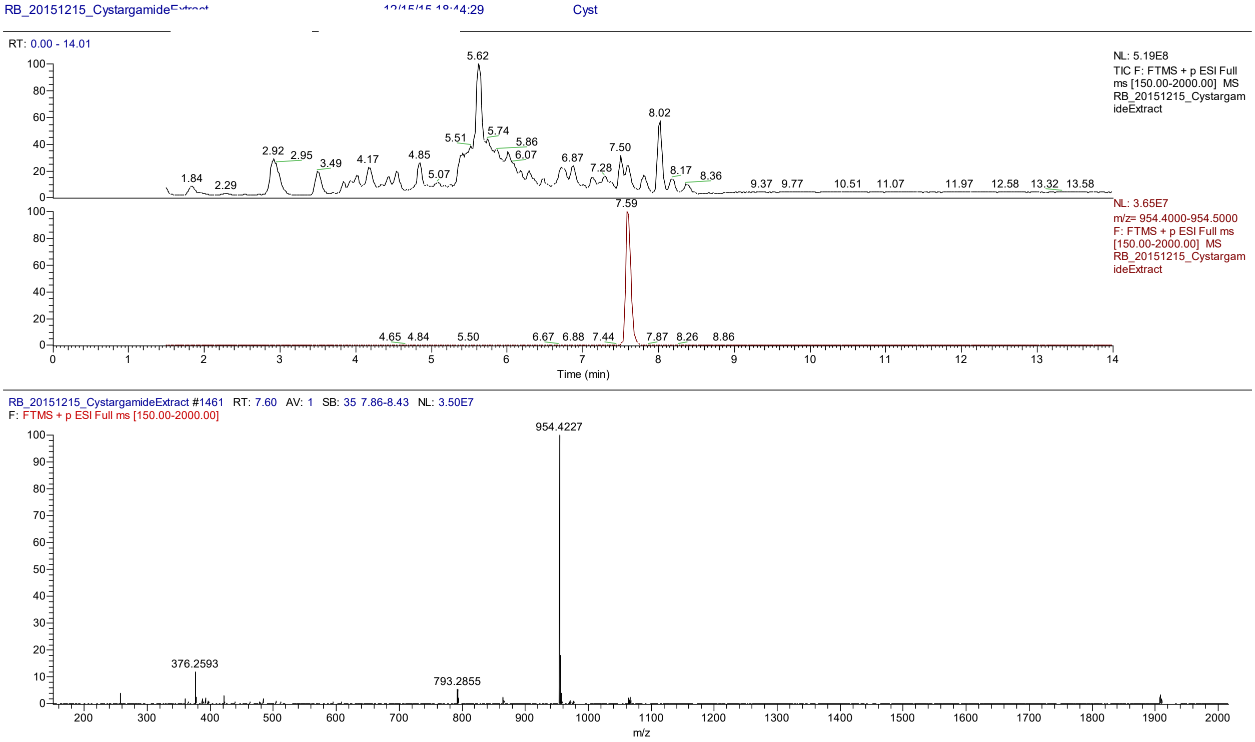

MS2 spectrum and fragment assignment

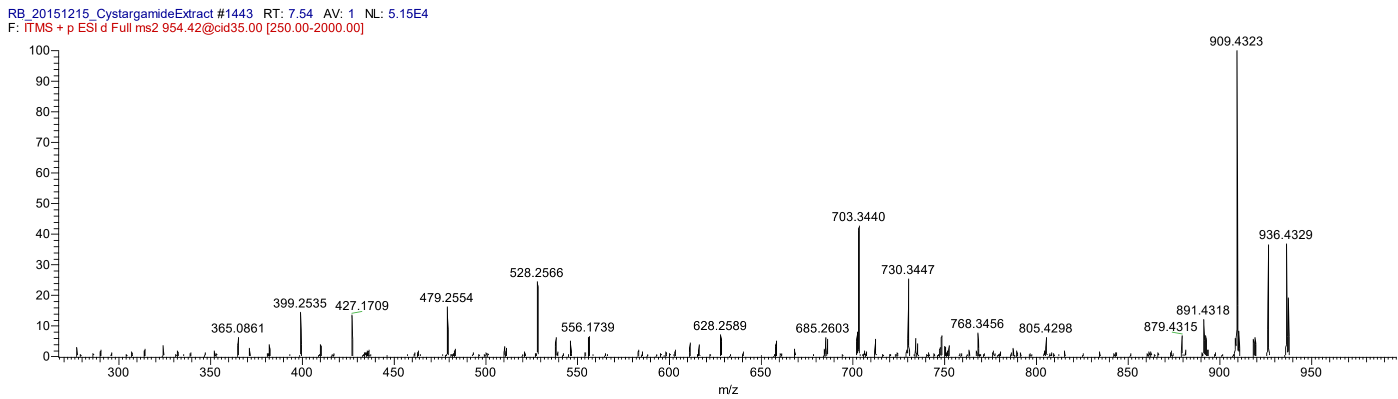

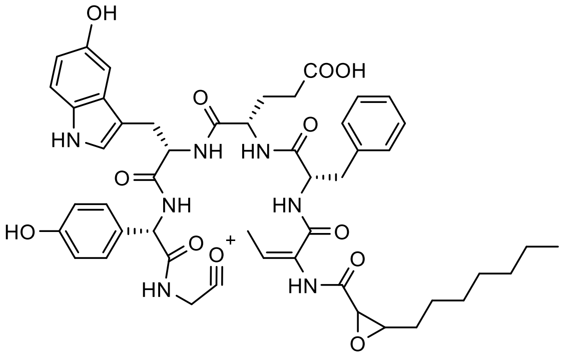

Chemical Formula: $C_{49}H_{58}N_7O_{12}^+$
Exact Mass: 936.4138

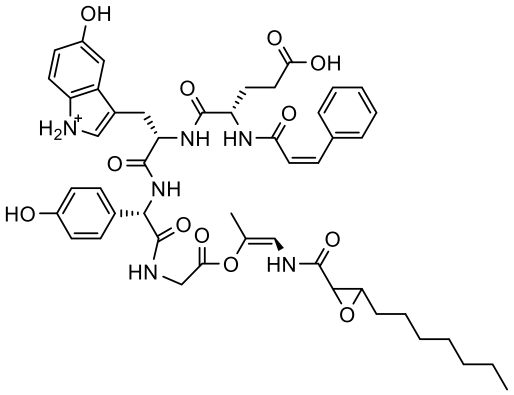

Chemical Formula: $C_{48}H_{57}N_6O_{12}^+$
Exact Mass: 909.4029

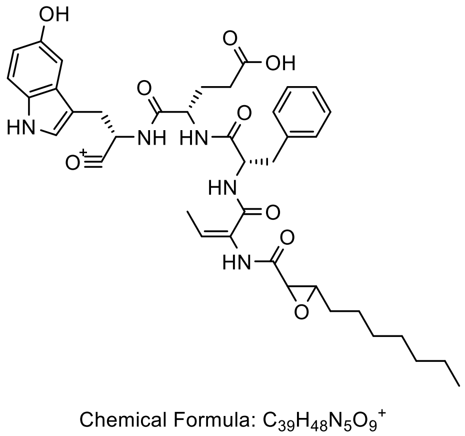

Chemical Formula: $C_{39}H_{48}N_5O_9^+$
Exact Mass: 730.3447

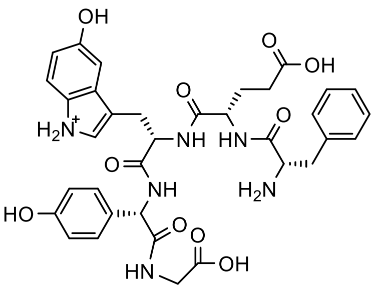

Chemical Formula: $C_{35}H_{39}N_6O_{10}^+$
Exact Mass: 703.2722

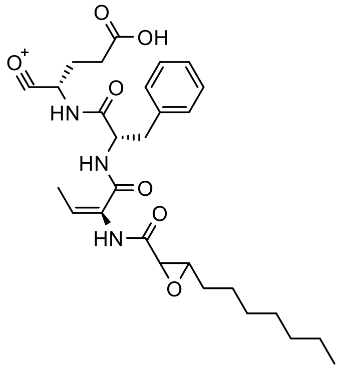

Chemical Formula: $C_{28}H_{38}N_3O_7^+$
Exact Mass: 528.2704

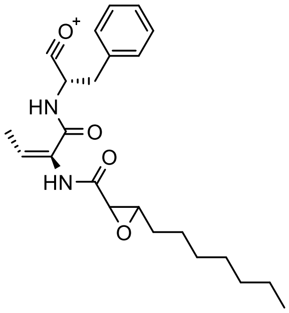

Chemical Formula: $C_{23}H_{31}N_2O_4^+$
Exact Mass: 399.2278

Linear cystargamide

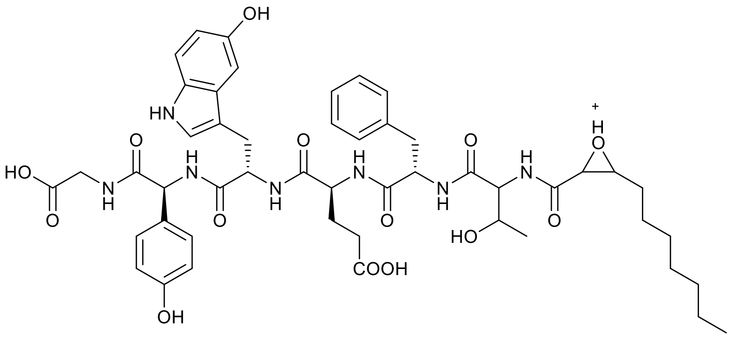

Chemical Formula: $C_{49}H_{62}N_7O_{14}^+$
Exact Mass: 972.4349

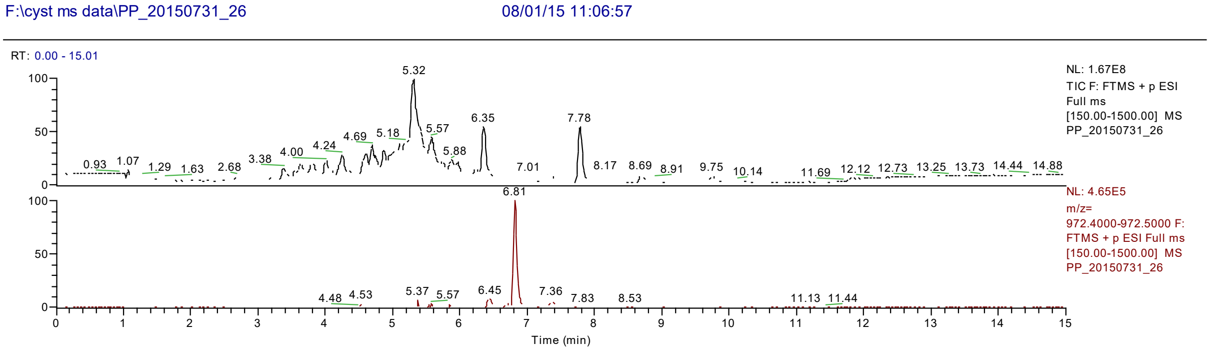

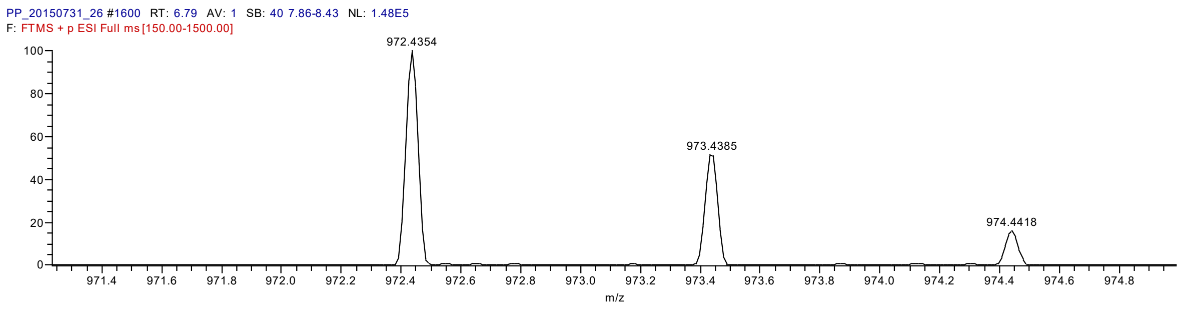

MS2 spectrum and fragment assignment

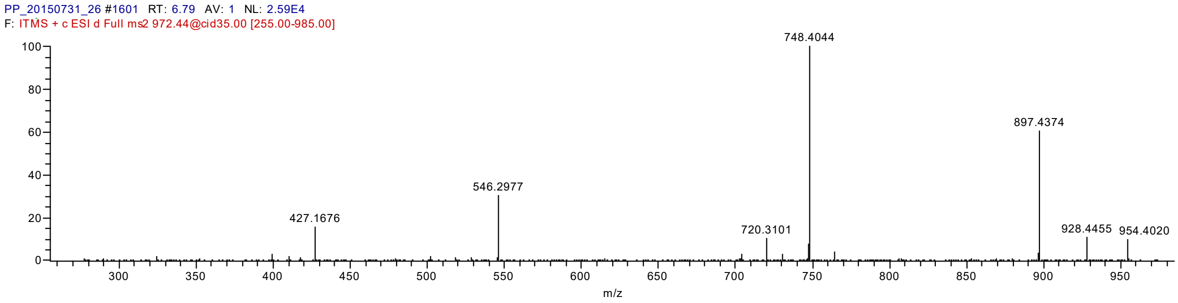

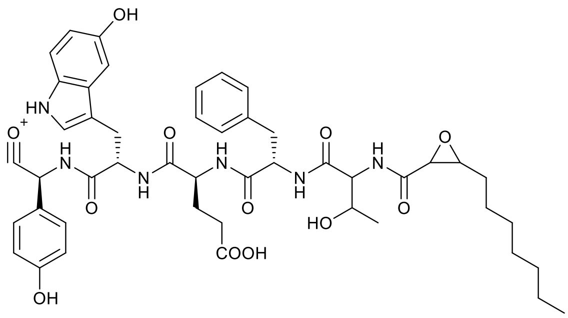

Chemical Formula: $C_{47}H_{57}N_6O_{12}^+$
Exact Mass: 897.4029

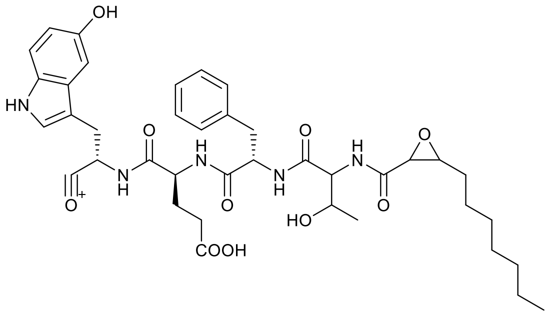

Chemical Formula: $C_{39}H_{50}N_5O_{10}^+$
Exact Mass: 748.3552

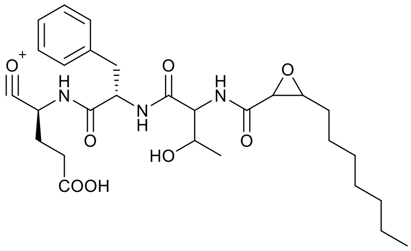

Chemical Formula: $C_{28}H_{40}N_3O_8^+$
Exact Mass: 546.2810

Natural cystargamide un-hydroxylated

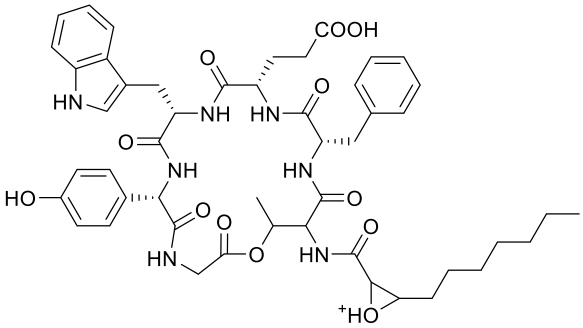

Chemical Formula: $C_{49}H_{60}N_7O_{12}^+$
Exact Mass: 938.4294

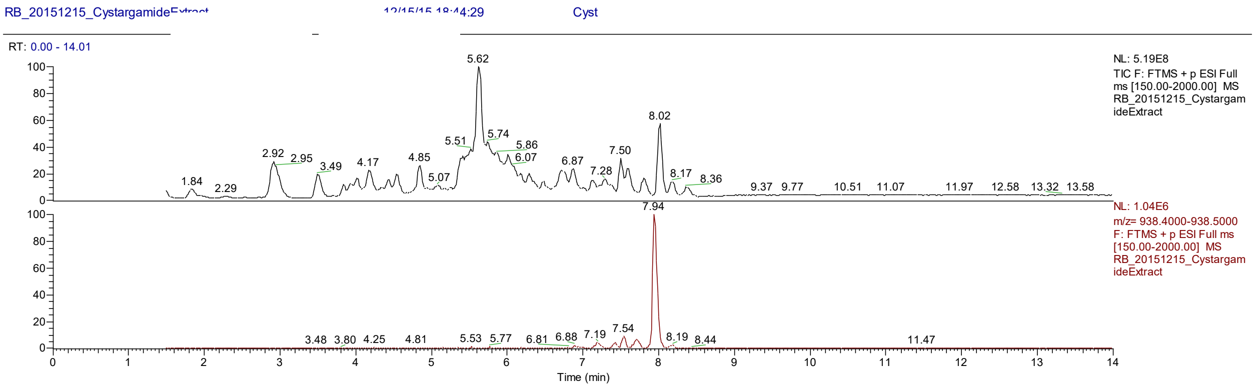

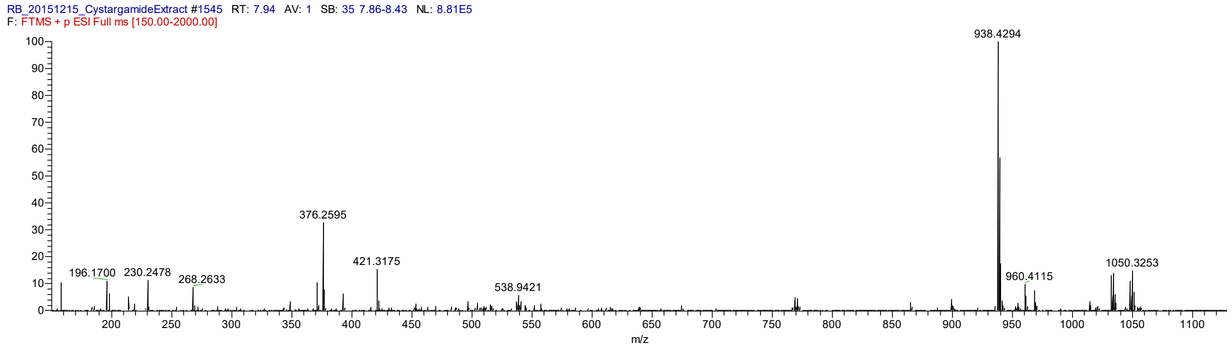

MS2 spectrum and fragment assignment

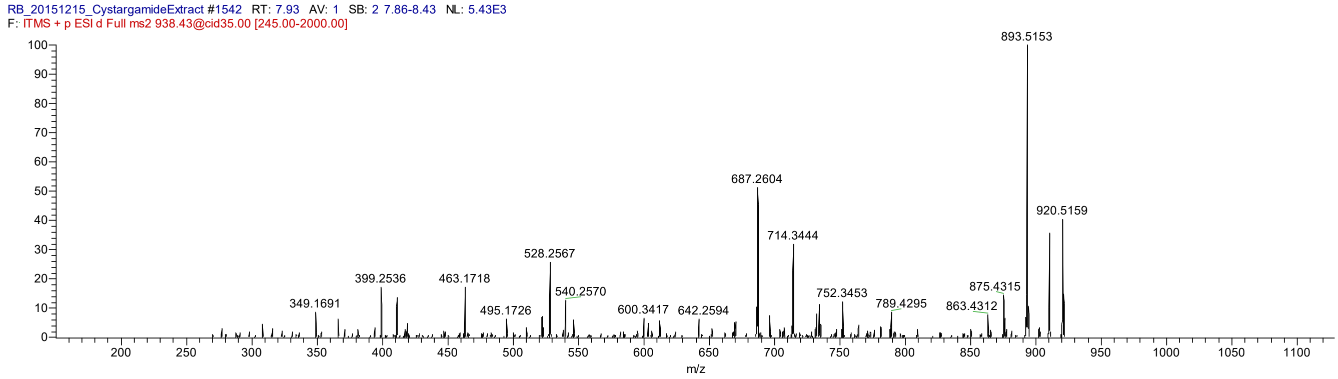

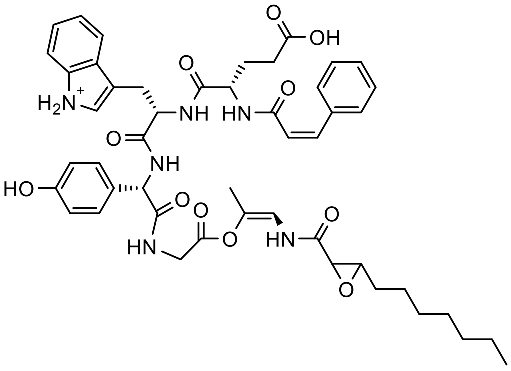

Chemical Formula: $C_{48}H_{57}N_6O_{11}^+$

Exact Mass: 893.4080

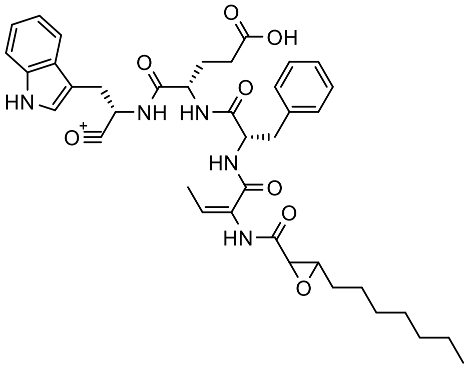

Chemical Formula: $C_{39}H_{48}N_5O_8^+$

Exact Mass: 714.3497

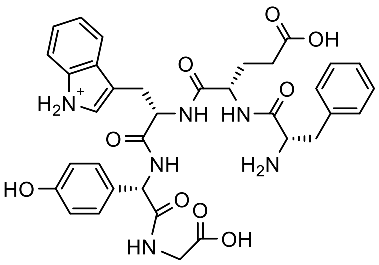

Chemical Formula: $C_{35}H_{39}N_6O_9^+$
Exact Mass: 687.2773

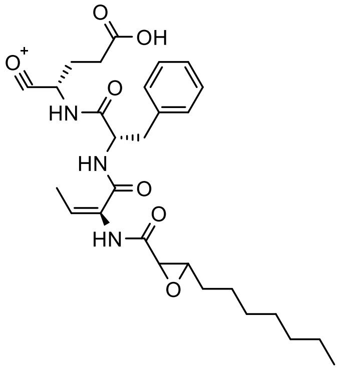

Chemical Formula: $C_{28}H_{38}N_3O_7^+$
Exact Mass: 528.2704

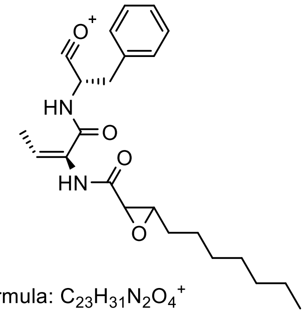

Chemical Formula: $C_{23}H_{31}N_2O_4^+$
Exact Mass: 399.2278

Bromo-cystargamide un-hydroxylated (63)

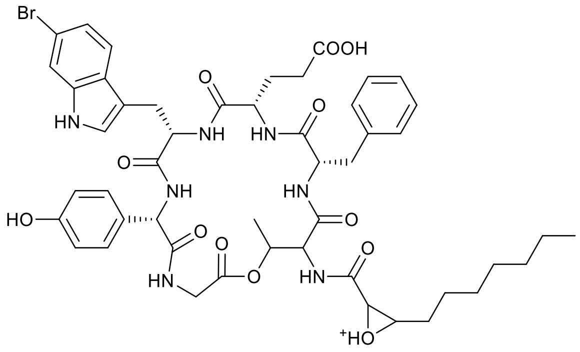

Chemical Formula: $C_{49}H_{59}BrN_7O_{12}^+$
Exact Mass: 1016.3400

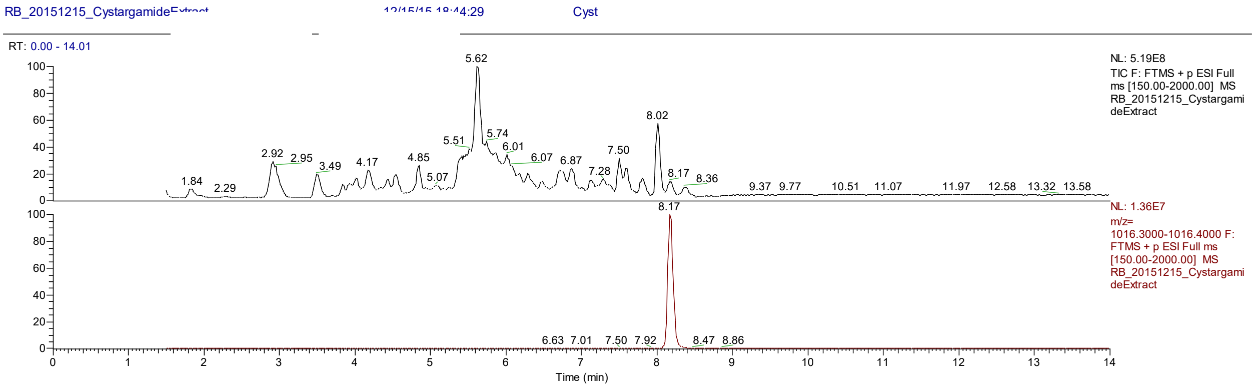

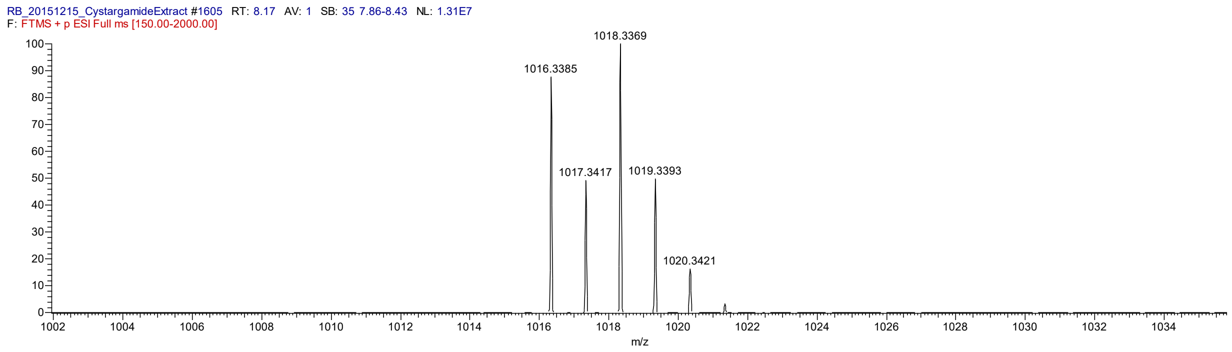

MS2 spectrum and fragment assignment

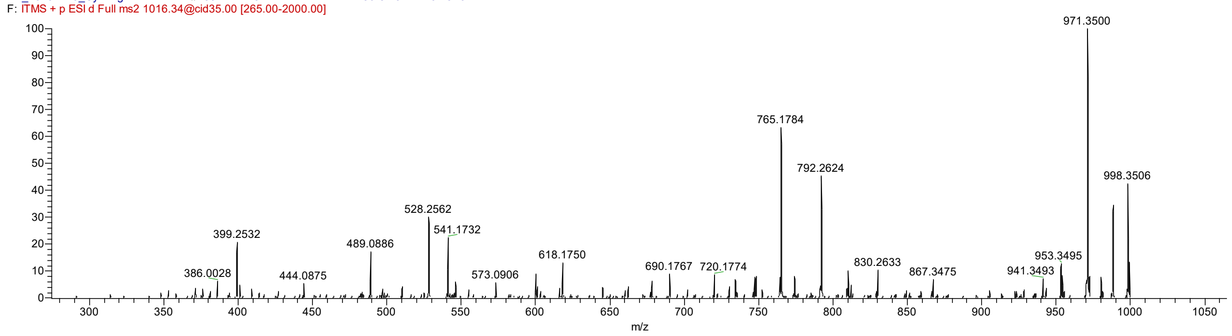

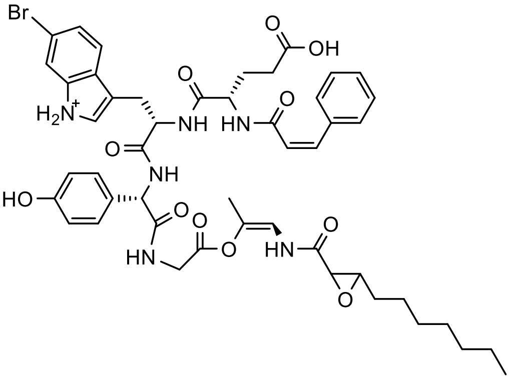

Chemical Formula: $C_{48}H_{56}BrN_6O_{11}^+$
Exact Mass: 971.3185

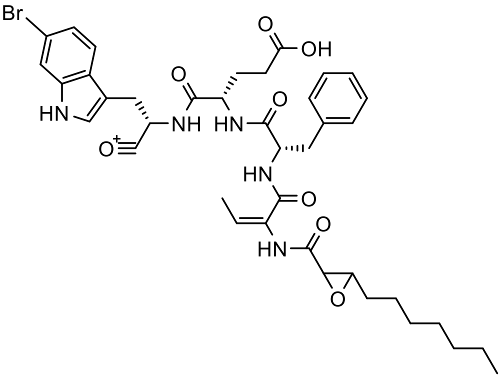

Chemical Formula: $C_{39}H_{47}BrN_5O_8^+$
Exact Mass: 792.2603

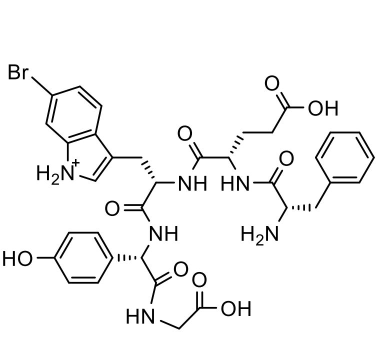

Chemical Formula: $C_{35}H_{38}BrN_6O_9^+$
Exact Mass: 765.1878

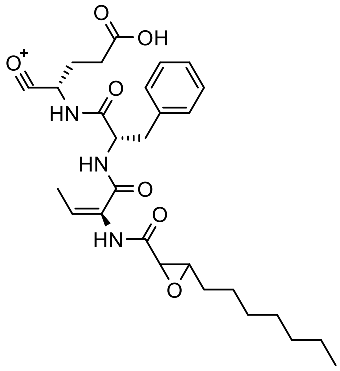

Chemical Formula: $C_{28}H_{38}N_3O_7^+$
Exact Mass: 528.2704

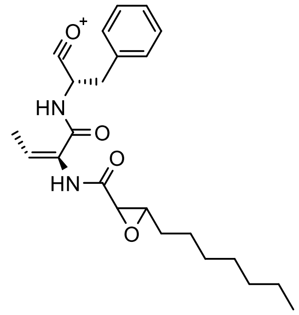

Chemical Formula: $C_{23}H_{31}N_2O_4^+$
Exact Mass: 399.2278

Brominated linear cystargamide un-hydroxylated

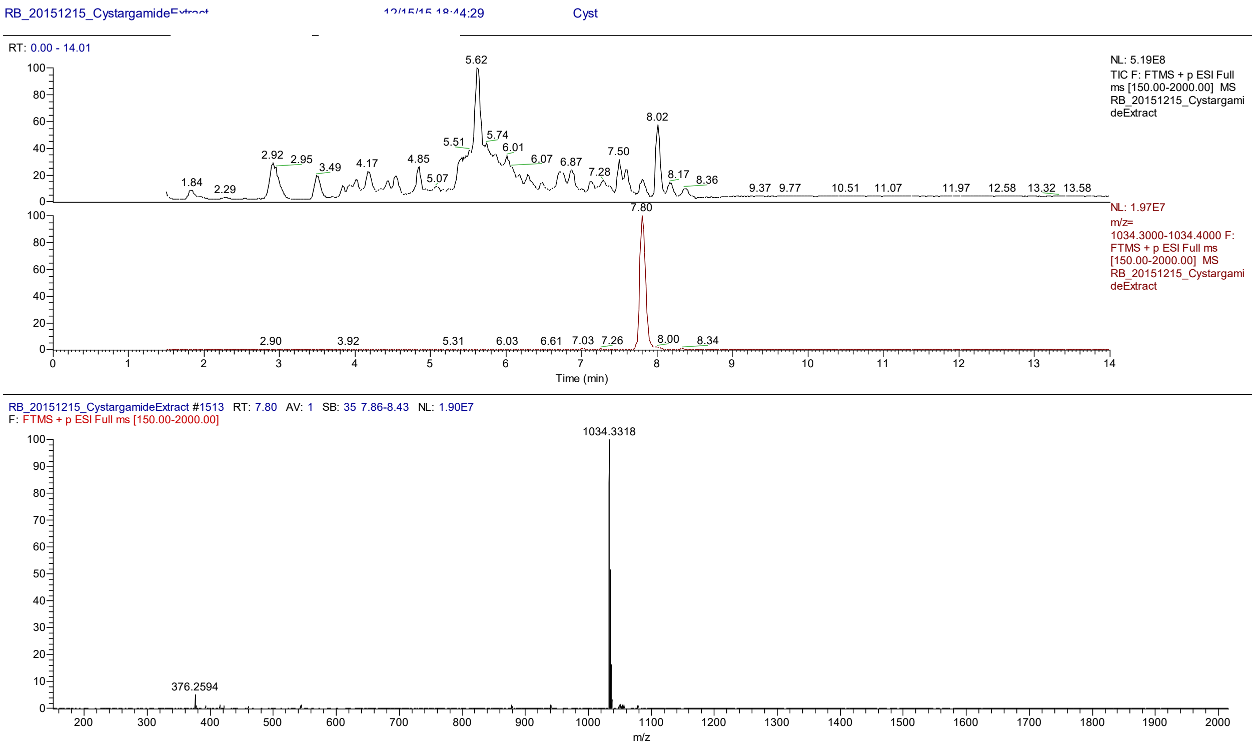

Brominated cystargamide (9)

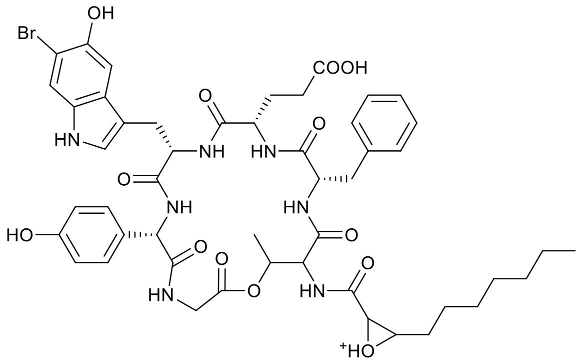

Chemical Formula: $C_{49}H_{59}BrN_7O_{13}^+$
Exact Mass: 1032.3349

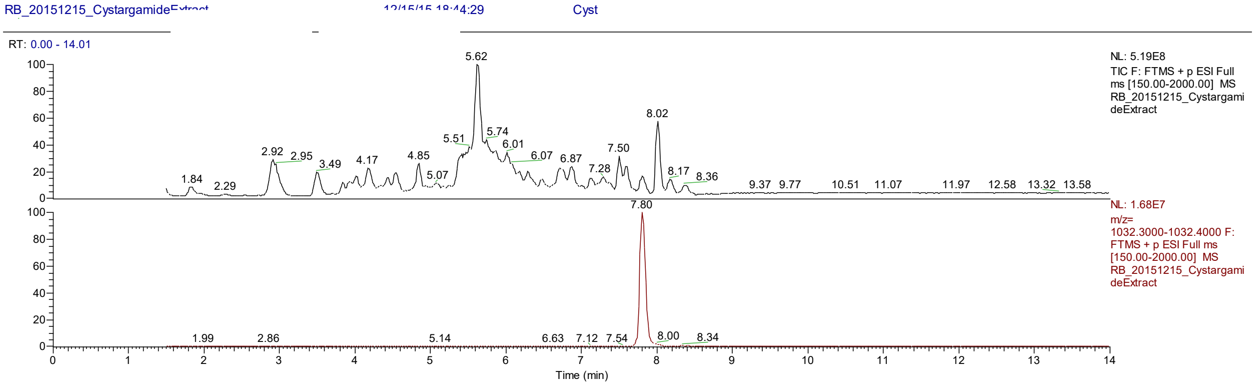

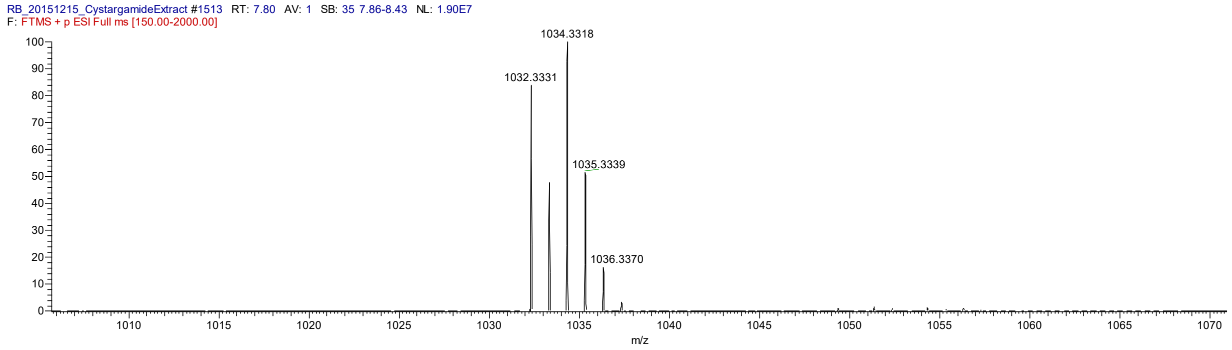

MS2 spectrum and fragment assignment

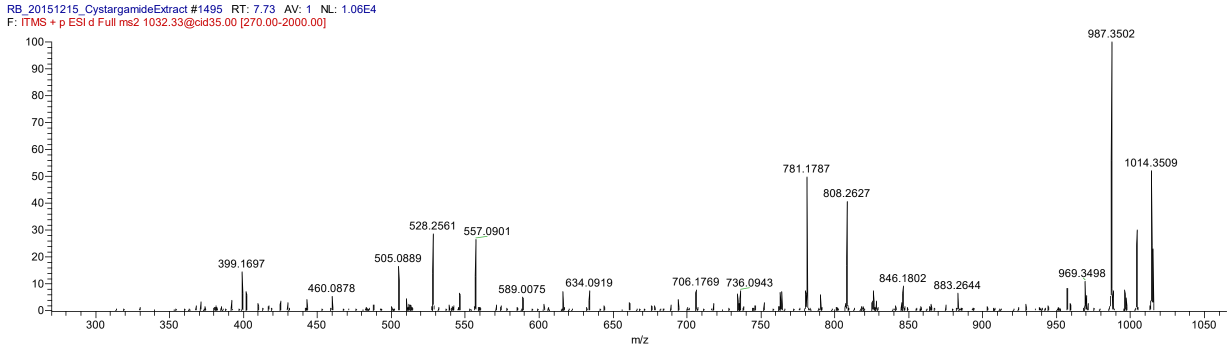

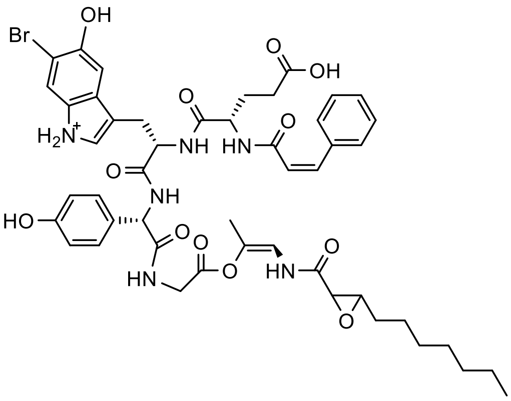

Chemical Formula: $C_{48}H_{56}BrN_6O_{12}^+$
Exact Mass: 987.3134

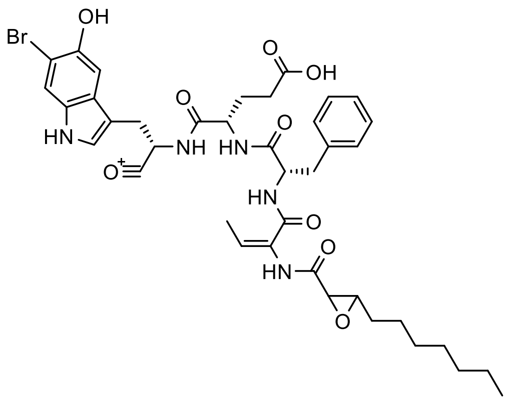

Chemical Formula: $C_{39}H_{47}BrN_5O_9^+$
Exact Mass: 808.2552

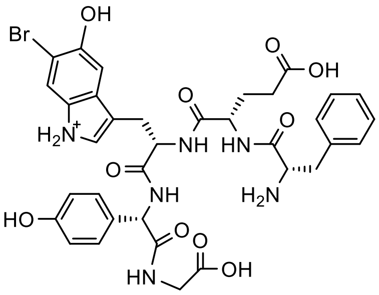

Chemical Formula: $C_{35}H_{38}BrN_6O_{10}^+$
Exact Mass: 781.1827

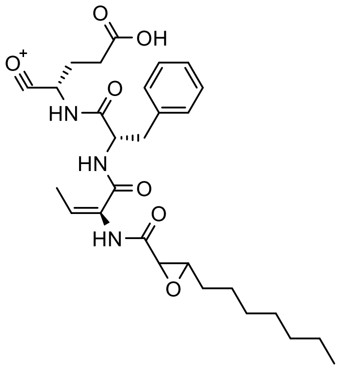

Chemical Formula: $C_{28}H_{38}N_3O_7^+$
Exact Mass: 528.2704

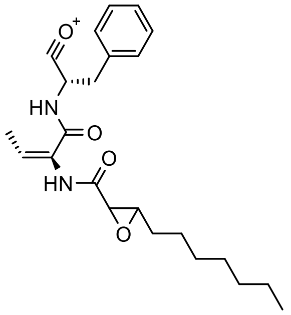

Chemical Formula: $C_{23}H_{31}N_2O_4^+$
Exact Mass: 399.2278

Linear cystargamide[ALA]

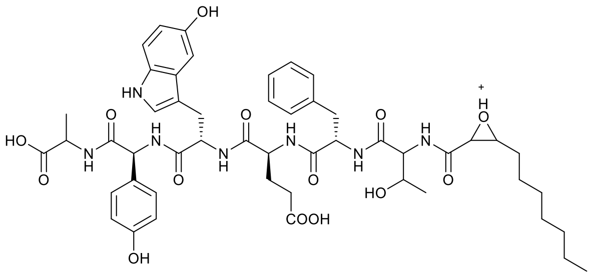

Chemical Formula: $C_{50}H_{64}N_7O_{14}^+$
Exact Mass: 986.4506

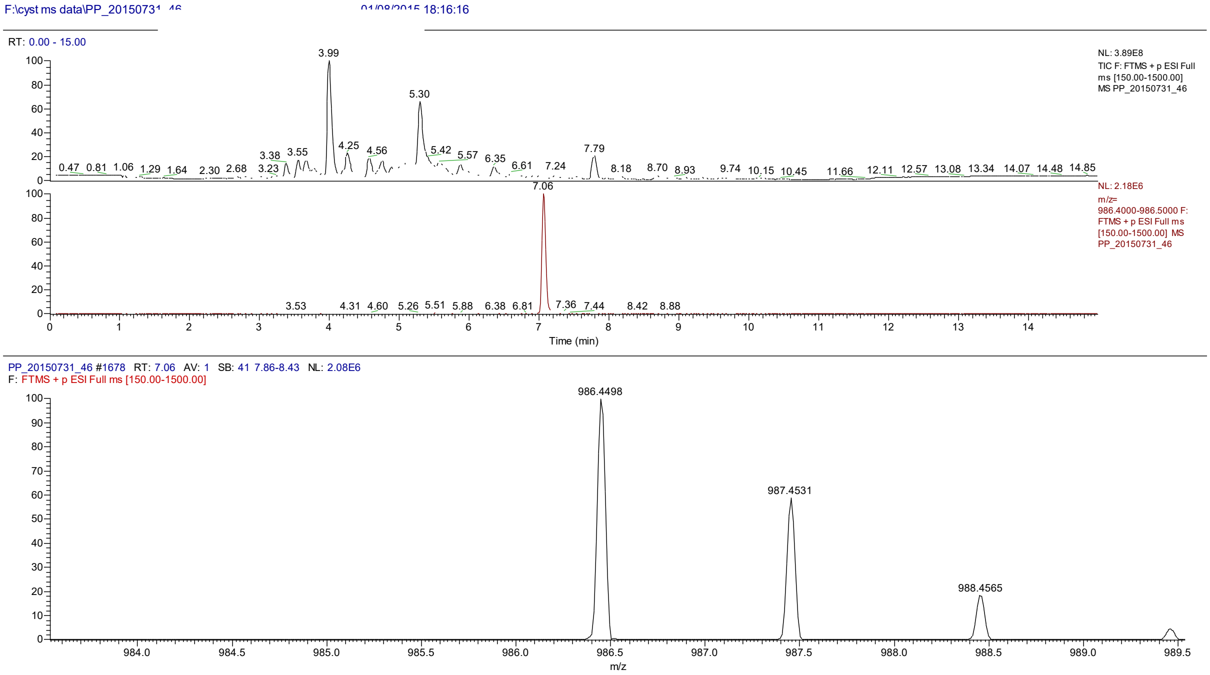

MS2 spectrum and fragment assignment

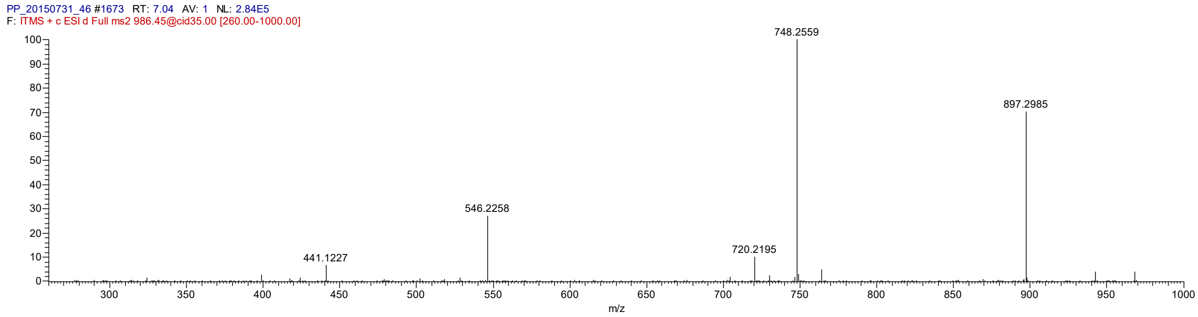

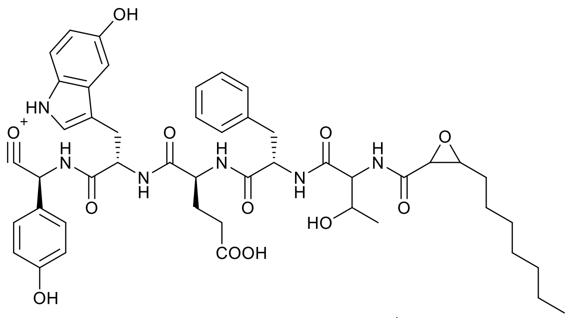

Chemical Formula: $C_{47}H_{57}N_6O_{12}^+$
Exact Mass: 897.4029

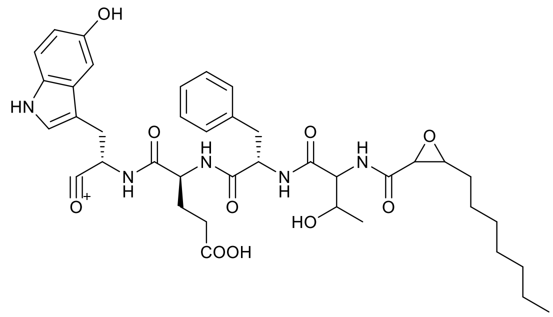

Chemical Formula: $C_{39}H_{50}N_5O_{10}^+$
Exact Mass: 748.3552

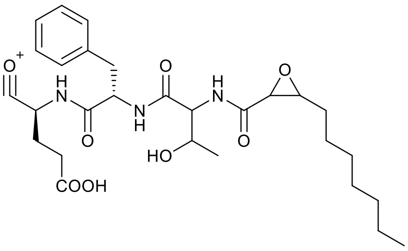

Chemical Formula: $C_{28}H_{40}N_3O_8^+$
Exact Mass: 546.2810

Linear cystargamide[ALA] un hydroxylated

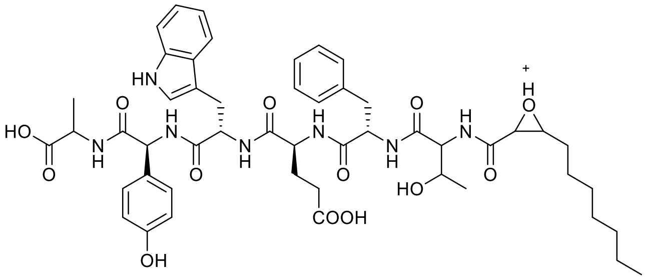

Chemical Formula: $C_{50}H_{64}N_7O_{13}^+$
Exact Mass: 970.4557

F:\cyst ms data\PP_20150731_41 08/01/15 16:33:14

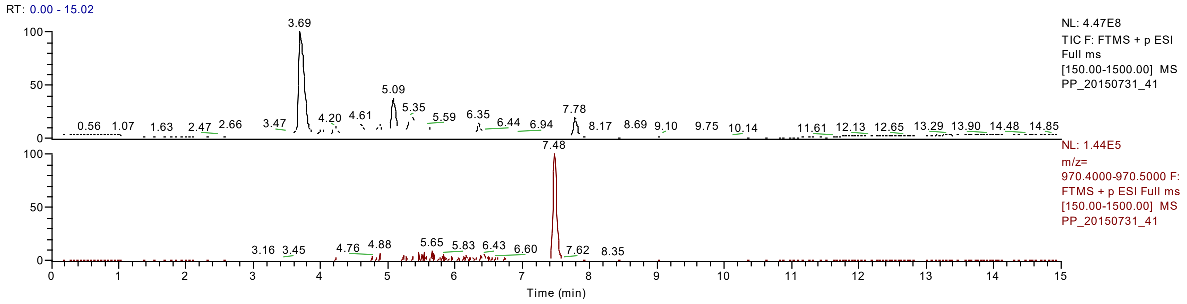

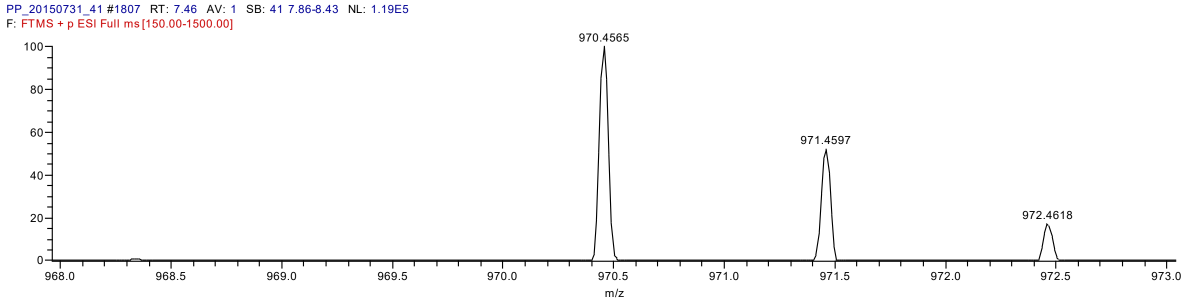

MS2 spectrum and fragment assignment

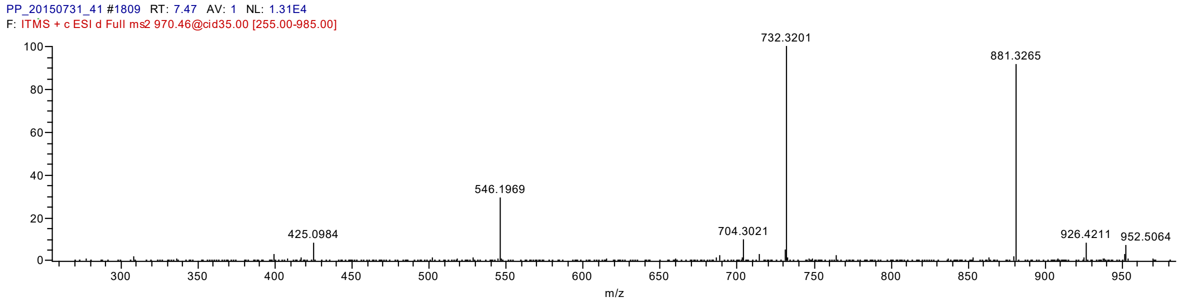

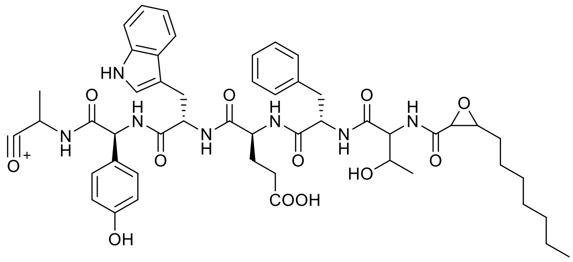

Chemical Formula: $C_{50}H_{62}N_7O_{12}^+$
Exact Mass: 952.4451

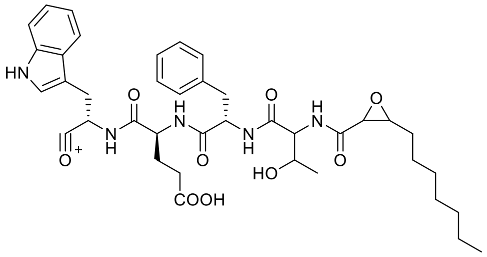

Chemical Formula: $C_{39}H_{50}N_5O_9^+$
Exact Mass: 732.3603

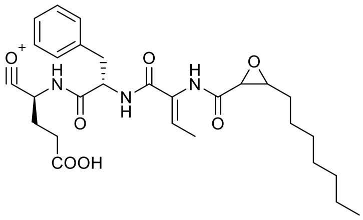

Chemical Formula: $C_{28}H_{38}N_3O_7^+$
Exact Mass: 528.2704

Brominated linear cystargamide[ALA] un hydroxylated

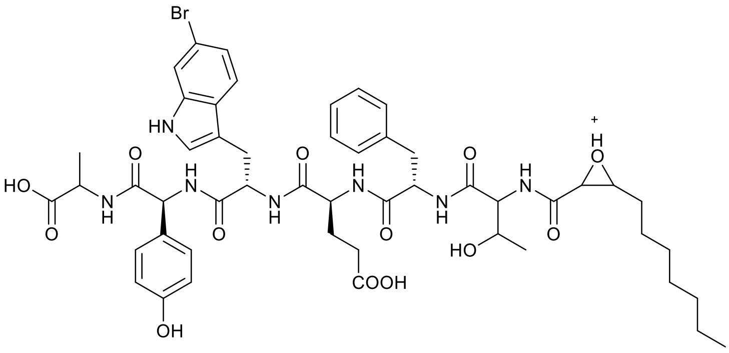

Chemical Formula: $C_{50}H_{63}BrN_7O_{13}^+$
Exact Mass: 1048.3662

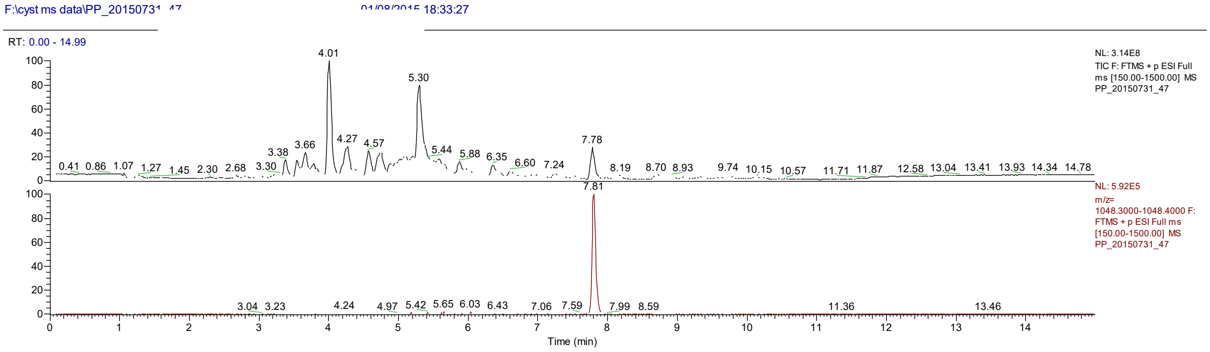

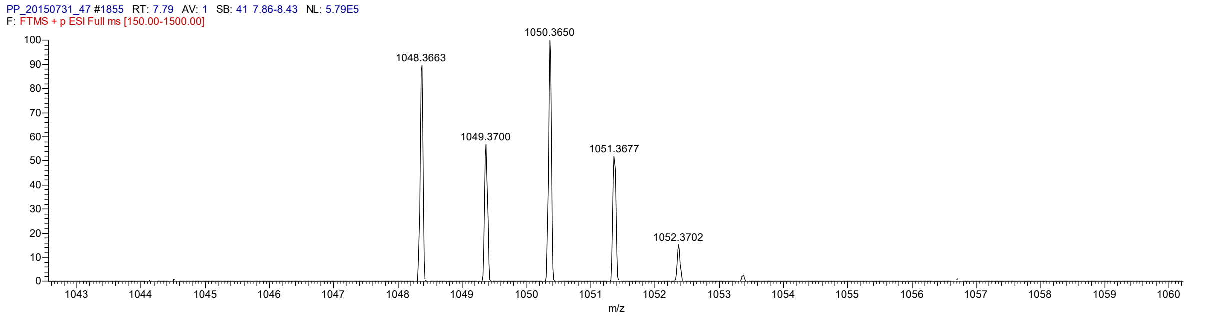

MS2 spectrum and fragment assignment

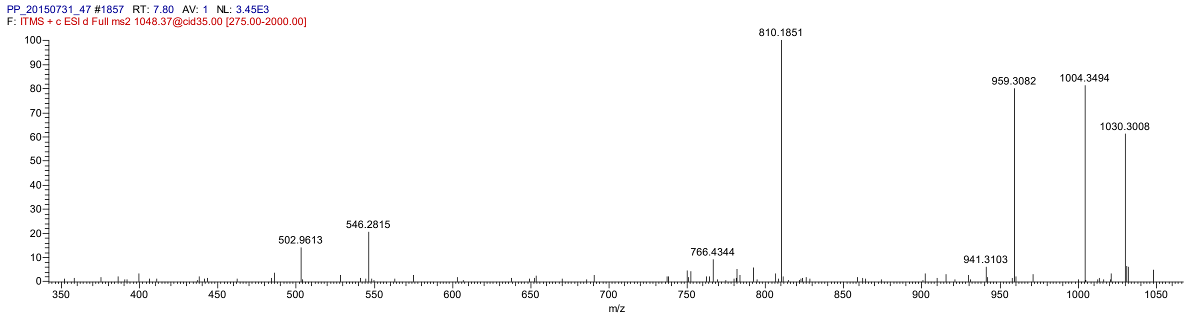

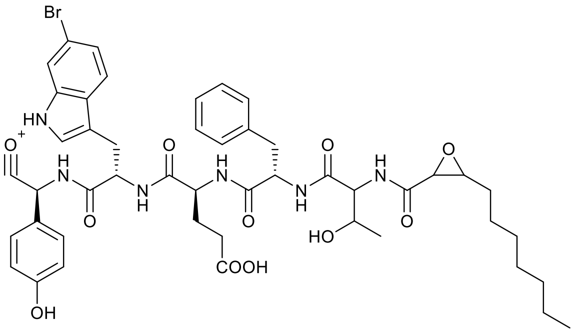

Chemical Formula: $C_{47}H_{56}BrN_6O_{11}^+$
Exact Mass: 959.3185

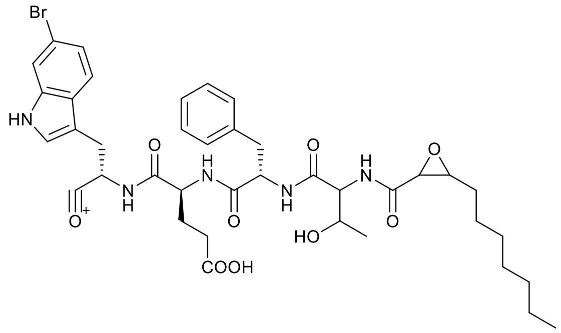

Chemical Formula: $C_{39}H_{49}BrN_5O_9^+$
Exact Mass: 810.2708

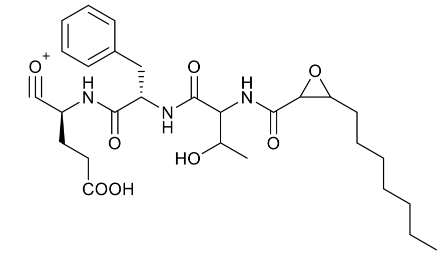

Chemical Formula: $C_{28}H_{40}N_3O_8^+$
Exact Mass: 546.2810

Brominated linear cystargamide[ALA]

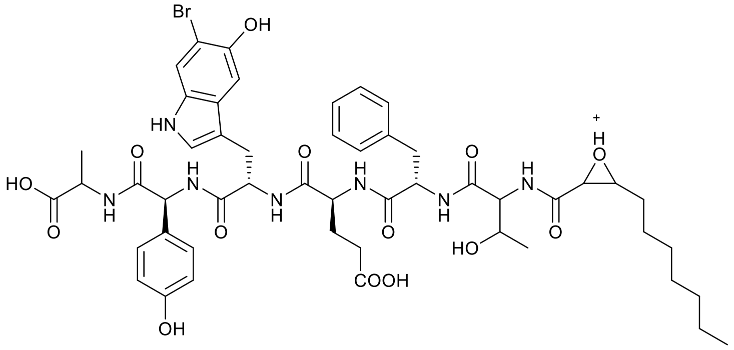

Chemical Formula: $C_{50}H_{63}BrN_7O_{14}^+$
Exact Mass: 1064.3611

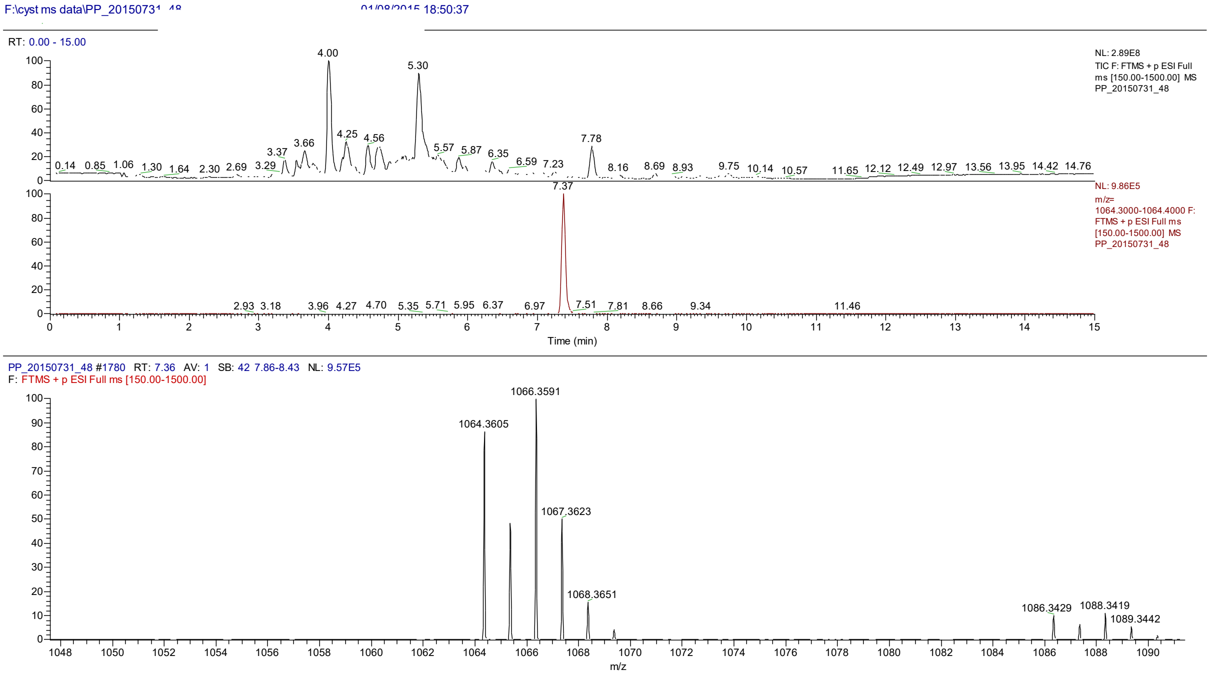

MS2 spectrum and fragment assignment

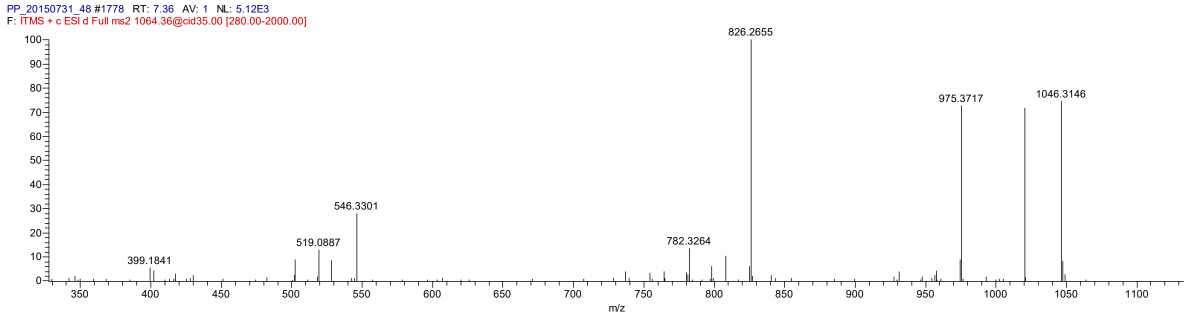

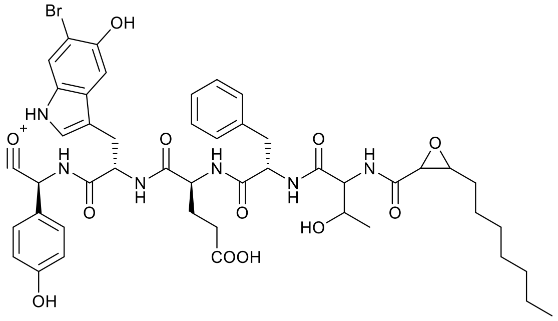

Chemical Formula: $C_{47}H_{56}BrN_6O_{12}^+$
Exact Mass: 975.3134

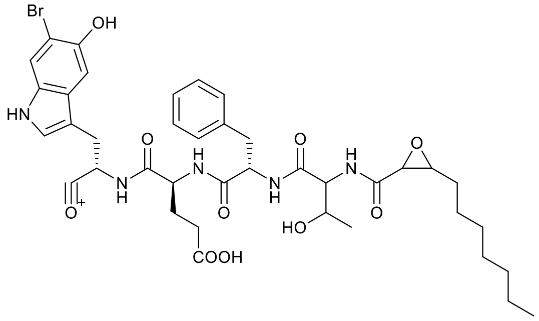

Chemical Formula: $C_{39}H_{49}BrN_5O_{10}^+$
Exact Mass: 826.2657

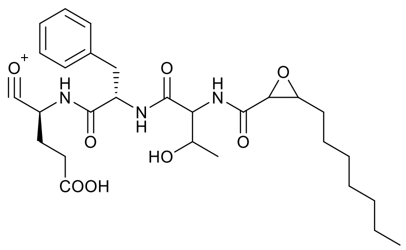

Chemical Formula: $C_{28}H_{40}N_3O_8^+$
Exact Mass: 546.2810

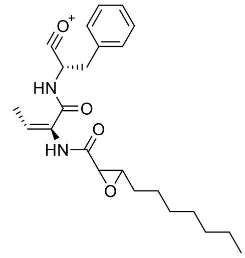

Chemical Formula: $C_{23}H_{31}N_2O_4^+$
Exact Mass: 399.2278

(5BrW) Brominated cystargamide unhydroxylated

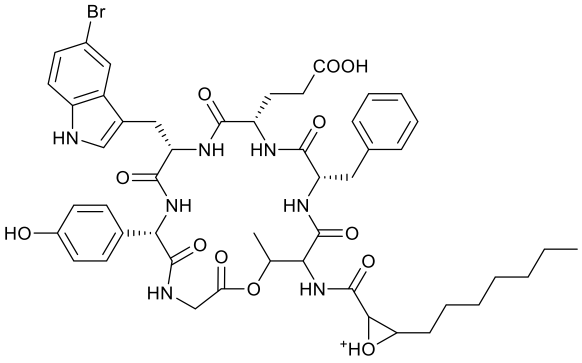

Chemical Formula: $C_{49}H_{59}BrN_7O_{12}^+$
Exact Mass: 1016.3400

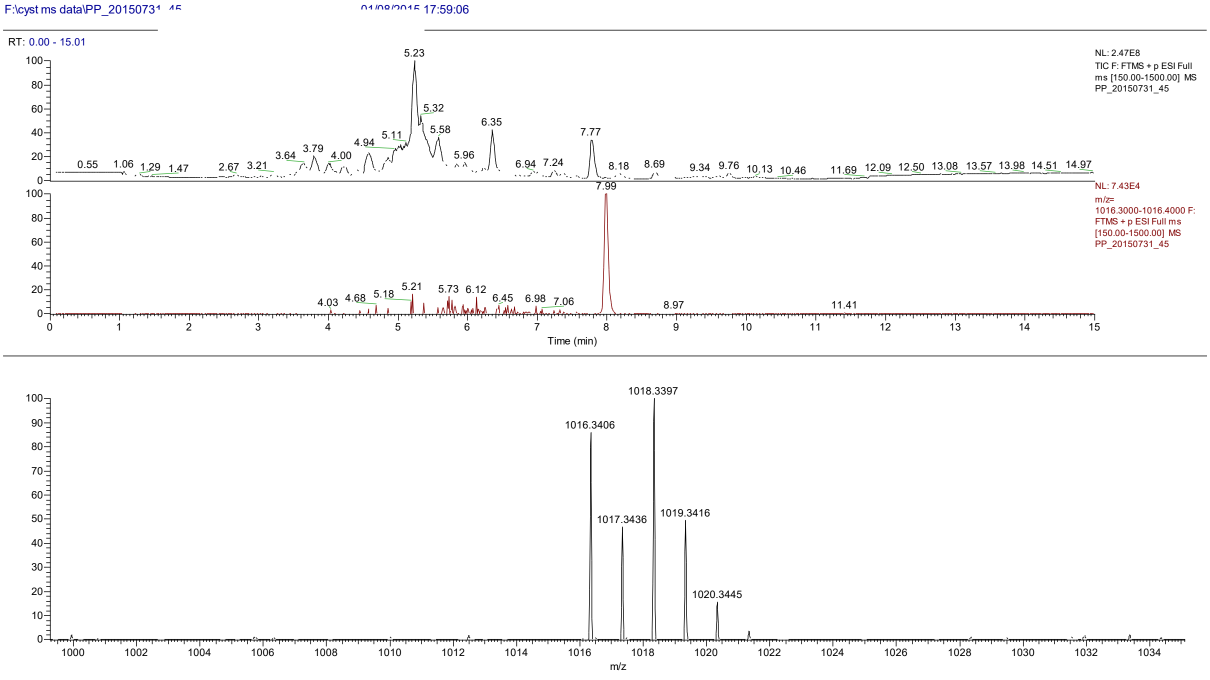

MS2 spectrum and fragment assignment

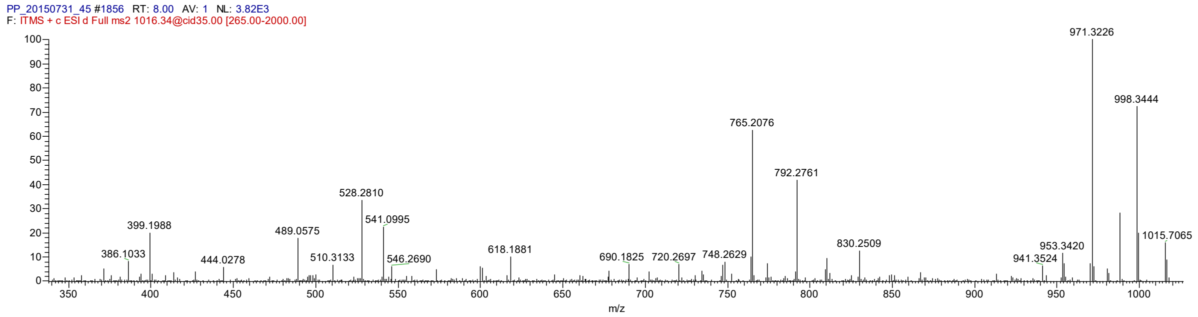

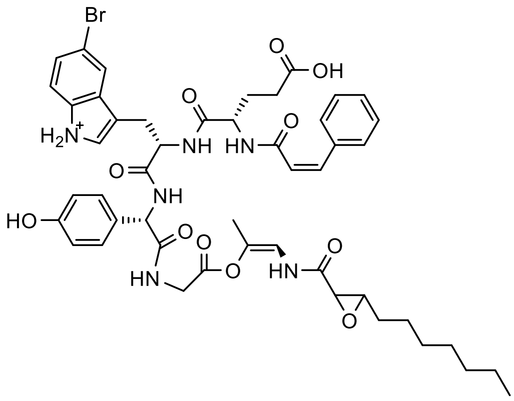

Chemical Formula: $C_{48}H_{56}BrN_6O_{11}^+$
Exact Mass: 971.3185

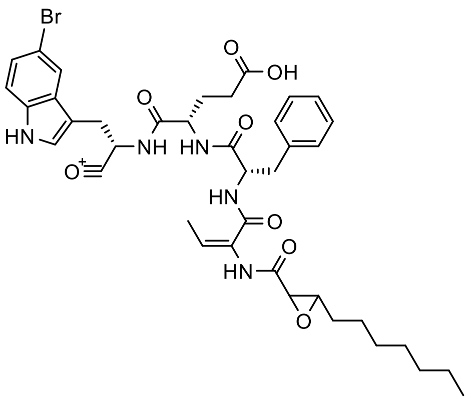

Chemical Formula: $C_{39}H_{47}BrN_5O_8^+$
Exact Mass: 792.2603

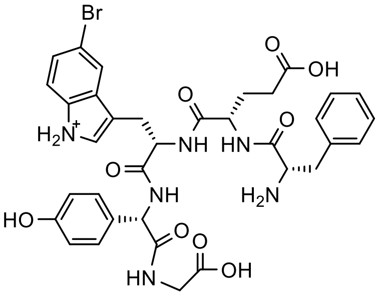

Chemical Formula: $C_{35}H_{38}BrN_6O_9^+$
Exact Mass: 765.1878

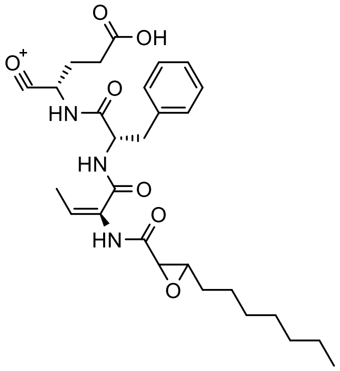

Chemical Formula: $C_{28}H_{38}N_3O_7^+$
Exact Mass: 528.2704

Chemical Formula: $C_{23}H_{31}N_2O_4^+$
Exact Mass: 399.2278

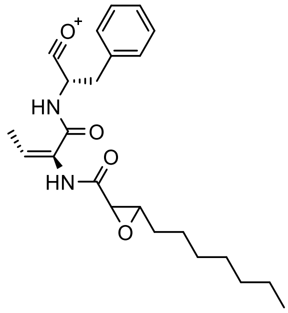

(5BrW) Brominated linear cystargamide[ALA] un hydroxylated

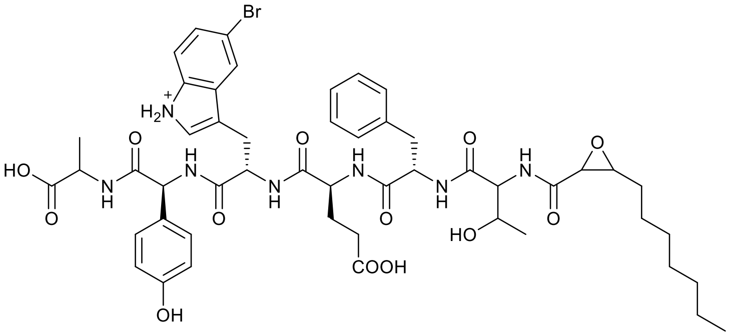

Chemical Formula: $C_{50}H_{63}BrN_7O_{13}^+$
Exact Mass: 1048.3662

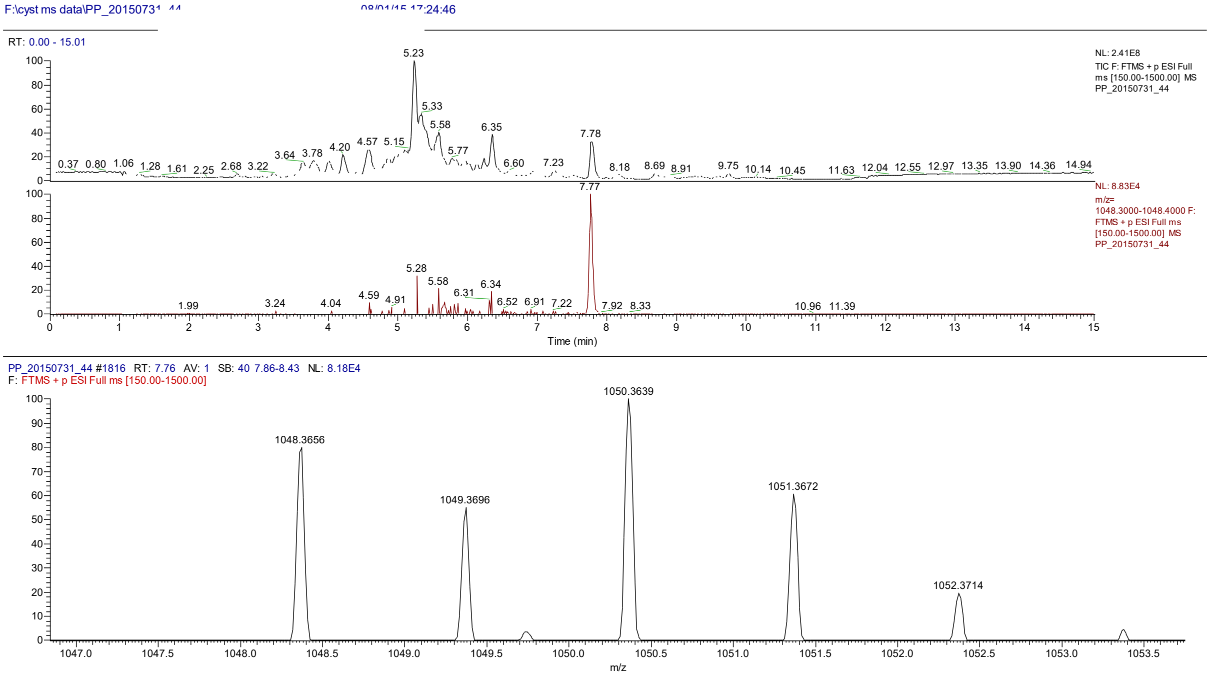

(7-BrW) Brominated cystargamide

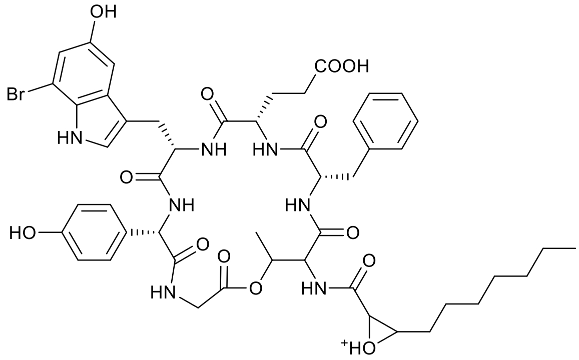

Chemical Formula: $C_{49}H_{59}BrN_7O_{13}^{+}$
Exact Mass: 1032.3349

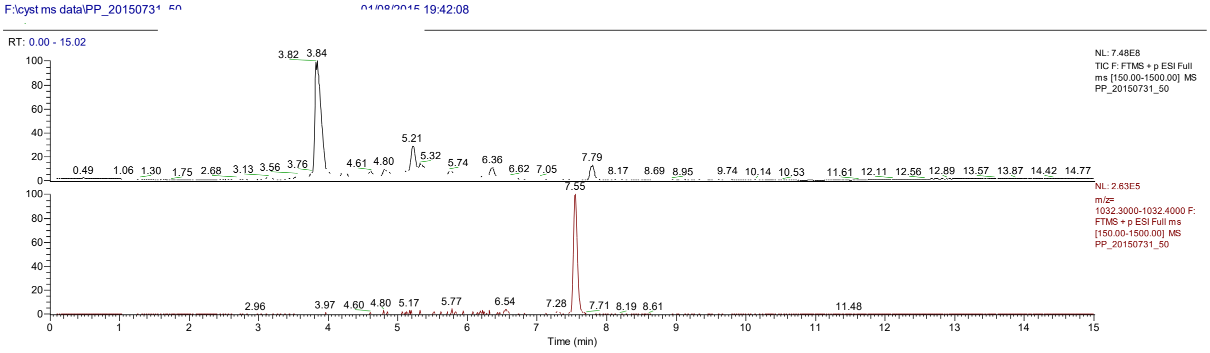

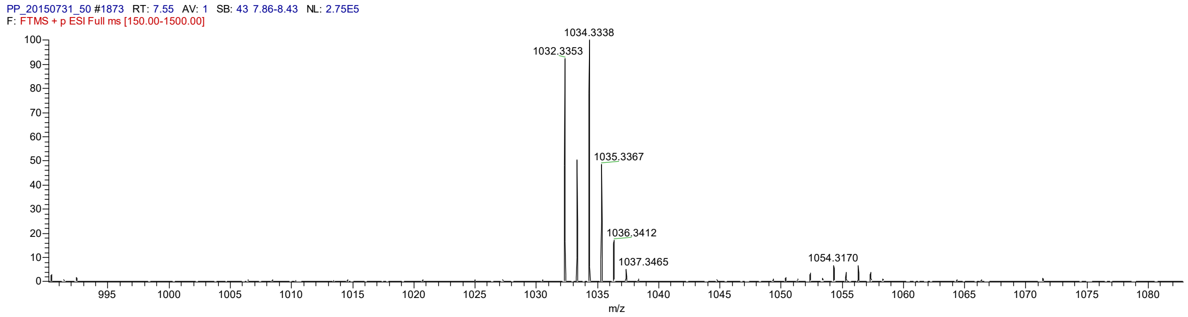

MS2 spectrum and fragment assignment

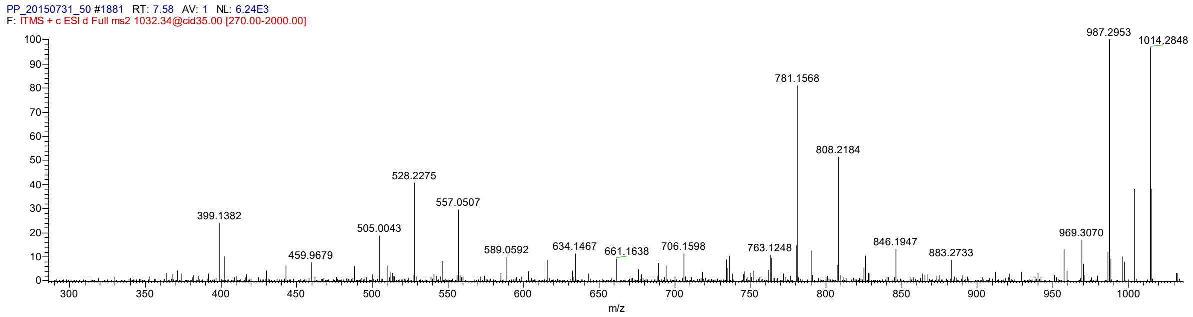

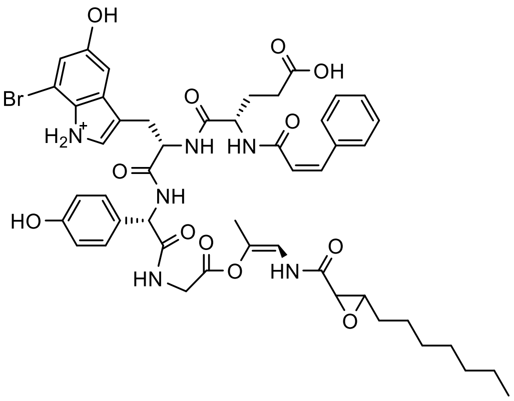

Chemical Formula: $C_{48}H_{56}BrN_6O_{12}^+$
Exact Mass: 987.3134

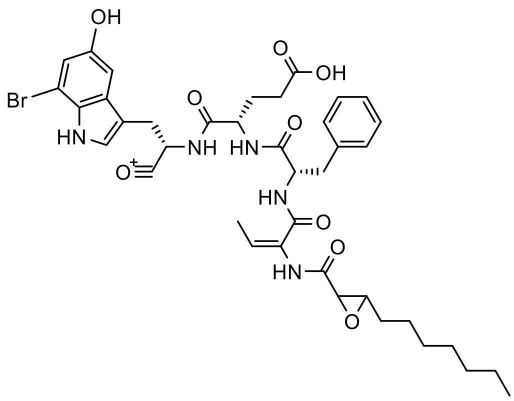

Chemical Formula: $C_{39}H_{47}BrN_5O_9^+$
Exact Mass: 808.2552

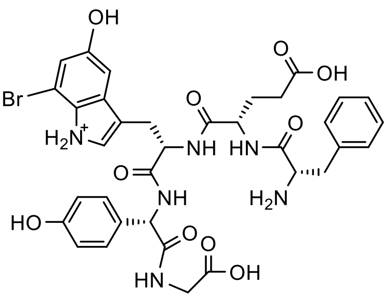

Chemical Formula: $C_{35}H_{38}BrN_6O_{10}^+$
Exact Mass: 781.1827

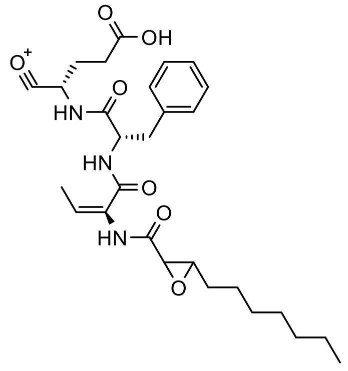

Chemical Formula: $C_{28}H_{38}N_3O_7^+$
Exact Mass: 528.2704

Chemical Formula: $C_{23}H_{31}N_2O_4^+$
Exact Mass: 399.2278

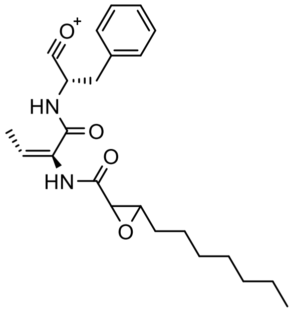

(7BrW) Brominated linear cystargamide[ALA] un hydroxylated

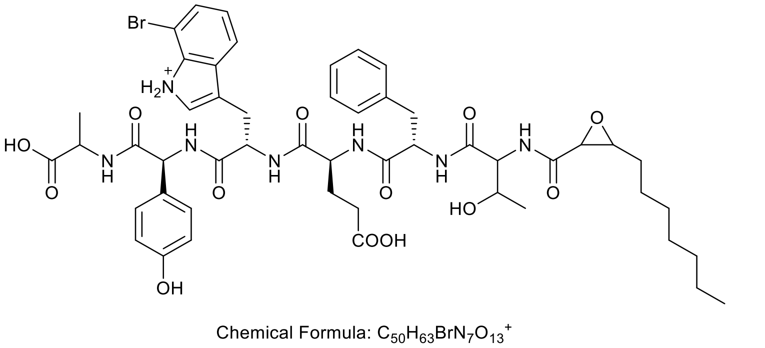

Chemical Formula: $C_{50}H_{63}BrN_7O_{13}^+$
Exact Mass: 1048.3662

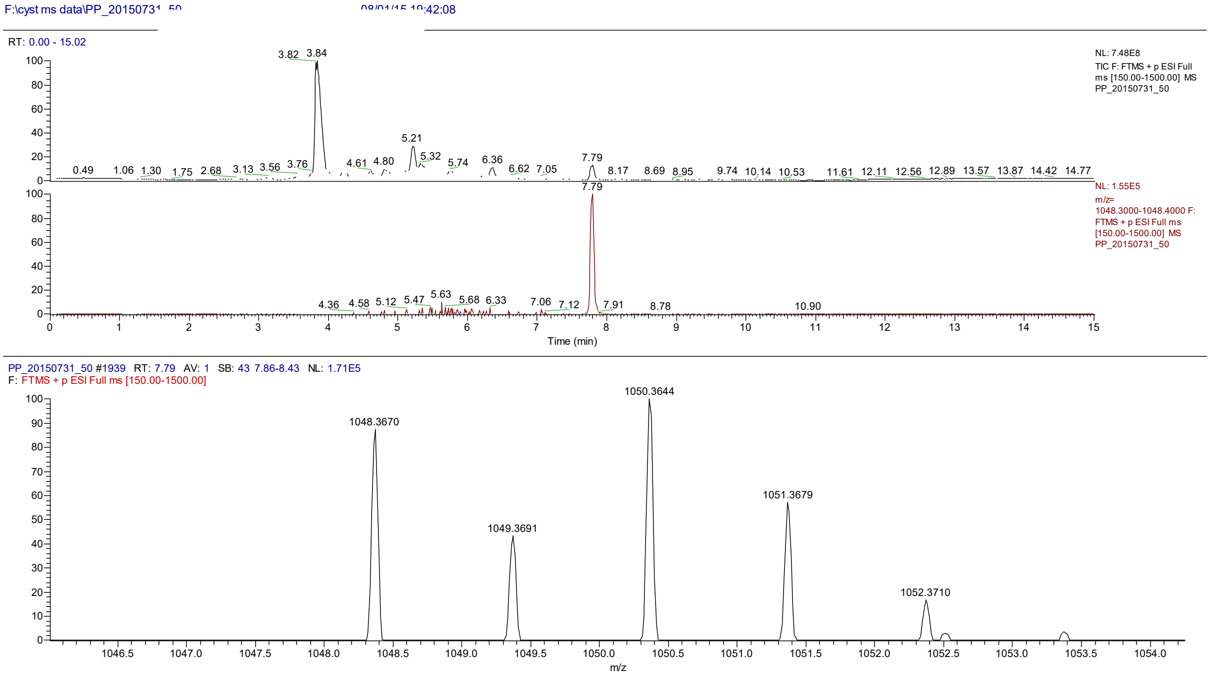

(6BrW) Brominated linear cystargamide[ALA]

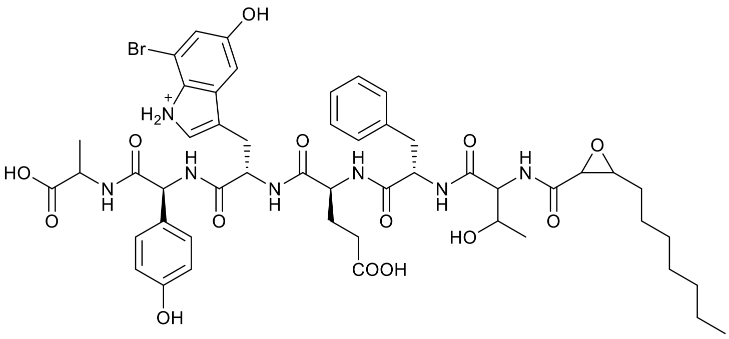

Chemical Formula: $C_{50}H_{63}BrN_7O_{14}^+$

Exact Mass: 1064.3611

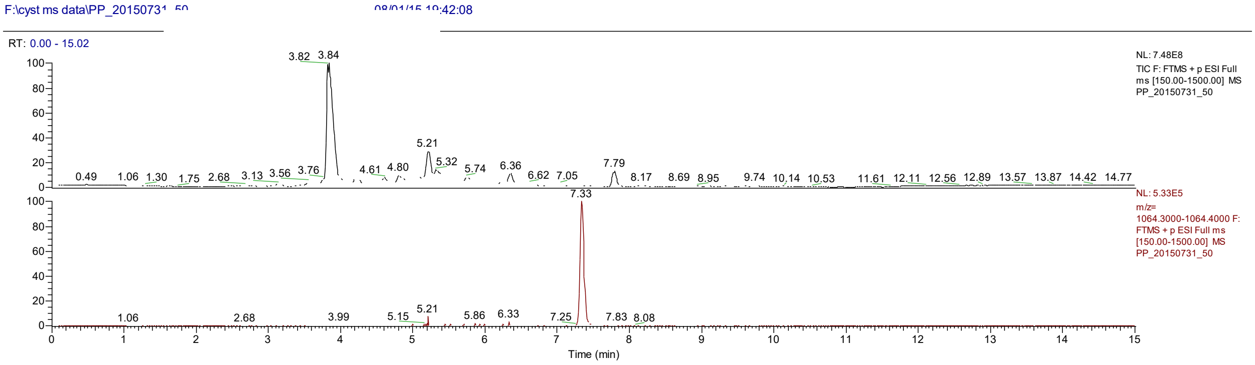

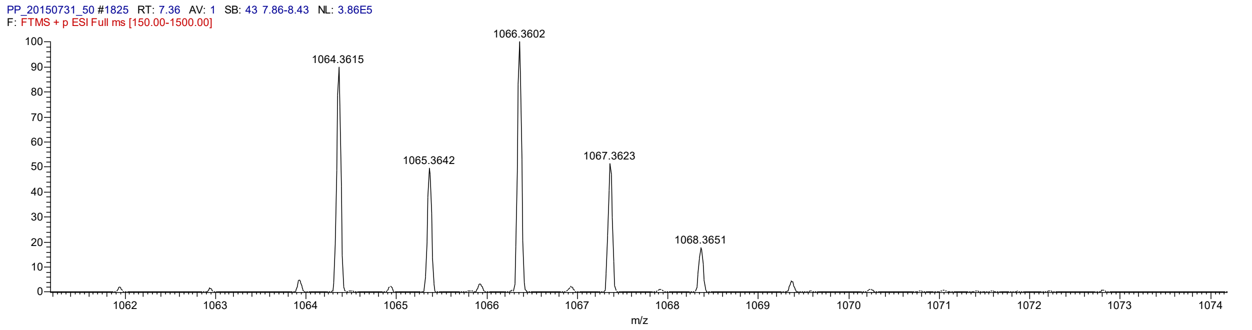

MS2 spectrum and fragment assignment

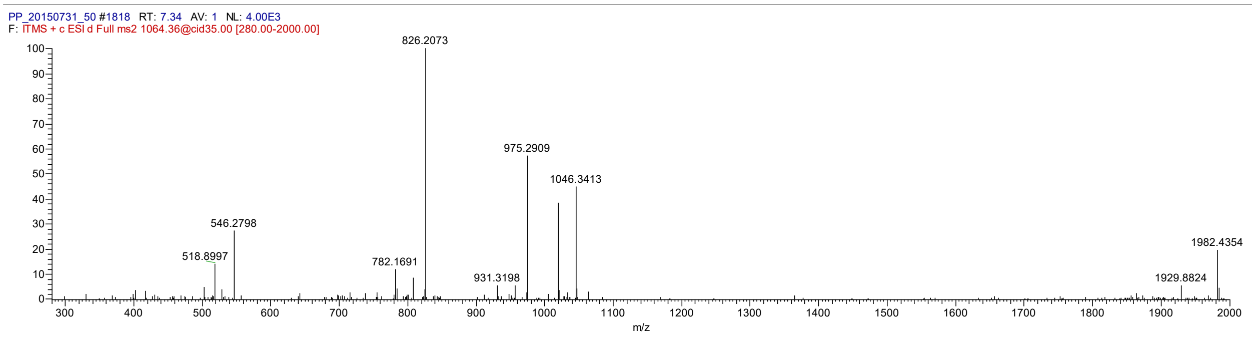

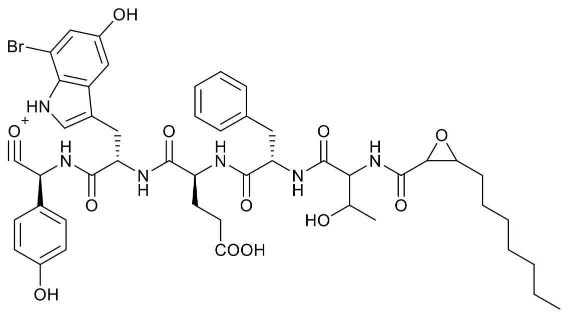

Chemical Formula: $C_{47}H_{56}BrN_6O_{12}^+$
Exact Mass: 975.3134

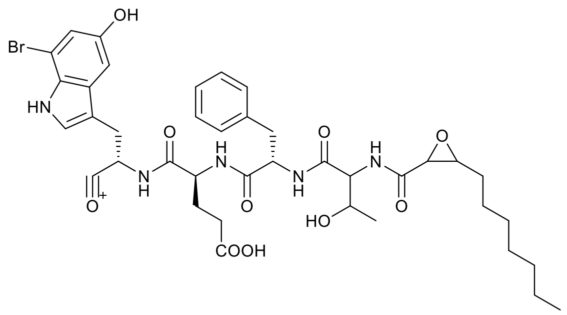

Chemical Formula: $C_{39}H_{49}BrN_5O_{10}^+$
Exact Mass: 826.2657

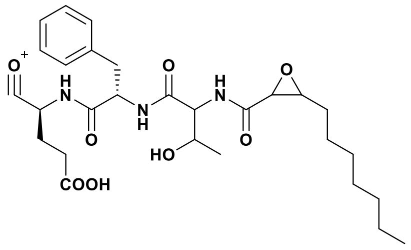

Chemical Formula: $C_{28}H_{40}N_3O_8^+$
Exact Mass: 546.2810

(5ClW) Chlorinated linear cystargamide no hydroxylated

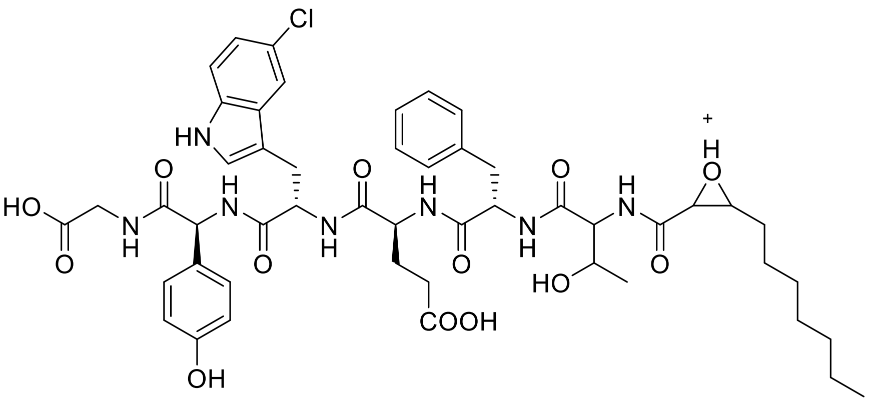

Chemical Formula: $C_{49}H_{61}ClN_7O_{13}^+$
Exact Mass: 990.4010

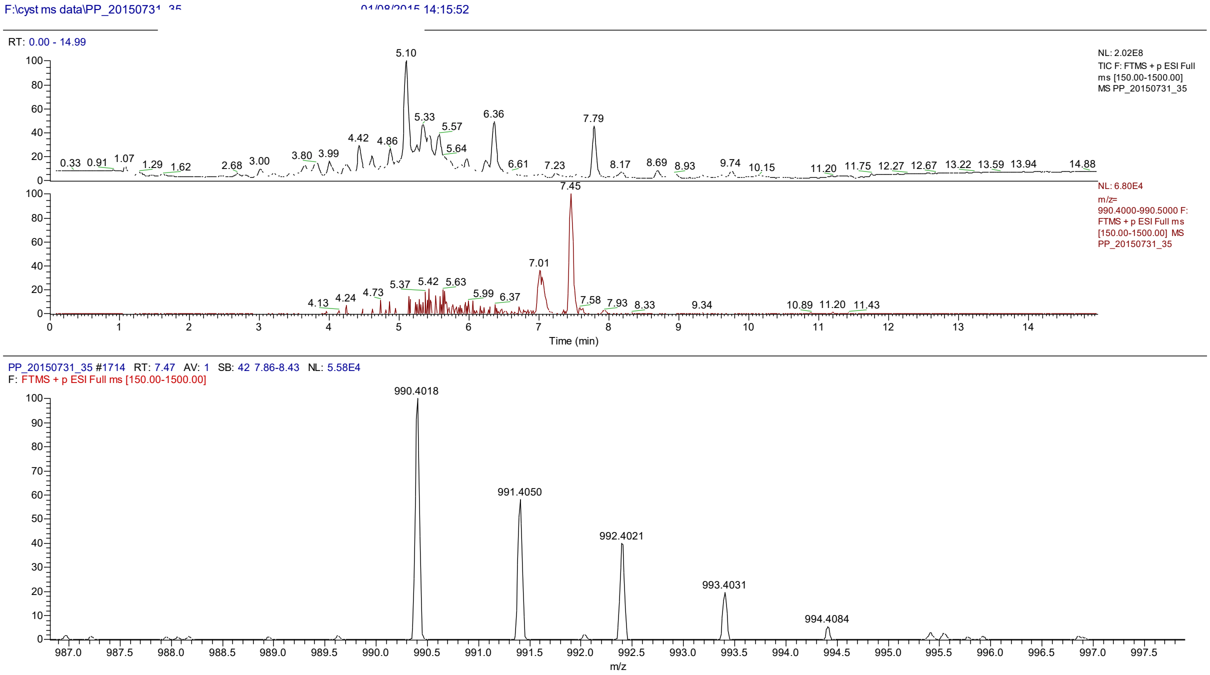

MS2 spectrum and fragment assignment

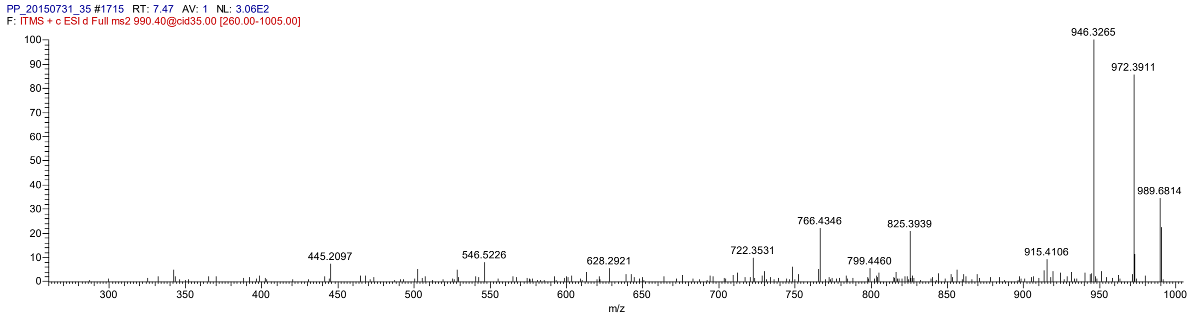

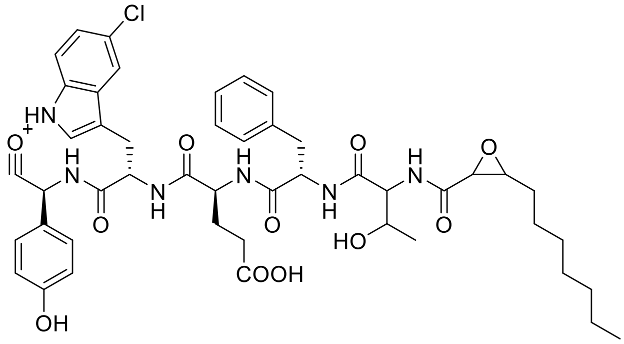

Chemical Formula: $C_{47}H_{56}ClN_6O_{11}^{+}$
Exact Mass: 915.3690

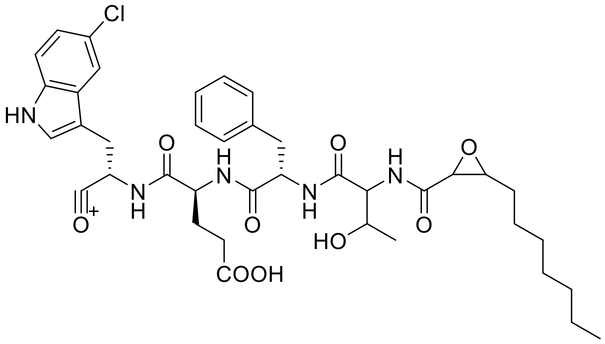

Chemical Formula: $C_{39}H_{49}ClN_5O_9^{+}$
Exact Mass: 766.3213

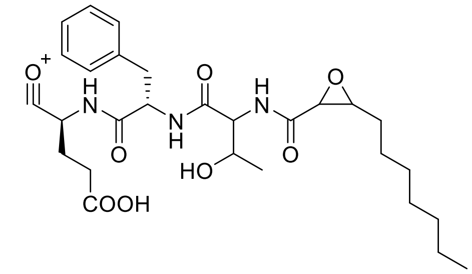

Chemical Formula: $C_{28}H_{40}N_3O_8^{+}$
Exact Mass: 546.2810

(5ClW) Chlorinated linear cystargamide[ALA] unhydroxylated

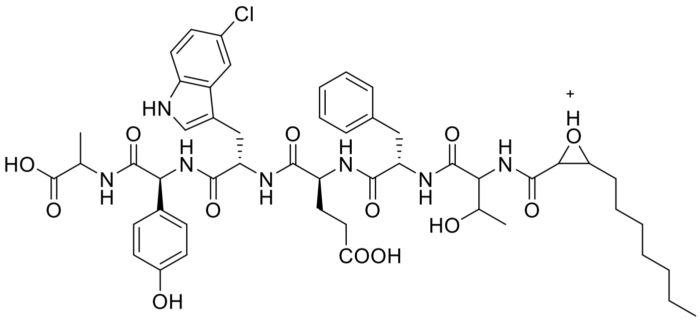

Chemical Formula: $C_{50}H_{63}ClN_7O_{13}^{+}$
Exact Mass: 1004.4167

F:\cyst ms data\PP_20150731_34 01/08/2015 13:58:41

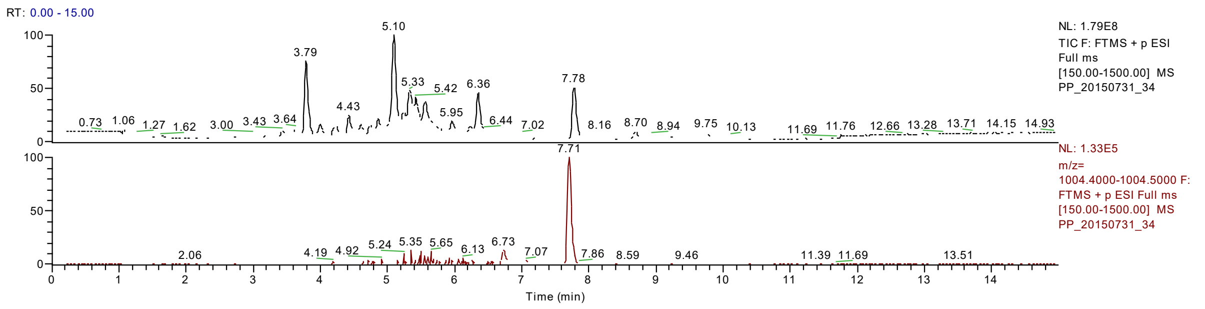

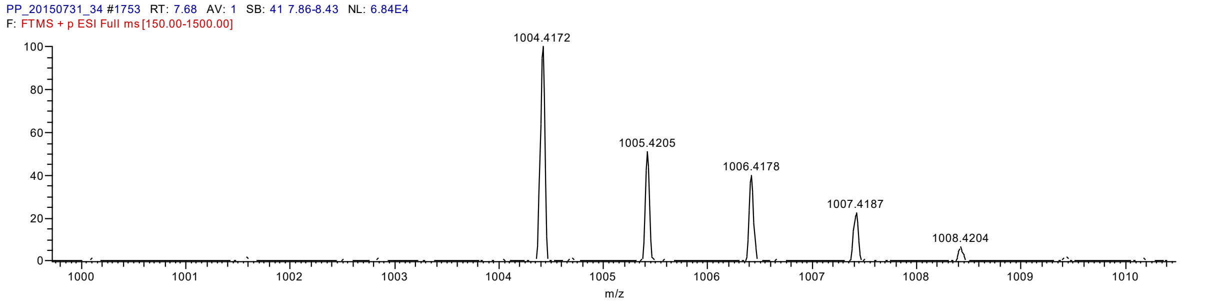

MS2 spectrum and fragment assignment

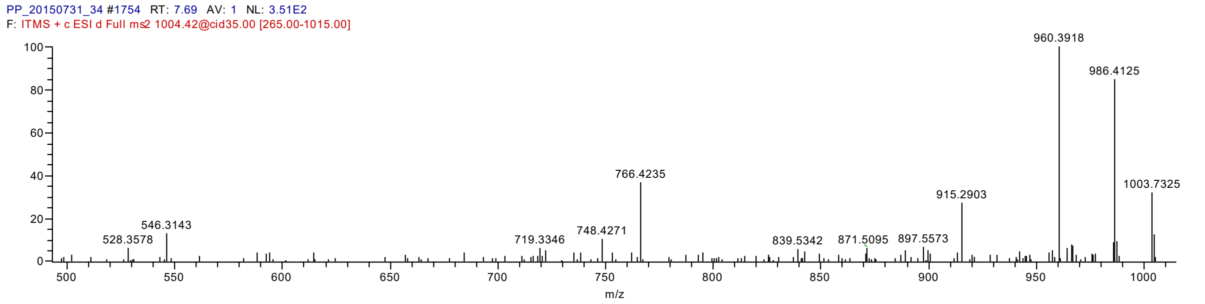

(6ClW) Chlorinated cystargamide un hydroxylated

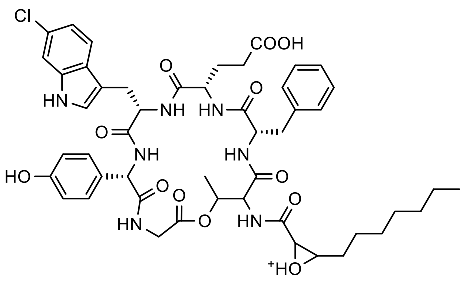

Chemical Formula: $C_{49}H_{59}ClN_7O_{12}^+$

Exact Mass: 972.3905

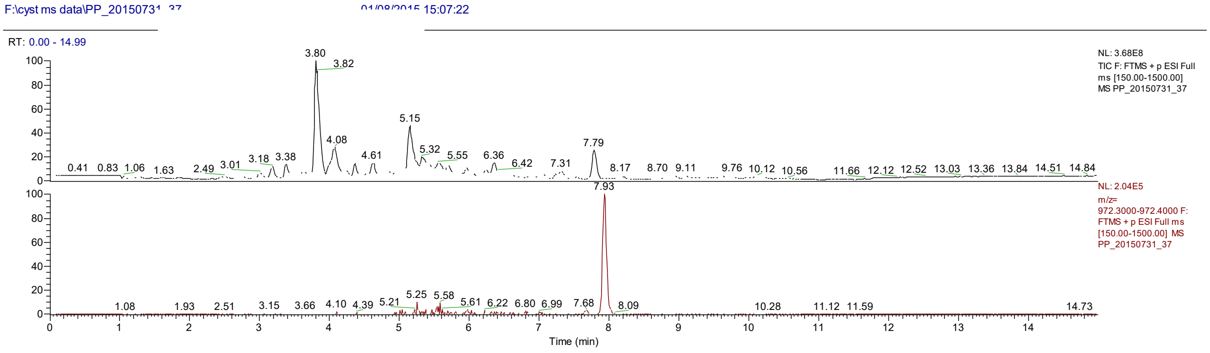

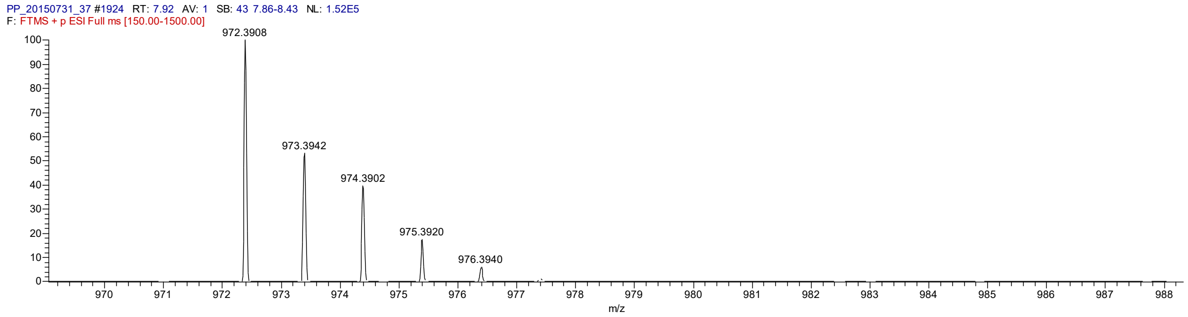

MS2 spectrum and fragment assignment

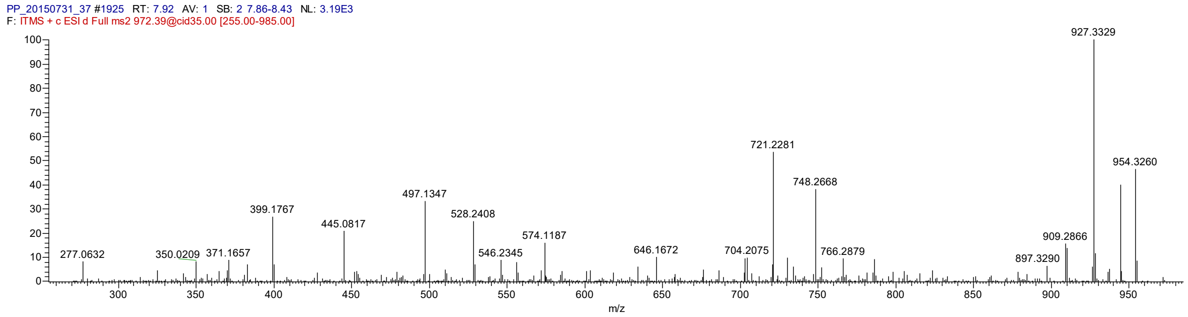

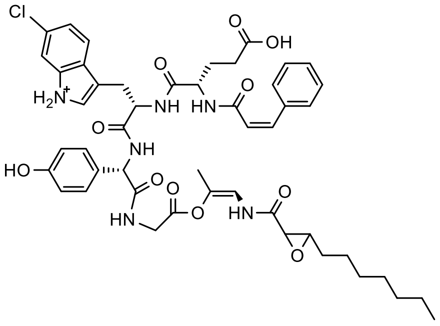

Chemical Formula: $C_{48}H_{56}ClN_6O_{11}^+$
Exact Mass: 927.3690

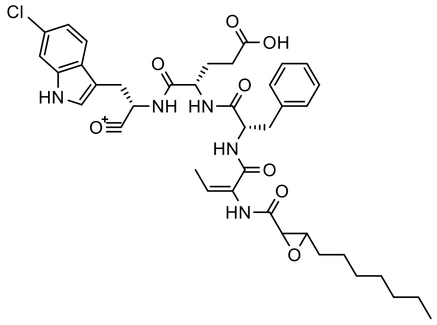

Chemical Formula: $C_{39}H_{47}ClN_5O_8^+$
Exact Mass: 748.3108

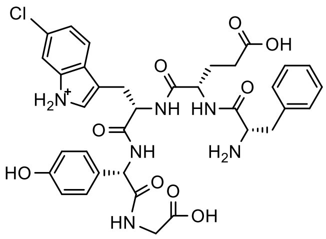

Chemical Formula: $C_{35}H_{38}ClN_6O_9^+$
Exact Mass: 721.2383

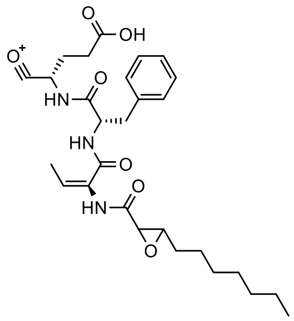

Chemical Formula: $C_{28}H_{38}N_3O_7^+$
Exact Mass: 528.2704

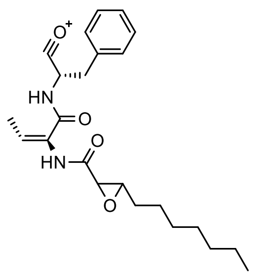

Chemical Formula: $C_{23}H_{31}N_2O_4^+$
Exact Mass: 399.2278

(6ClW) Chlorinated linear cystargamide un hydroxylated

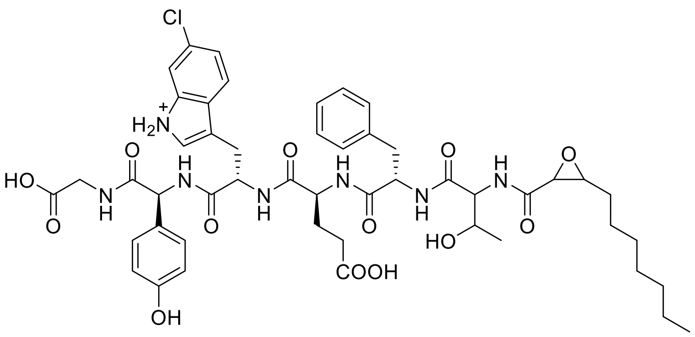

Chemical Formula: $C_{49}H_{61}ClN_7O_{13}^+$
Exact Mass: 990.4010

F:\cyst ms data\PP_20150731_38 08/01/15 15:24:33

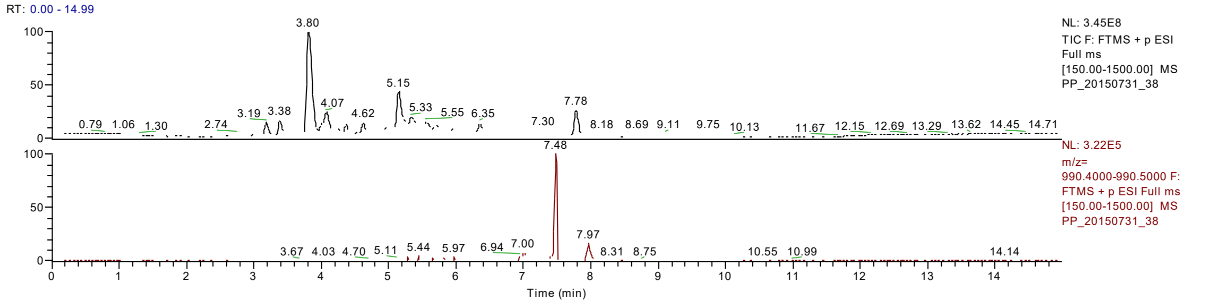

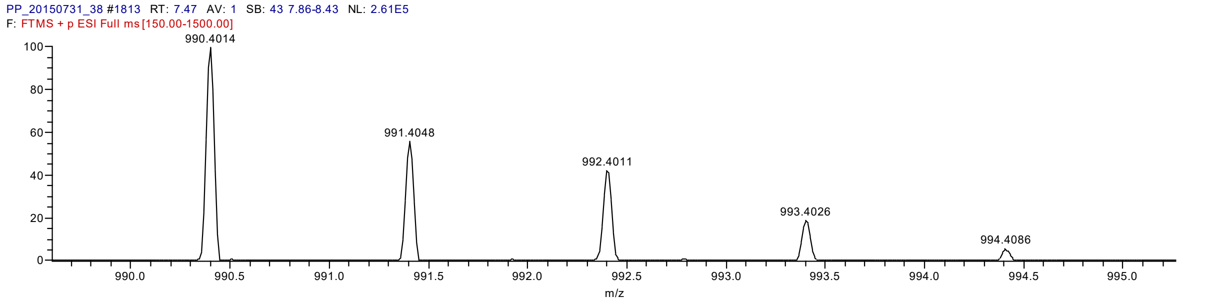

MS2 spectrum and fragment assignment

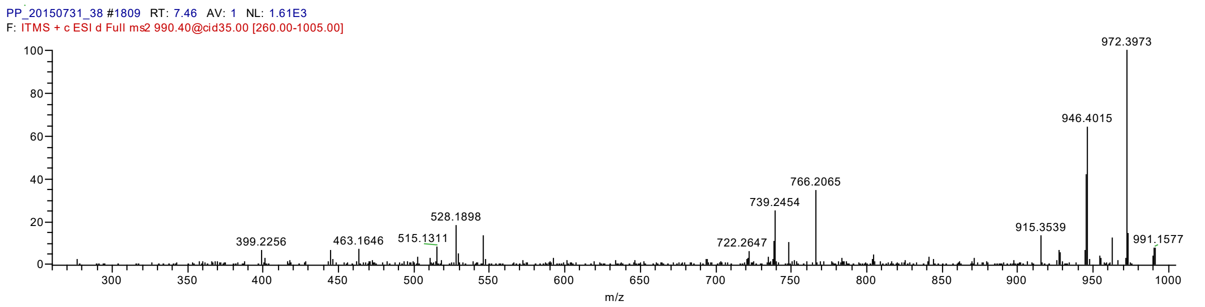

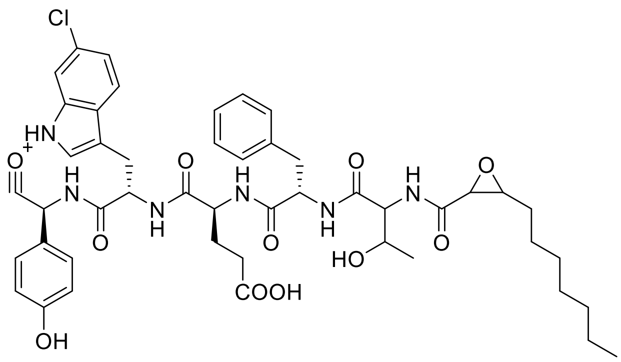

Chemical Formula: $C_{47}H_{56}ClN_6O_{11}^+$
Exact Mass: 915.3690

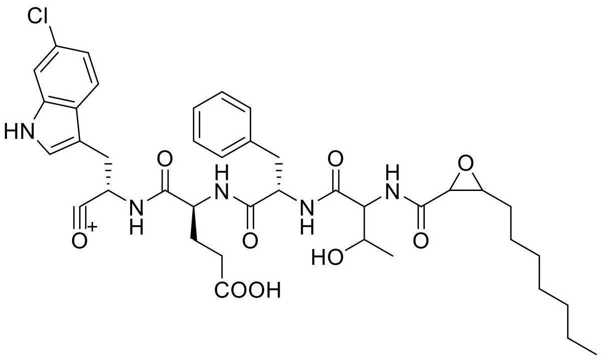

Chemical Formula: $C_{39}H_{49}ClN_5O_9^+$
Exact Mass: 766.3213

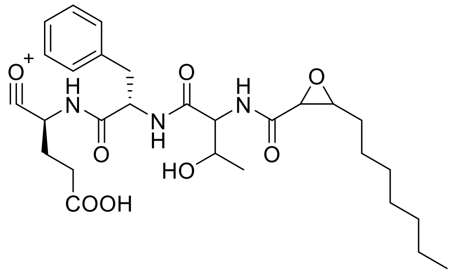

Chemical Formula: $C_{28}H_{40}N_3O_8^+$
Exact Mass: 546.2810

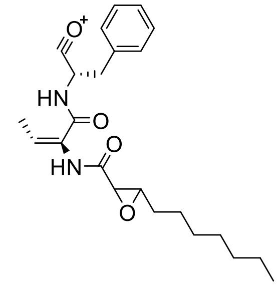

Chemical Formula: $C_{23}H_{31}N_2O_4^+$
Exact Mass: 399.2278

(6ClW) Chlorinated cystargamide

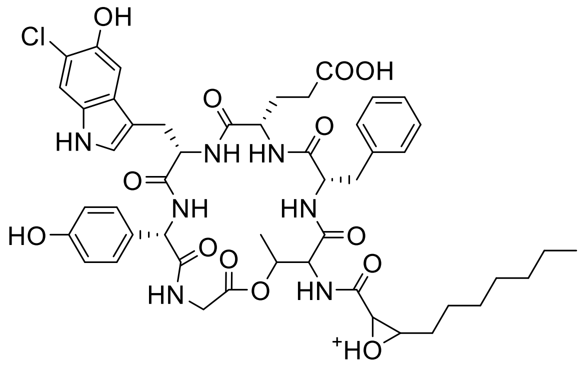

Chemical Formula: $C_{49}H_{59}ClN_7O_{13}^+$
Exact Mass: 988.3854

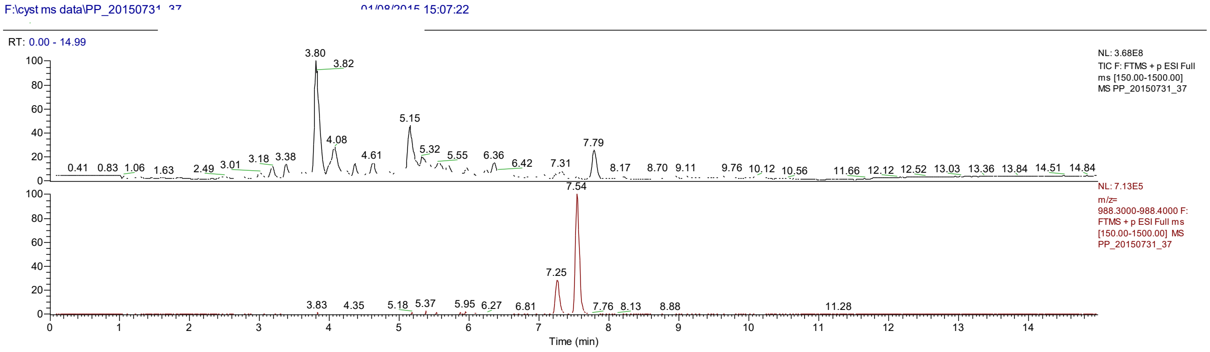

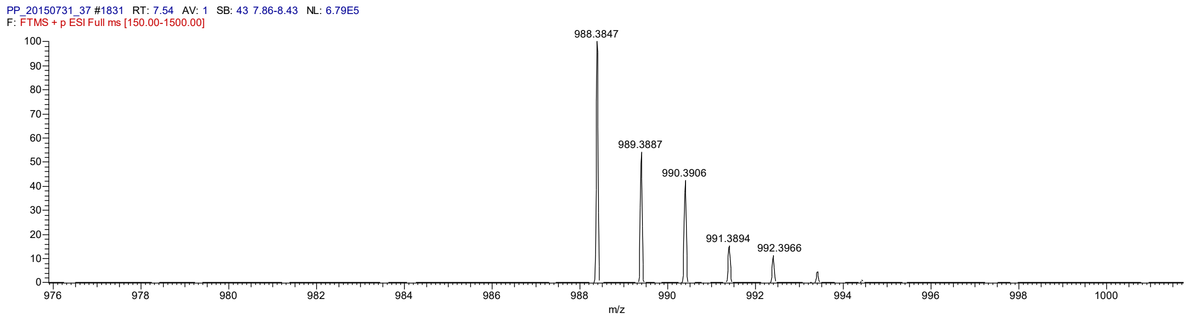

(6ClW) Chlorinated linear cystargamide

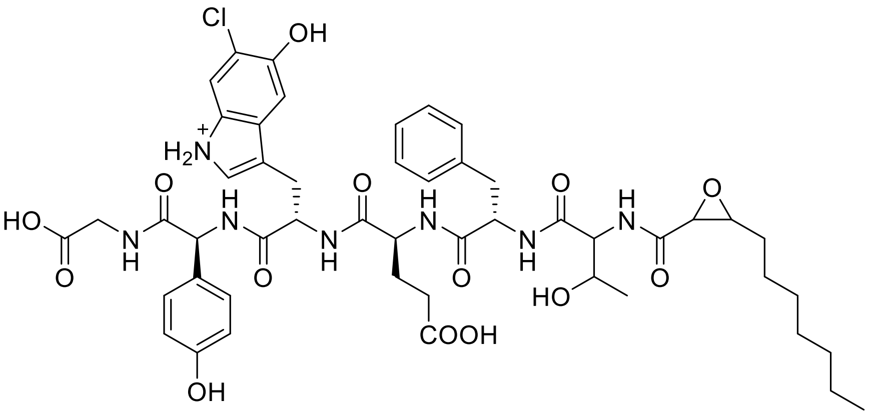

Chemical Formula: $C_{49}H_{61}ClN_7O_{14}^+$

Exact Mass: 1006.3960

F:\cyst ms data\PP_20150731_38 08/01/15 15:24:33

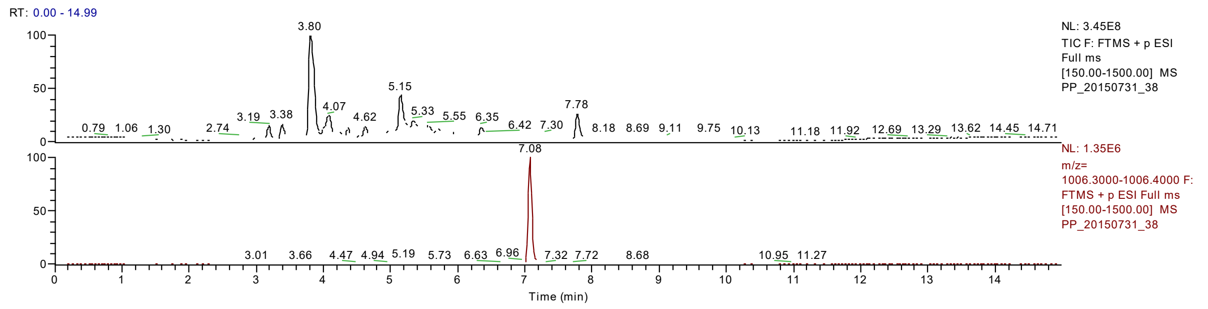

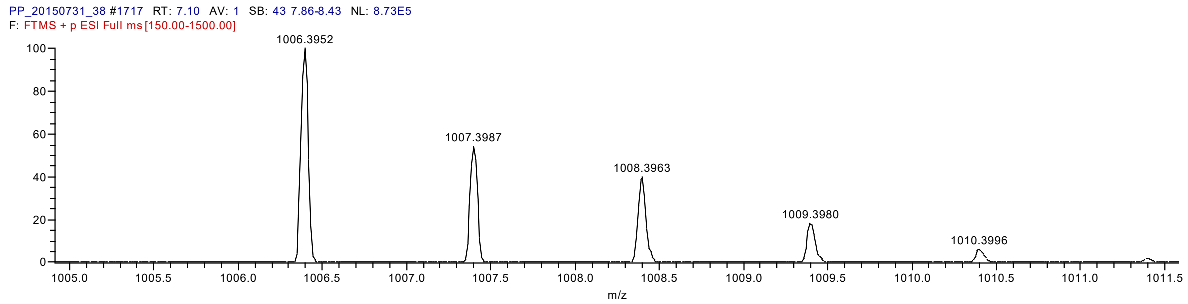

MS2 spectrum and fragment assignment

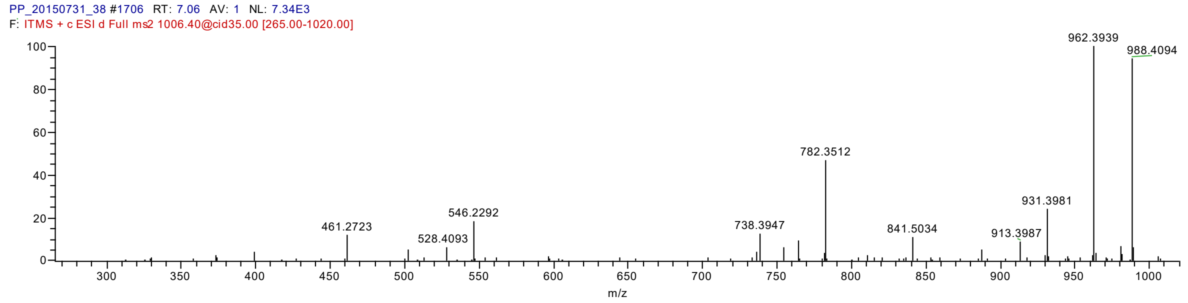

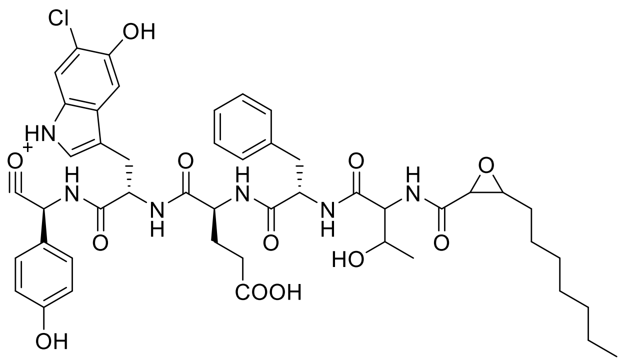

Chemical Formula: $C_{47}H_{56}ClN_6O_{12}^{+}$
Exact Mass: 931.3639

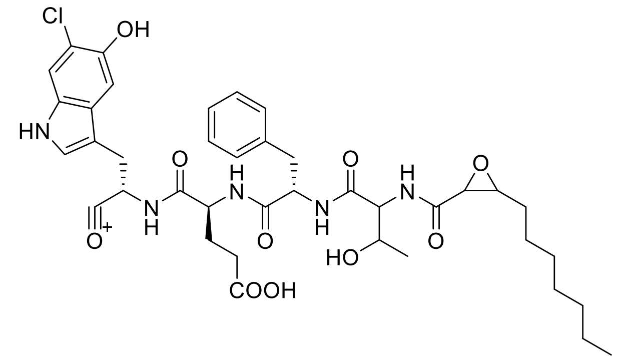

Chemical Formula: $C_{39}H_{49}ClN_5O_{10}^{+}$
Exact Mass: 782.3162

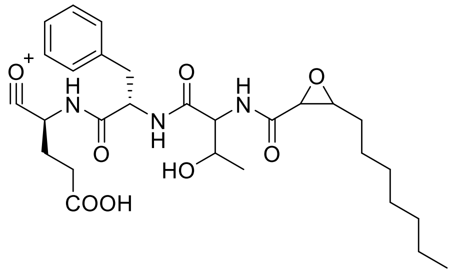

Chemical Formula: $C_{28}H_{40}N_3O_8^{+}$
Exact Mass: 546.2810

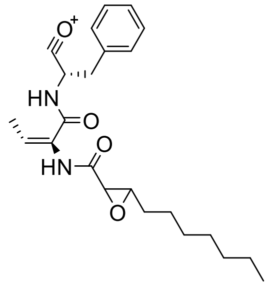

Chemical Formula: $C_{23}H_{31}N_2O_4^{+}$
Exact Mass: 399.2278

(6ClW) Chlorinated linear cystargamide[ALA] un hydroxylated

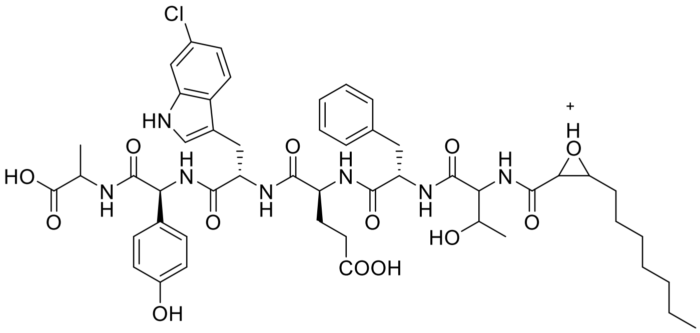

Chemical Formula: $C_{50}H_{63}ClN_7O_{13}^{+}$
Exact Mass: 1004.4167

F:\cyst ms data\PP_20150731_37 01/08/2015 15:07:22

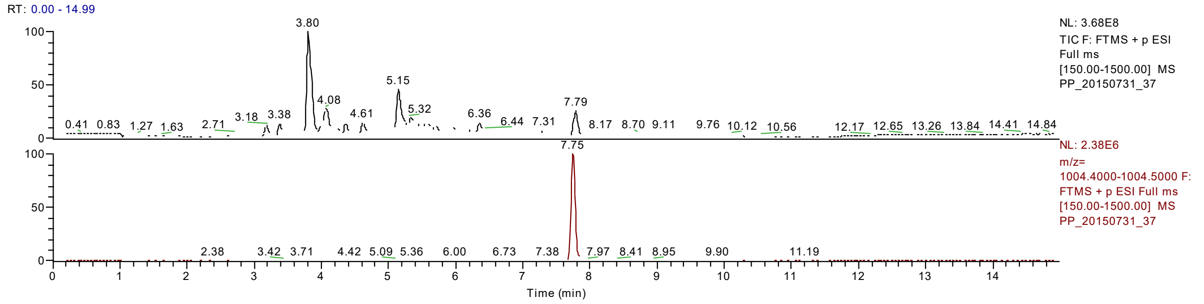

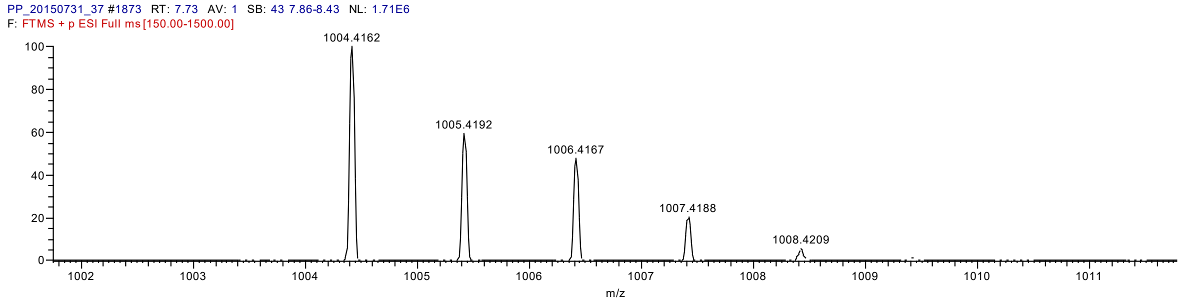

MS2 spectrum and fragment assignment

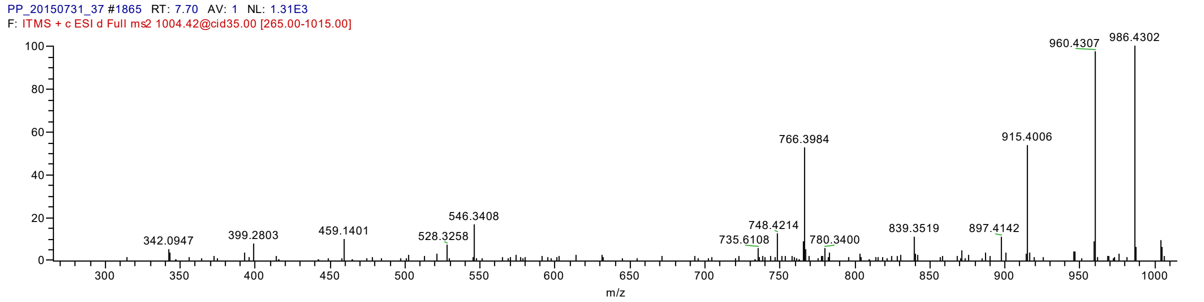

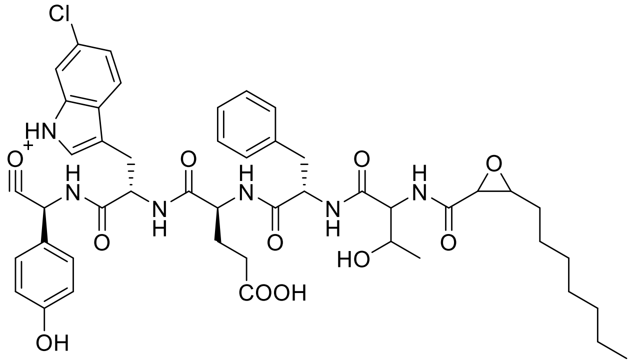

Chemical Formula: $C_{47}H_{56}ClN_6O_{11}^{+}$
Exact Mass: 915.3690

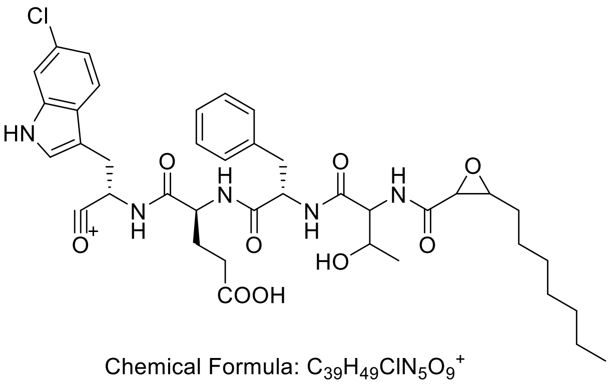

Chemical Formula: $C_{39}H_{49}ClN_5O_9^{+}$
Exact Mass: 766.3213

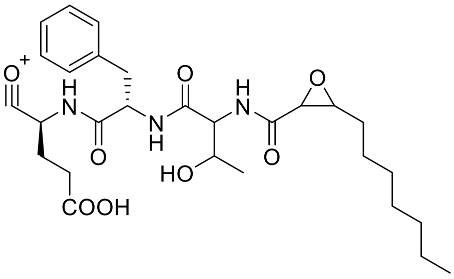

Chemical Formula: $C_{28}H_{40}N_3O_8^{+}$
Exact Mass: 546.2810

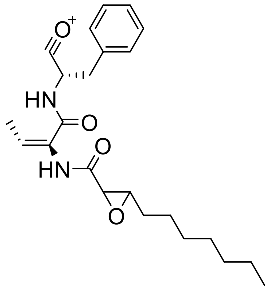

Chemical Formula: $C_{23}H_{31}N_2O_4^{+}$
Exact Mass: 399.2278

(6ClW)-Chlorinated linear cystargamide[ALA]

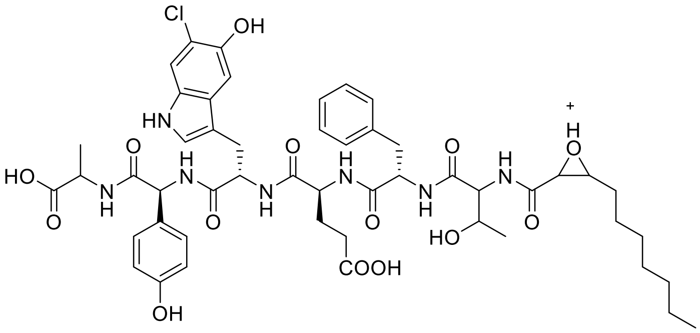

Chemical Formula: $C_{50}H_{63}ClN_7O_{14}^{+}$
Exact Mass: 1020.4116

F:\cyst ms data\PP_20150731_37 01/08/2015 15:07:22

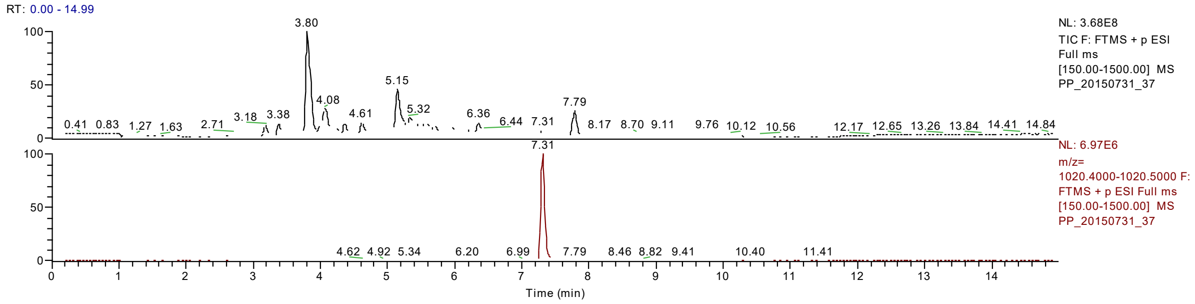

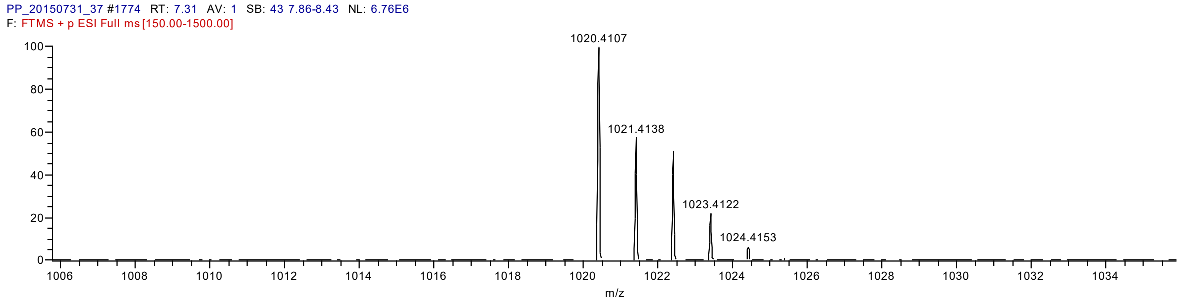

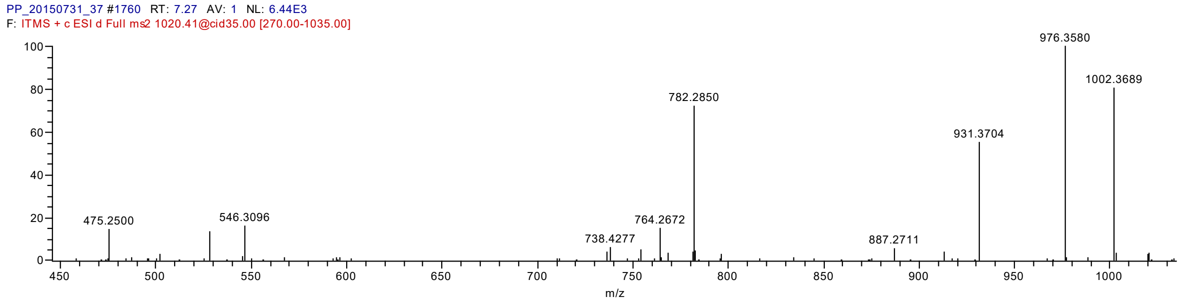

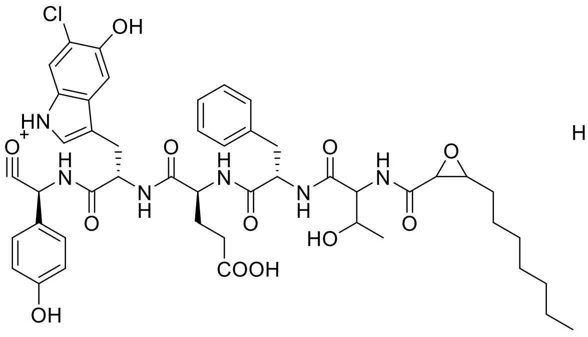

Chemical Formula: $C_{47}H_{56}ClN_6O_{12}^+$
Exact Mass: 931.3639

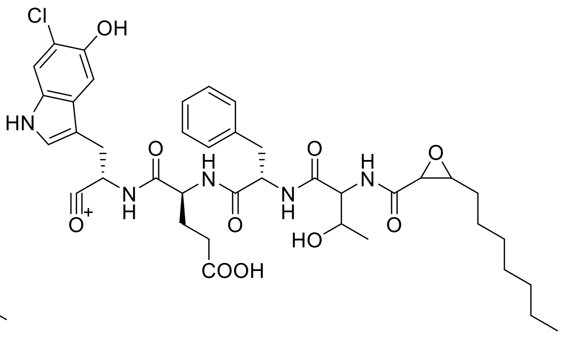

Chemical Formula: $C_{39}H_{49}ClN_5O_{10}^+$
Exact Mass: 782.3162

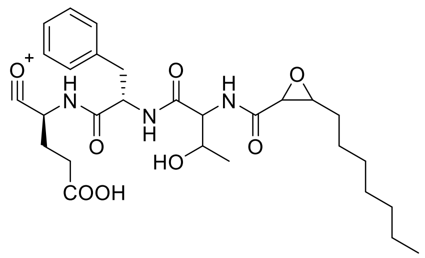

Chemical Formula: $C_{28}H_{40}N_3O_8^+$
Exact Mass: 546.2810

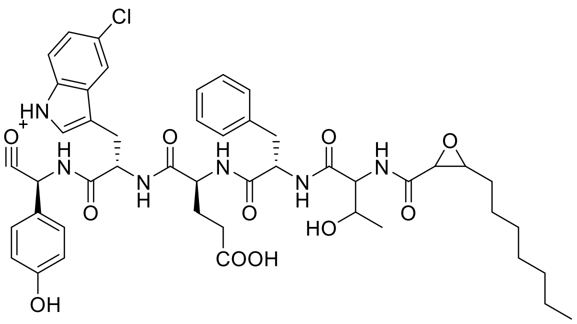

Chemical Formula: $C_{47}H_{56}ClN_6O_{11}^+$
Exact Mass: 915.3690

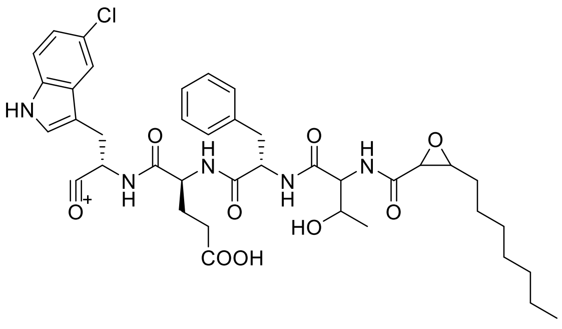

Chemical Formula: $C_{39}H_{49}ClN_5O_9^+$
Exact Mass: 766.3213

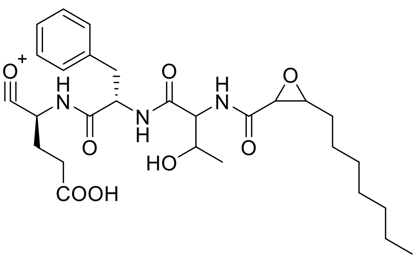

Chemical Formula: $C_{28}H_{40}N_3O_8^+$
Exact Mass: 546.2810

(7ClW) Chlorinated cystargamide unhydroxylated

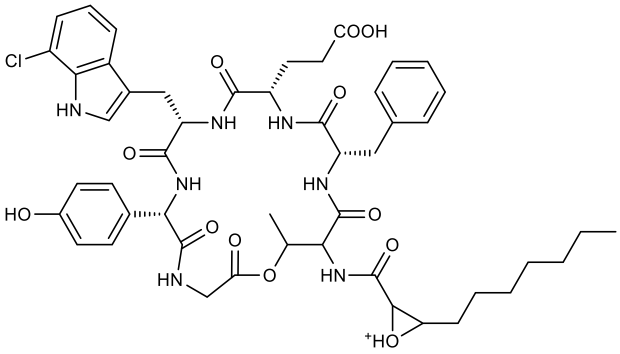

Chemical Formula: $C_{49}H_{59}ClN_7O_{12}^+$
Exact Mass: 972.3905

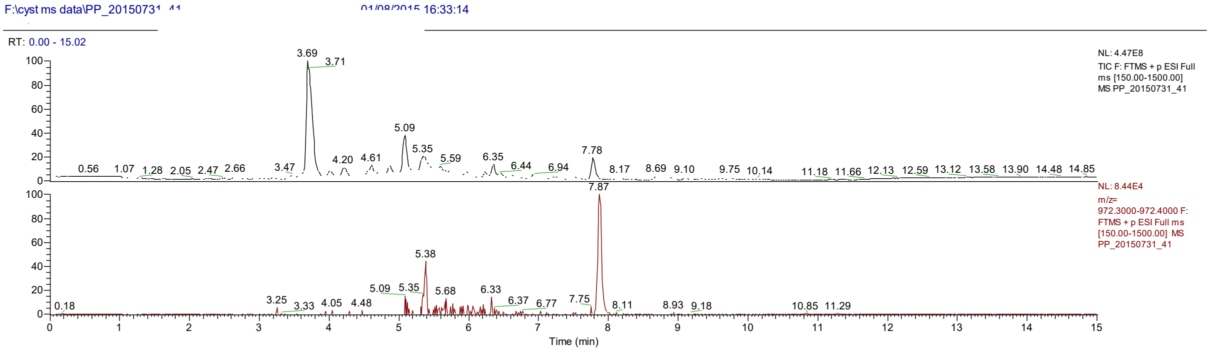

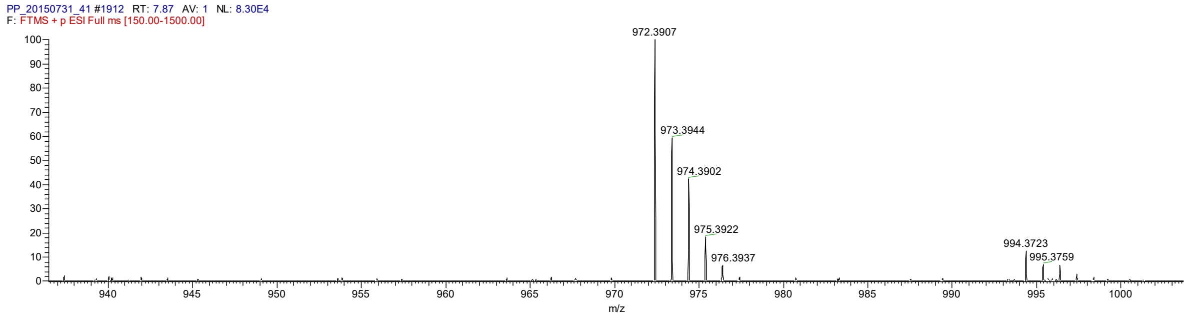

MS2 spectrum and fragment assignment

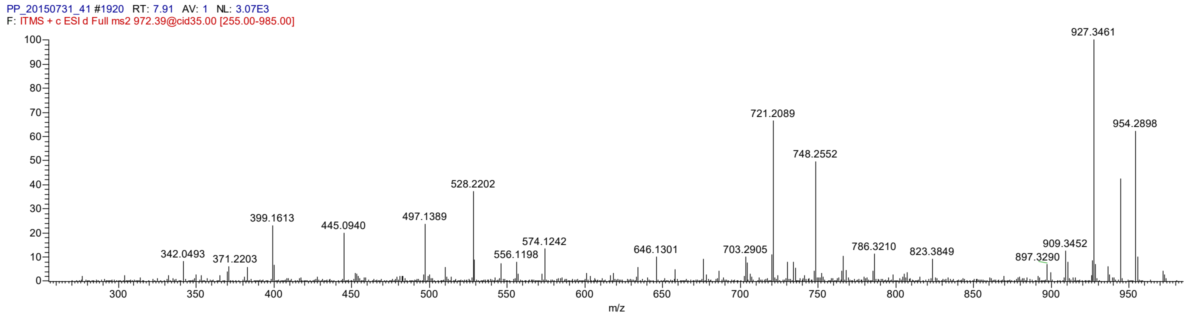

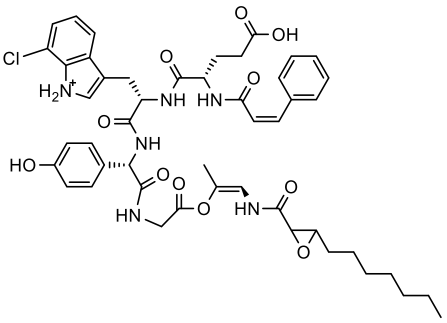

Chemical Formula: $C_{48}H_{56}ClN_6O_{11}^+$
Exact Mass: 927.3690

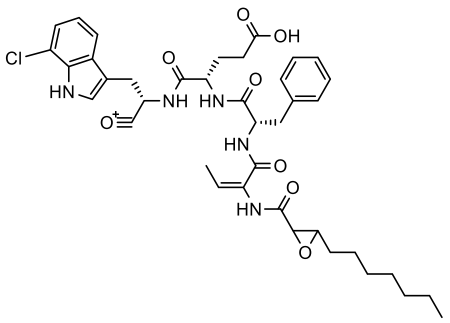

Chemical Formula: $C_{39}H_{47}ClN_5O_8^+$
Exact Mass: 748.3108

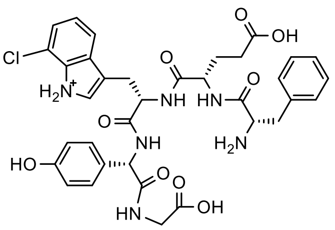

Chemical Formula: $C_{35}H_{38}ClN_6O_9^+$
Exact Mass: 721.2383

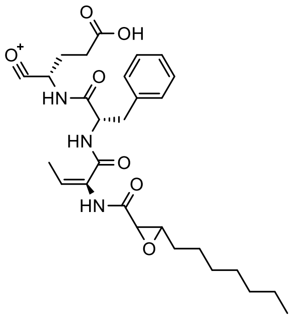

Chemical Formula: $C_{28}H_{38}N_3O_7^+$
Exact Mass: 528.2704

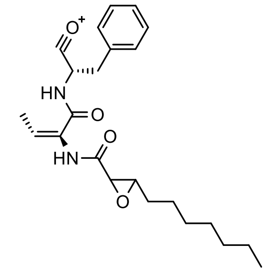

Chemical Formula: $C_{23}H_{31}N_2O_4^+$
Exact Mass: 399.2278

(7ClW) Chlorinated linear cystargamide un hydroxylated

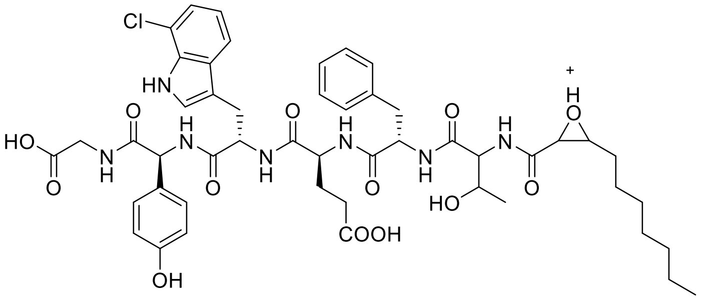

Chemical Formula: $C_{49}H_{61}ClN_7O_{13}^+$
Exact Mass: 990.4010

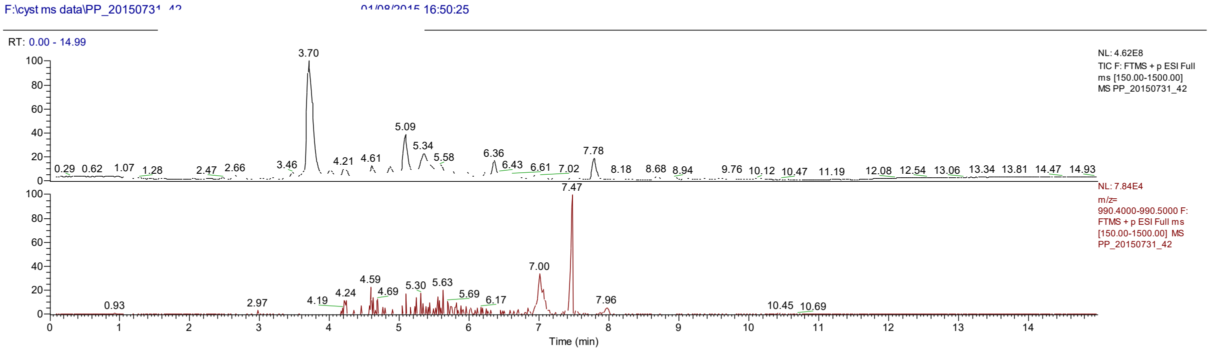

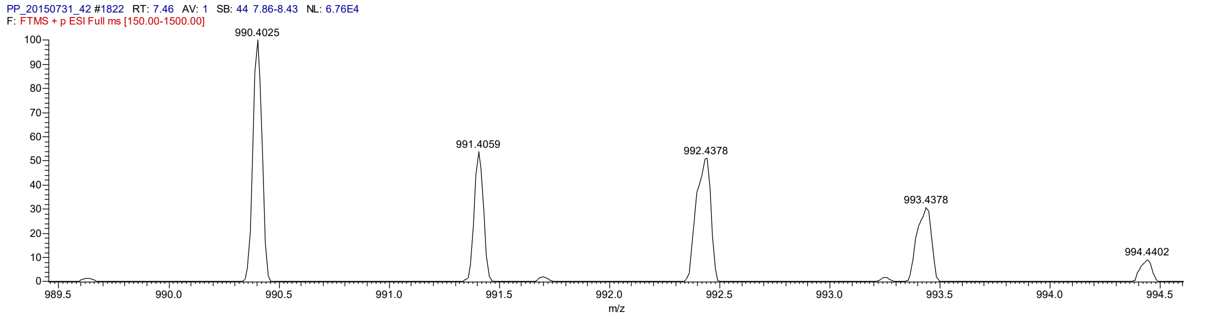

MS2 spectrum and fragment assignment

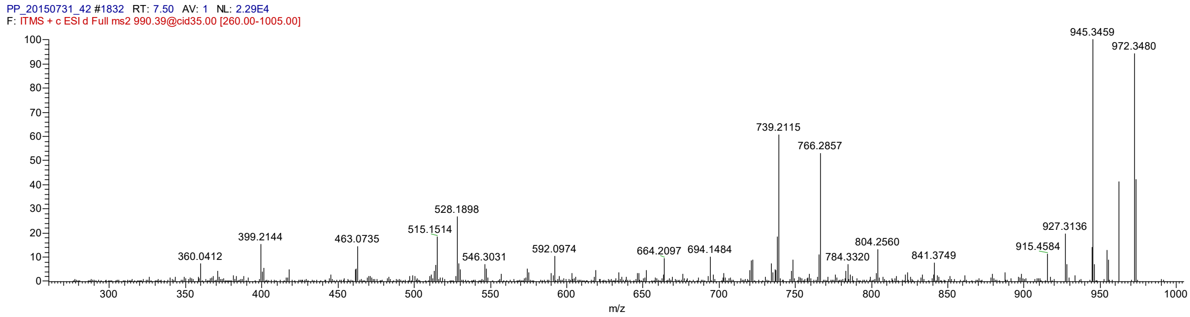

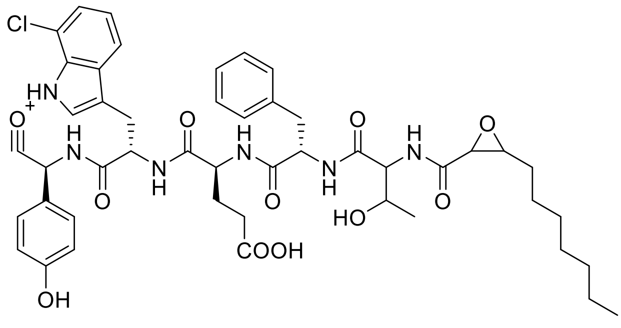

Chemical Formula: $C_{47}H_{56}ClN_6O_{11}^+$
Exact Mass: 915.3690

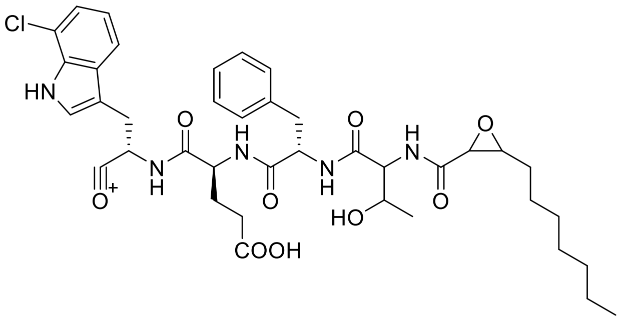

Chemical Formula: $C_{39}H_{49}ClN_5O_9^+$
Exact Mass: 766.3213

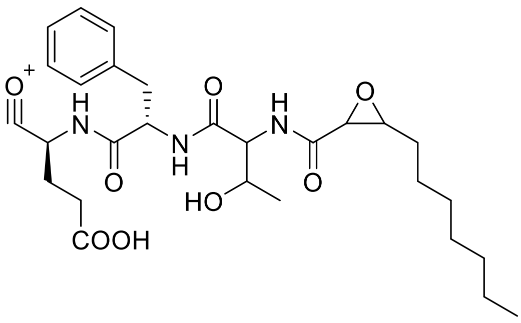

Chemical Formula: $C_{28}H_{40}N_3O_8^+$
Exact Mass: 546.2810

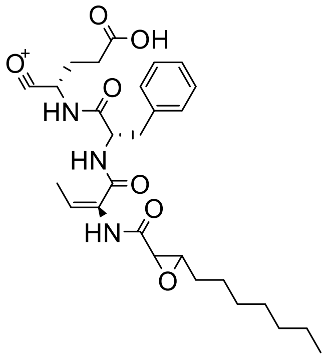

Chemical Formula: $C_{28}H_{38}N_3O_7^+$
Exact Mass: 528.2704

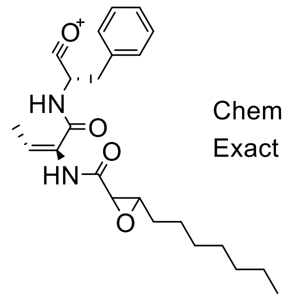

Chemical Formula: $C_{23}H_{31}N_2O_4^+$
Exact Mass: 399.2278

(7ClW) Chlorinated cystargamide

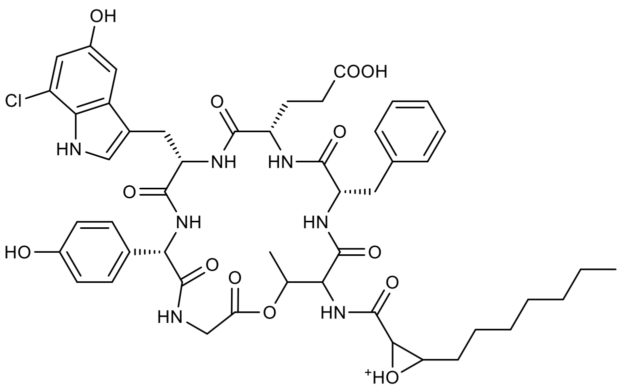

Chemical Formula: $C_{49}H_{59}ClN_7O_{13}^+$
Exact Mass: 988.3854

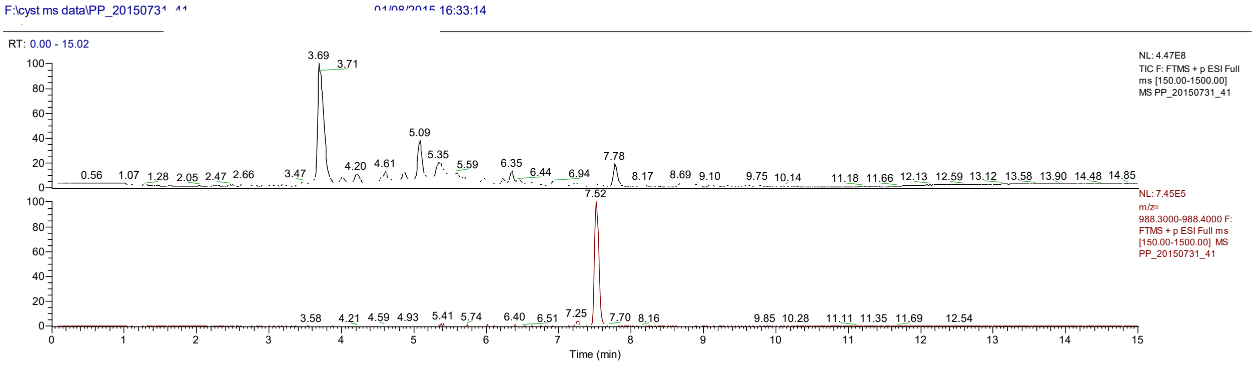

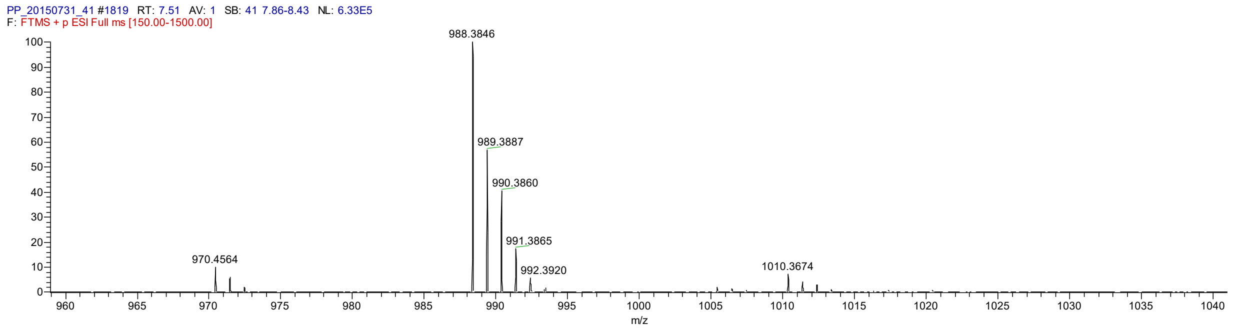

(7ClW) Chlorinated linear cystargamide

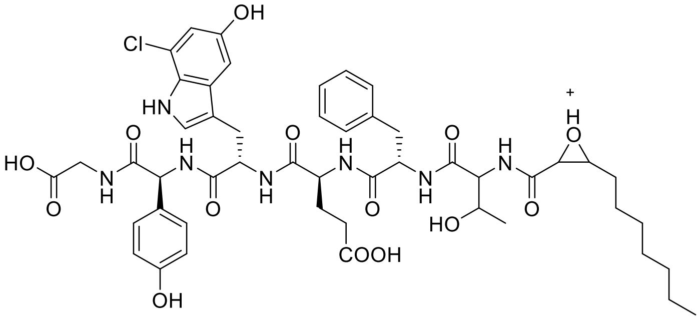

Chemical Formula: $C_{49}H_{61}ClN_7O_{14}^+$

Exact Mass: 1006.3960

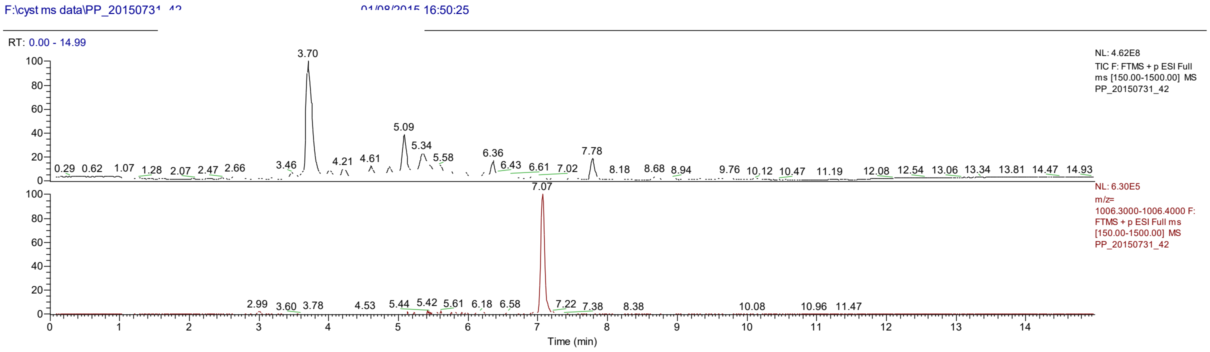

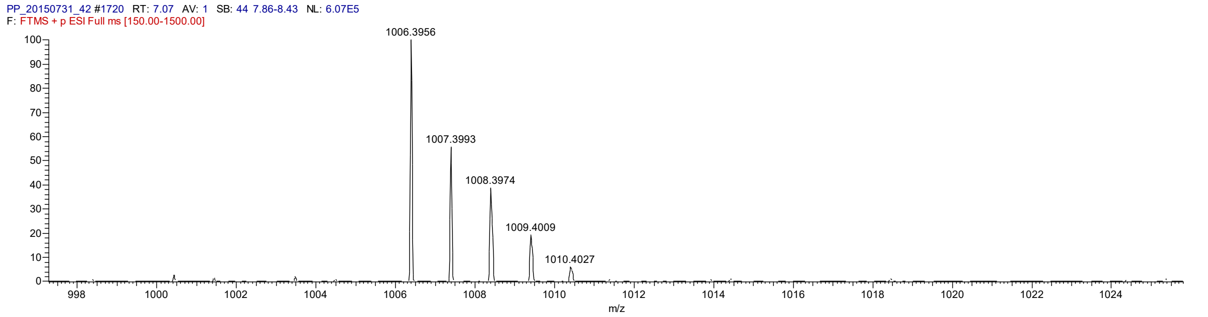

MS2 spectrum and fragment assignment

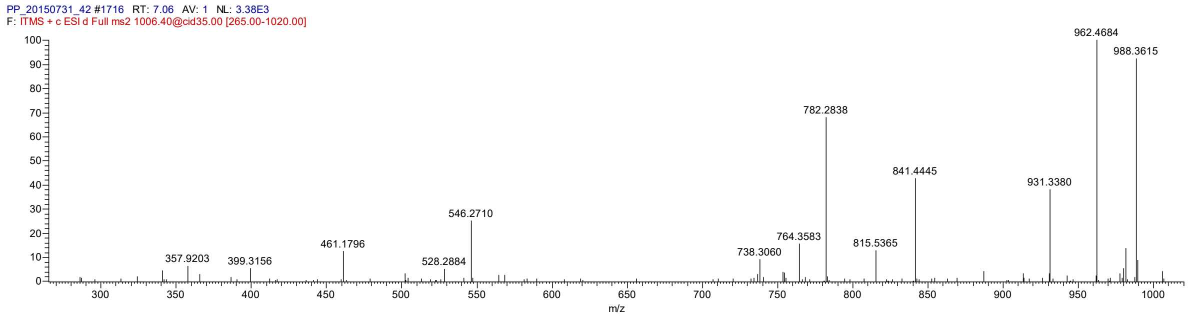

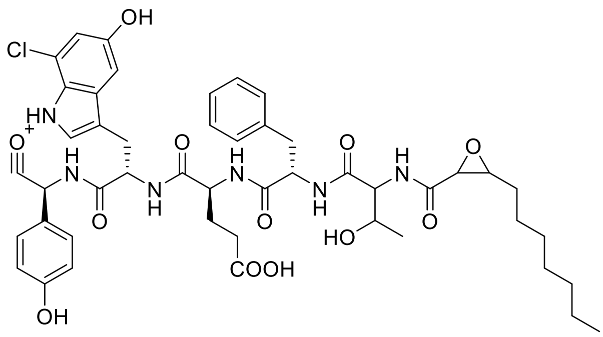

Chemical Formula: $C_{47}H_{56}ClN_6O_{12}^{+}$
Exact Mass: 931.3639

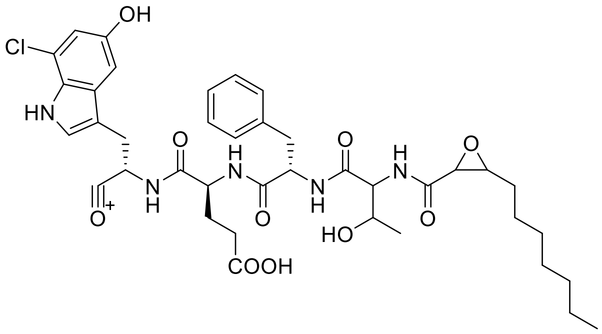

Chemical Formula: $C_{39}H_{49}ClN_5O_{10}^{+}$
Exact Mass: 782.3162

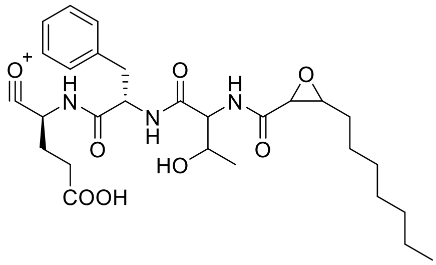

Chemical Formula: $C_{28}H_{40}N_3O_8^{+}$
Exact Mass: 546.2810

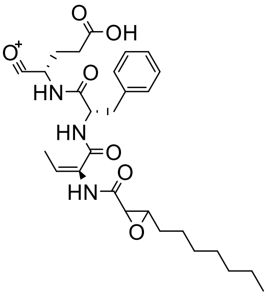

Chemical Formula: $C_{28}H_{38}N_3O_7^{+}$
Exact Mass: 528.2704

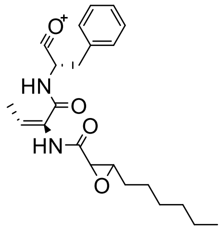

Chemical Formula: $C_{23}H_{33}N_2O_4^{+}$
Exact Mass: 401.2435

(7ClW) Chlorinated linear cystargamide[ALA] unhydroxylated

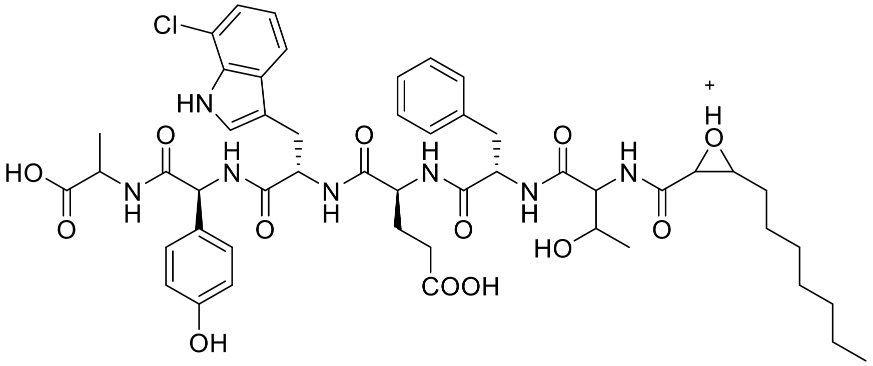

Chemical Formula: $C_{50}H_{63}ClN_{7}O_{13}^{+}$
Exact Mass: 1004.4167

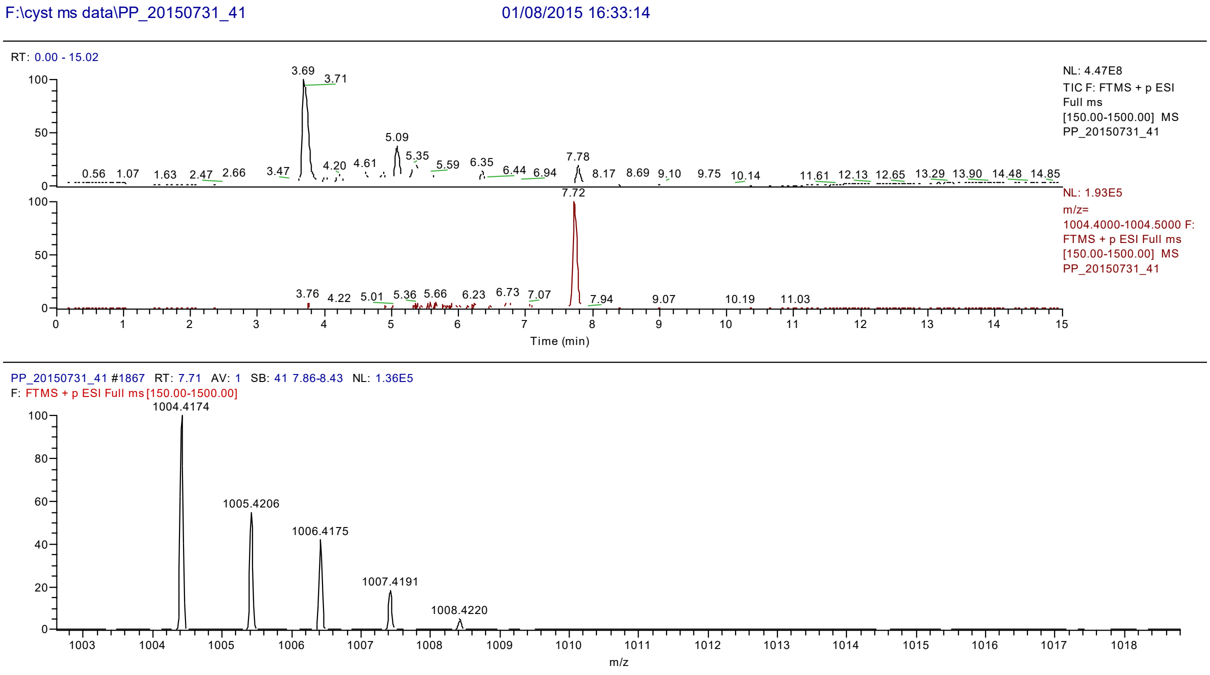

MS2 spectrum and fragment assignment

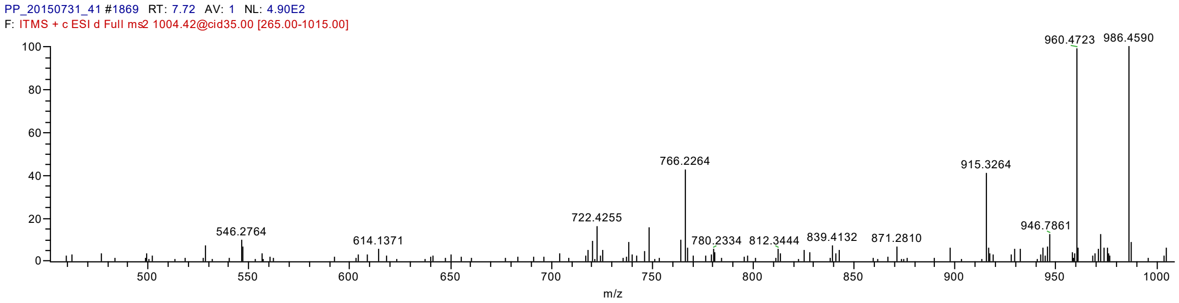

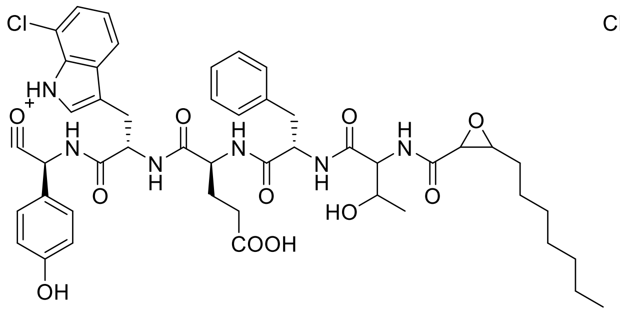

Chemical Formula: $C_{47}H_{56}ClN_6O_{11}^+$
Exact Mass: 915.3690

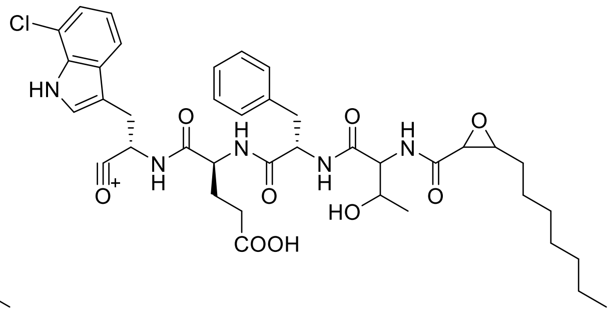

Chemical Formula: $C_{39}H_{49}ClN_5O_9^+$
Exact Mass: 766.3213

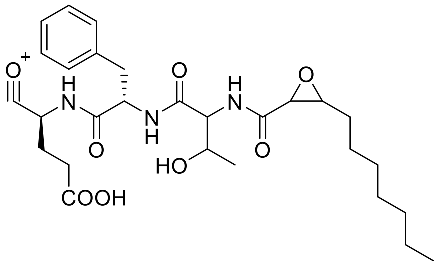

Chemical Formula: $C_{28}H_{40}N_3O_8^+$
Exact Mass: 546.2810

(7ClW) Chlorinated linear cystargamide[ALA]

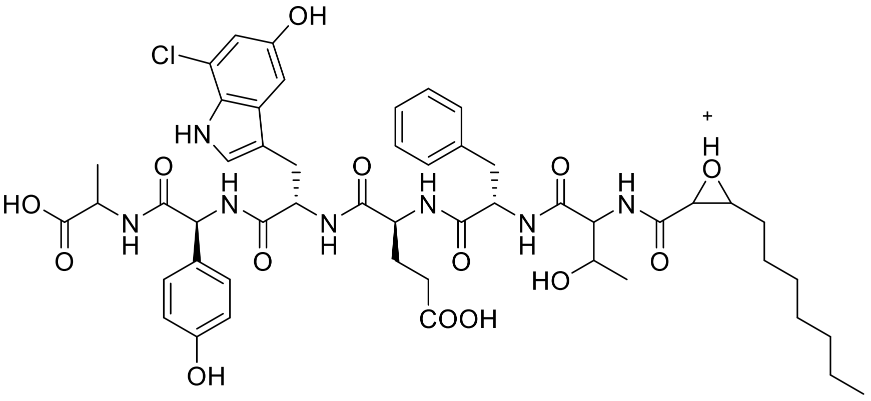

Chemical Formula: $C_{50}H_{63}ClN_7O_{14}^{+}$
Exact Mass: 1020.4116

F:\cyst ms data\PP_20150731_41   01/08/2015 16:33:14

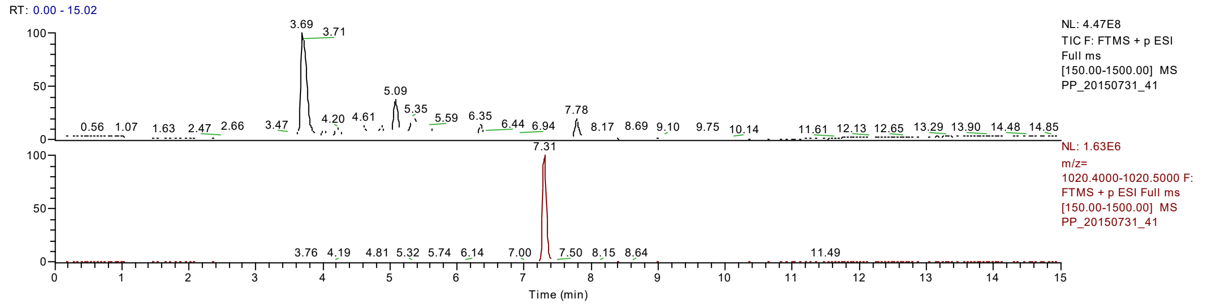

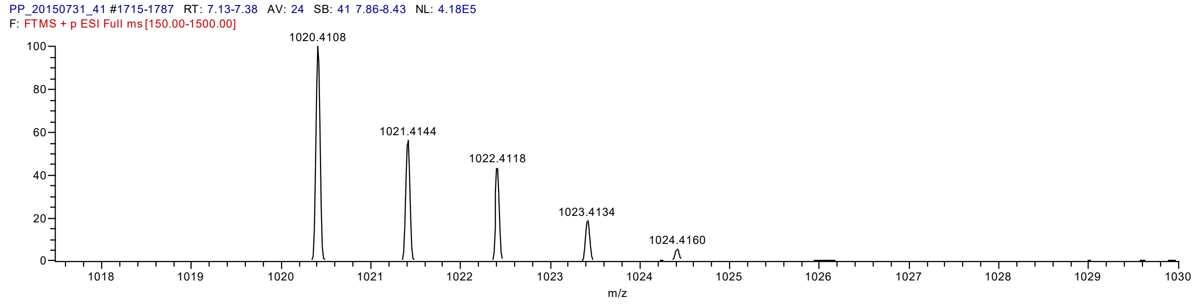

MS2 spectrum and fragment assignment

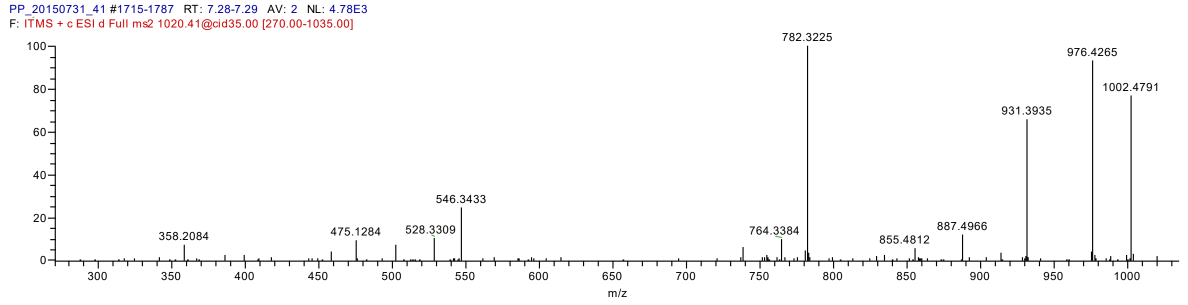

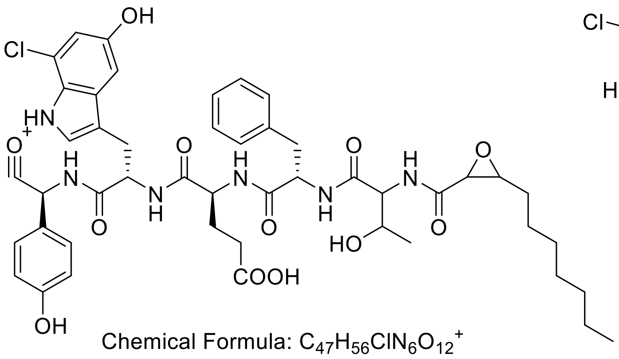

Chemical Formula: $C_{47}H_{56}ClN_6O_{12}^{+}$
Exact Mass: 931.3639

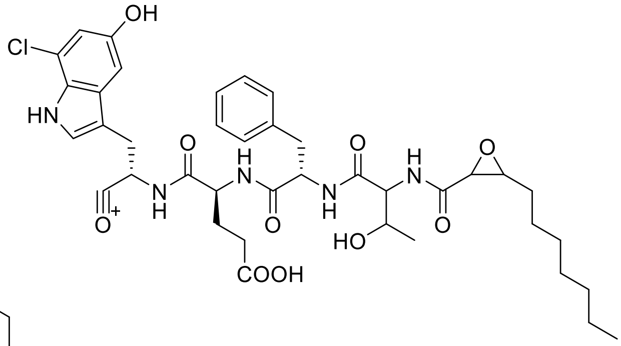

Chemical Formula: $C_{39}H_{49}ClN_5O_{10}^{+}$
Exact Mass: 782.3162

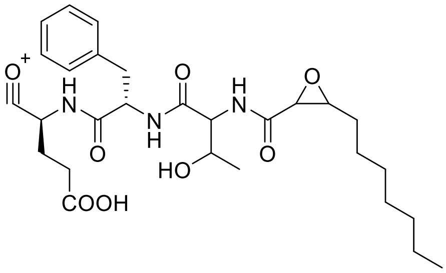

Chemical Formula: $C_{28}H_{40}N_3O_8^{+}$
Exact Mass: 546.2810

Cross coupling reaction on Br-Cystargamide

sXPhos **12** (0.6 mg, 1.2 μmol) and $CsCO_3$ (6.0 mg, 20 μmol) were added to a microwave tube and flushed with nitrogen. A stock solution of *bis*(acetonitrile)dichloropalladium (II) (0.8 mg, 4 μmol) in degassed H_2O:CH_3CN (1:1, 100 μL) was prepared. The catalyst stock solution (10 μL, 0.4 μmol) was added to the microwave tube, followed by a solution of the Br-Cystargamide **9** (1 mg) in H_2O:CH_3CN (1:1, 0.2 mL). 3-Fluorophenylacetylene **24** (10 μL) was then added. The microwave tube was flushed with nitrogen, then sealed. The reaction mixture was heated at 100 °C for 2 h. The reaction mixture was colled to r.t. A portion of the reaction mixture (0.1 mL) was removed and diluted with H_2O:CH_3CN (1:1, 0.2 mL). Polystyrene supported triphenylphosphine (5 mg) was added the sample was well shaken. The sample was filtered through a plug of cotton wool, then centrifuged (13,000 rpm, 16060 g, 5 min). The reaction mixture (30 μL) diluted with H_2O:CH_3CN (9:1, 970 µL) and analysed by LC-MS.

LC-MS analysis showed signals consistent with the product **64** at 1072 $[M+H]^+$, as well as the hydrolysed linear form of the peptide **65** at 1090 $[M+H]^+$.

^{19}F NMR (470 MHz, D_2O) δ= -114.05 (1F, ddd, *J*(H,F)= 9.9, 9.3, 5.7 Hz), -114.43 (1F, ddd, *J*(H,F)= 9.7, 9.6, 6.3 Hz); Assignment of ^{19}F NMR signals to the cyclized and linear products respectively is tentative, and based on the observed ratios by LC-MS.

Compound **65** MS (ESI) 1090 (50) $[M+H]^+$, 1046 (40), 641 (40), 515 (100), 376 (50); HRMS: *m*/*z* calcd for $C_{57}H_{65}F_1N_7O_{14}$ $[M+H]^+$: 1090.4568; found: 1090.4562;

Compound **64** MS (ESI) 1072 (30) $[M+H]^+$, 1028 (20), 635 (40), 567 (20), 421 (60), 376 (100); HRMS: *m*/*z* calcd for $C_{57}H_{63}F_1N_7O_{13}$ $[M+H]^+$: 1072.4462; found: 1072.4464.

Cross Coupled Cyclised Cystargamide (64)

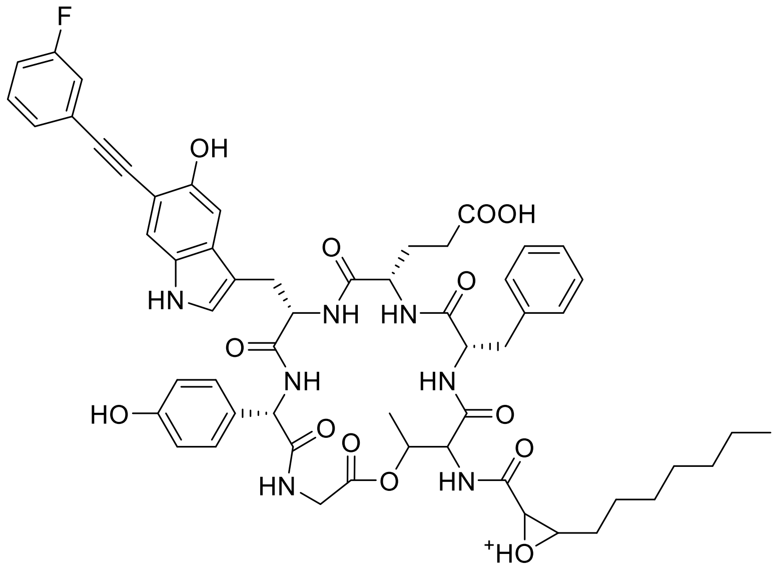

F:\160217MJC20503 02/17/16 14:31:50 MJC 205

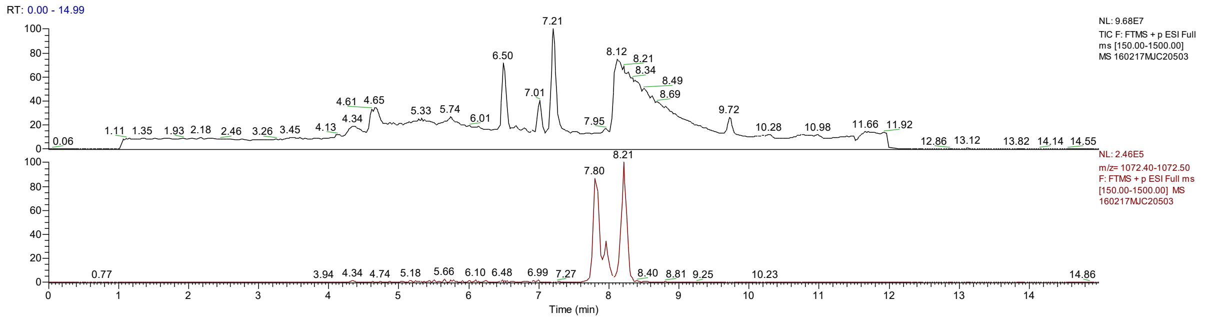

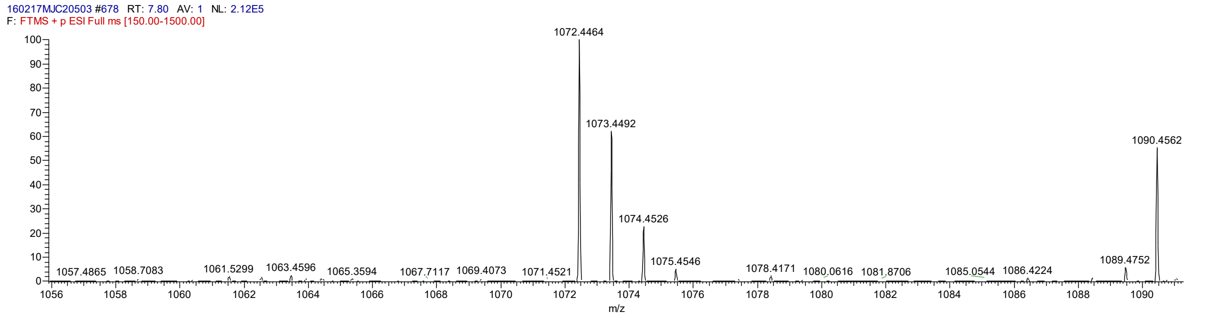

F:\160217MJC20503 02/17/16 14:31:50 MJC 205

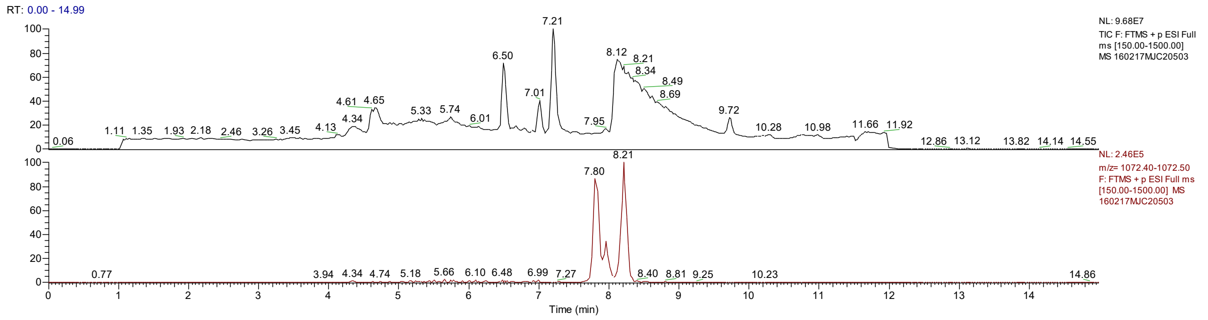

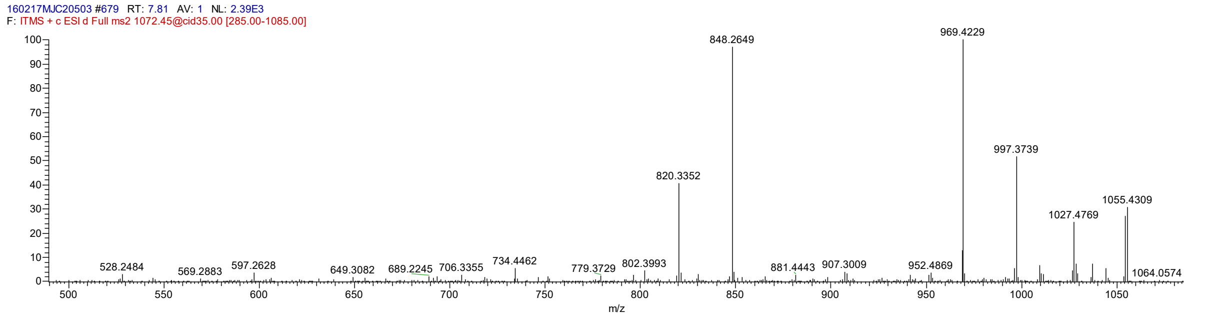

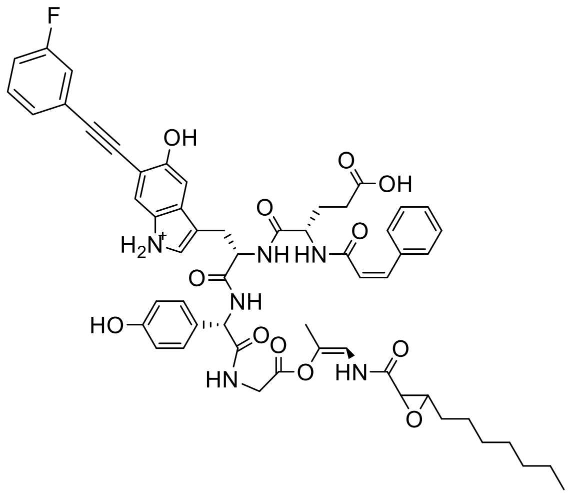

Exact Mass: 1027.4248

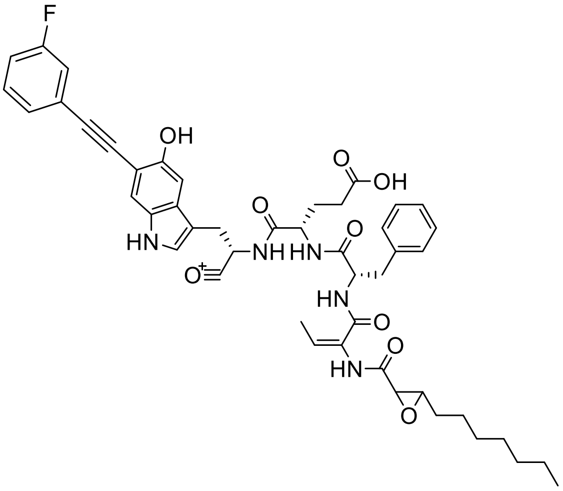

Exact Mass: 848.3665

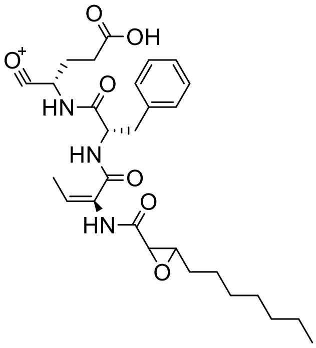

Exact Mass: 528.2704

Cross Coupled Linear Cystargamide (65)

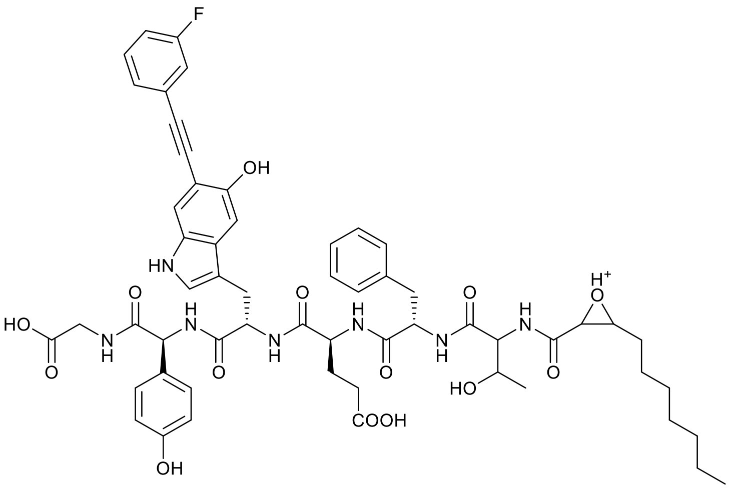

F:\160217MJC20503 02/17/16 14:31:50 MJC 205

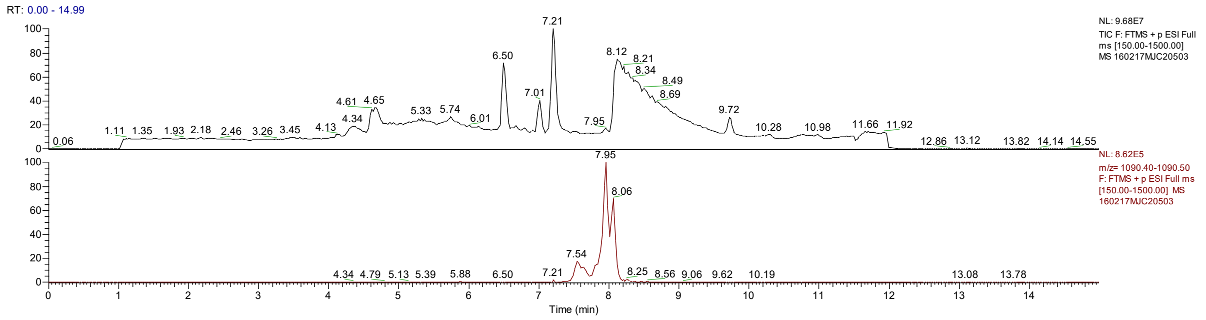

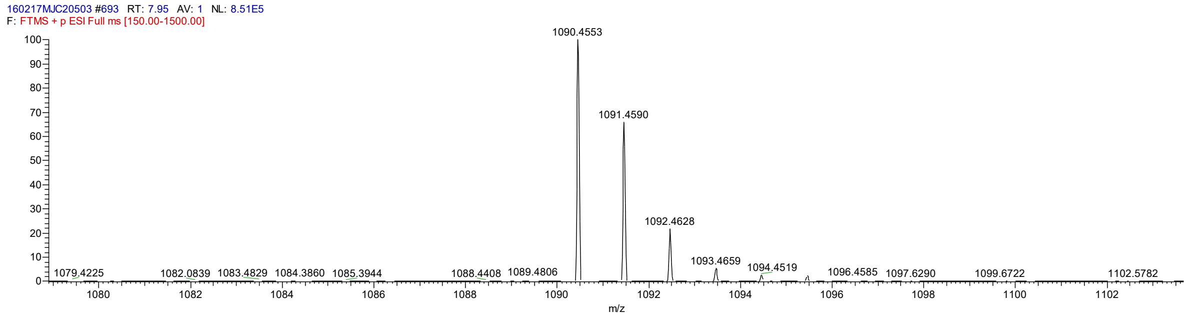

F:\160217MJC20503 02/17/16 14:31:50 MJC 205

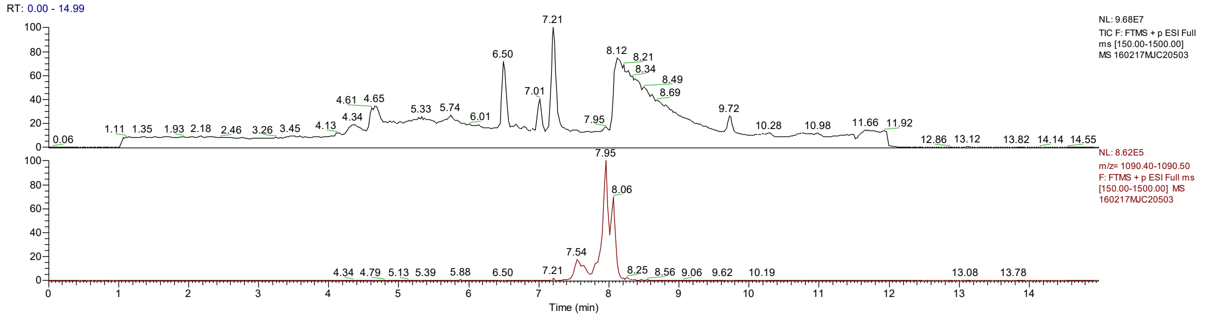

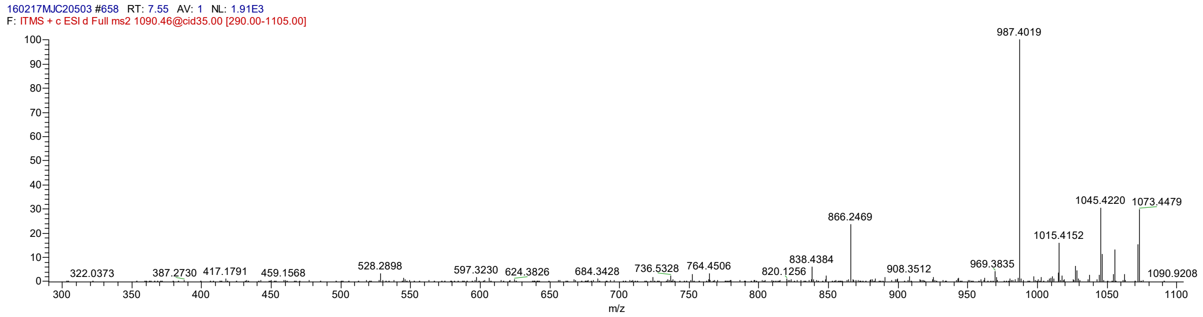

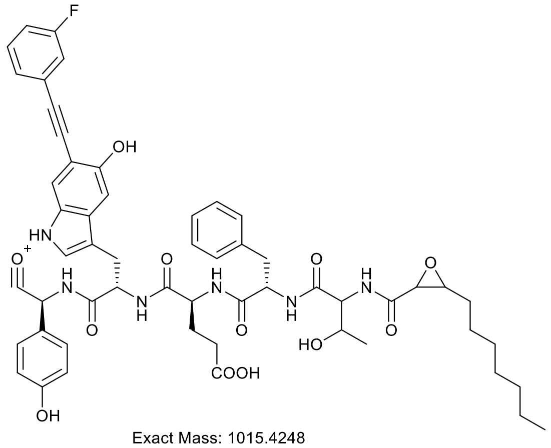

Exact Mass: 1015.4248

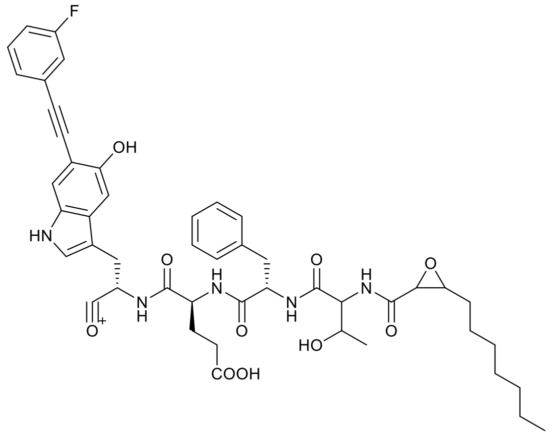

Exact Mass: 866.3771

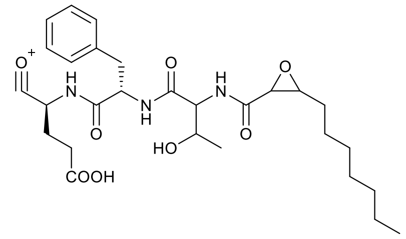

Exact Mass: 546.2810

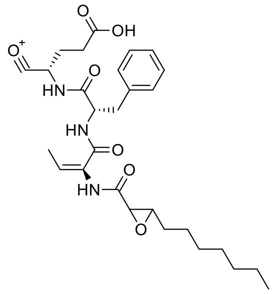

Exact Mass: 528.2704

UPLC chromatograms of purified cystargamide (top/black) and the best purified fraction of brominated cystargamide (bottom/blue) used for cross coupling experiment.

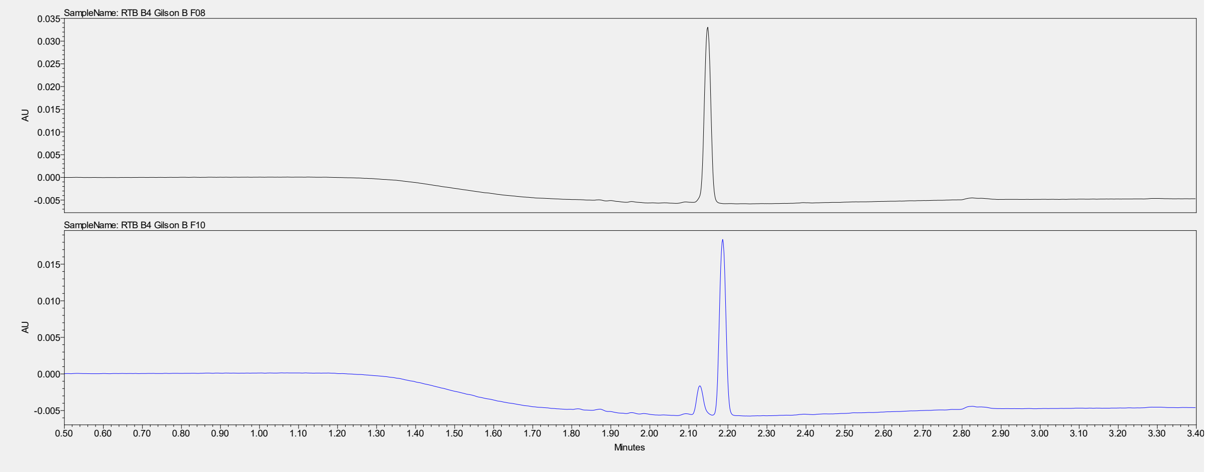

	Name	Retention Time	Area	% Area
1	Cystargamide (top chromatogram)	2.128	4619	14.53
2	Brominated cystargamide derivatives (bottom chromatogram)	2.187	27181	85.47

1. D. R. M. Smith, T. Willemse, D. S. Gkotsi, W. Schepens, B. U. W. Maes, S. Ballet and R. J. M. Goss, *Org. Lett*. 2014, **16**, 2622-2625.
2. K. W. Anderson and S. L. Buchwald, *Angew. Chem. Int. Ed.* 2005, **44**, 6173-6177.
3. K. A. Gill, F. Berrué, J. C. Arens and R. G. Kerr, *J. Nat. Prod.*, 2014, **77**, 1372-1376.

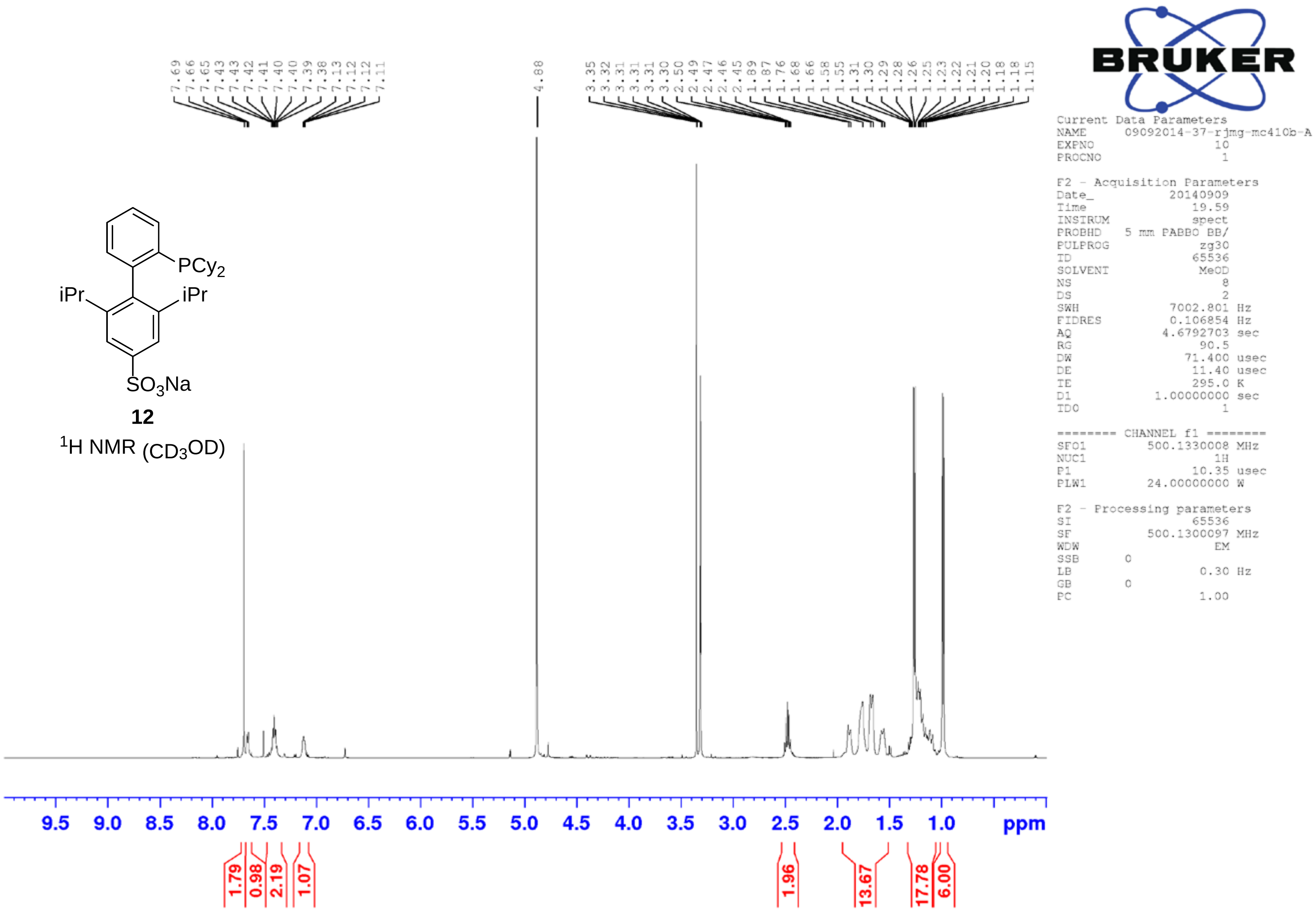

12

^{1}H NMR (CD_3OD)

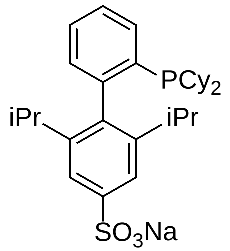

12

^{13}C NMR (CD_3OD)

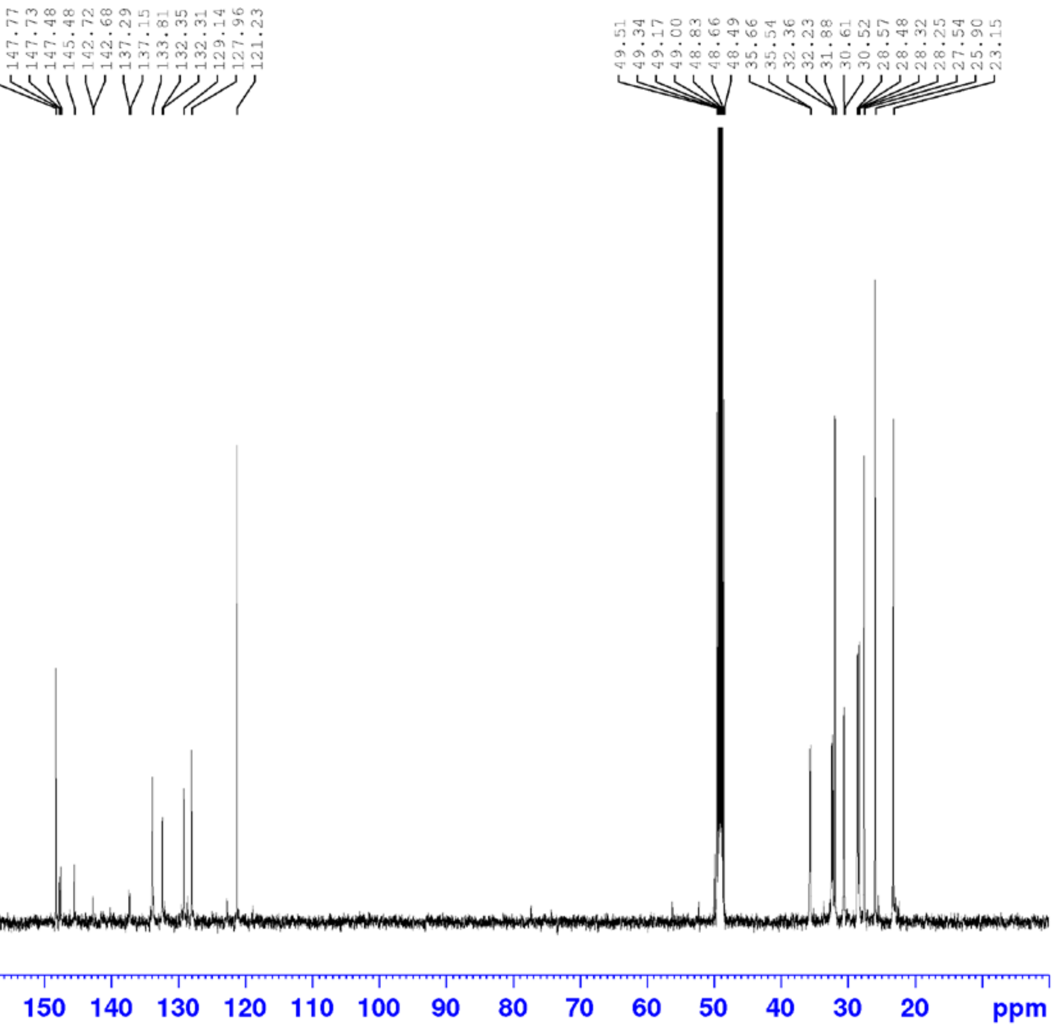

BRUKER

```
Current Data Parameters
NAME      09092014-37-rjmg-mc410b-A
EXPNO                    11
PROCNO                    1

F2 - Acquisition Parameters
Date_              20140909
Time                  20.54
INSTRUM               spect
PROBHD     5 mm PABBO BB/
PULPROG               udeft
TD                    21424
SOLVENT                MeOD
NS                      800
DS                        0
SWH               29761.904 Hz
FIDRES             1.389185 Hz
AQ                0.3599232 sec
RG                      128
DW                   16.800 usec
DE                     7.96 usec
TE                    295.0 K
D1               3.00000000 sec
D11              0.03000000 sec
D12              0.00002000 sec
D20            200.00000000 sec
TD0                       1

======== CHANNEL f1 ========
SFO1            125.7716224 MHz
NUC1                    13C
P1                     8.90 usec
P13                 2000.00 usec
P26                  500.00 usec
PLW1           125.00000000 W
SPNAM[5]       Crp60comp.4
SPOAL5                0.500
SPOFFS5   0 Hz
SPW5            15.12800026 W
SPNAM[8]  Crp60,0.5,20.1
SPOAL8                0.500
SPOFFS8   0 Hz
SPW8            15.12800026 W

======== CHANNEL f2 ========
SFO2            500.1320005 MHz
NUC2                     1H
CPDPRG[2            waltz65
PCPD2                 80.00 usec
PLW2            24.00000000 W
PLW12            0.39015001 W

F2 - Processing parameters
SI                   262144
SF              125.7576141 MHz
WDW                      EM
SSB       0
LB                     2.00 Hz
GB        0
PC                     1.40
```

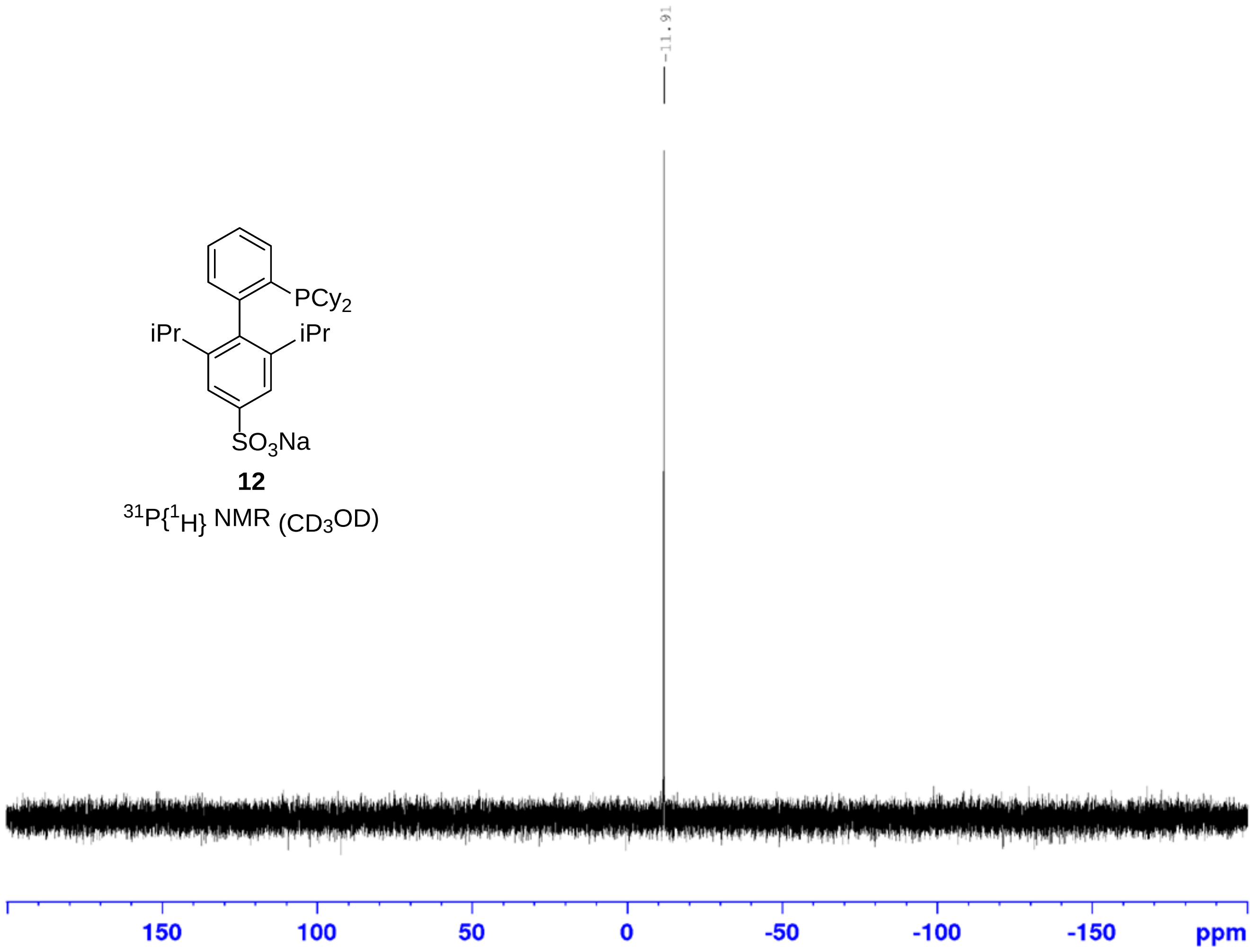

12

$^{31}P\{^{1}H\}$ NMR (CD_3OD)

Current Data Parameters

NAME	09052014-11-rjmg-mc410b-N
EXPNO	10
PROCNO	1

F2 - Acquisition Parameters

Date_	20140905
Time	9.41
INSTRUM	AV400
PROBHD	5 mm PABBO BB-
PULPROG	zgpg30
TD	65536
SOLVENT	MeOD
NS	128
DS	4
SWH	64935.066 Hz
FIDRES	0.990830 Hz
AQ	0.5046272 sec
RG	2048
DW	7.700 usec
DE	6.50 usec
TE	297.7 K
D1	1.50000000 sec
D11	0.03000000 sec
TD0	1

======== CHANNEL f1 ========

NUC1	31P
P1	15.00 usec
PL1	3.74 dB
PL1W	11.00252819 W
SFO1	162.0444100 MHz

======== CHANNEL f2 ========

CPDPRG[2	waltz65
NUC2	1H
PCPD2	80.00 usec
PL2	-3.00 dB
PL12	14.00 dB
PL13	14.00 dB
PL2W	19.24057770 W
PL12W	0.38389999 W
PL13W	0.38389999 W
SFO2	400.3020015 MHz

F2 - Processing parameters

SI	65536
SF	162.0444100 MHz
WDW	EM
SSB	0
LB	1.00 Hz
GB	0
PC	1.40

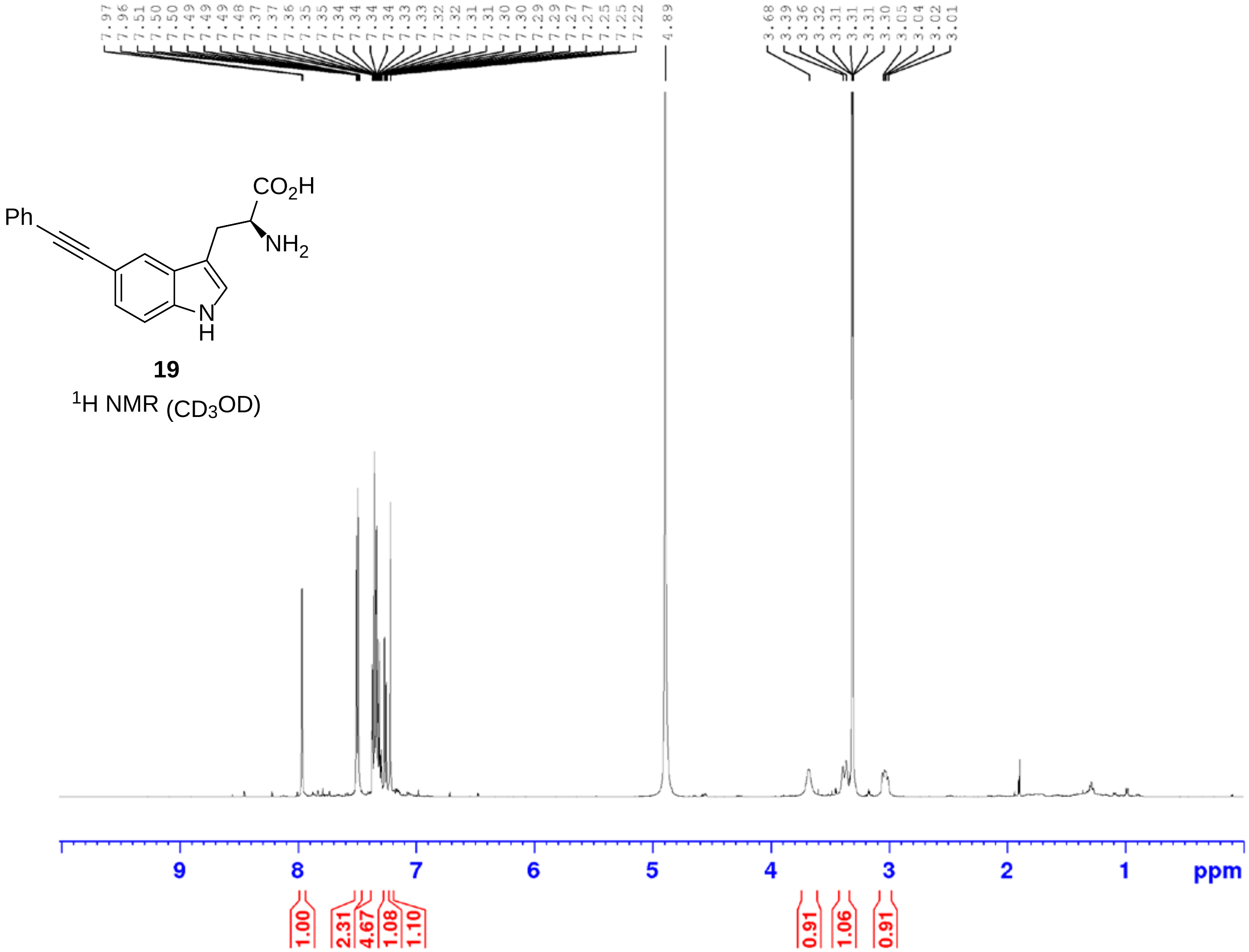

19

^{1}H NMR (CD_3OD)


```
Current Data Parameters
NAME        02172016-7-rjmg-mc410-A
EXPNO                            10
PROCNO                            1

F2 - Acquisition Parameters
Date_                      20160217
Time                          23.47
INSTRUM                       spect
PROBHD      5 mm CPPBBO BB
PULPROG                        zg30
TD                            65536
SOLVENT                        MeOD
NS                               64
DS                                2
SWH                        7002.801 Hz
FIDRES                     0.106854 Hz
AQ                        4.6792703 sec
RG                               64
DW                           71.400 usec
DE                            10.95 usec
TE                            295.0 K
D1                       1.00000000 sec
TD0                               1

======== CHANNEL f1 ========
SFO1                    500.1330008 MHz
NUC1                             1H
P1                            12.50 usec
PLW1                    14.00000000 W

F2 - Processing parameters
SI                            65536
SF                      500.1300097 MHz
WDW                              EM
SSB         0
LB                             0.30 Hz
GB          0
PC                             1.00
```

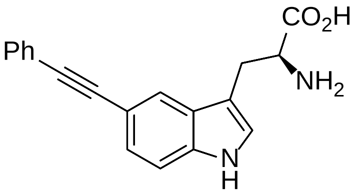

19

^{13}C NMR (CD_3OD)

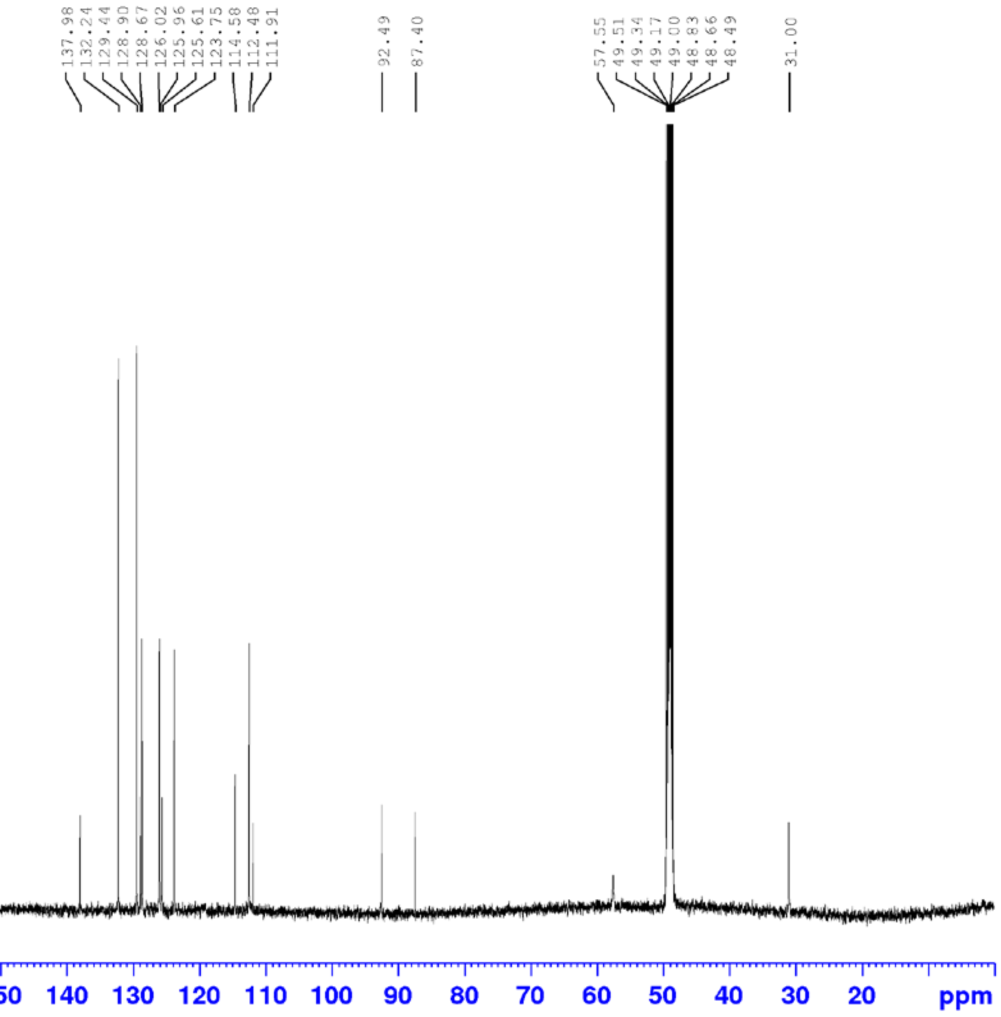

BRUKER

```
Current Data Parameters
NAME       02172016-7-rjmg-mc410-A
EXPNO                 11
PROCNO                 1

F2 - Acquisition Parameters
Date_           20160218
Time                1.06
INSTRUM            spect
PROBHD    5 mm CPPBBO BB
PULPROG            udeft
TD                 21424
SOLVENT             MeOD
NS                  1200
DS                     0
SWH            29761.904 Hz
FIDRES          1.389185 Hz
AQ             0.3599232 sec
RG                   128
DW                16.800 usec
DE                 19.22 usec
TE                 295.0 K
D1            3.00000000 sec
D11           0.03000000 sec
D12           0.00002000 sec
D20         200.00000000 sec
TD0                    1

======== CHANNEL f1 ========
SFO1         125.7716219 MHz
NUC1                 13C
P1                 11.00 usec
P13              2000.00 usec
P26               500.00 usec
PLW1         63.00000000 W
SPNAM[5]    Crp60comp.4
SPOAL5             0.500
SPOFFS5   0 Hz
SPW5         11.64700031 W
SPNAM[8] Crp60,0.5,20.1
SPOAL8             0.500
SPOFFS8   0 Hz
SPW8         11.64700031 W

======== CHANNEL f2 ========
SFO2         500.1320005 MHz
NUC2                  1H
CPDPRG[2         waltz65
PCPD2              80.00 usec
PLW2         14.00000000 W
PLW12         0.31500000 W

F2 - Processing parameters
SI                262144
SF          125.7576124 MHz
WDW                   EM
SSB       0
LB                  2.00 Hz
GB        0
PC                  1.40
```

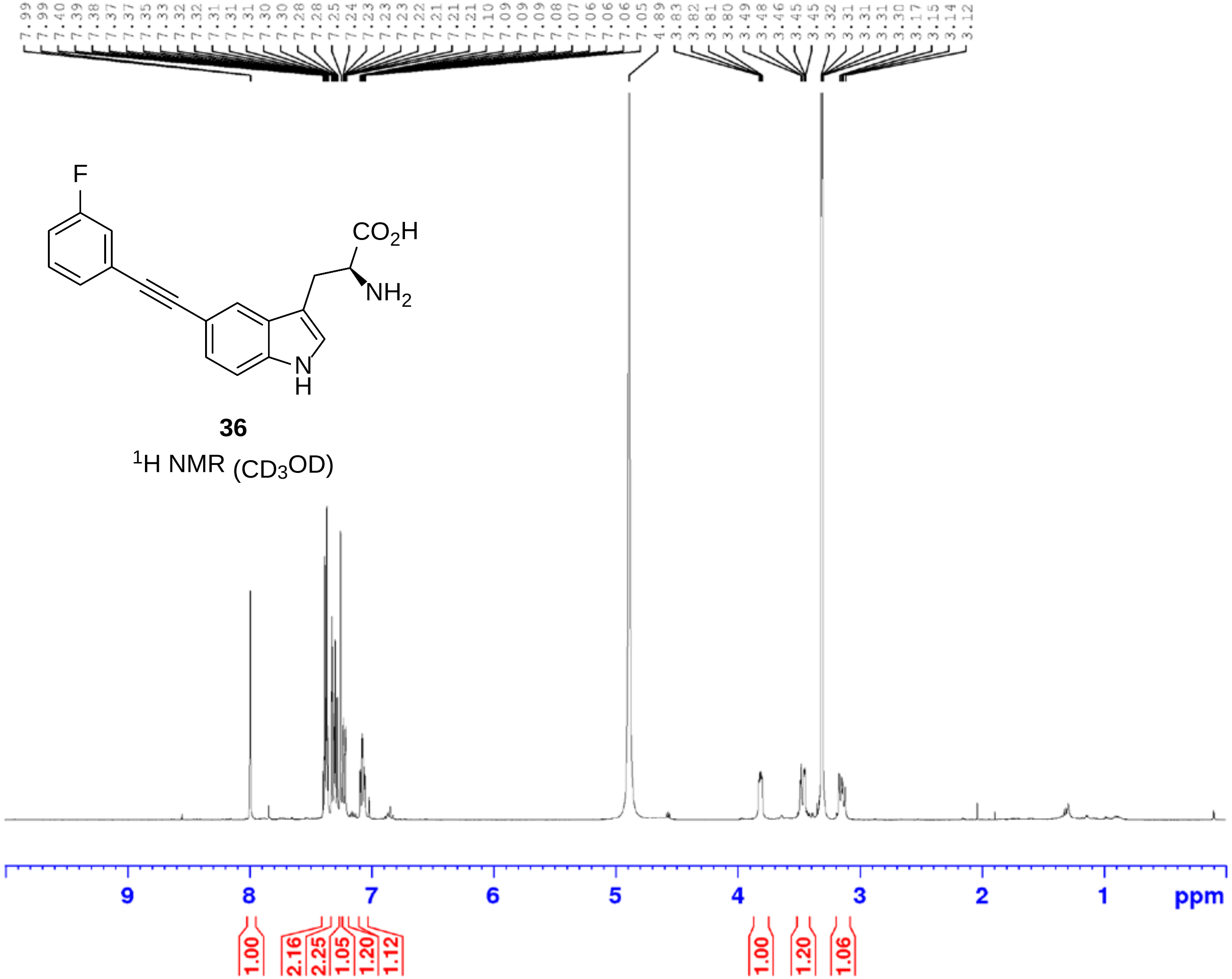

36

^{1}H NMR (CD_3OD)

Current Data Parameters
NAME 03112016-6-rjmg-mc410-A
EXPNO 10
PROCNO 1

F2 - Acquisition Parameters
Date_ 20160312
Time 1.17
INSTRUM spect
PROBHD 5 mm CPPBBO BB
PULPROG zg30
TD 65536
SOLVENT MeOD
NS 64
DS 2
SWH 7002.801 Hz
FIDRES 0.106854 Hz
AQ 4.6792703 sec
RG 64
DW 71.400 usec
DE 10.95 usec
TE 295.0 K
D1 1.00000000 sec
TD0 1

======== CHANNEL f1 ========
SFO1 500.1330008 MHz
NUC1 1H
P1 12.50 usec
PLW1 14.00000000 W

F2 - Processing parameters
SI 65536
SF 500.1300097 MHz
WDW EM
SSB 0
LB 0.30 Hz
GB 0
PC 1.00

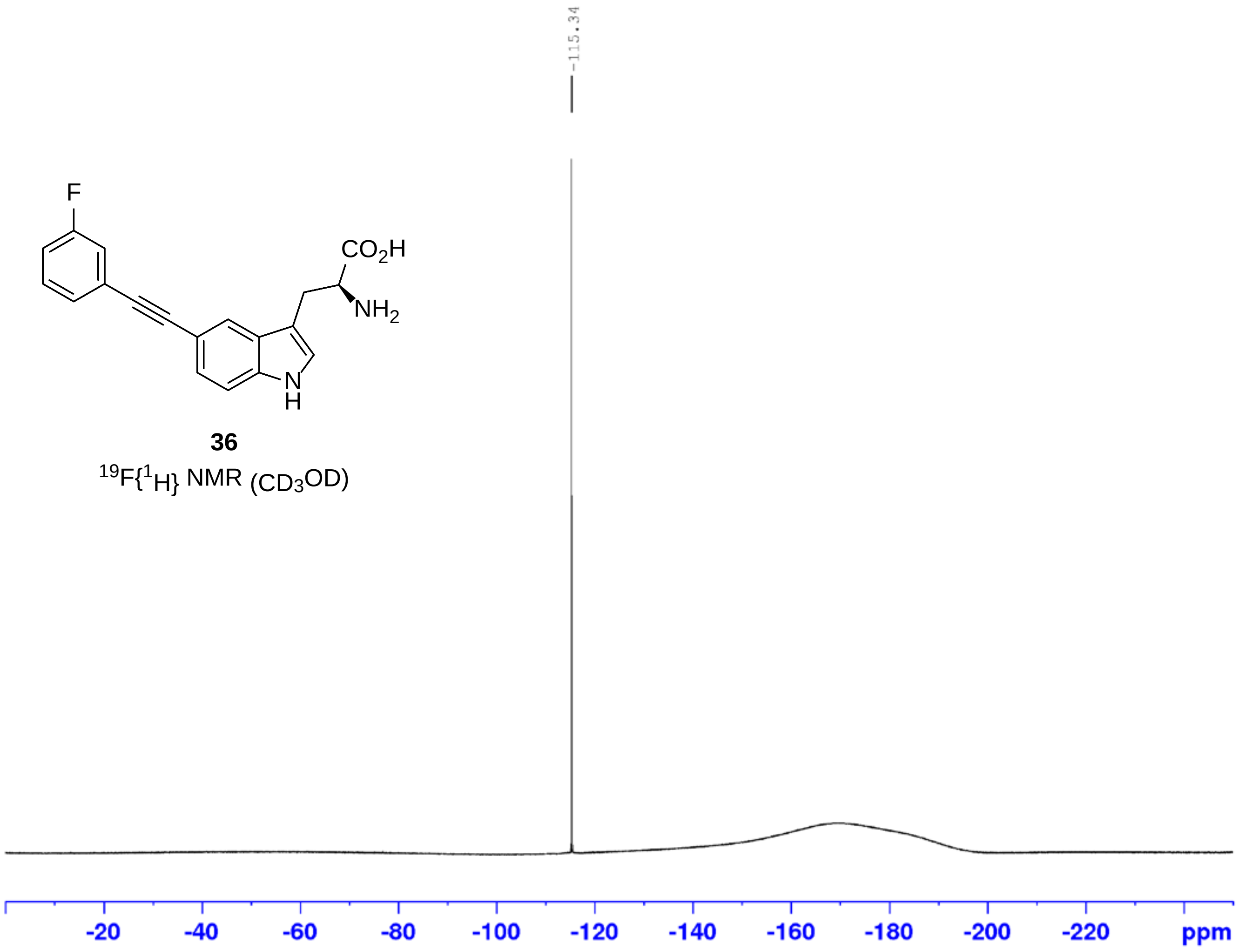

36

$^{19}F\{^{1}H\}$ NMR (CD_3OD)

```
Current Data Parameters
NAME      01262016-1-rjmg-mc410-F
EXPNO                   11
PROCNO                   1

F2 - Acquisition Parameters
Date_             20160126
Time                 19.10
INSTRUM              spect
PROBHD    5 mm PABBO BB/
PULPROG   zgfhigqn.3.and
TD                  131072
SOLVENT               MeOD
NS                      64
DS                       4
SWH             125000.000 Hz
FIDRES            0.953674 Hz
AQ               0.5242880 sec
RG                  121.22
DW                   4.000 usec
DE                    6.93 usec
TE                   295.0 K
D1              1.00000000 sec
D11             0.03000000 sec
D12             0.00002000 sec
TD0                      1

======== CHANNEL f1 ========
SFO1           470.3477405 MHz
NUC1                   19F
P1                   14.60 usec
PLW1           48.00000000 W

======== CHANNEL f2 ========
SFO2           499.9319997 MHz
NUC2                    1H
CPDPRG[2           waltz65
PCPD2                80.00 usec
PLW2           16.00000000 W
PLW12           0.27563000 W

F2 - Processing parameters
SI                   65536
SF             470.4041892 MHz
WDW                     EM
SSB       0
LB                    0.30 Hz
GB        0
PC                    1.00
```

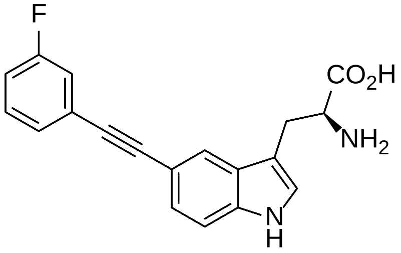

36

^{19}F NMR (CD_3OD)

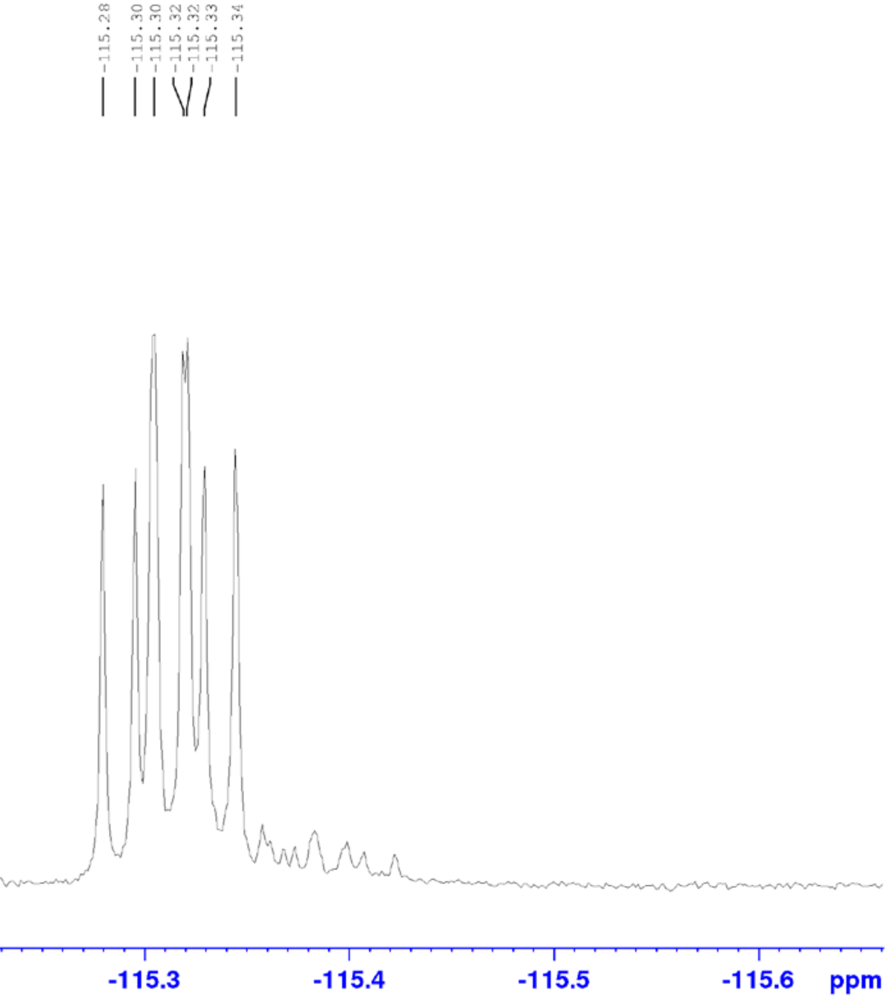


```
Current Data Parameters
NAME     03162016-7-rjmg-mc410-M
EXPNO                  10
PROCNO                  1

F2 - Acquisition Parameters
Date_            20160316
Time                11.04
INSTRUM           AVII400
PROBHD    5 mm PABBO BB/
PULPROG           zgflqn
TD                 65536
SOLVENT             MeOD
NS                    64
DS                     4
SWH            30000.000 Hz
FIDRES          0.457764 Hz
AQ             1.0922667 sec
RG                   128
DW                16.667 usec
DE                  8.37 usec
TE                 294.8 K
D1            1.00000000 sec
TD0                    1

======== CHANNEL f1 ========
SFO1         376.4569514 MHz
NUC1                 19F
P1                 15.00 usec
PLW1         17.00000000 W

F2 - Processing parameters
SI                 65536
SF           376.4983662 MHz
WDW                   EM
SSB      0
LB                  0.30 Hz
GB       0
PC                  1.00
```

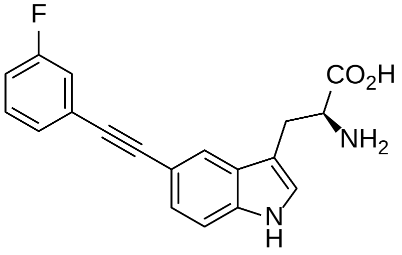

36

^{13}C NMR (CD_3OD)

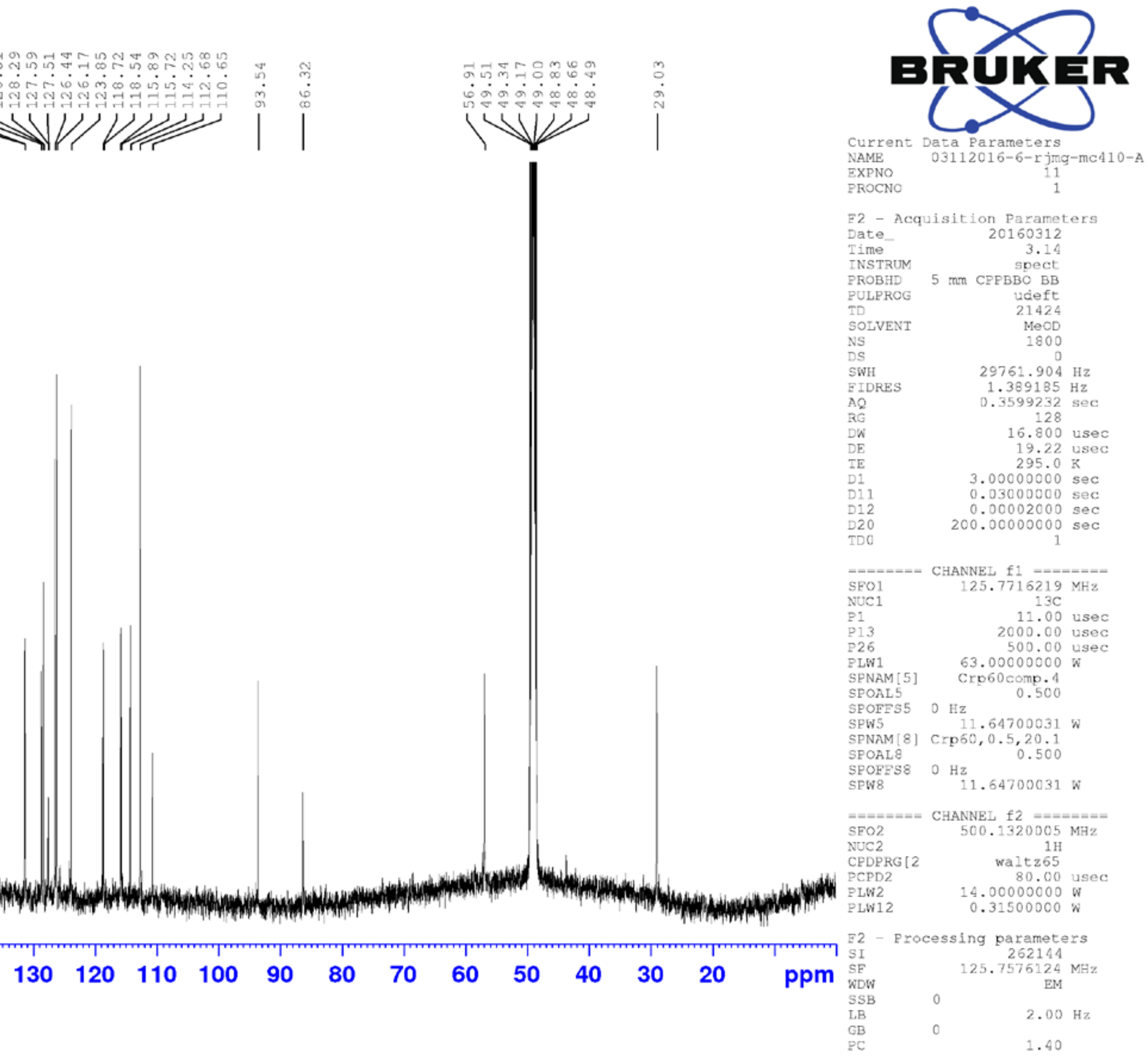

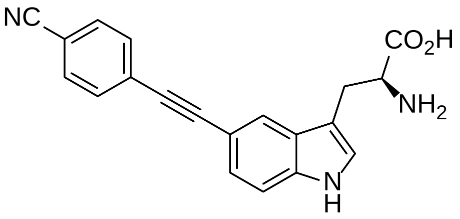

39

^1H NMR (CD_3OD)

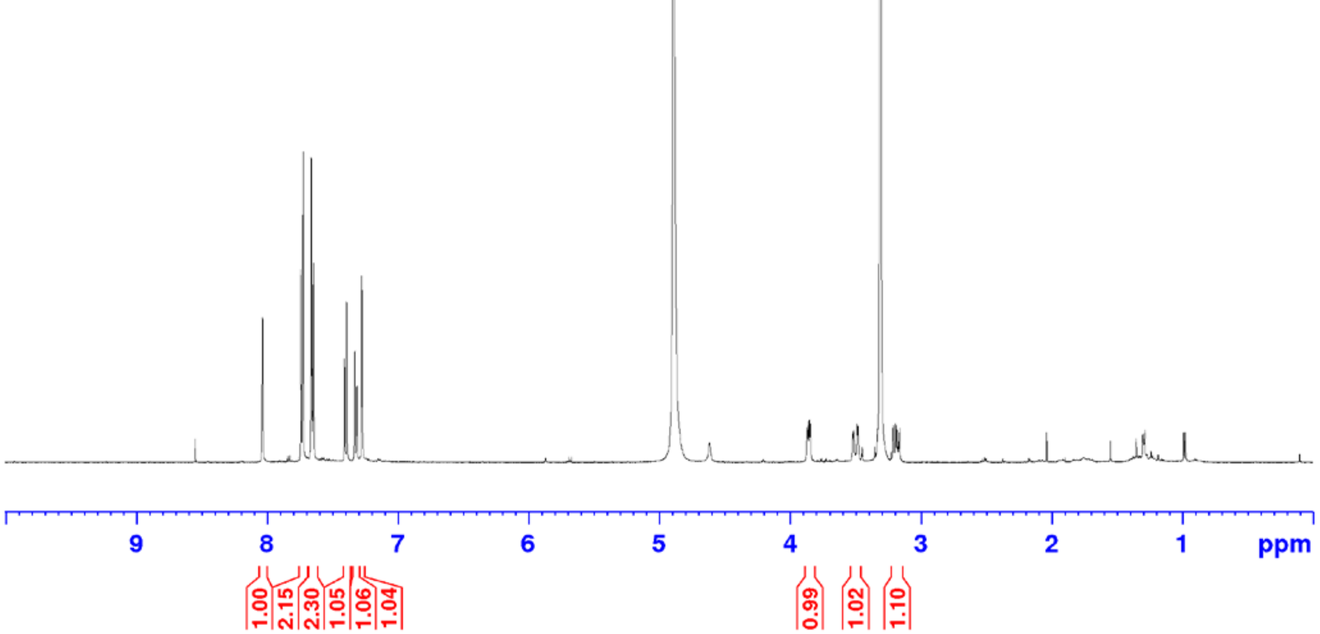


```
Current Data Parameters
NAME       03142016-29-rjmg-mc410-A
EXPNO                    10
PROCNO                    1

F2 - Acquisition Parameters
Date_              20160314
Time                  19.09
INSTRUM               spect
PROBHD    5 mm CPPBBO BB
PULPROG                zg30
TD                    65536
SOLVENT                MeOD
NS                       64
DS                        2
SWH                7002.801 Hz
FIDRES             0.106854 Hz
AQ                4.6792703 sec
RG                       57
DW                   71.400 usec
DE                    10.95 usec
TE                    295.0 K
D1               1.00000000 sec
TD0                       1

======== CHANNEL f1 ========
SFO1            500.1330008 MHz
NUC1                     1H
P1                    12.50 usec
PLW1            14.00000000 W

F2 - Processing parameters
SI                    65536
SF              500.1300097 MHz
WDW                      EM
SSB      0
LB                     0.30 Hz
GB       0
PC                     1.00
```

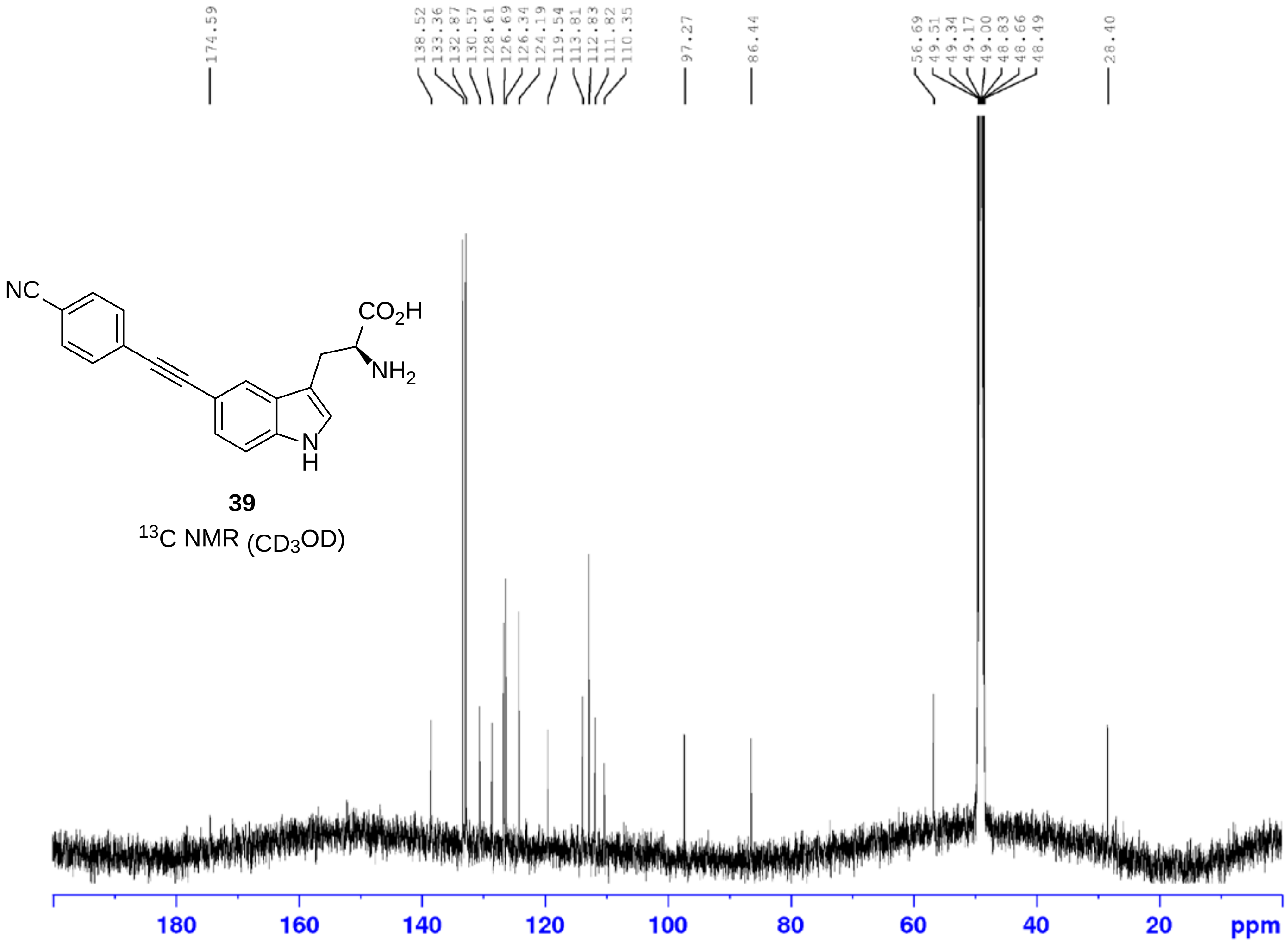

39

^{13}C NMR (CD_3OD)


```
Current Data Parameters
NAME       03142016-29-rjmg-mc410-A
EXPNO                    11
PROCNO                    1

F2 - Acquisition Parameters
Date_              20160314
Time                  20.53
INSTRUM               spect
PROBHD     5 mm CPPBBO BB
PULPROG               udeft
TD                    21424
SOLVENT                MeOD
NS                     1600
DS                        0
SWH               29761.904 Hz
FIDRES             1.389185 Hz
AQ                0.3599232 sec
RG                      128
DW                   16.800 usec
DE                    19.22 usec
TE                    295.0 K
D1               3.00000000 sec
D11              0.03000000 sec
D12              0.00002000 sec
D20            200.00000000 sec
TD0                       1

======== CHANNEL f1 ========
SFO1         125.7716219 MHz
NUC1                 13C
P1                 11.00 usec
P13              2000.00 usec
P26               500.00 usec
PLW1         63.00000000 W
SPNAM[5]    Crp60comp.4
SPOAL5             0.500
SPOFFS5    0 Hz
SPW5         11.64700031 W
SPNAM[8]  Crp60,0.5,20.1
SPOAL8             0.500
SPOFFS8    0 Hz
SPW8         11.64700031 W

======== CHANNEL f2 ========
SFO2         500.1320005 MHz
NUC2                  1H
CPDPRG[2          waltz65
PCPD2              80.00 usec
PLW2         14.00000000 W
PLW12         0.31500000 W

F2 - Processing parameters
SI                262144
SF           125.7576120 MHz
WDW                   EM
SSB        0
LB                  2.00 Hz
GB         0
PC                  1.40
```

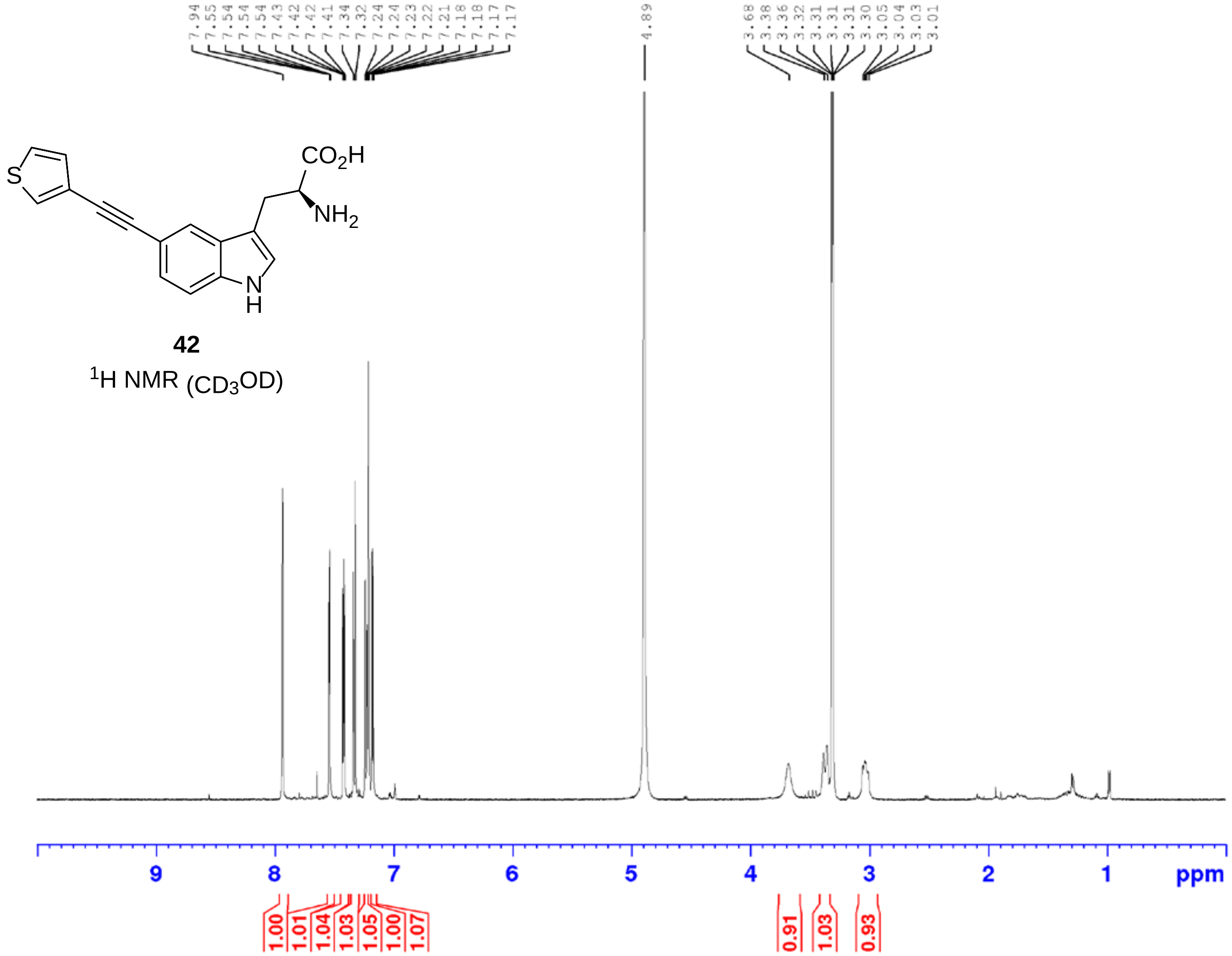

42

^{1}H NMR (CD_3OD)

Current Data Parameters
NAME 03172016-32-rjmg-mc410-F
EXPNO 10
PROCNO 1

F2 - Acquisition Parameters
Date_ 20160317
Time 19.05
INSTRUM spect
PROBHD 5 mm PABBO BB/
PULPROG zg30
TD 65536
SOLVENT MeOD
NS 8
DS 2
SWH 6996.269 Hz
FIDRES 0.106755 Hz
AQ 4.6836395 sec
RG 121.22
DW 71.467 usec
DE 11.38 usec
TE 295.0 K
D1 1.00000000 sec
TD0 1

======== CHANNEL f1 ========
SFO1 499.9329996 MHz
NUC1 1H
P1 10.60 usec
PLW1 16.00000000 W

F2 - Processing parameters
SI 65536
SF 499.9300095 MHz
WDW EM
SSB 0
LB 0.30 Hz
GB 0
PC 1.00

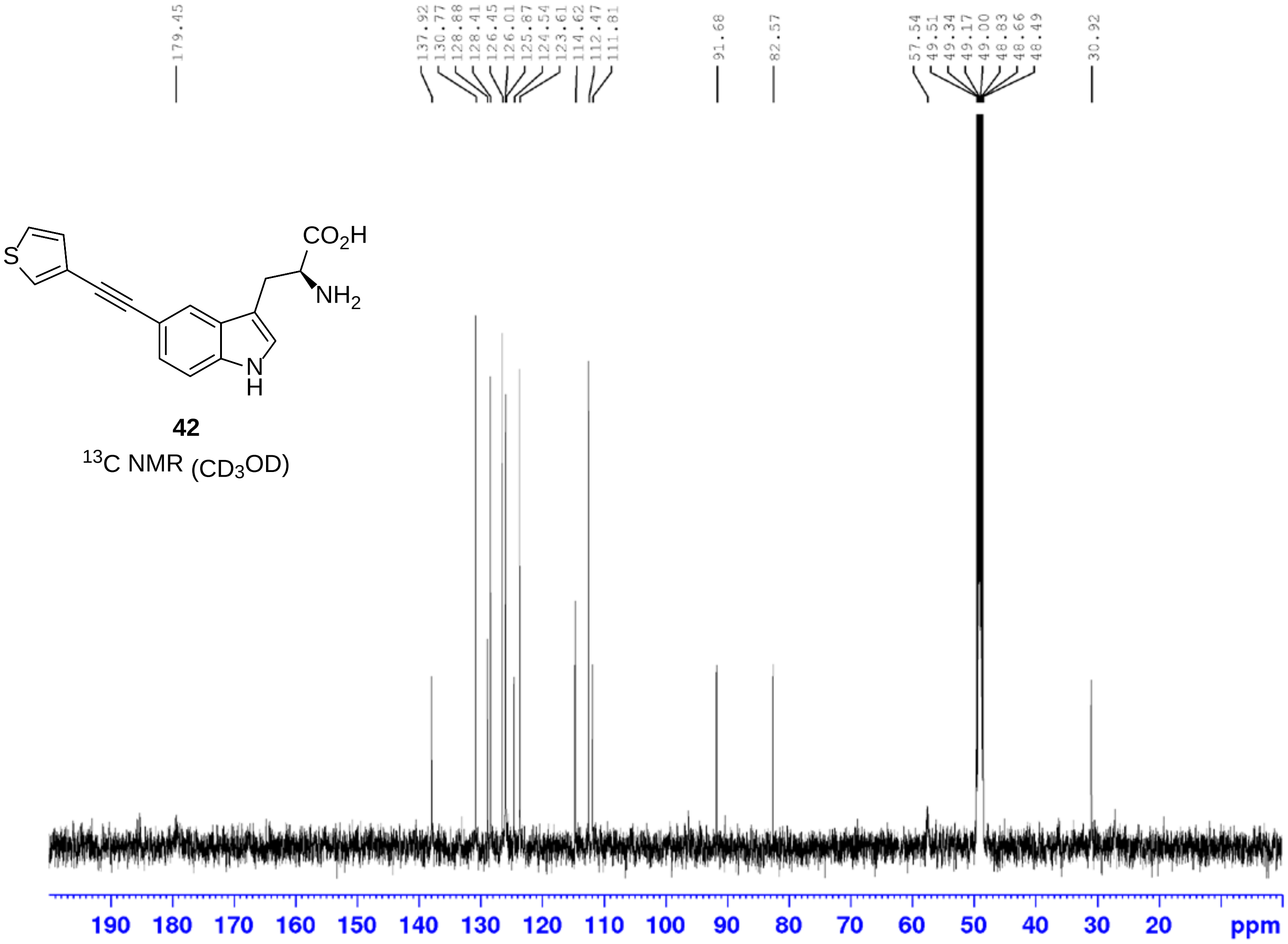

42

^{13}C NMR (CD_3OD)

Current Data Parameters
NAME 03172016-32-rjmg-mc410-F
EXPNO 11
PROCNO 1

F2 - Acquisition Parameters
Date_ 20160317
Time 20.49
INSTRUM spect
PROBHD 5 mm PABBO BB/
PULPROG udeft
TD 21424
SOLVENT MeOD
NS 1600
DS 0
SWH 29761.904 Hz
FIDRES 1.389185 Hz
AQ 0.3599232 sec
RG 121.22
DW 16.800 usec
DE 9.36 usec
TE 295.0 K
D1 3.00000000 sec
D11 0.03000000 sec
D12 0.00002000 sec
D20 200.00000000 sec
TD0 1

======== CHANNEL f1 ========
SFO1 125.7213263 MHz
NUC1 13C
P1 10.00 usec
P13 2000.00 usec
P26 500.00 usec
PLW1 85.00000000 W
SPNAM[5] Crp60comp.4
SPOAL5 0.500
SPOFFS5 0 Hz
SPW5 12.98700047 W
SPNAM[8] Crp60,0.5,20.1
SPOAL8 0.500
SPOFFS8 0 Hz
SPW8 12.98700047 W

======== CHANNEL f2 ========
SFO2 499.9319997 MHz
NUC2 1H
CPDPRG[2 waltz65
PCPD2 80.00 usec
PLW2 16.00000000 W
PLW12 0.27563000 W

F2 - Processing parameters
SI 262144
SF 125.7073222 MHz
WDW EM
SSB 0
LB 2.00 Hz
GB 0
PC 1.40

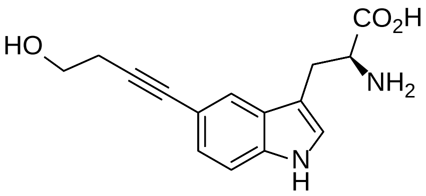

45

^1H NMR (CD_3OD)

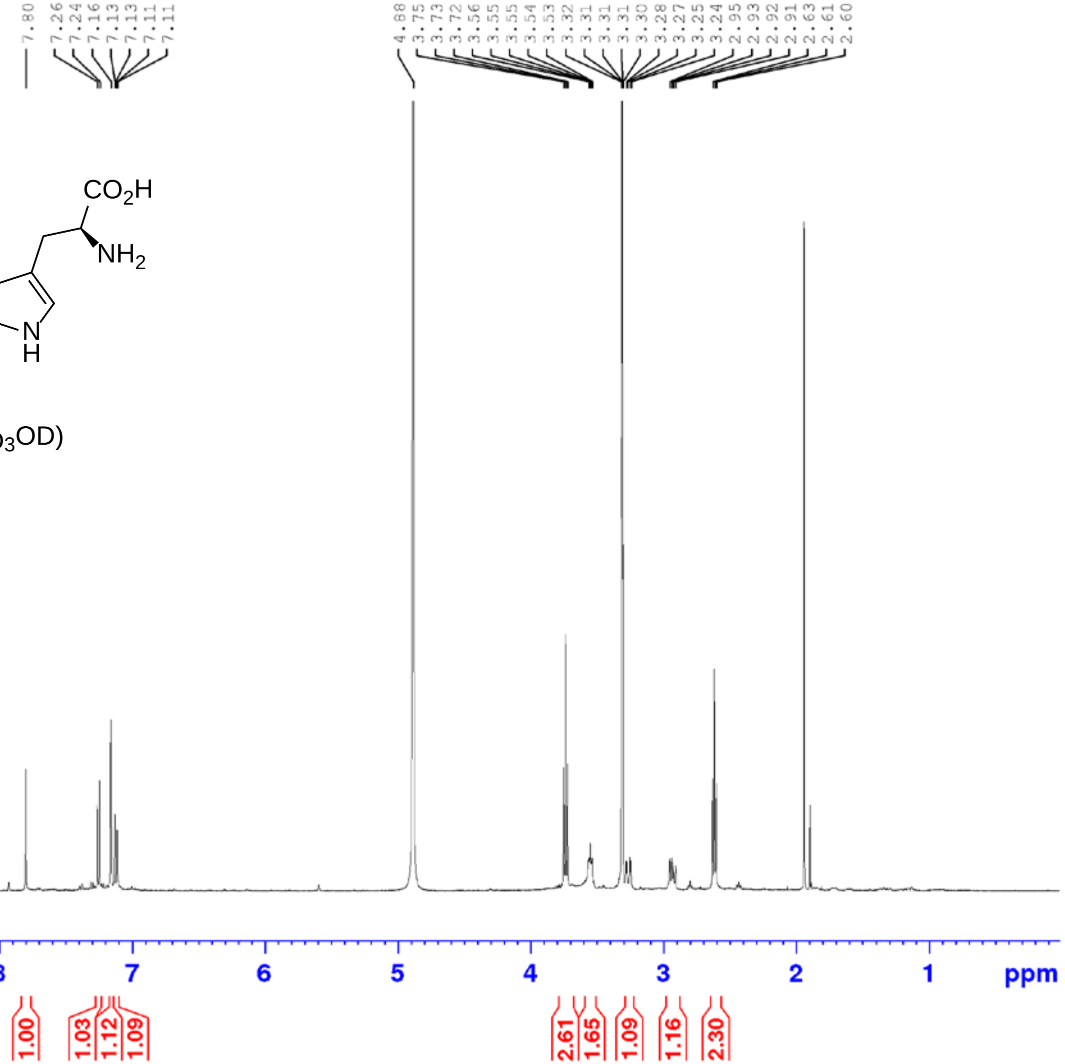


```
Current Data Parameters
NAME       02252015-18-rjmg-mc410-F
EXPNO                    10
PROCNO                    1

F2 - Acquisition Parameters
Date_              20150225
Time                  22.59
INSTRUM               spect
PROBHD     5 mm PABBO BB/
PULPROG                zg30
TD                    65536
SOLVENT                MeOD
NS                        8
DS                        2
SWH             6996.269 Hz
FIDRES          0.106755 Hz
AQ             4.6836395 sec
RG                   121.22
DW                   71.467 usec
DE                    11.38 usec
TE                    295.8 K
D1               1.00000000 sec
TD0                       1

======== CHANNEL f1 ========
SFO1          499.9329996 MHz
NUC1                     1H
P1                    10.60 usec
PLW1          16.00000000 W

F2 - Processing parameters
SI                    65536
SF            499.9300097 MHz
WDW                      EM
SSB        0
LB                     0.30 Hz
GB         0
PC                     1.00
```

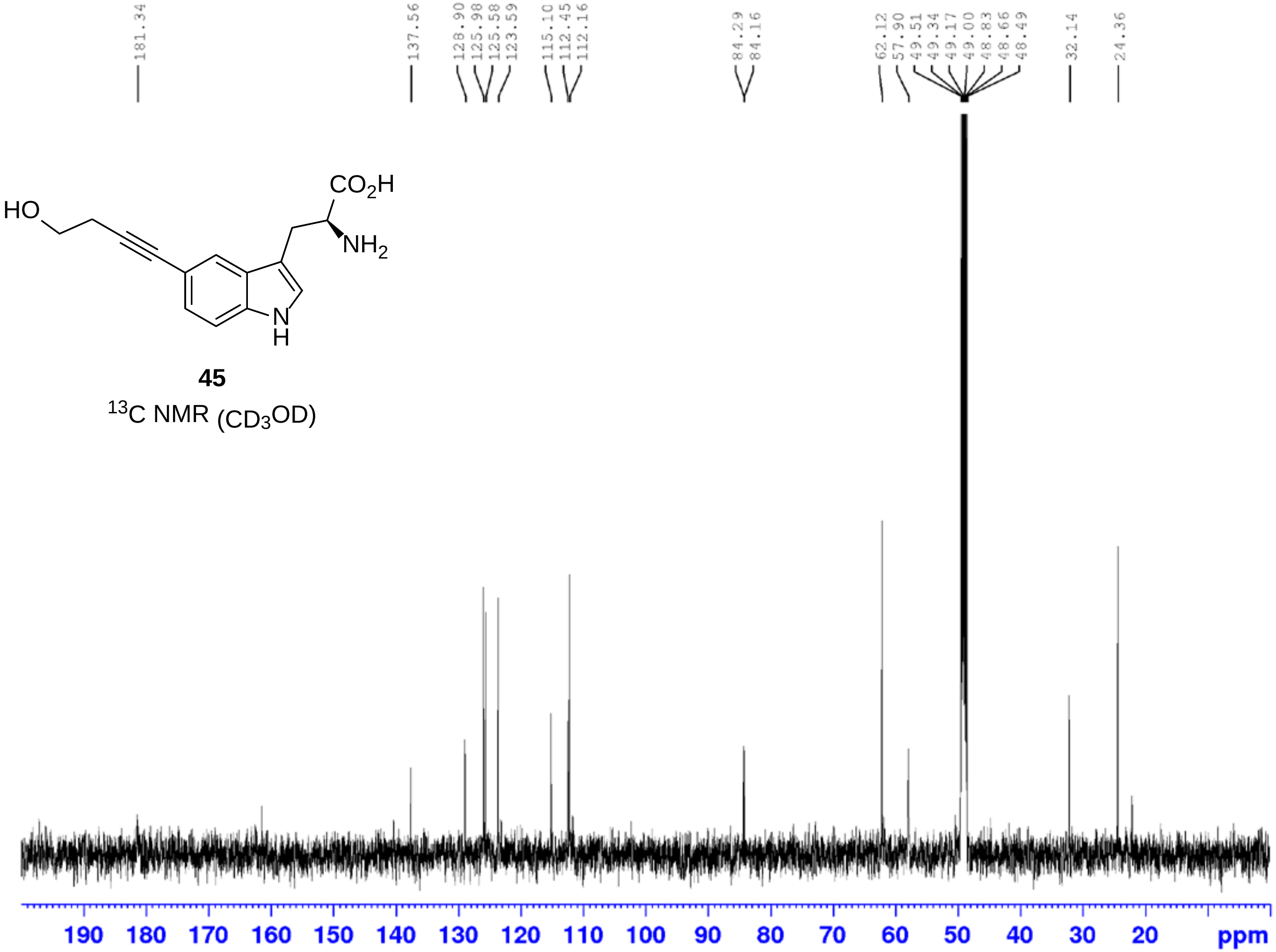

45

^{13}C NMR (CD_3OD)

BRUKER

```
Current Data Parameters
NAME      02252015-18-rjmg-mc410-F
EXPNO                         11
PROCNO                         1

F2 - Acquisition Parameters
Date_                   20150225
Time                       23.54
INSTRUM                    spect
PROBHD      5 mm PABBO BB/
PULPROG                    udeft
TD                         21424
SOLVENT                     MeOD
NS                           800
DS                             0
SWH                    29761.904 Hz
FIDRES                  1.389185 Hz
AQ                     0.3599232 sec
RG                        121.22
DW                        16.800 usec
DE                          9.36 usec
TE                         297.0 K
D1                    3.00000000 sec
D11                   0.03000000 sec
D12                   0.00002000 sec
D20                 200.00000000 sec
TD0                            1

======== CHANNEL f1 ========
SFO1                 125.7213263 MHz
NUC1                         13C
P1                         10.00 usec
P13                      2000.00 usec
P26                       500.00 usec
PLW1                 85.00000000 W
SPNAM[5]          Crp60comp.4
SPOAL5                     0.500
SPOFFS5     0 Hz
SPW5                 12.98700047 W
SPNAM[8]   Crp60,0.5,20.1
SPOAL8                     0.500
SPOFFS8     0 Hz
SPW8                 12.98700047 W

======== CHANNEL f2 ========
SFO2                 499.9319997 MHz
NUC2                          1H
CPDPRG[2                 waltz65
PCPD2                      80.00 usec
PLW2                 16.00000000 W
PLW12                 0.27563000 W

F2 - Processing parameters
SI                        262144
SF                   125.7073225 MHz
WDW                           EM
SSB         0
LB                          2.00 Hz
GB          0
PC                          1.40
```

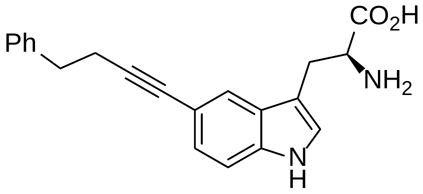

48

^{1}H NMR (CD_3OD)

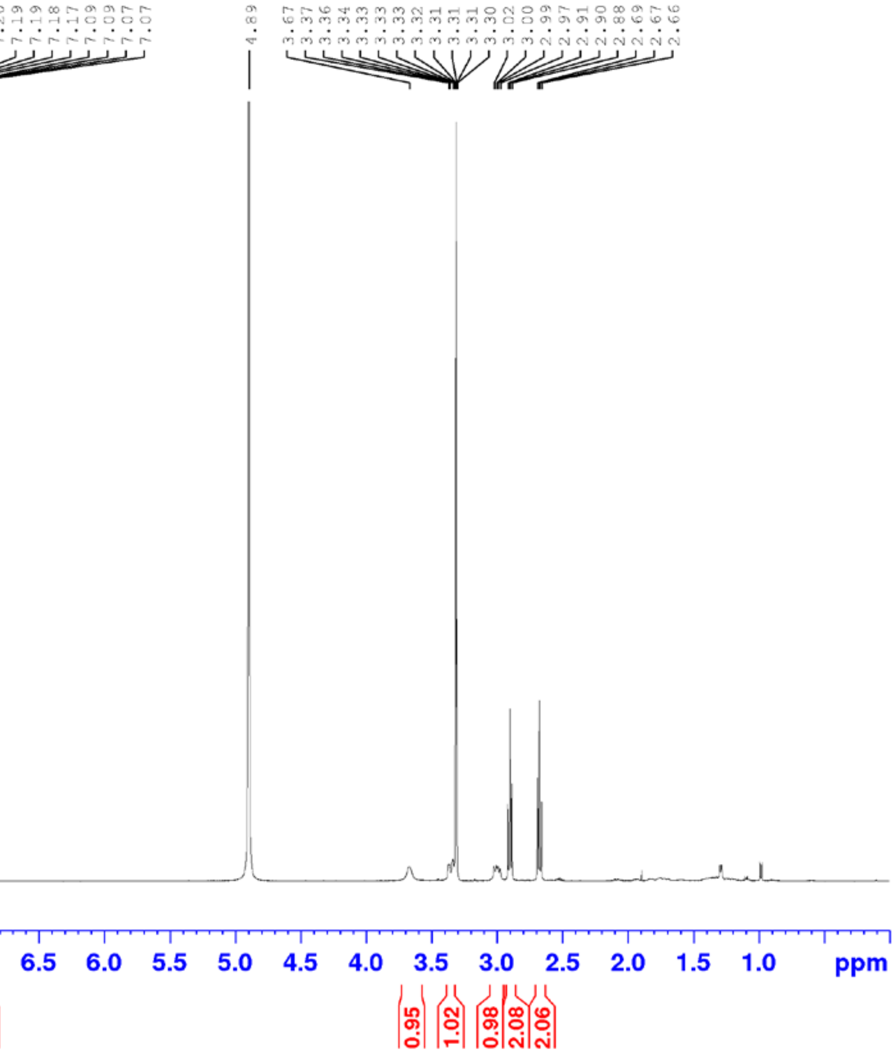

Current Data Parameters
NAME 01202016-5-rjmg-mc410-F
EXPNO 10
PROCNO 1

F2 - Acquisition Parameters
Date_ 20160120
Time 19.04
INSTRUM spect
PROBHD 5 mm PABBO BB/
PULPROG zg30
TD 65536
SOLVENT MeOD
NS 8
DS 2
SWH 6996.269 Hz
FIDRES 0.106755 Hz
AQ 4.6836395 sec
RG 121.22
DW 71.467 usec
DE 11.38 usec
TE 295.0 K
D1 1.00000000 sec
TD0 1

======== CHANNEL f1 ========
SFO1 499.9329996 MHz
NUC1 1H
P1 10.60 usec
PLW1 16.00000000 W

F2 - Processing parameters
SI 65536
SF 499.9300096 MHz
WDW EM
SSB 0
LB 0.30 Hz
GB 0
PC 1.00

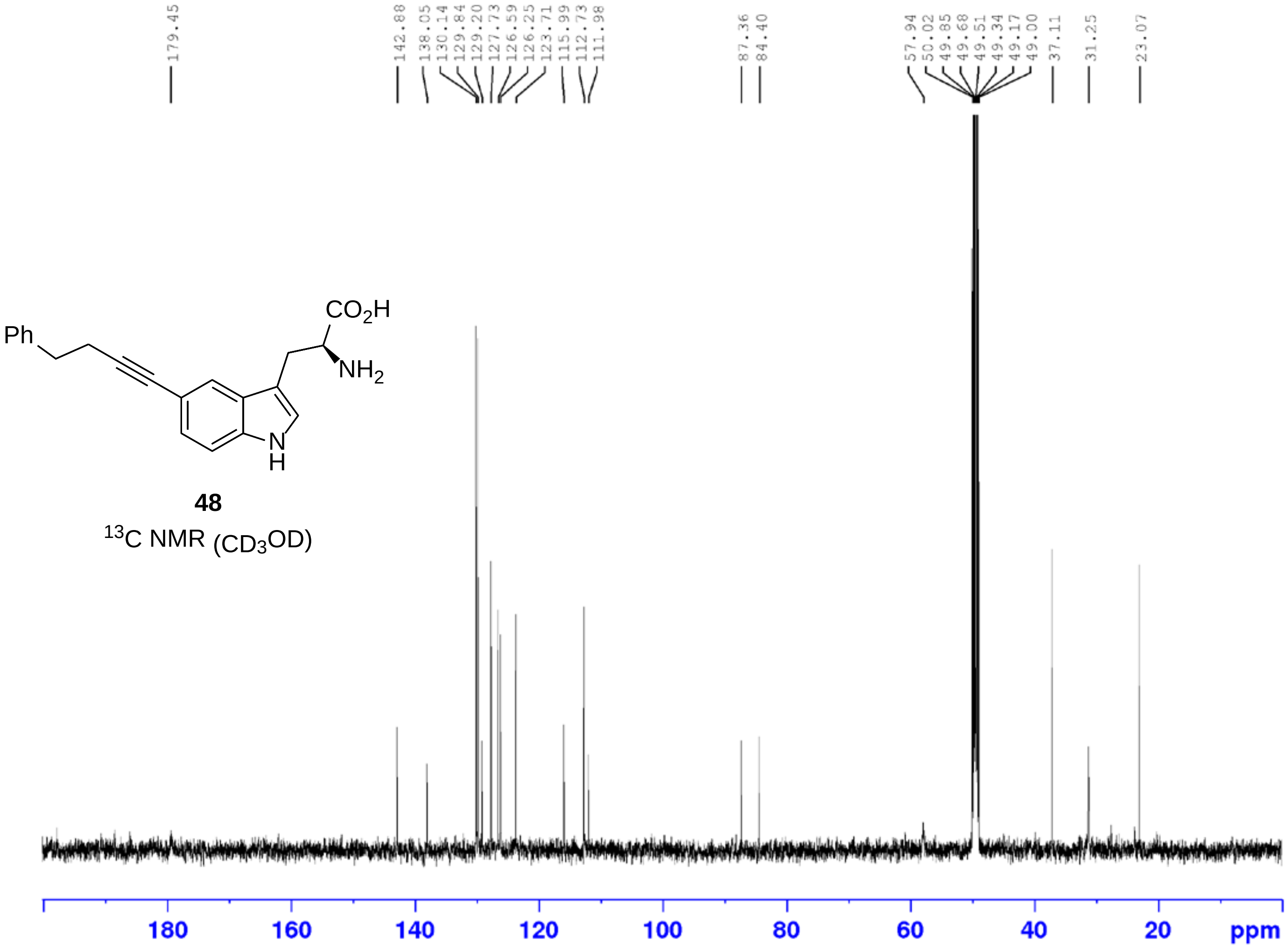

48

^{13}C NMR (CD_3OD)

BRUKER

Current Data Parameters
NAME 01202016-5-rjmg-mc410-F
EXPNO 11
PROCNO 1

F2 - Acquisition Parameters
Date_ 20160120
Time 19.59
INSTRUM spect
PROBHD 5 mm PABBO BB/
PULPROG udeft
TD 21424
SOLVENT MeOD
NS 800
DS 0
SWH 29761.904 Hz
FIDRES 1.389185 Hz
AQ 0.3599232 sec
RG 121.22
DW 16.800 usec
DE 9.36 usec
TE 295.0 K
D1 3.00000000 sec
D11 0.03000000 sec
D12 0.00002000 sec
D20 200.00000000 sec
TD0 1

======== CHANNEL f1 ========
SFO1 125.7213263 MHz
NUC1 13C
P1 10.00 usec
P13 2000.00 usec
P26 500.00 usec
PLW1 85.00000000 W
SPNAM[5] Crp60comp.4
SPOAL5 0.500
SPOFFS5 0 Hz
SPW5 12.98700047 W
SPNAM[8] Crp60,0.5,20.1
SPOAL8 0.500
SPOFFS8 0 Hz
SPW8 12.98700047 W

======== CHANNEL f2 ========
SFO2 499.9319997 MHz
NUC2 1H
CPDPRG[2 waltz65
PCPD2 80.00 usec
PLW2 16.00000000 W
PLW12 0.27563000 W

F2 - Processing parameters
SI 262144
SF 125.7072585 MHz
WDW EM
SSB 0
LB 2.00 Hz
GB 0
PC 1.40

51

^{1}H NMR (CD_3OD)

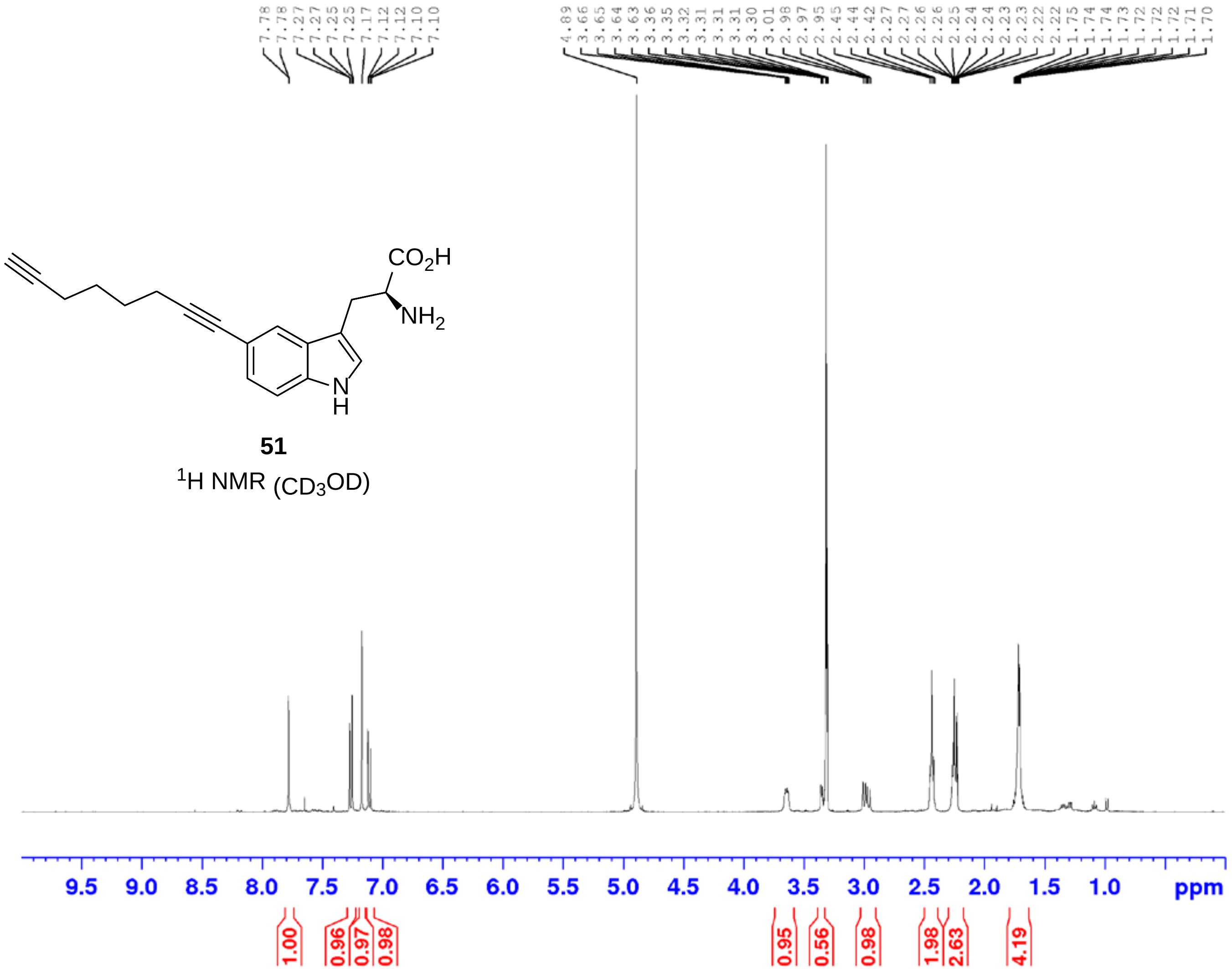

Current Data Parameters
NAME 07142015-11-rjmg-mc410-M
EXPNO 10
PROCNO 1

F2 - Acquisition Parameters
Date_ 20150714
Time 22.42
INSTRUM AVII400
PROBHD 5 mm PABBO BB/
PULPROG zg30
TD 65536
SOLVENT MeOD
NS 8
DS 2
SWH 5605.381 Hz
FIDRES 0.085531 Hz
AQ 5.8458114 sec
RG 128
DW 89.200 usec
DE 13.44 usec
TE 294.9 K
D1 1.00000000 sec
TD0 1

======== CHANNEL f1 ========
SFO1 400.1324008 MHz
NUC1 1H
P1 15.50 usec
PLW1 12.00000000 W

F2 - Processing parameters
SI 65536
SF 400.1300079 MHz
WDW EM
SSB 0
LB 0.30 Hz
GB 0
PC 1.00

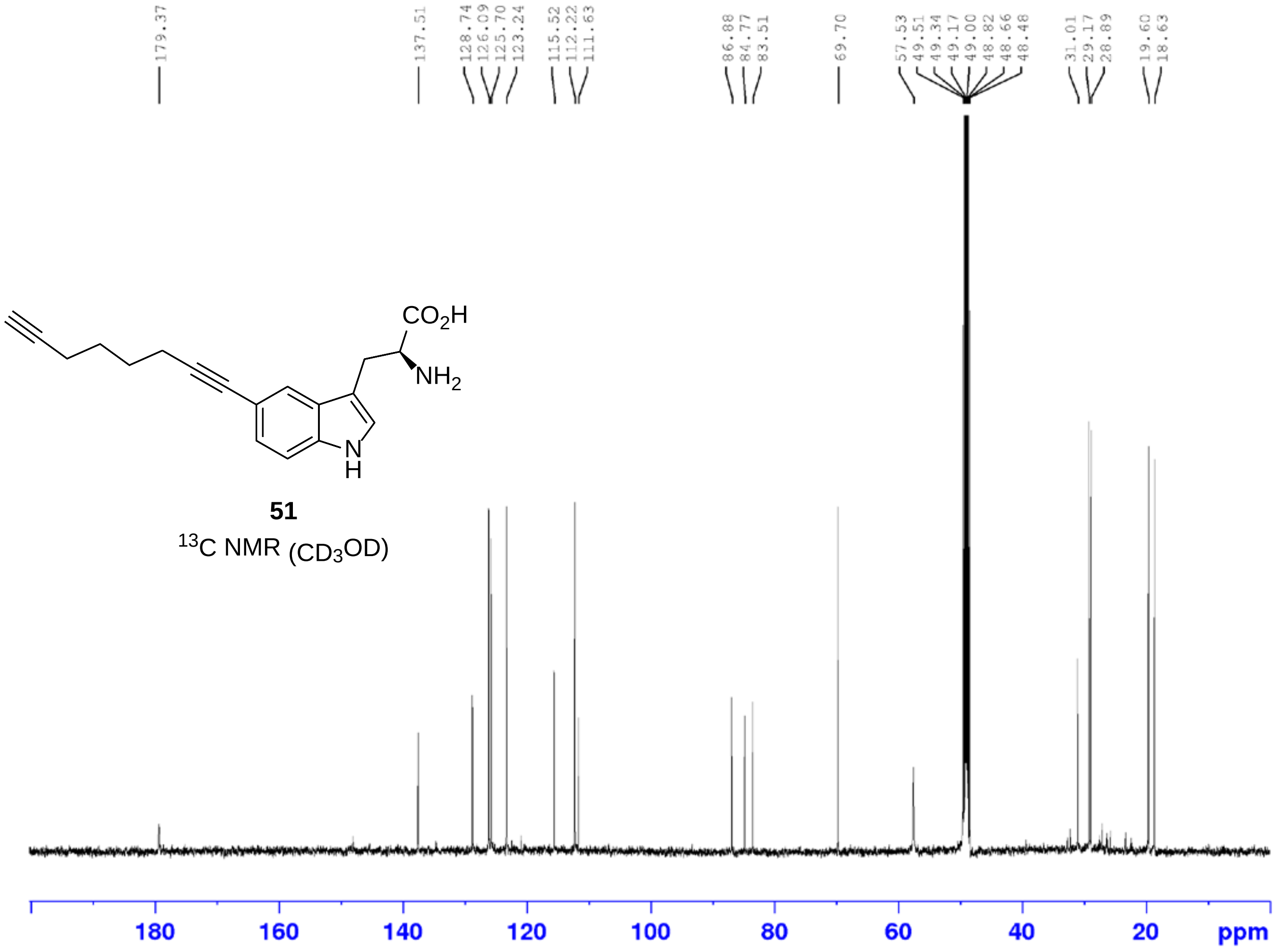

51

^{13}C NMR (CD_3OD)

```
Current Data Parameters
NAME      07132015-11-rjmg-mc410-A
EXPNO                   10
PROCNO                   1

F2 - Acquisition Parameters
Date_             20150713
Time                 21.04
INSTRUM              spect
PROBHD    5 mm CPPBBO BB
PULPROG              udeft
TD                   21424
SOLVENT               MeOD
NS                     400
DS                       0
SWH              29761.904 Hz
FIDRES            1.389185 Hz
AQ               0.3599232 sec
RG                     128
DW                  16.800 usec
DE                   19.86 usec
TE                   295.0 K
D1              3.00000000 sec
D11             0.03000000 sec
D12             0.00002000 sec
D20           200.00000000 sec
TD0                      1

======== CHANNEL f1 ========
SFO1           125.7716219 MHz
NUC1                   13C
P1                   10.00 usec
P13                2000.00 usec
P26                 500.00 usec
PLW1           68.00000000 W
SPNAM[5]     Crp60comp.4
SPOAL5               0.500
SPOFFS5   0 Hz
SPW5           10.39000034 W
SPNAM[8]  Crp60,0.5,20.1
SPOAL8               0.500
SPOFFS8   0 Hz
SPW8           10.39000034 W

======== CHANNEL f2 ========
SFO2           500.1320005 MHz
NUC2                    1H
CPDPRG[2           waltz65
PCPD2                80.00 usec
PLW2           15.00000000 W
PLW12           0.32633999 W

F2 - Processing parameters
SI                  262144
SF             125.7576133 MHz
WDW                     EM
SSB       0
LB                    2.00 Hz
GB        0
PC                    1.40
```

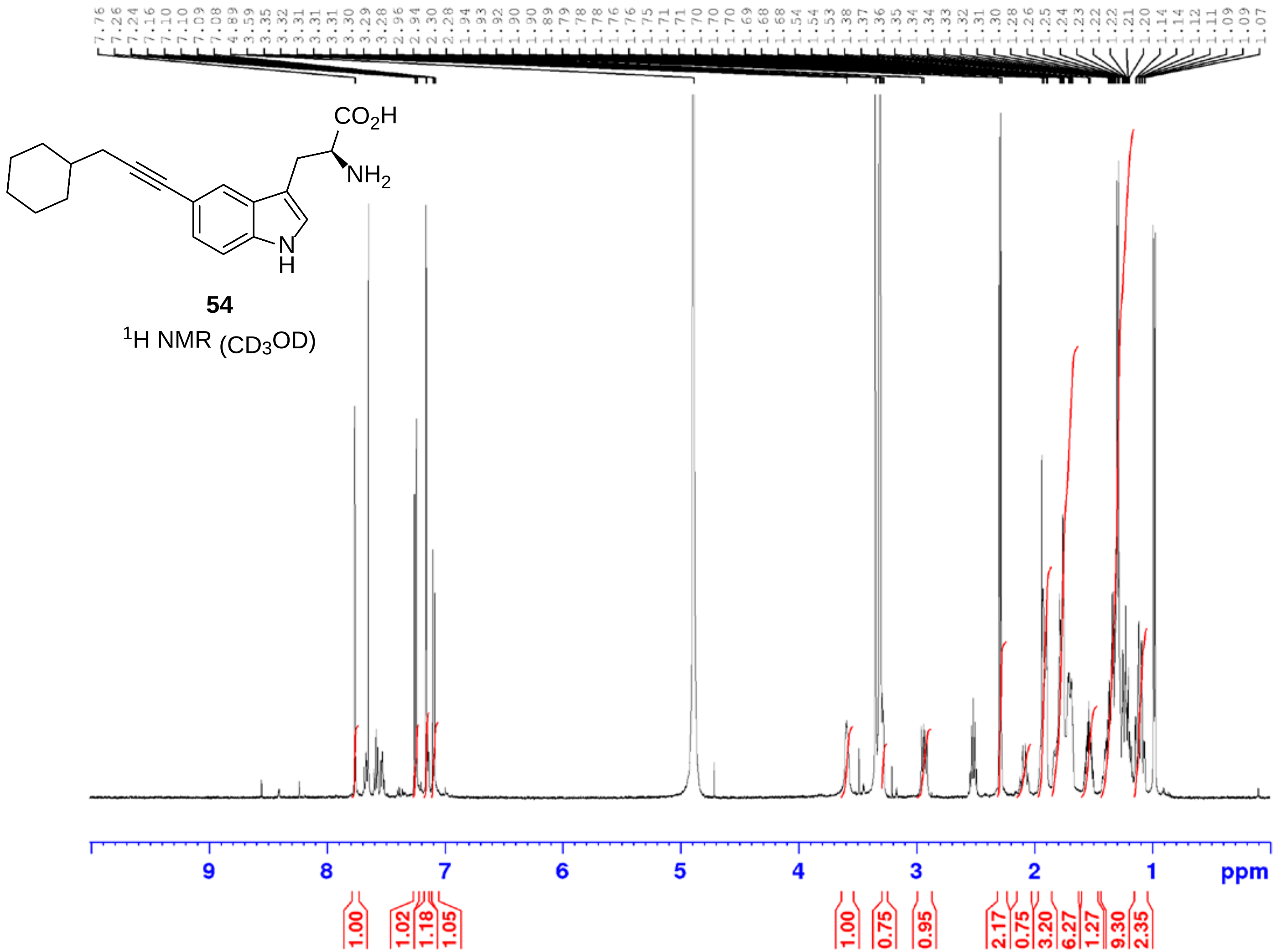

54

^{1}H NMR (CD_3OD)

BRUKER

Current Data Parameters
NAME 03182016-14-rjmg-mc410-F
EXPNO 10
PROCNO 1

F2 - Acquisition Parameters
Date_ 20160318
Time 21.11
INSTRUM spect
PROBHD 5 mm PABBO BB/
PULPROG zg30
TD 65536
SOLVENT MeOD
NS 8
DS 2
SWH 6996.269 Hz
FIDRES 0.106755 Hz
AQ 4.6836395 sec
RG 121.22
DW 71.467 usec
DE 11.38 usec
TE 295.0 K
D1 1.00000000 sec
TD0 1

======== CHANNEL f1 ========
SFO1 499.9329996 MHz
NUC1 1H
P1 10.60 usec
PLW1 16.00000000 W

F2 - Processing parameters
SI 65536
SF 499.9300095 MHz
WDW EM
SSB 0
LB 0.30 Hz
GB 0
PC 1.00

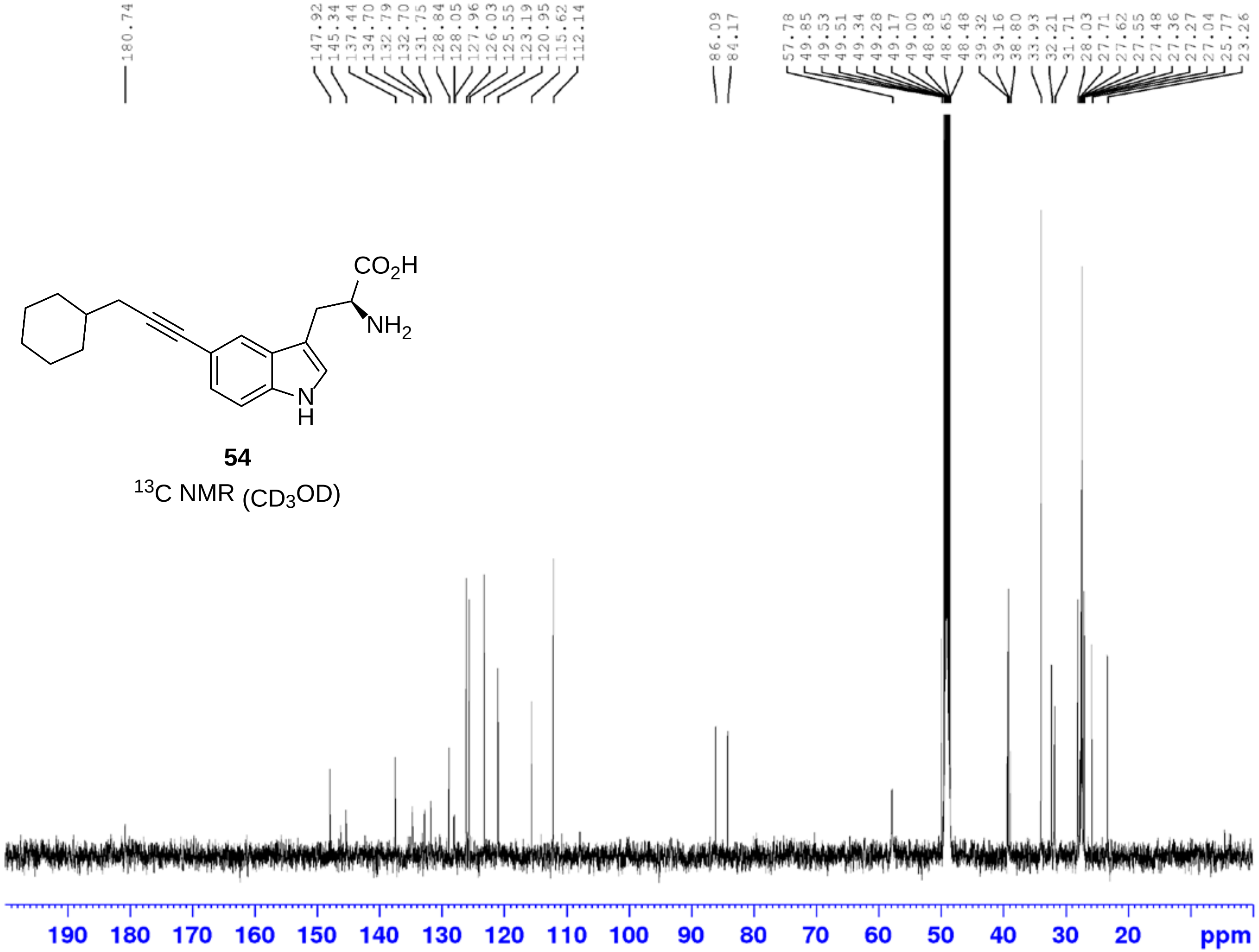

54

^{13}C NMR (CD_3OD)

Current Data Parameters
NAME 03182016-14-rjmg-mc410-F
EXPNO 11
PROCNO 1

F2 - Acquisition Parameters
Date_ 20160318
Time 23.20
INSTRUM spect
PROBHD 5 mm PABBO BB/
PULPROG udeft
TD 21424
SOLVENT MeOD
NS 2000
DS 0
SWH 29761.904 Hz
FIDRES 1.389185 Hz
AQ 0.3599232 sec
RG 121.22
DW 16.800 usec
DE 9.36 usec
TE 295.0 K
D1 3.00000000 sec
D11 0.03000000 sec
D12 0.00002000 sec
D20 200.00000000 sec
TD0 1

======== CHANNEL f1 ========
SFO1 125.7213263 MHz
NUC1 13C
P1 10.00 usec
P13 2000.00 usec
P26 500.00 usec
PLW1 85.00000000 W
SPNAM[5] Crp60comp.4
SPOAL5 0.500
SPOFFS5 0 Hz
SPW5 12.98700047 W
SPNAM[8] Crp60,0.5,20.1
SPOAL8 0.500
SPOFFS8 0 Hz
SPW8 12.98700047 W

======== CHANNEL f2 ========
SFO2 499.9319997 MHz
NUC2 1H
CPDPRG[2 waltz65
PCPD2 80.00 usec
PLW2 16.00000000 W
PLW12 0.27563000 W

F2 - Processing parameters
SI 262144
SF 125.7073227 MHz
WDW EM
SSB 0
LB 2.00 Hz
GB 0
PC 1.40

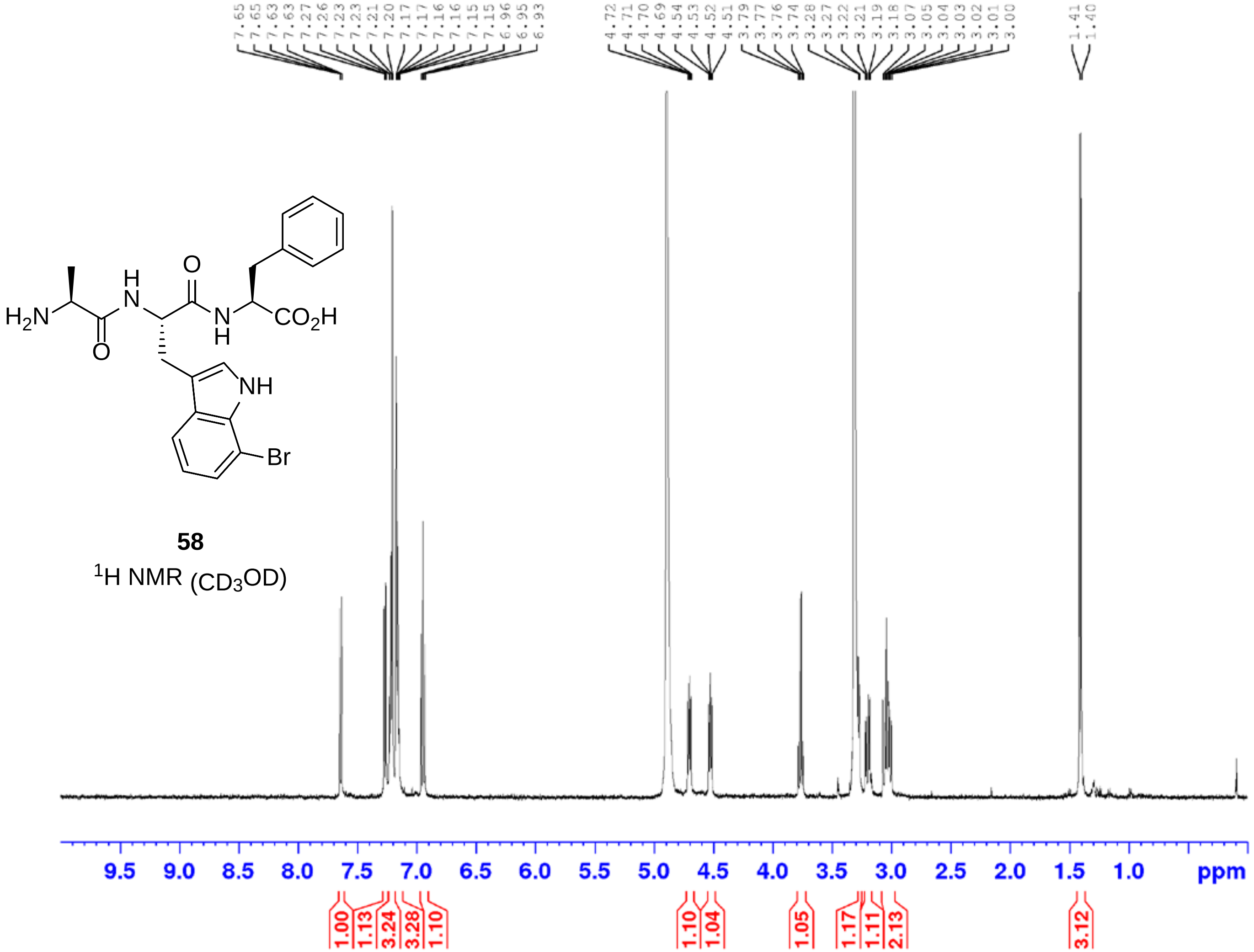

58

^{1}H NMR (CD$_3$OD)

```
Current Data Parameters
NAME       03182016-15-rjmg-mc410-F
EXPNO                    10
PROCNO                    1

F2 - Acquisition Parameters
Date_              20160318
Time                  23.25
INSTRUM               spect
PROBHD    5 mm PABBO BB/
PULPROG                zg30
TD                    65536
SOLVENT                MeOD
NS                        8
DS                        2
SWH                6996.269 Hz
FIDRES             0.106755 Hz
AQ                4.6836395 sec
RG                   121.22
DW                   71.467 usec
DE                    11.38 usec
TE                    295.0 K
D1               1.00000000 sec
TD0                       1

======== CHANNEL f1 ========
SFO1            499.9329996 MHz
NUC1                     1H
P1                    10.60 usec
PLW1            16.00000000 W

F2 - Processing parameters
SI                    65536
SF              499.9300096 MHz
WDW                      EM
SSB      0
LB                     0.30 Hz
GB       0
PC                     1.00
```

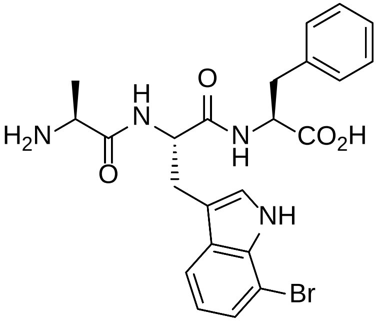

58

^{13}C NMR (CD_3OD)

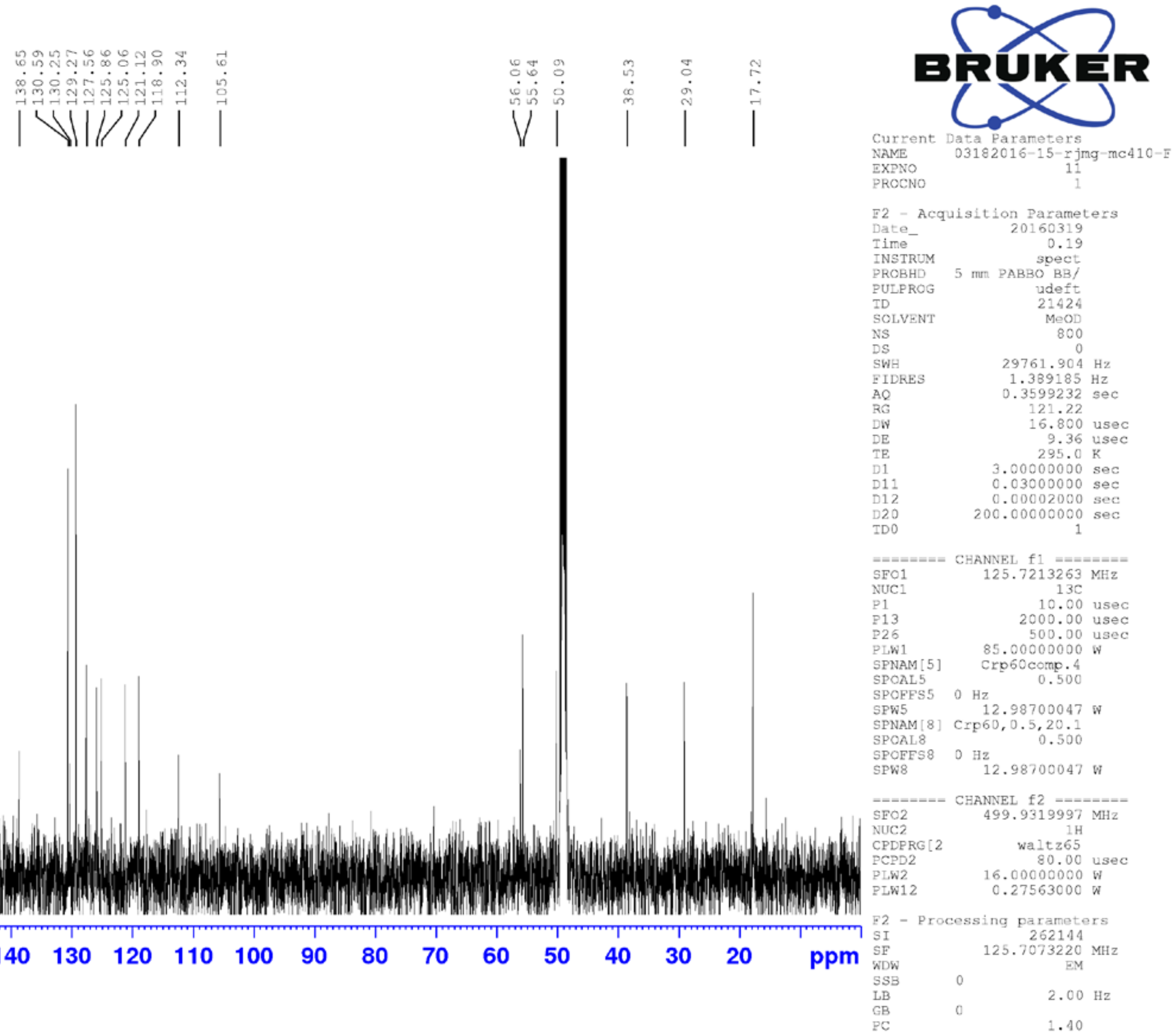

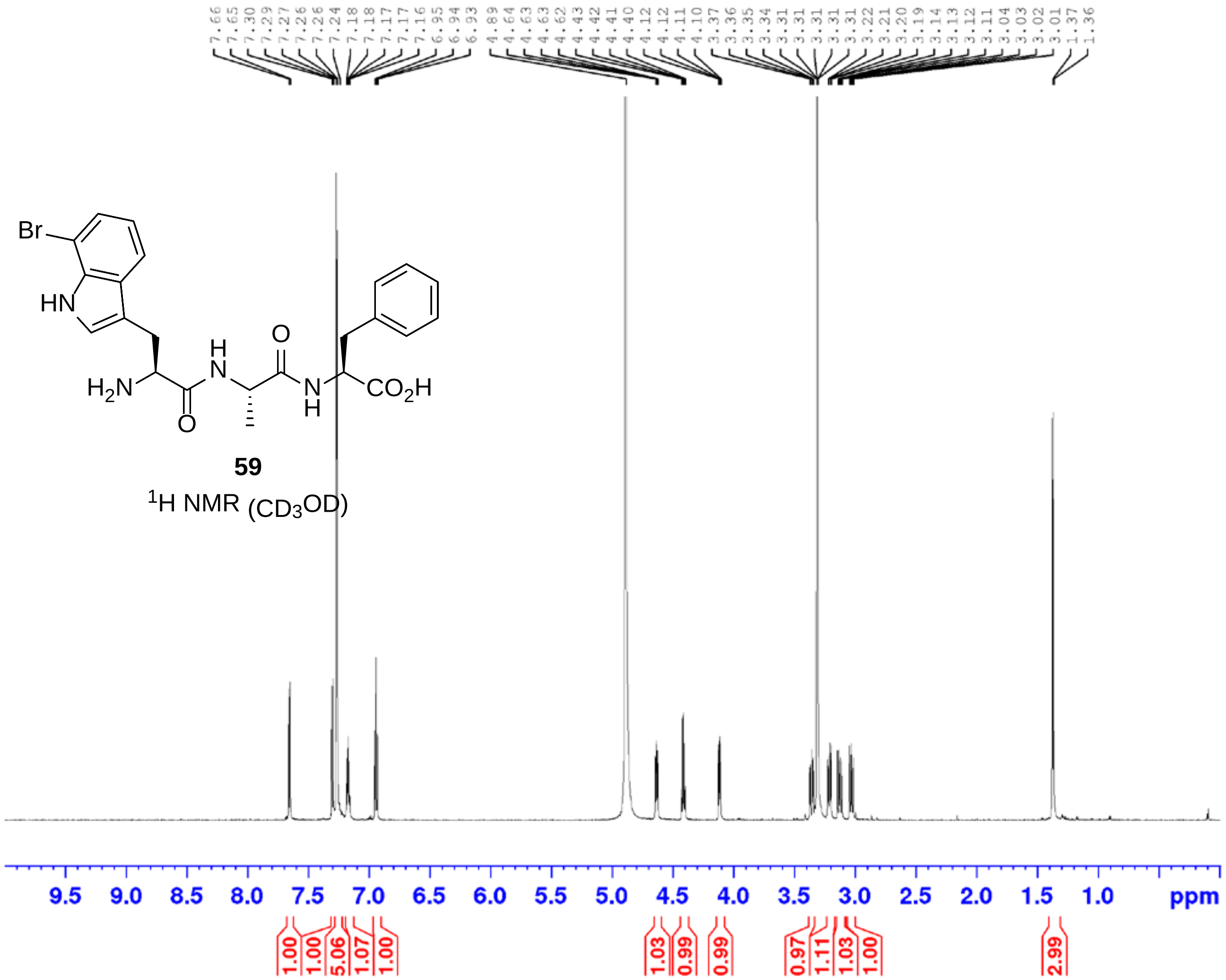

59

^{1}H NMR (CD_3OD)

```
Current Data Parameters
NAME      03222016-11-rjmg-mc410-E
EXPNO                   10
PROCNO                   1

F2 - Acquisition Parameters
Date_             20160322
Time                 16.49
INSTRUM              spect
PROBHD    5 mm CPPTCI 1H
PULPROG               zg30
TD                   65536
SOLVENT               MeOD
NS                       4
DS                       2
SWH               9803.922 Hz
FIDRES            0.149596 Hz
AQ               3.3423359 sec
RG                  111.35
DW                  51.000 usec
DE                   59.79 usec
TE                   295.0 K
D1              1.00000000 sec
TD0                      1

======== CHANNEL f1 ========
SFO1          700.1342008 MHz
NUC1                    1H
P1                    8.00 usec
PLW1           15.30000019 W

F2 - Processing parameters
SI                   65536
SF             700.1300134 MHz
WDW                     EM
SSB          0
LB                    0.30 Hz
GB           0
PC                    1.00
```

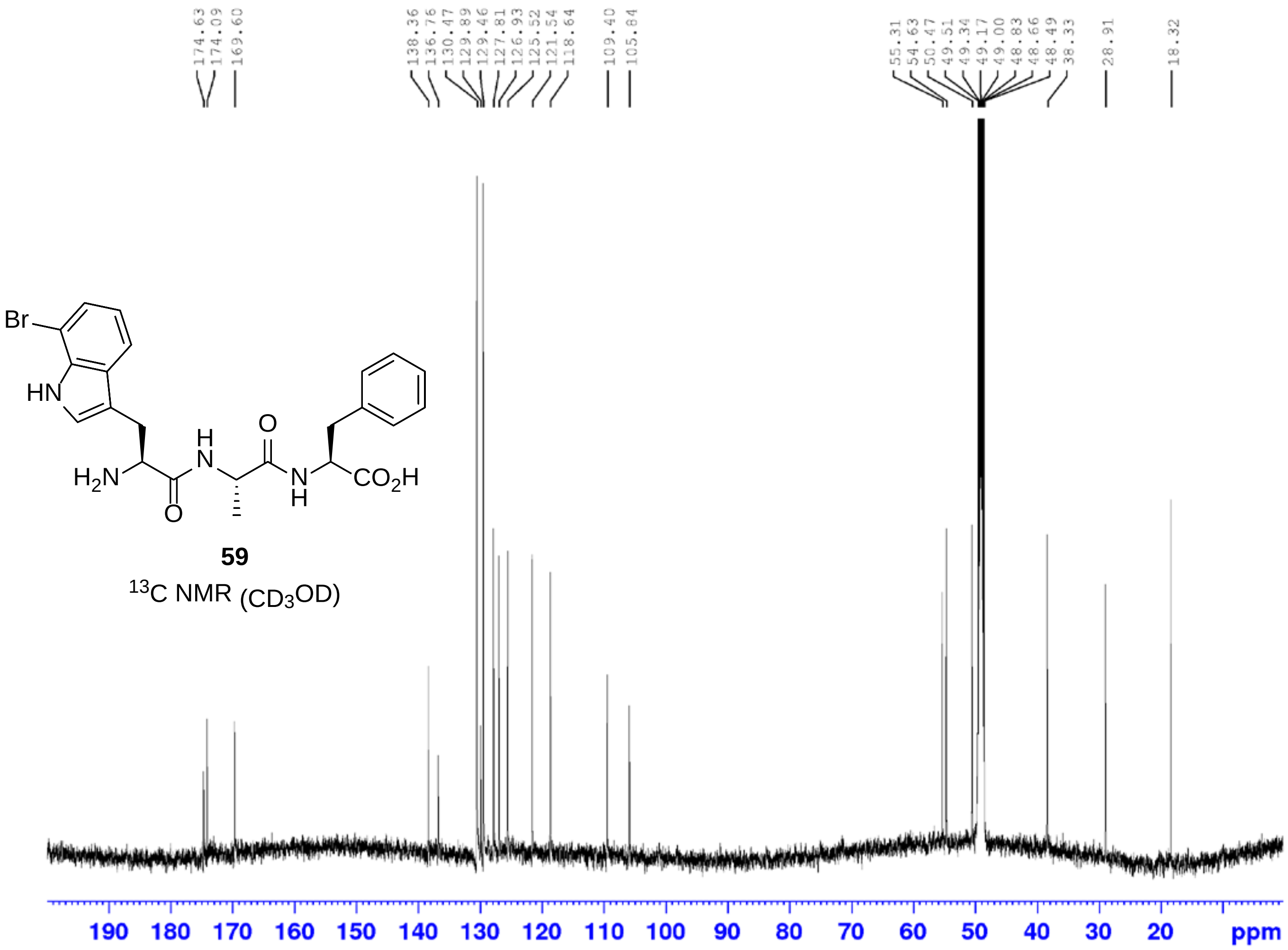

59

^{13}C NMR (CD_3OD)

BRUKER

```
Current Data Parameters
NAME      03232016-11-rjmg-mc410-A
EXPNO                    11
PROCNO                    1

F2 - Acquisition Parameters
Date_              20160323
Time                  21.48
INSTRUM               spect
PROBHD   5 mm CPPBBO BB
PULPROG               udeft
TD                    21424
SOLVENT                MeOD
NS                     1600
DS                        0
SWH               29761.904 Hz
FIDRES             1.389185 Hz
AQ                0.3599232 sec
RG                      128
DW                   16.800 usec
DE                    19.22 usec
TE                    295.0 K
D1               3.00000000 sec
D11              0.03000000 sec
D12              0.00002000 sec
D20            200.00000000 sec
TD0                       1

======== CHANNEL f1 ========
SFO1            125.7716219 MHz
NUC1                    13C
P1                    11.00 usec
P13                 2000.00 usec
P26                  500.00 usec
PLW1            63.00000000 W
SPNAM[5]        Crp60comp.4
SPOAL5                0.500
SPOFFS5    0 Hz
SPW5            11.64700031 W
SPNAM[8]  Crp60,0.5,20.1
SPOAL8                0.500
SPOFFS8    0 Hz
SPW8            11.64700031 W

======== CHANNEL f2 ========
SFO2            500.1320005 MHz
NUC2                     1H
CPDPRG[2            waltz65
PCPD2                 80.00 usec
PLW2            14.00000000 W
PLW12            0.31500000 W

F2 - Processing parameters
SI                   262144
SF              125.7576120 MHz
WDW                      EM
SSB        0
LB                     2.00 Hz
GB         0
PC                     1.40
```

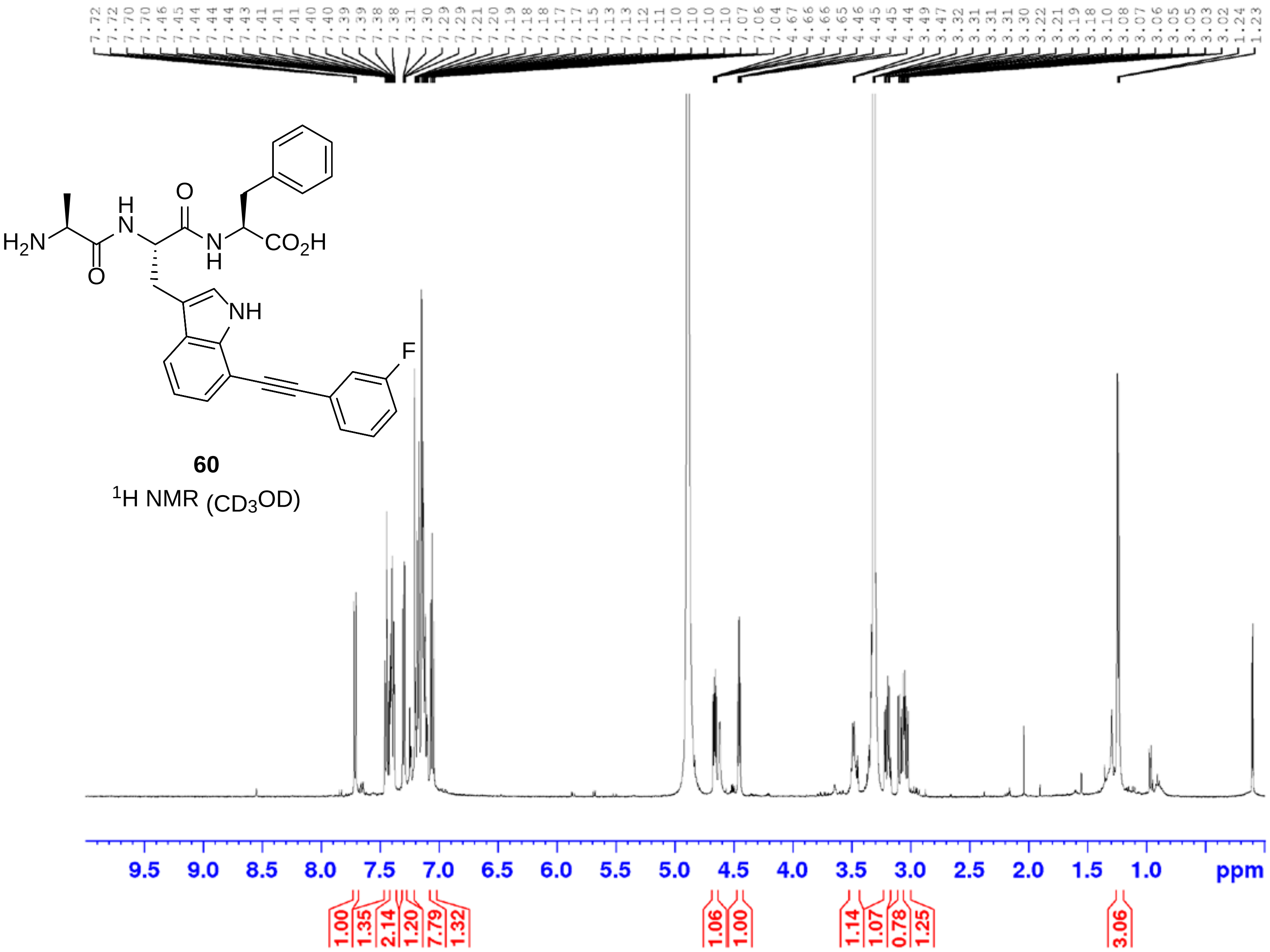

60

^{1}H NMR (CD_3OD)

Current Data Parameters
NAME 03142016-30-rjmg-mc410-A
EXPNO 10
PROCNO 1

F2 - Acquisition Parameters
Date_ 20160314
Time 21.02
INSTRUM spect
PROBHD 5 mm CPPBBO BB
PULPROG zg30
TD 65536
SOLVENT MeOD
NS 64
DS 2
SWH 7002.801 Hz
FIDRES 0.106854 Hz
AQ 4.6792703 sec
RG 64
DW 71.400 usec
DE 10.95 usec
TE 295.0 K
D1 1.00000000 sec
TD0 1

======== CHANNEL f1 ========
SFO1 500.1330008 MHz
NUC1 1H
P1 12.50 usec
PLW1 14.00000000 W

F2 - Processing parameters
SI 65536
SF 500.1300097 MHz
WDW EM
SSB 0
LB 0.30 Hz
GB 0
PC 1.00

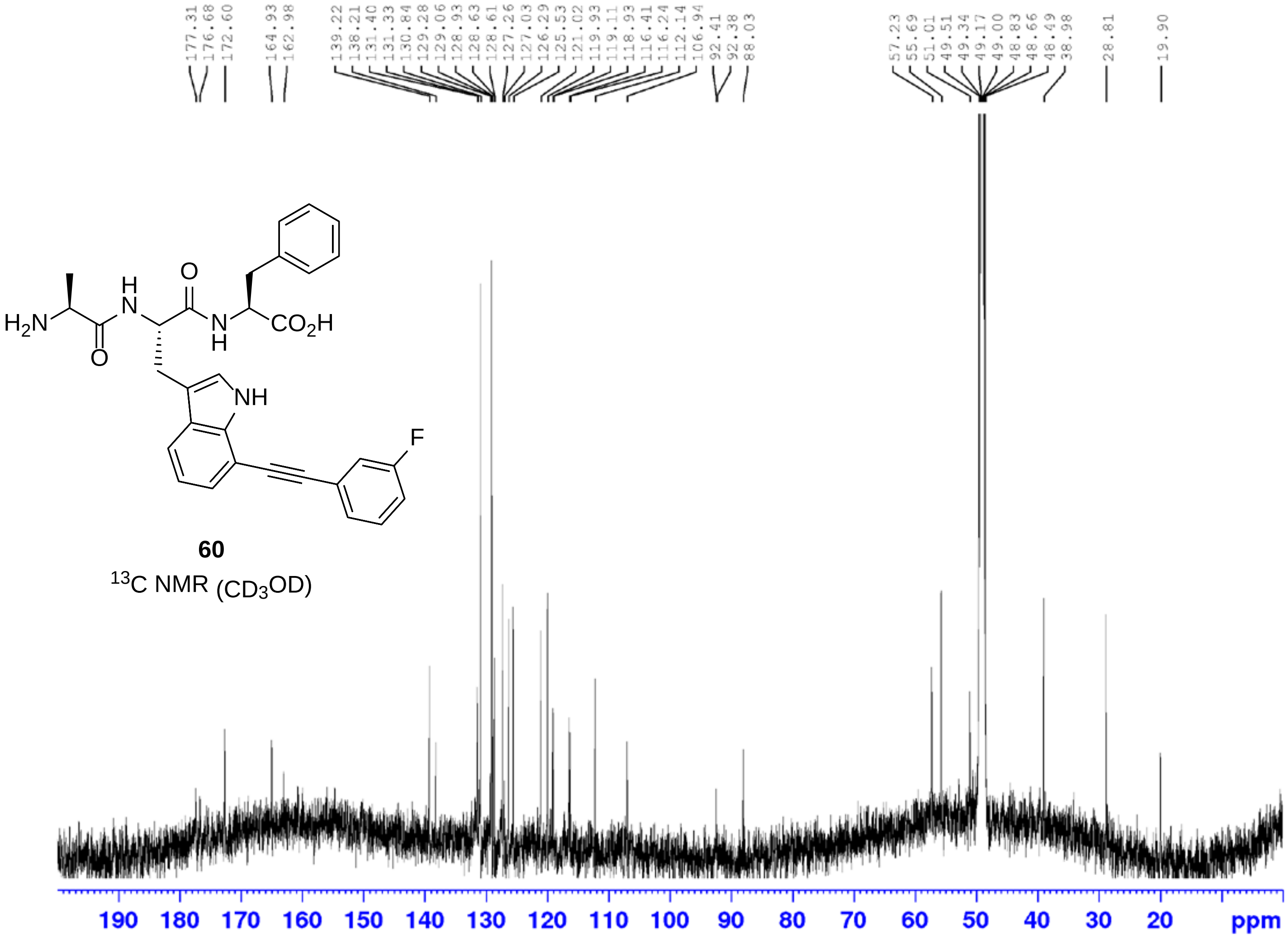

60

^{13}C NMR (CD_3OD)


```
Current Data Parameters
NAME      03142016-30-rjmg-mc410-A
EXPNO                   12
PROCNO                   1

F2 - Acquisition Parameters
Date_             20160314
Time                 22.53
INSTRUM              spect
PROBHD    5 mm CPPBBO BB
PULPROG              udeft
TD                   21424
SOLVENT               MeOD
NS                    1600
DS                       0
SWH              29761.904 Hz
FIDRES            1.389185 Hz
AQ               0.3599232 sec
RG                     128
DW                  16.800 usec
DE                   19.22 usec
TE                   295.0 K
D1              3.00000000 sec
D11             0.03000000 sec
D12             0.00002000 sec
D20           200.00000000 sec
TD0                      1

======== CHANNEL f1 ========
SFO1          125.7716219 MHz
NUC1                   13C
P1                   11.00 usec
P13                2000.00 usec
P26                 500.00 usec
PLW1           63.00000000 W
SPNAM[5]     Crp60comp.4
SPOAL5               0.500
SPOFFS5    0 Hz
SPW5           11.64700031 W
SPNAM[8]   Crp60,0.5,20.1
SPOAL8               0.500
SPOFFS8    0 Hz
SPW8           11.64700031 W

======== CHANNEL f2 ========
SFO2          500.1320005 MHz
NUC2                    1H
CPDPRG[2           waltz65
PCPD2                80.00 usec
PLW2           14.00000000 W
PLW12           0.31500000 W

F2 - Processing parameters
SI                  262144
SF            125.7576119 MHz
WDW                     EM
SSB      0
LB                    2.00 Hz
GB       0
PC                    1.40
```

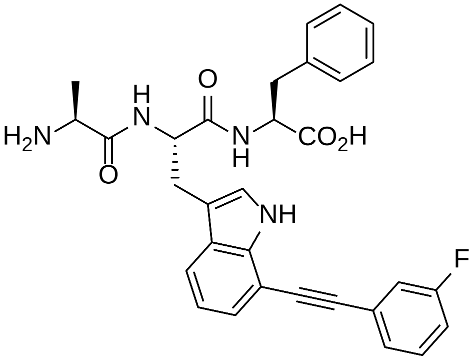

60

$^{19}F\{^{1}H\}$ NMR (CD_3OD)

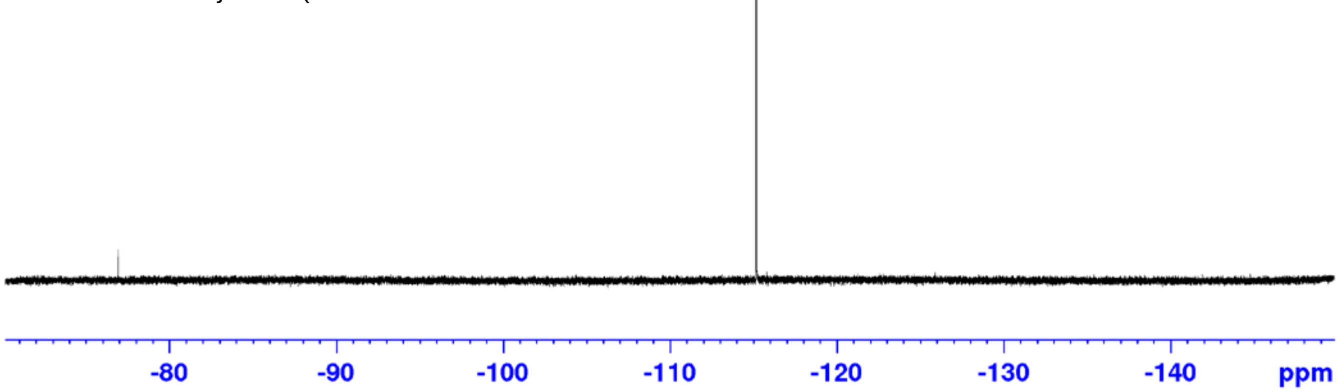


```
Current Data Parameters
NAME       03152016-2-rjmg-mc410-M
EXPNO                    10
PROCNO                    1

F2 - Acquisition Parameters
Date_            20160315
Time                16.50
INSTRUM           AVII400
PROBHD     5 mm PABBO BB/
PULPROG    zgfhigqn.3.and
TD                  65536
SOLVENT              MeOD
NS                     64
DS                      4
SWH             30000.000 Hz
FIDRES           0.457764 Hz
AQ              1.0922667 sec
RG                    128
DW                 16.667 usec
DE                   8.37 usec
TE                  295.3 K
D1             1.00000000 sec
D11            0.03000000 sec
D12            0.00002000 sec
TD0                     1

======== CHANNEL f1 ========
SFO1          376.4569514 MHz
NUC1                  19F
P1                  15.00 usec
PLW1          17.00000000 W

======== CHANNEL f2 ========
SFO2          400.1316005 MHz
NUC2                   1H
CPDPRG[2          waltz65
PCPD2               90.00 usec
PLW2          16.00000000 W
PLW12          0.48688999 W

F2 - Processing parameters
SI                  65536
SF            376.4983662 MHz
WDW                    EM
SSB      0
LB                   0.30 Hz
GB       0
PC                   1.00
```

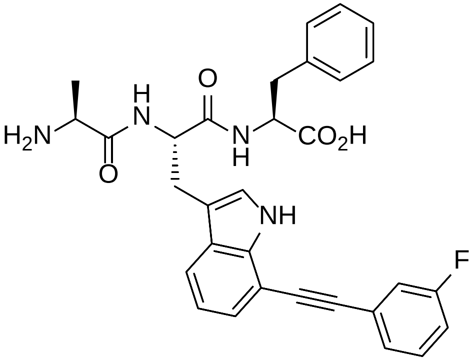

60

^{19}F NMR (CD_3OD)

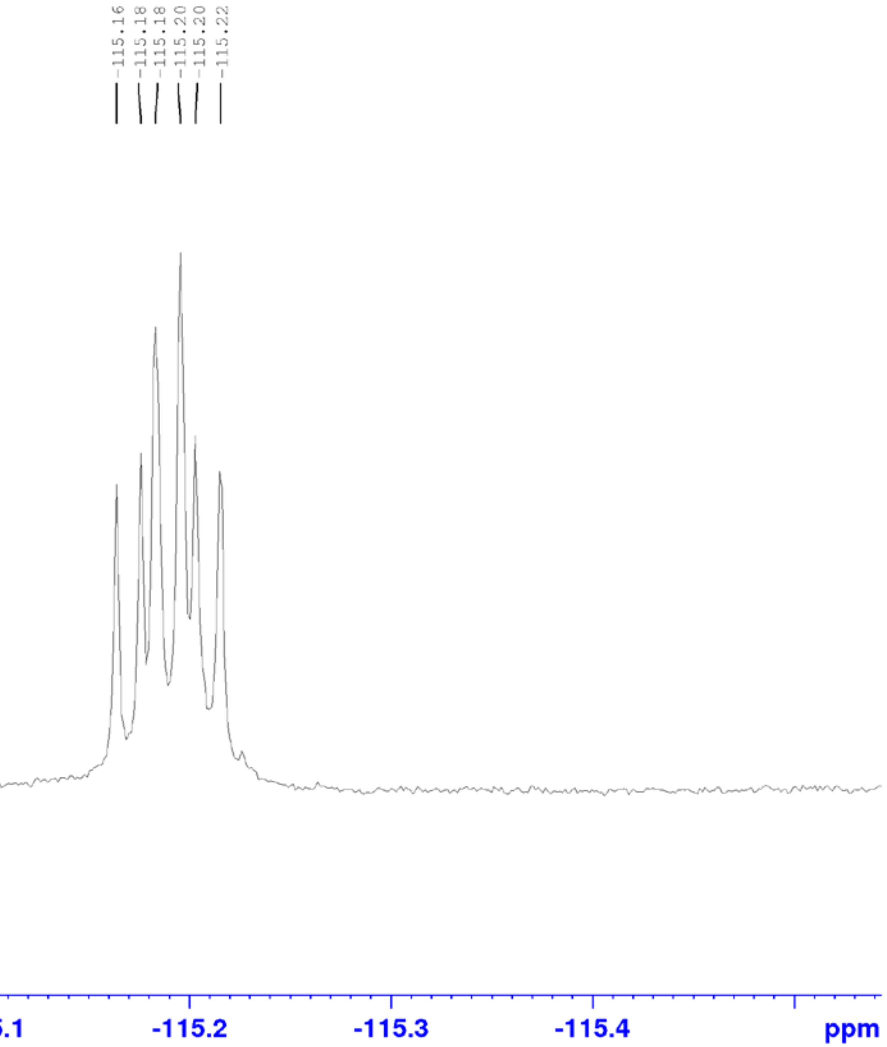

Current Data Parameters
NAME 03142016-30-rjmg-mc410-A
EXPNO 11
PROCNO 1

F2 - Acquisition Parameters
Date_ 20160314
Time 21.06
INSTRUM spect
PROBHD 5 mm CPPBBO BB
PULPROG zg
TD 65536
SOLVENT MeOD
NS 32
DS 4
SWH 37500.000 Hz
FIDRES 0.572205 Hz
AQ 0.8738133 sec
RG 20.2
DW 13.333 usec
DE 18.16 usec
TE 295.0 K
D1 1.00000000 sec
TD0 1

======== CHANNEL f1 ========
SFO1 470.5406119 MHz
NUC1 19F
P1 15.00 usec
PLW1 15.00000000 W

F2 - Processing parameters
SI 65536
SF 470.5923772 MHz
WDW EM
SSB 0
LB 0.30 Hz
GB 0
PC 1.00

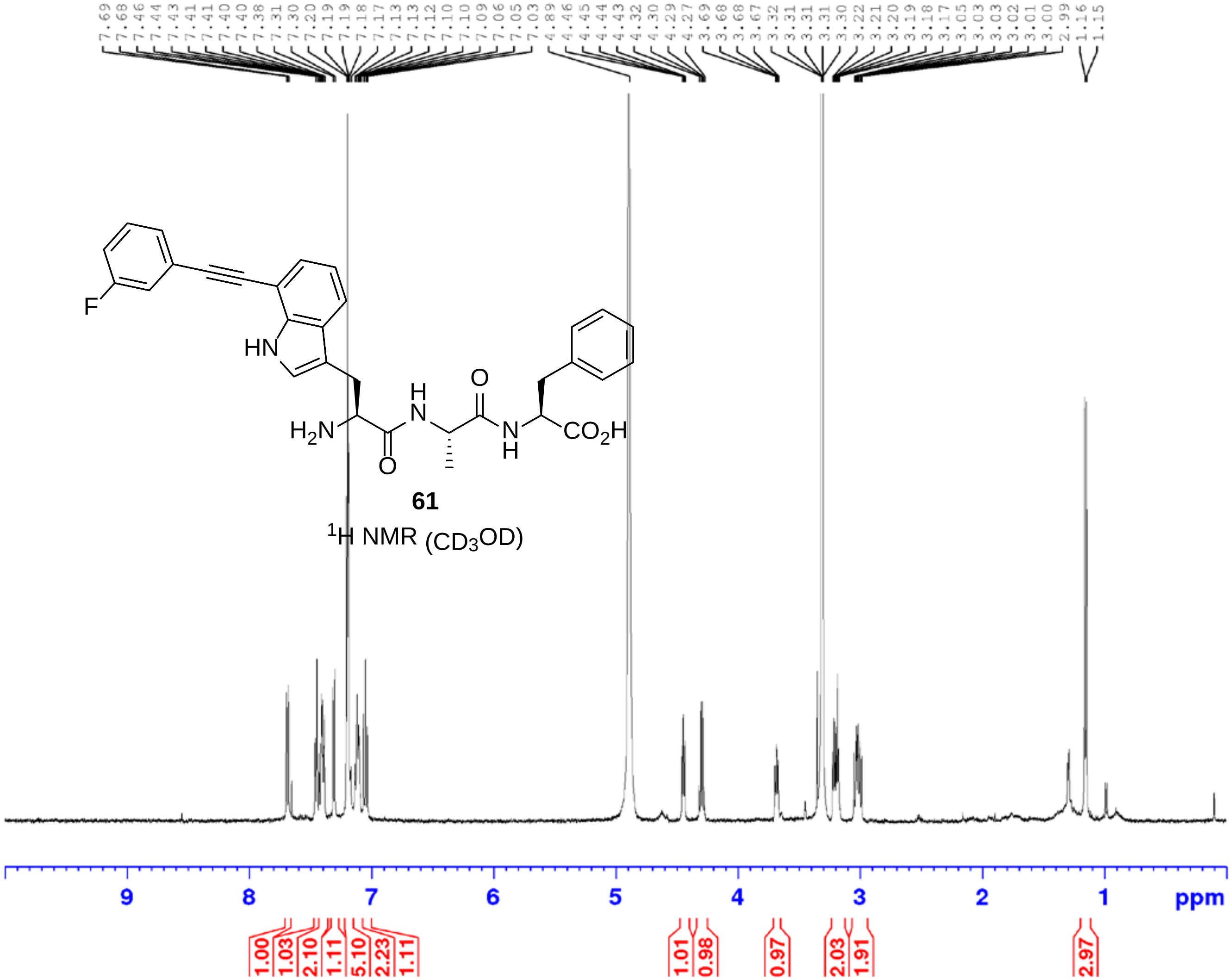

Current Data Parameters
NAME 03212016-17-rjmg-mc410-F
EXPNO 10
PROCNO 1

F2 - Acquisition Parameters
Date_ 20160321
Time 16.28
INSTRUM spect
PROBHD 5 mm PABBO BB/
PULPROG zg30
TD 65536
SOLVENT MeOD
NS 8
DS 2
SWH 6996.269 Hz
FIDRES 0.106755 Hz
AQ 4.6836395 sec
RG 121.22
DW 71.467 usec
DE 11.38 usec
TE 295.0 K
D1 1.00000000 sec
TD0 1

======== CHANNEL f1 ========
SFO1 499.9329996 MHz
NUC1 1H
P1 10.60 usec
PLW1 16.00000000 W

F2 - Processing parameters
SI 65536
SF 499.9300094 MHz
WDW EM
SSB 0
LB 0.30 Hz
GB 0
PC 1.00

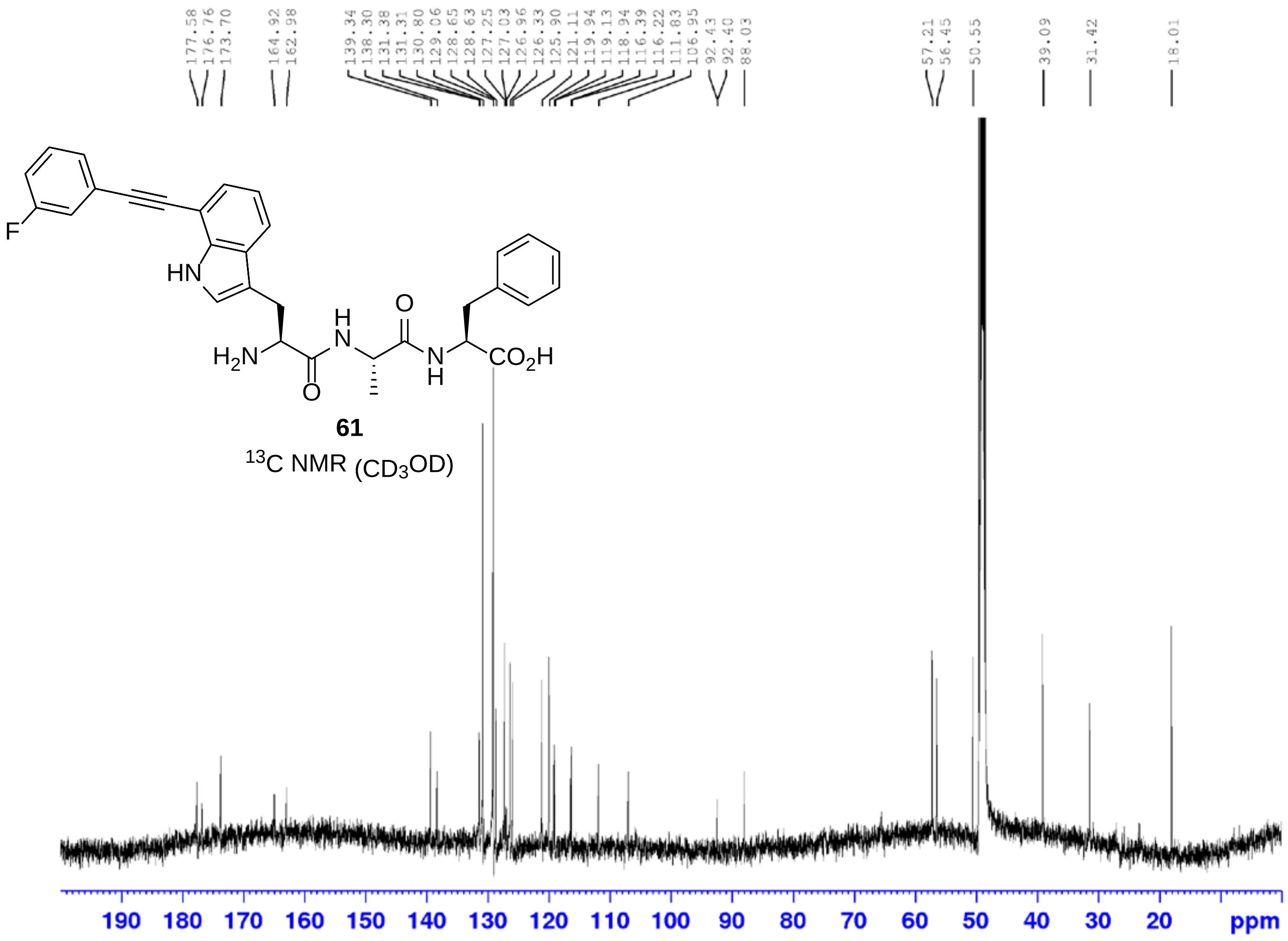


```
Current Data Parameters
NAME       03222016-1-rjmg-mc410-A
EXPNO                   10
PROCNO                   1

F2 - Acquisition Parameters
Date_              20160322
Time                  21.11
INSTRUM               spect
PROBHD   5 mm CPPBBO BB
PULPROG               udeft
TD                    21424
SOLVENT                MeOD
NS                     2000
DS                        0
SWH            29761.904 Hz
FIDRES          1.389185 Hz
AQ             0.3599232 sec
RG                      128
DW                16.800 usec
DE                 19.22 usec
TE                 295.0 K
D1            3.00000000 sec
D11           0.03000000 sec
D12           0.00002000 sec
D20         200.00000000 sec
TD0                       1

======== CHANNEL f1 ========
SFO1         125.7716219 MHz
NUC1                    13C
P1                 11.00 usec
P13              2000.00 usec
P26               500.00 usec
PLW1         63.00000000 W
SPNAM[5]     Crp60comp.4
SPOAL5                0.500
SPOFFS5   0 Hz
SPW5         11.64700031 W
SPNAM[8]  Crp60,0.5,20.1
SPOAL8                0.500
SPOFFS8   0 Hz
SPW8         11.64700031 W

======== CHANNEL f2 ========
SFO2         500.1320005 MHz
NUC2                     1H
CPDPRG[2            waltz65
PCPD2              80.00 usec
PLW2         14.00000000 W
PLW12         0.31500000 W

F2 - Processing parameters
SI                   262144
SF           125.7576123 MHz
WDW                      EM
SSB       0
LB                  2.00 Hz
GB        0
PC                     1.40
```

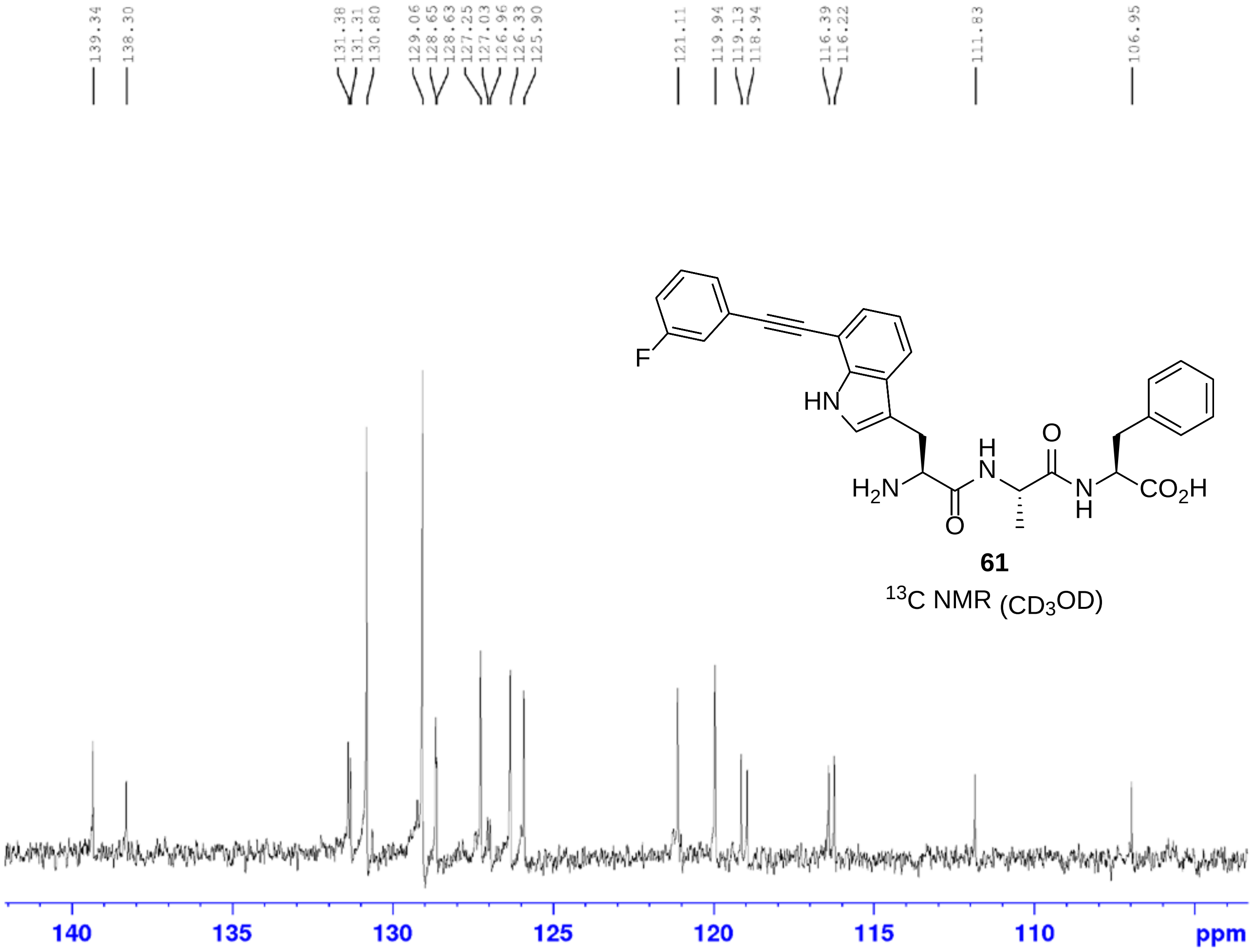

61

^{13}C NMR (CD_3OD)

BRUKER

```
Current Data Parameters
NAME      03222016-1-rjmg-mc410-A
EXPNO                    10
PROCNO                    1

F2 - Acquisition Parameters
Date_              20160322
Time                  21.11
INSTRUM               spect
PROBHD     5 mm CPPBBO BB
PULPROG               udeft
TD                    21424
SOLVENT                MeOD
NS                     2000
DS                        0
SWH             29761.904 Hz
FIDRES           1.389185 Hz
AQ              0.3599232 sec
RG                      128
DW                   16.800 usec
DE                    19.22 usec
TE                    295.0 K
D1               3.00000000 sec
D11              0.03000000 sec
D12              0.00002000 sec
D20            200.00000000 sec
TD0                       1

======== CHANNEL f1 ========
SFO1         125.7716219 MHz
NUC1                 13C
P1                  11.00 usec
P13               2000.00 usec
P26                500.00 usec
PLW1          63.00000000 W
SPNAM[5]     Crp60comp.4
SPOAL5             0.500
SPOFFS5    0 Hz
SPW5          11.64700031 W
SPNAM[8]  Crp60,0.5,20.1
SPOAL8             0.500
SPOFFS8    0 Hz
SPW8          11.64700031 W

======== CHANNEL f2 ========
SFO2         500.1320005 MHz
NUC2                  1H
CPDPRG[2          waltz65
PCPD2               80.00 usec
PLW2          14.00000000 W
PLW12          0.31500000 W

F2 - Processing parameters
SI                 262144
SF            125.7576123 MHz
WDW                    EM
SSB         0
LB                   2.00 Hz
GB          0
PC                   1.40
```

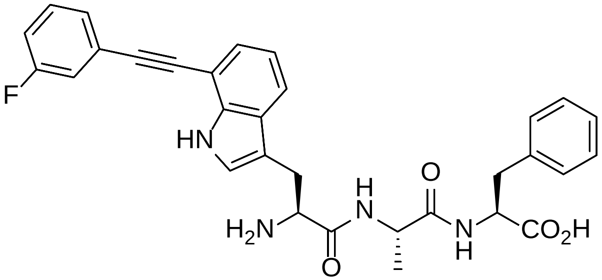

61

$^{19}F\{^{1}H\}$ NMR (CD_3OD)

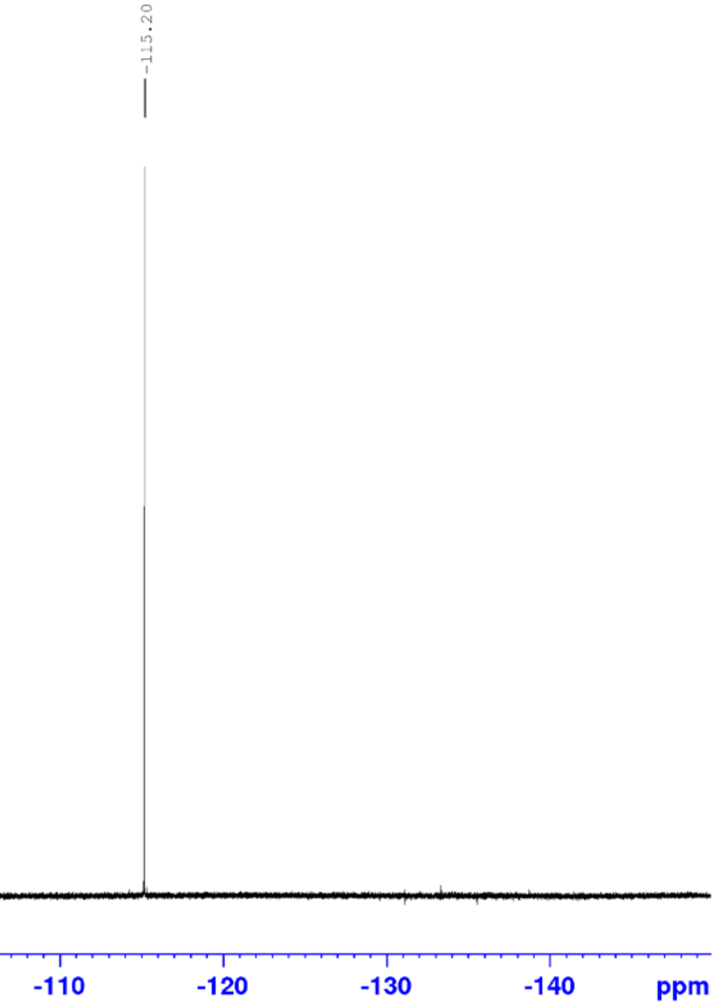


```
Current Data Parameters
NAME      03212016-17-rjmg-mc410-F
EXPNO                   11
PROCNO                   1

F2 - Acquisition Parameters
Date_             20160321
Time                 16.31
INSTRUM              spect
PROBHD    5 mm PABBO BB/
PULPROG   zgfhigqn.3.and
TD                   65536
SOLVENT               MeOD
NS                      32
DS                       4
SWH              37500.000 Hz
FIDRES            0.572205 Hz
AQ               0.8738133 sec
RG                   77.86
DW                  13.333 usec
DE                    6.75 usec
TE                   295.0 K
D1              1.00000000 sec
D11             0.03000000 sec
D12             0.00002000 sec
TD0                      1

======== CHANNEL f1 ========
SFO1        470.3524446 MHz
NUC1                19F
P1                14.60 usec
PLW1        48.00000000 W

======== CHANNEL f2 ========
SFO2        499.9319997 MHz
NUC2                 1H
CPDPRG[2        waltz65
PCPD2             80.00 usec
PLW2        16.00000000 W
PLW12        0.27563000 W

F2 - Processing parameters
SI                65536
SF          470.4041892 MHz
WDW                  EM
SSB       0
LB                 0.30 Hz
GB        0
PC                 1.00
```

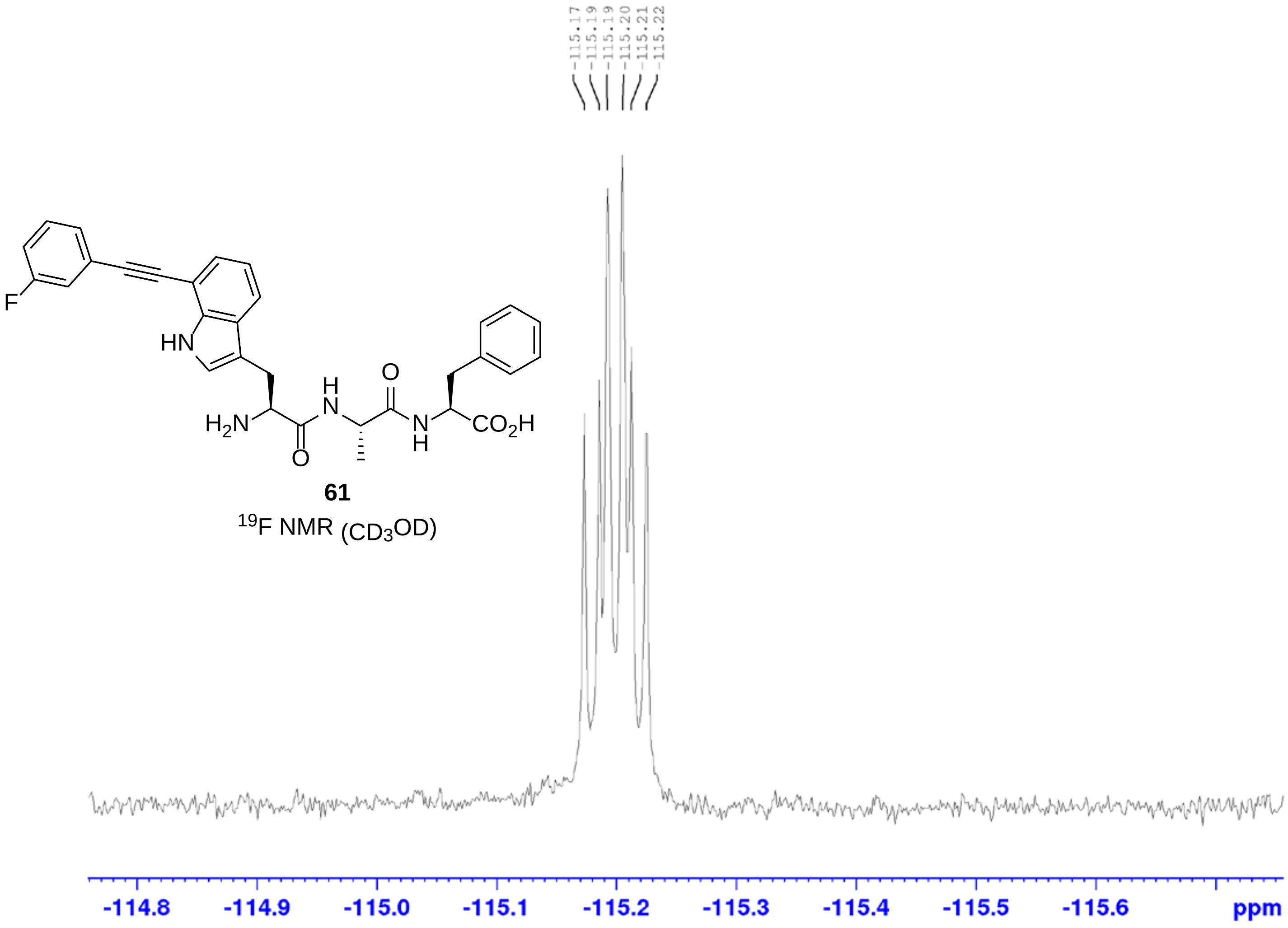

61

^{19}F NMR (CD_3OD)

Current Data Parameters
NAME 03232016-5-rjmg-mc410-F
EXPNO 10
PROCNO 1

F2 - Acquisition Parameters
Date_ 20160323
Time 14.45
INSTRUM spect
PROBHD 5 mm PABBO BB/
PULPROG zgflqn
TD 65536
SOLVENT MeOD
NS 32
DS 4
SWH 37500.000 Hz
FIDRES 0.572205 Hz
AQ 0.8738133 sec
RG 121.22
DW 13.333 usec
DE 6.75 usec
TE 295.0 K
D1 1.00000000 sec
TD0 1

======== CHANNEL f1 ========
SFO1 470.3524446 MHz
NUC1 19F
P1 14.60 usec
PLW1 48.00000000 W

F2 - Processing parameters
SI 65536
SF 470.4041892 MHz
WDW EM
SSB 0
LB 0.30 Hz
GB 0
PC 1.00

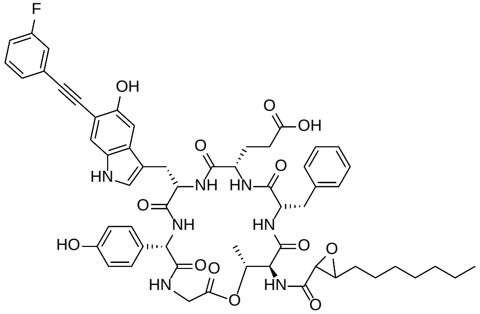

64

^{19}F NMR (D_2O)

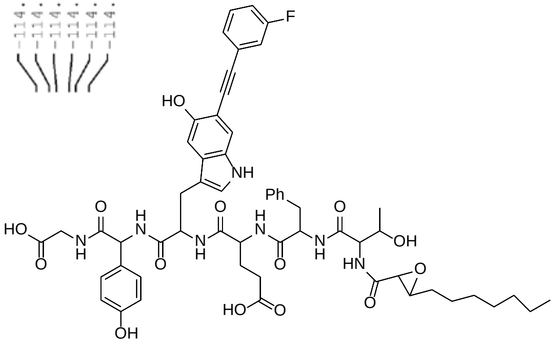

65

^{19}F NMR (D_2O)

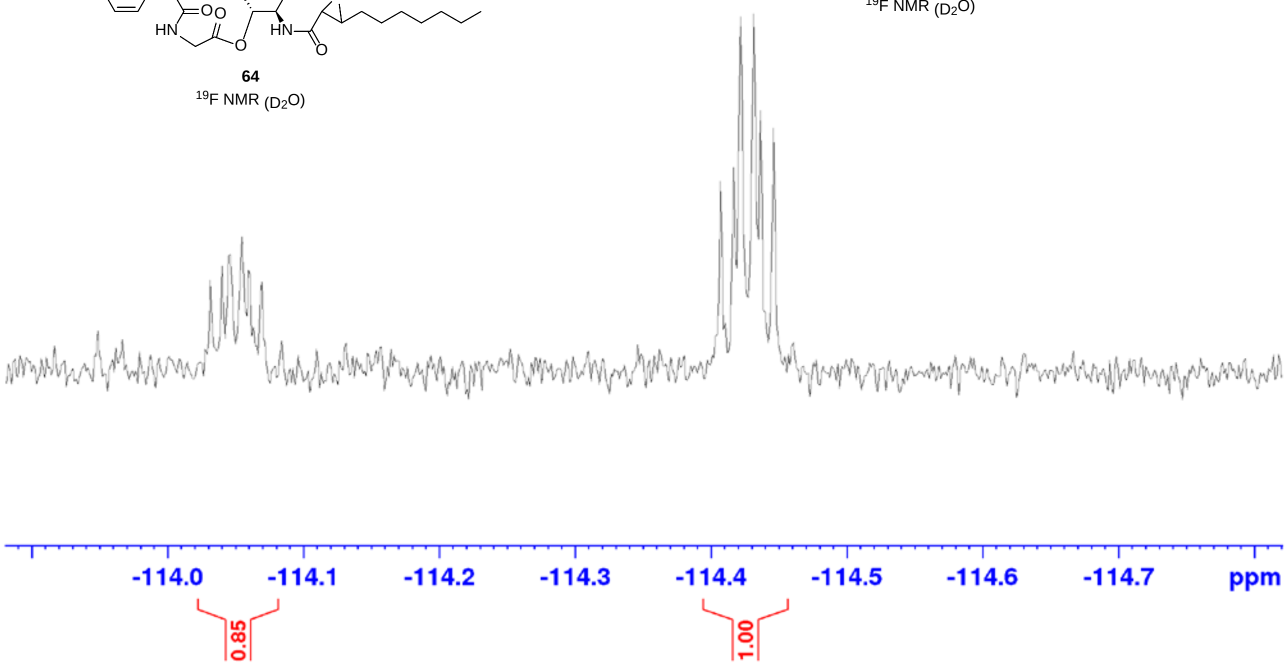


```
Current Data Parameters
NAME        07012016-7-rjmg-mc410-H
EXPNO                    10
PROCNO                    1

F2 - Acquisition Parameters
Date_              20160701
Time                  15.34
INSTRUM               spect
PROBHD    5 mm CPPTCI 1H
PULPROG                  zg
TD                    65536
SOLVENT                 D2O
NS                      256
DS                        4
SWH             52631.578 Hz
FIDRES           0.803094 Hz
AQ              0.6225920 sec
RG                    20.04
DW                    9.500 usec
DE                    18.25 usec
TE                    295.0 K
D1             1.00000000 sec
TD0                       1

======== CHANNEL f1 ========
SFO1          658.7046395 MHz
NUC1                    19F
P1                    11.00 usec
PLW1           13.80000019 W

F2 - Processing parameters
SI                    65536
SF            658.7803992 MHz
WDW                      EM
SSB       0
LB                     0.30 Hz
GB        0
PC                     1.00
```

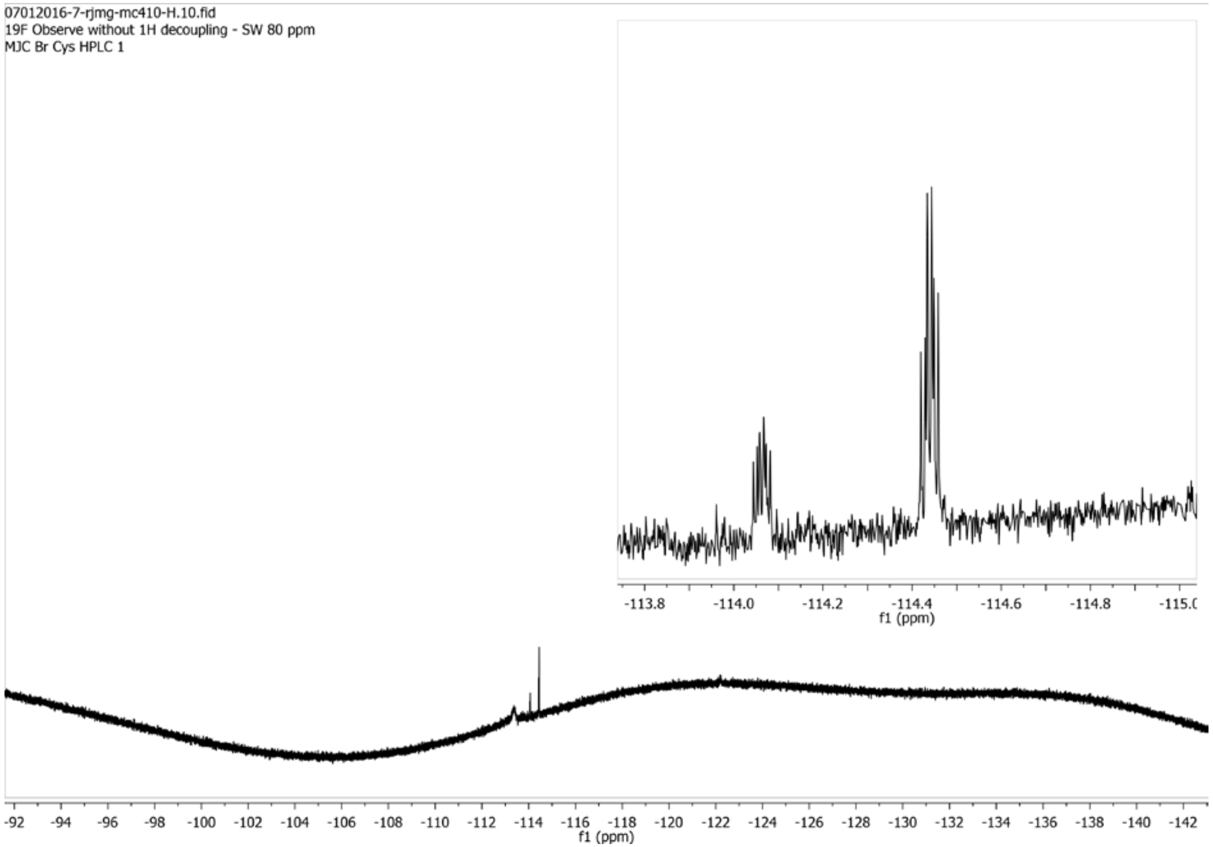

^{19}F NMR (D_2O) of **64** and **65**

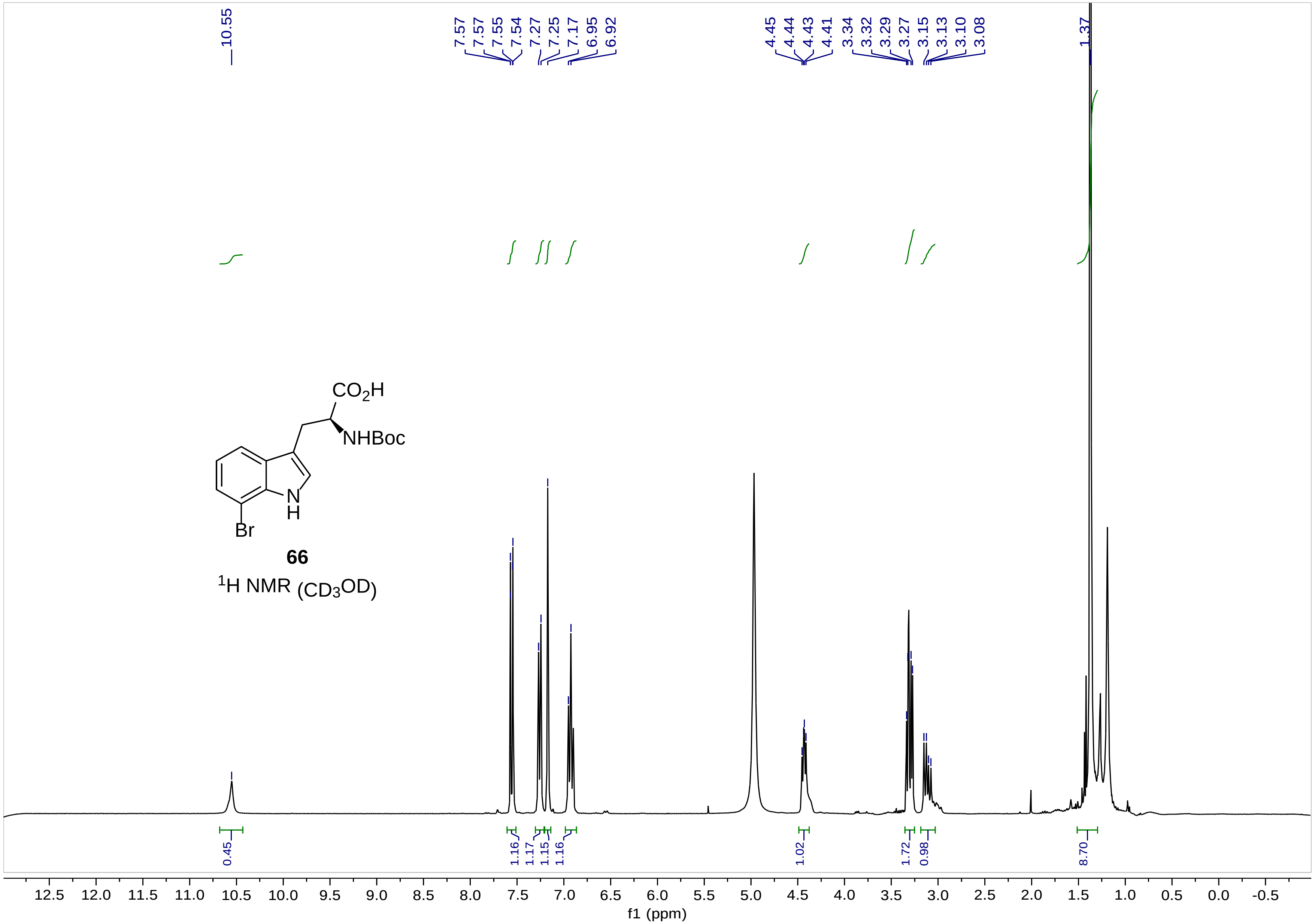

10.55
7.57
7.57
7.55
7.54
7.27
7.25
7.17
6.95
6.92
4.45
4.44
4.43
4.41
3.34
3.32
3.29
3.27
3.15
3.13
3.10
3.08
1.37
CO2H
NHBoc
N
H
Br
66
1H NMR (CD3OD)
0.45
1.16
1.17
1.15
1.16
1.02
1.72
0.98
8.70
12.5 12.0 11.5 11.0 10.5 10.0 9.5 9.0 8.5 8.0 7.5 7.0 6.5 6.0 5.5 5.0 4.5 4.0 3.5 3.0 2.5 2.0 1.5 1.0 0.5 0.0 -0.5
f1 (ppm)

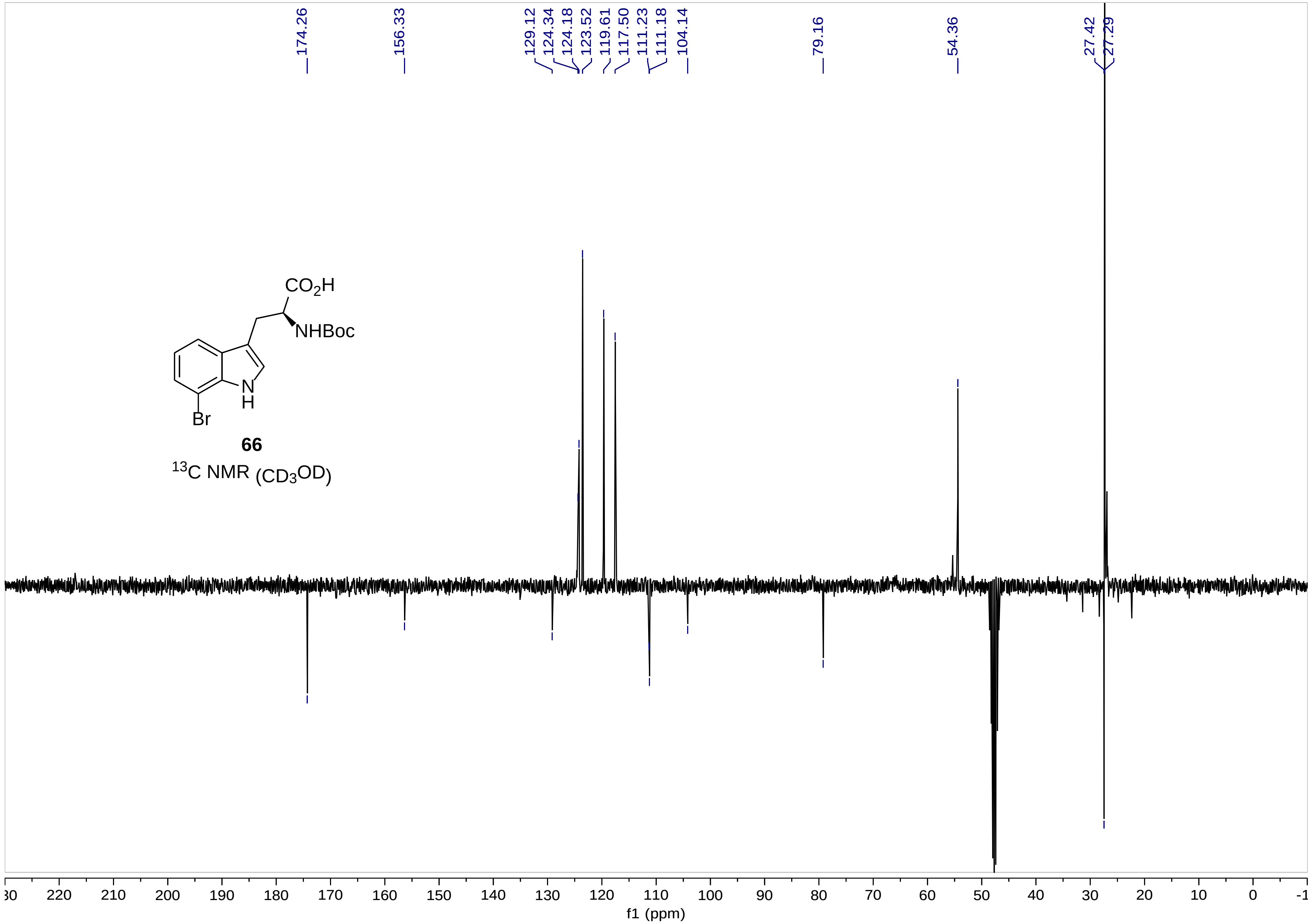

174.26
156.33
129.12
124.34
124.18
123.52
119.61
117.50
111.23
111.18
104.14
79.16
54.36
27.42
27.29
CO_2H
NHBoc
N
H
Br
66
^{13}C NMR (CD_3OD)
f1 (ppm)

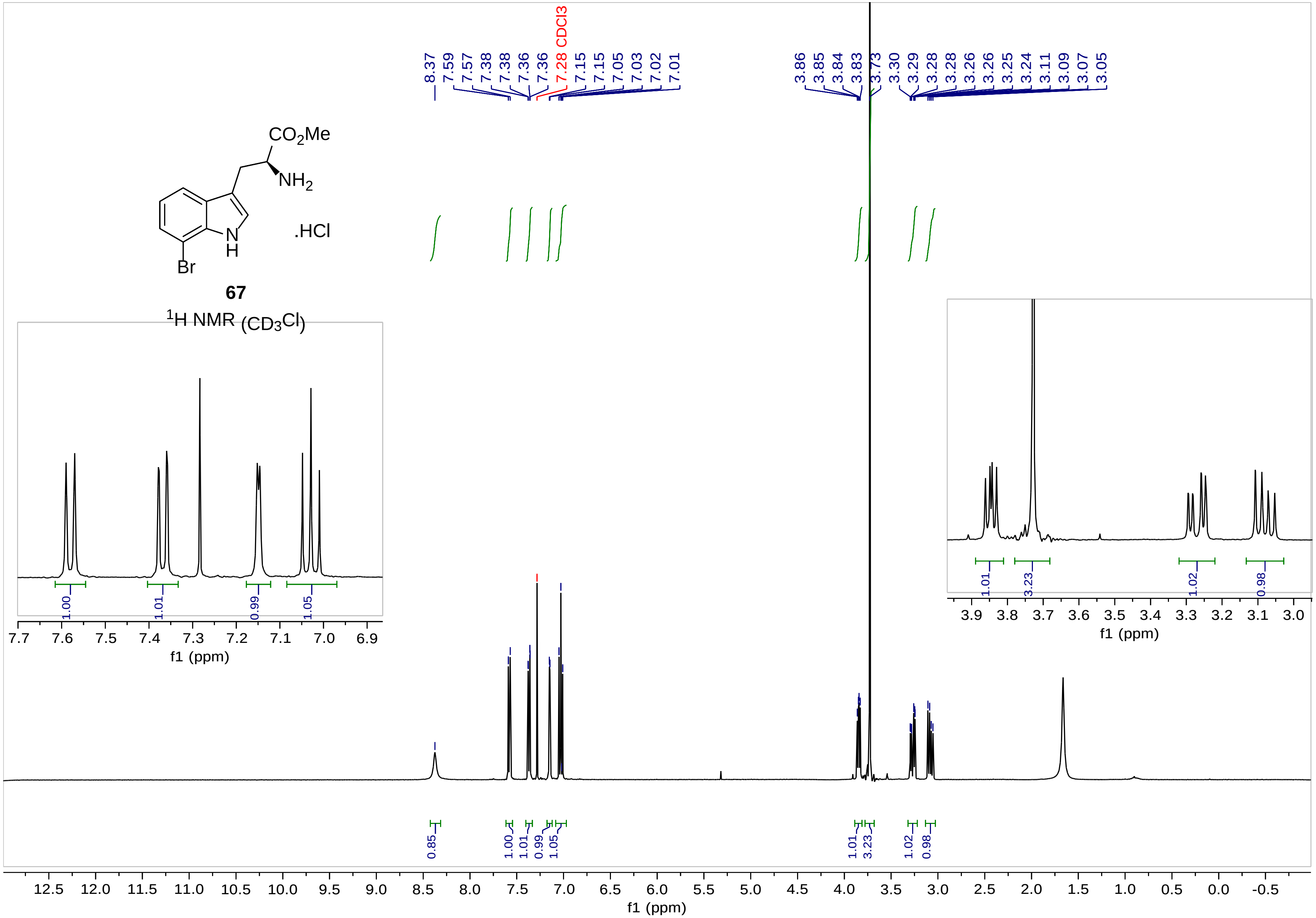

67

^{1}H NMR (CD$_3$Cl)

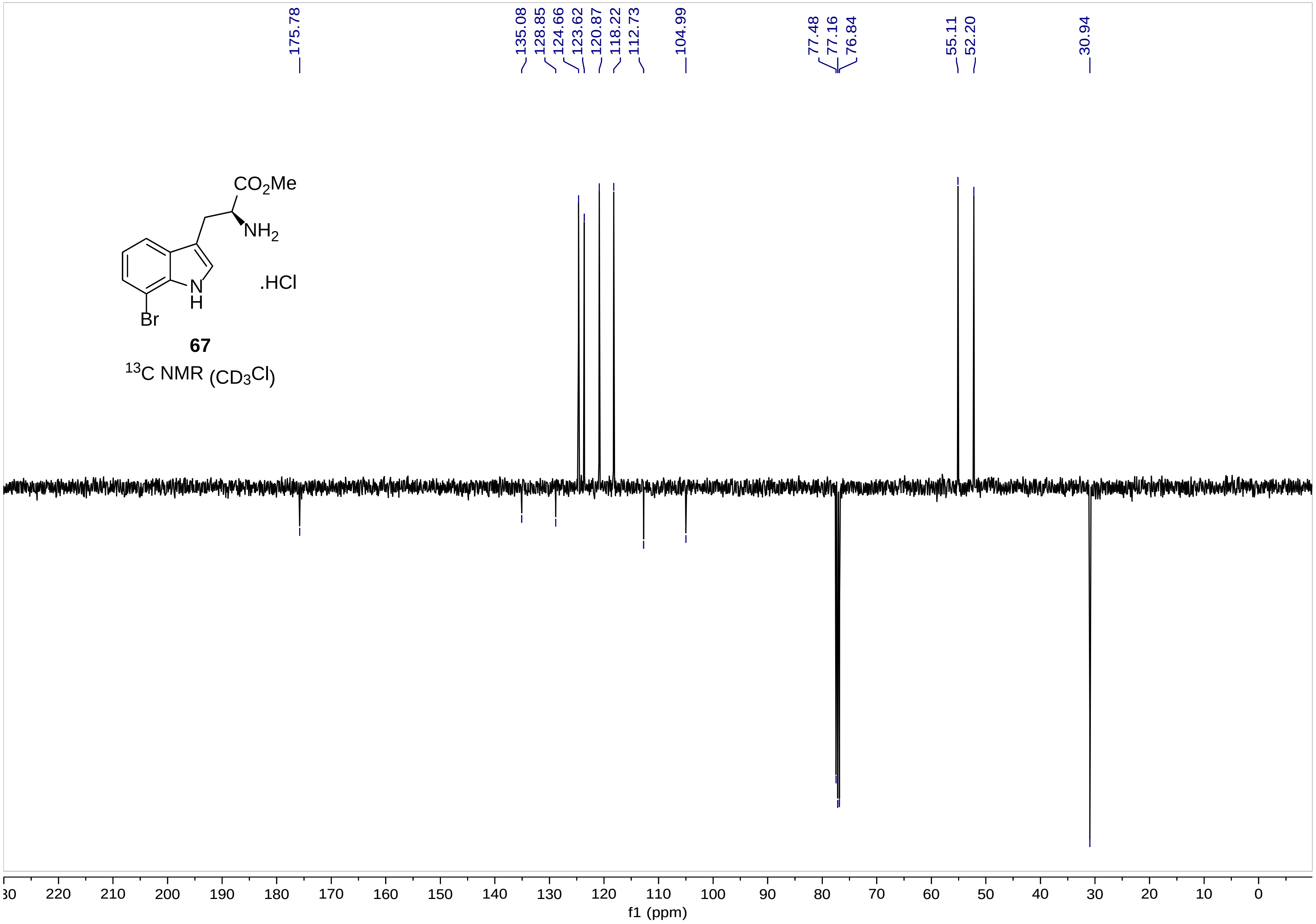
175.78
135.08
128.85
124.66
123.62
120.87
118.22
112.73
104.99
77.48
77.16
76.84
55.11
52.20
30.94
CO2Me
NH2
N
H
Br
.HCl
67
13C NMR (CD3Cl)
230
220
210
200
190
180
170
160
150
140
130
120
110
100
90
80
70
60
50
40
30
20
10
0
f1 (ppm)

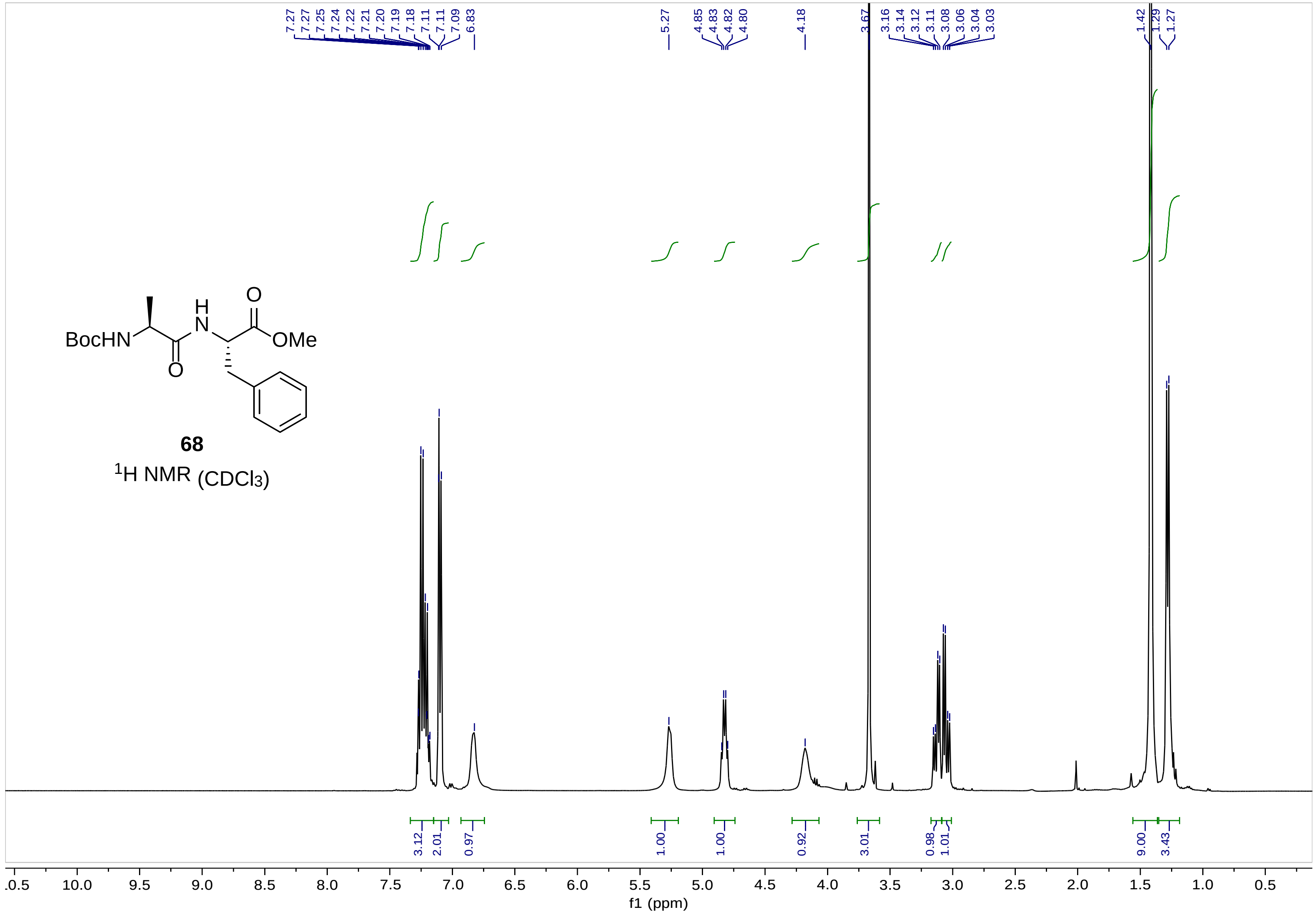

BocHN
H
N
O
O
OMe
68
^{1}H NMR (CDCl$_3$)
7.27 7.27 7.25 7.24 7.22 7.21 7.20 7.19 7.18 7.11 7.11 7.09 6.83
5.27
4.85 4.83 4.82 4.80
4.18
3.67
3.16 3.14 3.12 3.11 3.08 3.06 3.04 3.03
1.42 1.29 1.27
3.12 2.01 0.97 1.00 1.00 0.92 3.01 0.98 1.01 9.00 3.43
f1 (ppm)

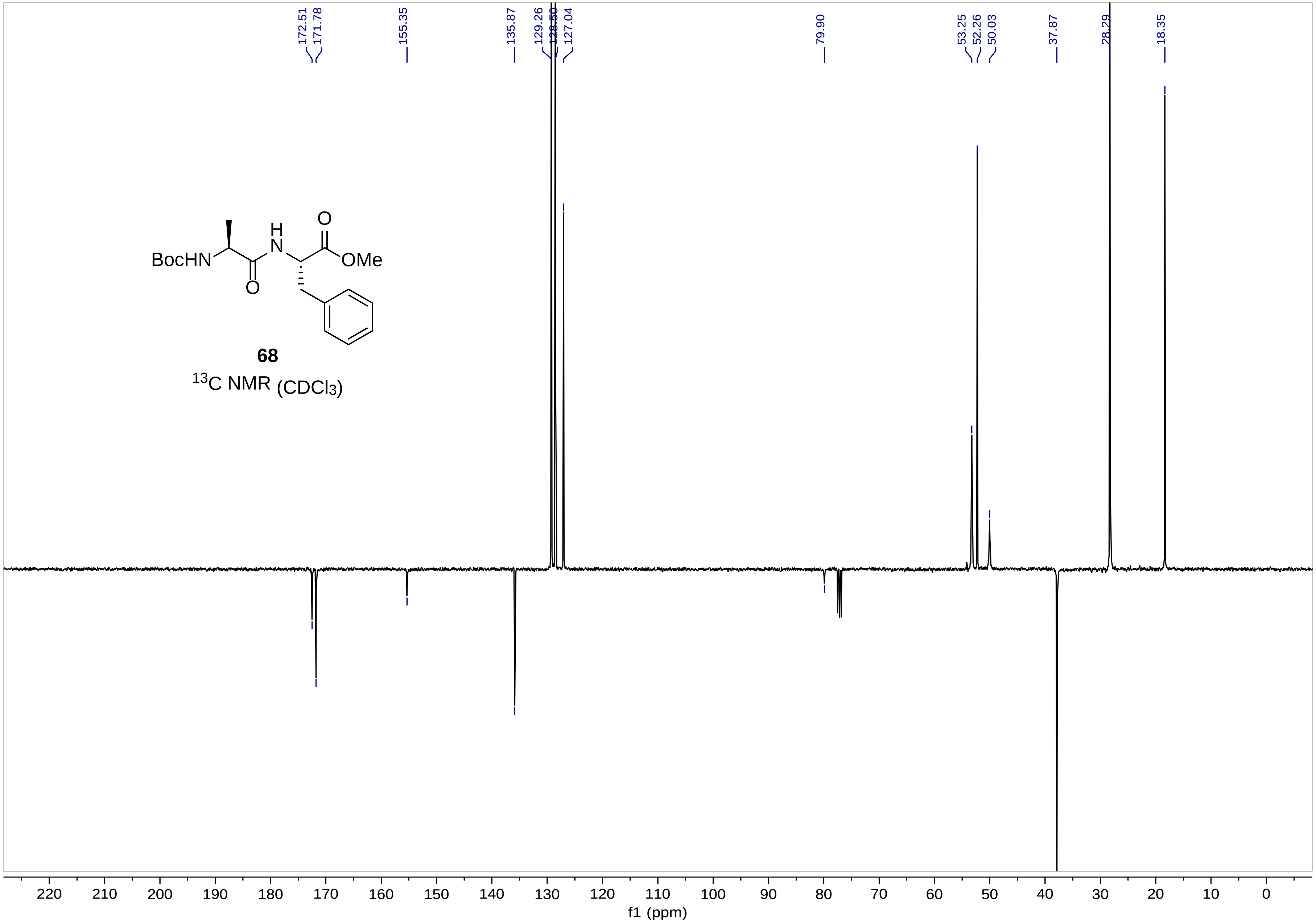

172.51
171.78
155.35
135.87
129.26
128.50
127.04
79.90
53.25
52.26
50.03
37.87
28.29
18.35
BocHN
H
N
O
OMe
O
68
^{13}C NMR (CDCl$_3$)
220 210 200 190 180 170 160 150 140 130 120 110 100 90 80 70 60 50 40 30 20 10 0
f1 (ppm)

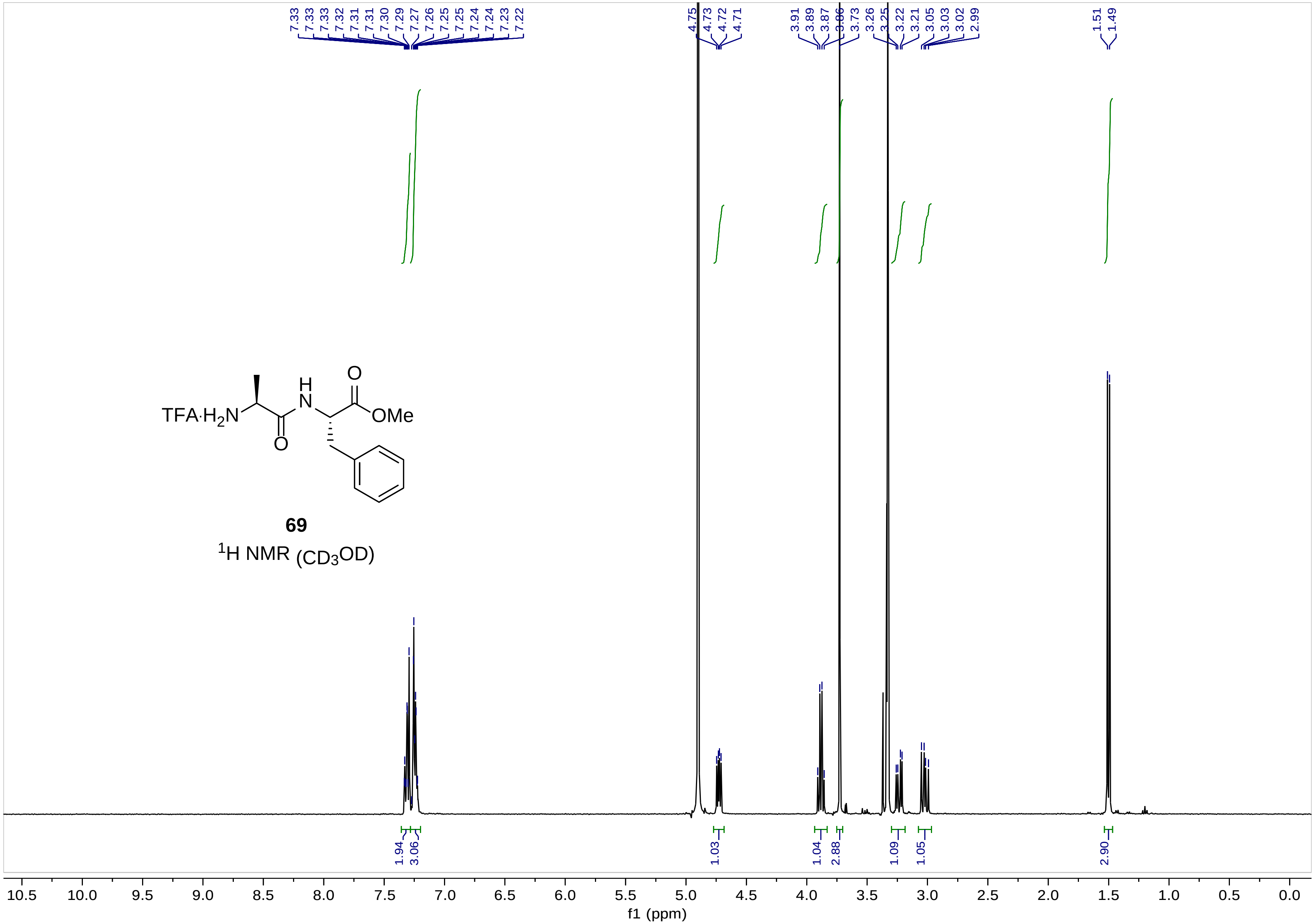
7.33 7.33 7.33 7.32 7.31 7.31 7.30 7.29 7.27 7.26 7.25 7.25 7.24 7.24 7.23 7.22
4.75 4.73 4.72 4.71
3.91 3.89 3.87 3.86 3.73 3.26 3.25 3.22 3.21 3.05 3.03 3.02 2.99
1.51 1.49
TFA·H2N
H
N
O
OMe
O
69
1H NMR (CD3OD)
1.94 3.06 1.03 1.04 2.88 1.09 1.05 2.90
10.5 10.0 9.5 9.0 8.5 8.0 7.5 7.0 6.5 6.0 5.5 5.0 4.5 4.0 3.5 3.0 2.5 2.0 1.5 1.0 0.5 0.0
f1 (ppm)

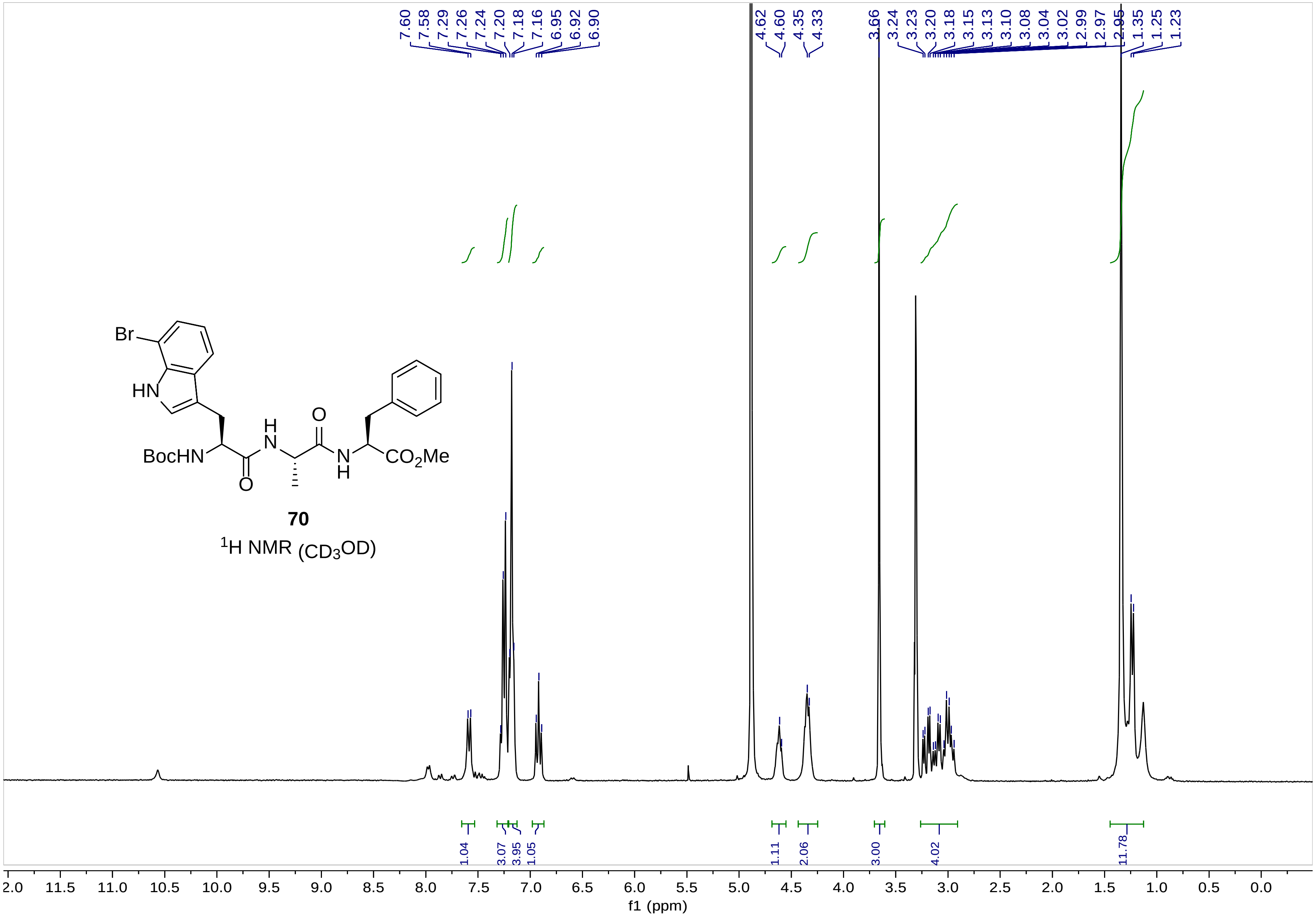
7.60 7.58 7.29 7.26 7.24 7.20 7.18 7.16 6.95 6.92 6.90
4.62 4.60 4.35 4.33
3.66 3.24 3.23 3.20 3.18 3.15 3.13 3.10 3.08 3.04 3.02 2.99 2.97 2.95 1.35 1.25 1.23
Br
HN
BocHN
H
N
O
O
N
H
CO_2Me
70
^{1}H NMR (CD_3OD)
1.04 3.07 3.95 1.05 1.11 2.06 3.00 4.02 11.78
2.0 11.5 11.0 10.5 10.0 9.5 9.0 8.5 8.0 7.5 7.0 6.5 6.0 5.5 5.0 4.5 4.0 3.5 3.0 2.5 2.0 1.5 1.0 0.5 0.0
f1 (ppm)

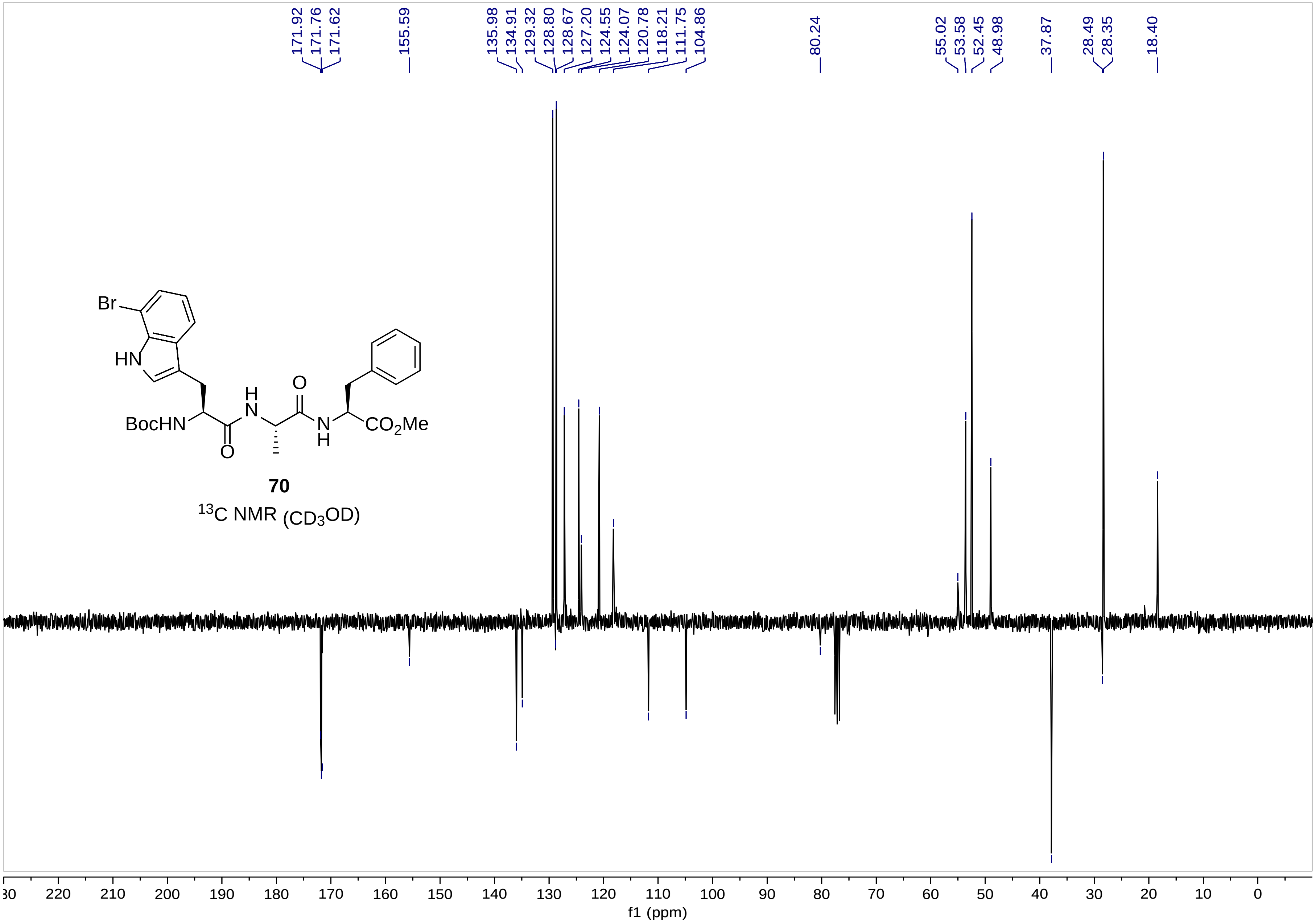

70

^{13}C NMR (CD_3OD)

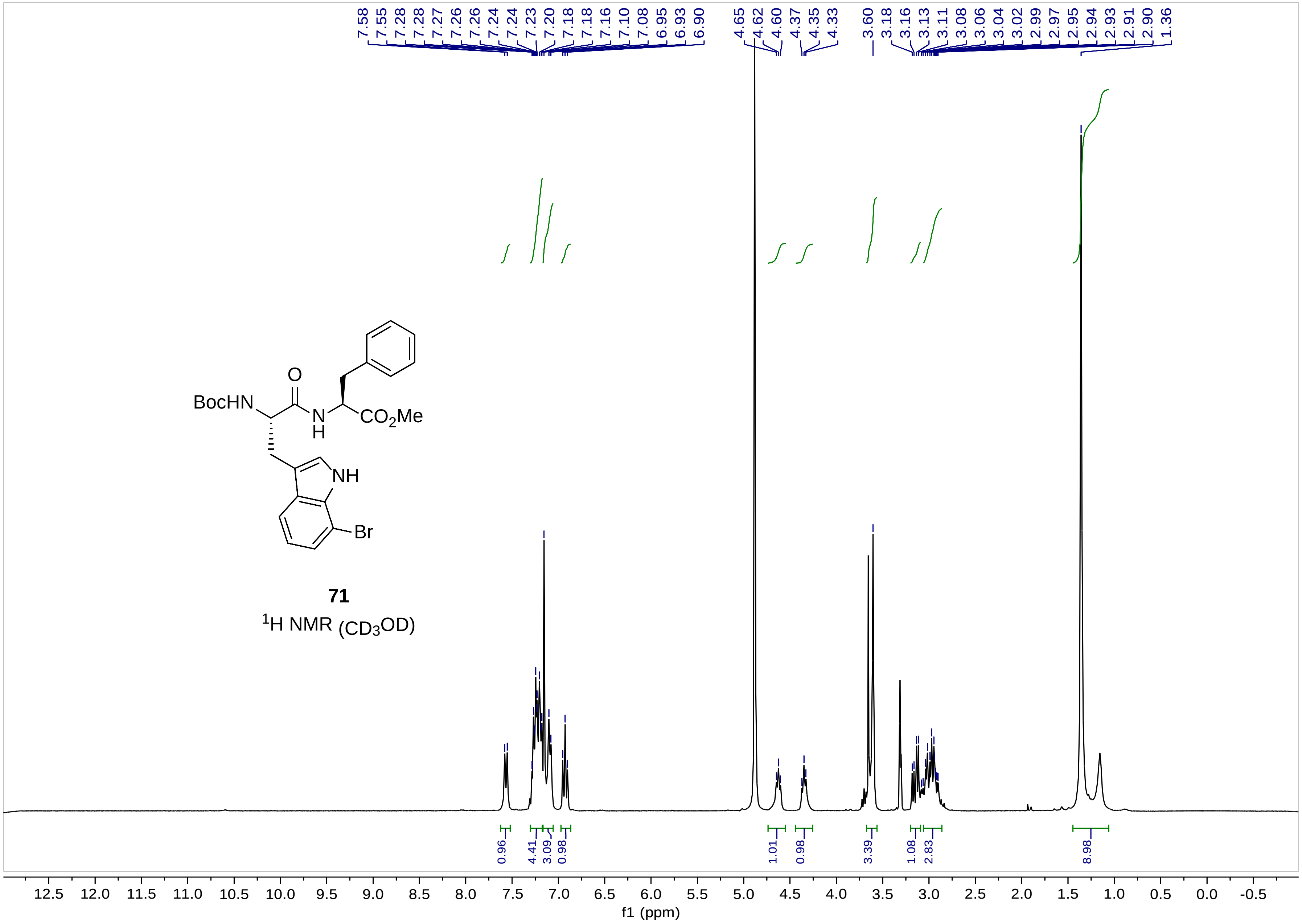

71

^{1}H NMR (CD_3OD)

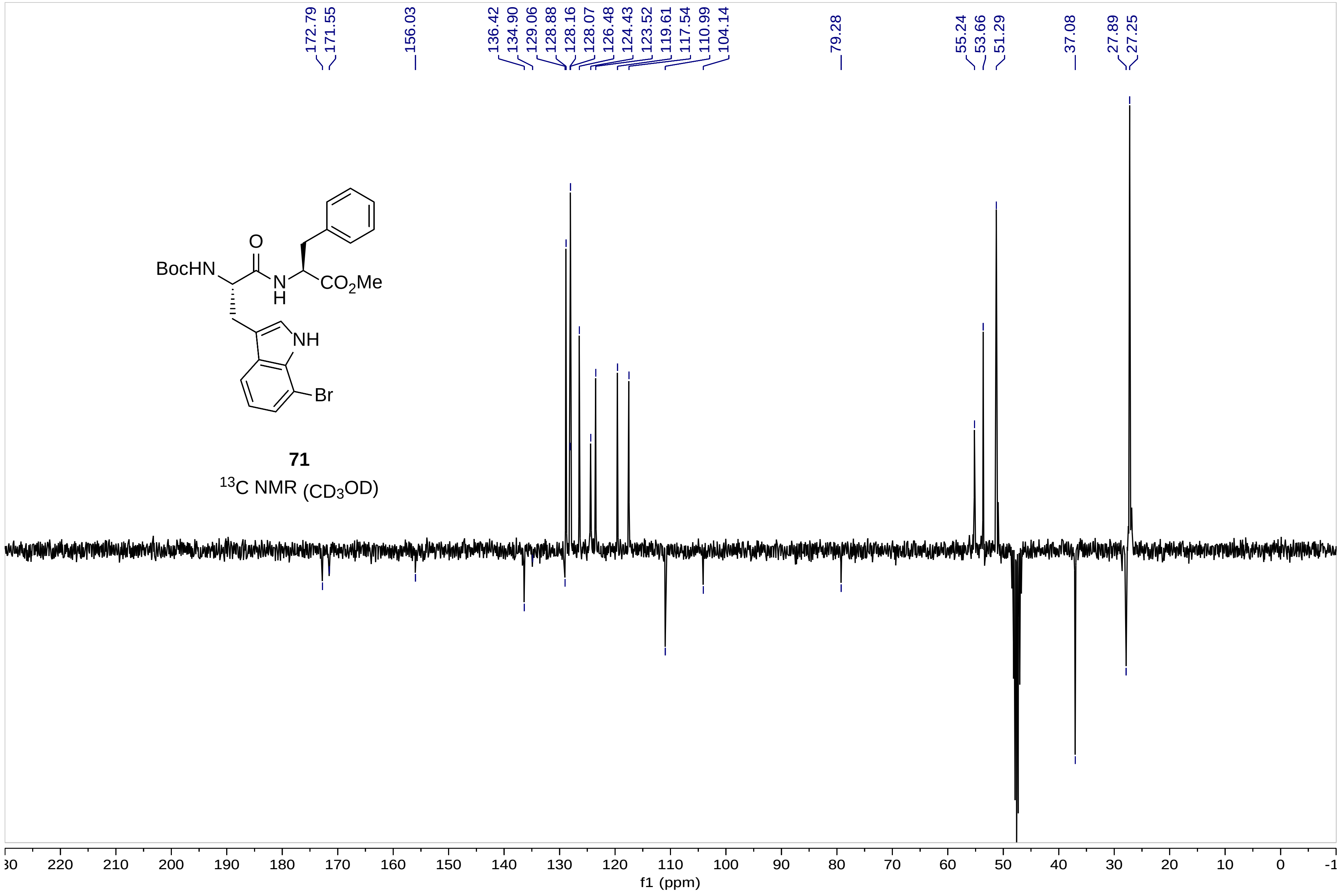
172.79
171.55
156.03
136.42
134.90
129.06
128.88
128.16
128.07
126.48
124.43
123.52
119.61
117.54
110.99
104.14
79.28
55.24
53.66
51.29
37.08
27.89
27.25
BocHN
O
N
H
CO2Me
NH
Br
71
13C NMR (CD3OD)
f1 (ppm)

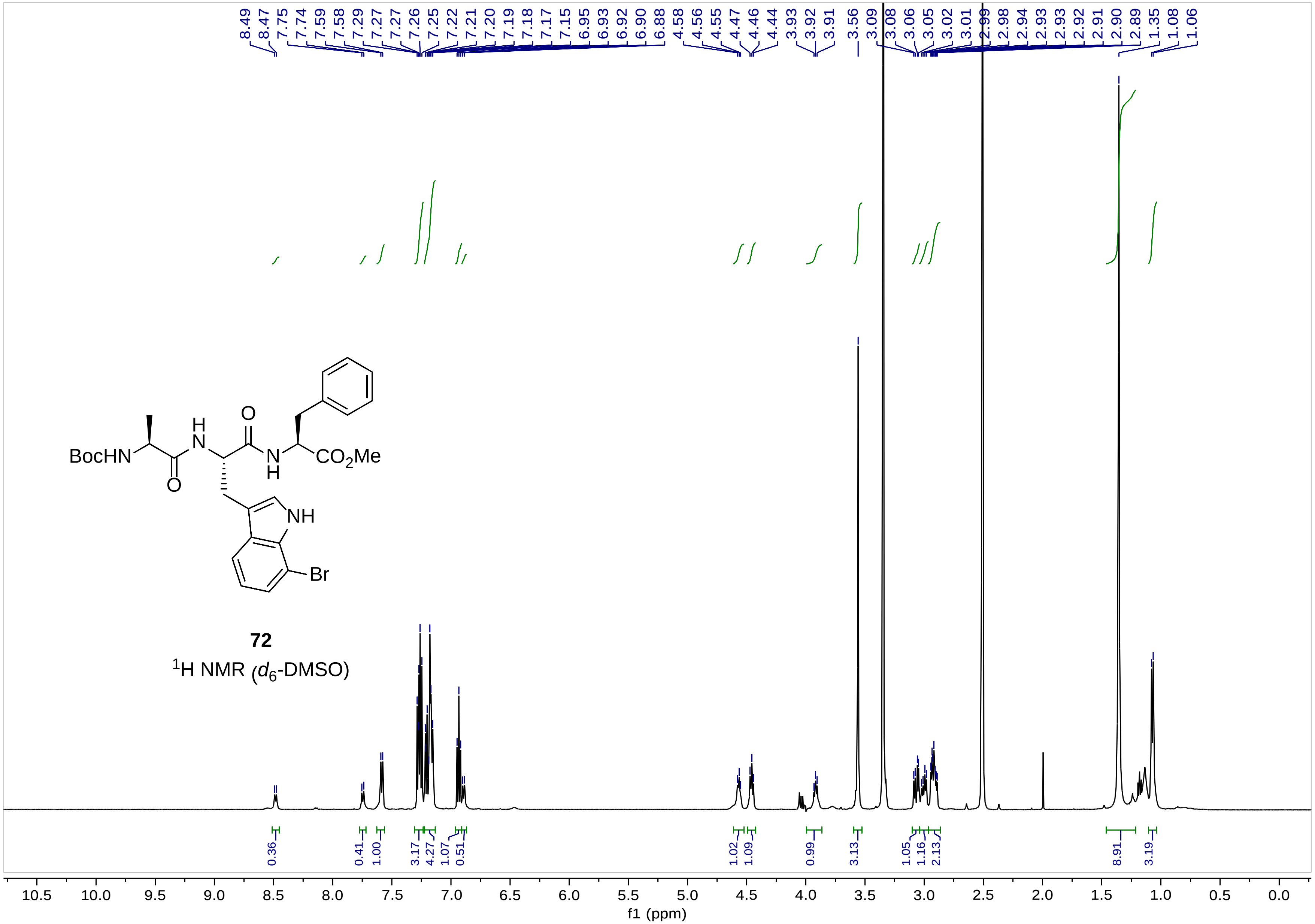
72
1H NMR (d6-DMSO)
BocHN
CO2Me
NH
Br
f1 (ppm)

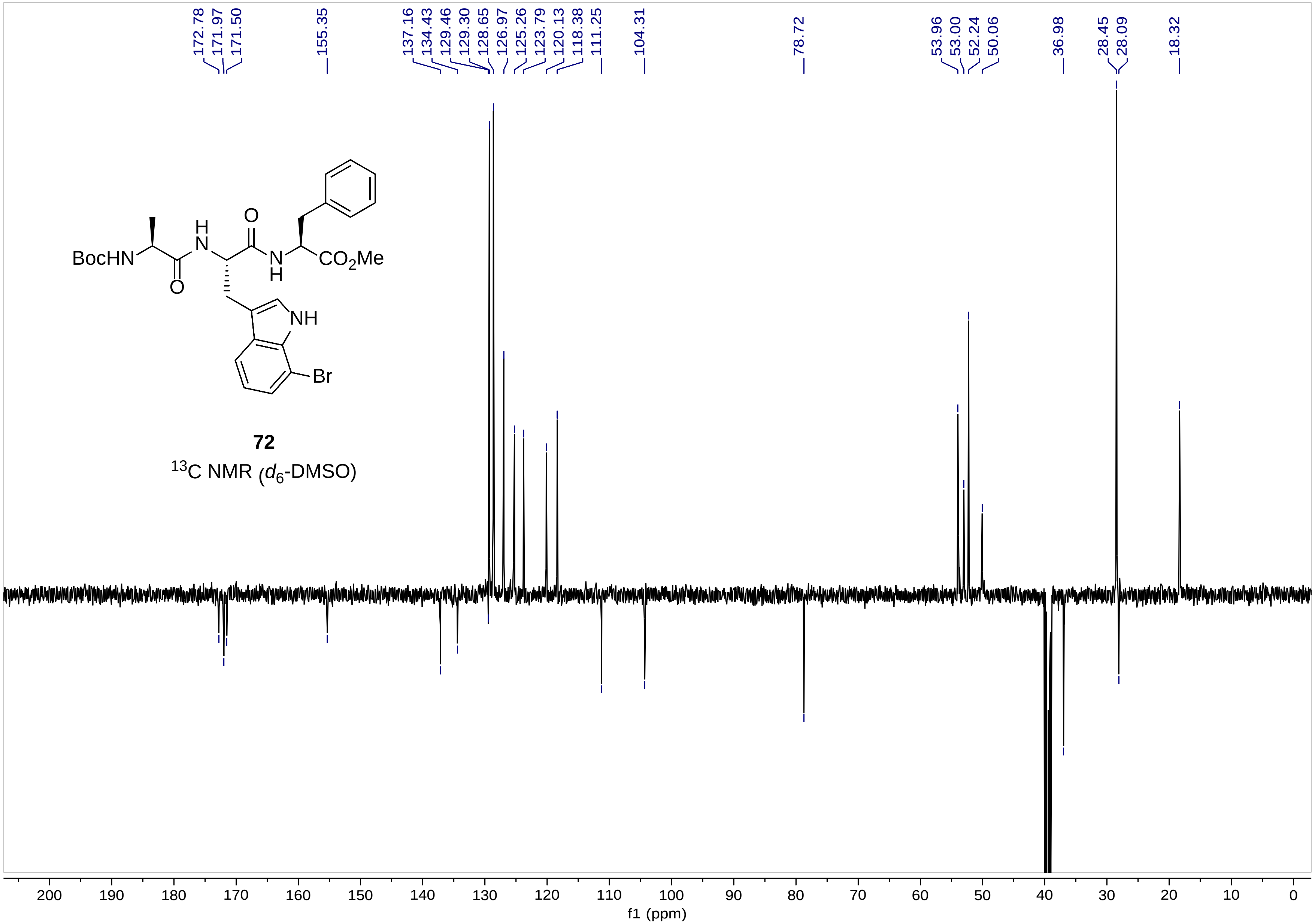
172.78
171.97
171.50
155.35
137.16
134.43
129.46
129.30
128.65
126.97
125.26
123.79
120.13
118.38
111.25
104.31
78.72
53.96
53.00
52.24
50.06
36.98
28.45
28.09
18.32
BocHN
H
N
O
N
H
CO2Me
NH
Br
72
13C NMR (d6-DMSO)
200
190
180
170
160
150
140
130
120
110
100
90
80
70
60
50
40
30
20
10
0
f1 (ppm)

Enantiopurity analyses by UPLC using Marfey's Reagent

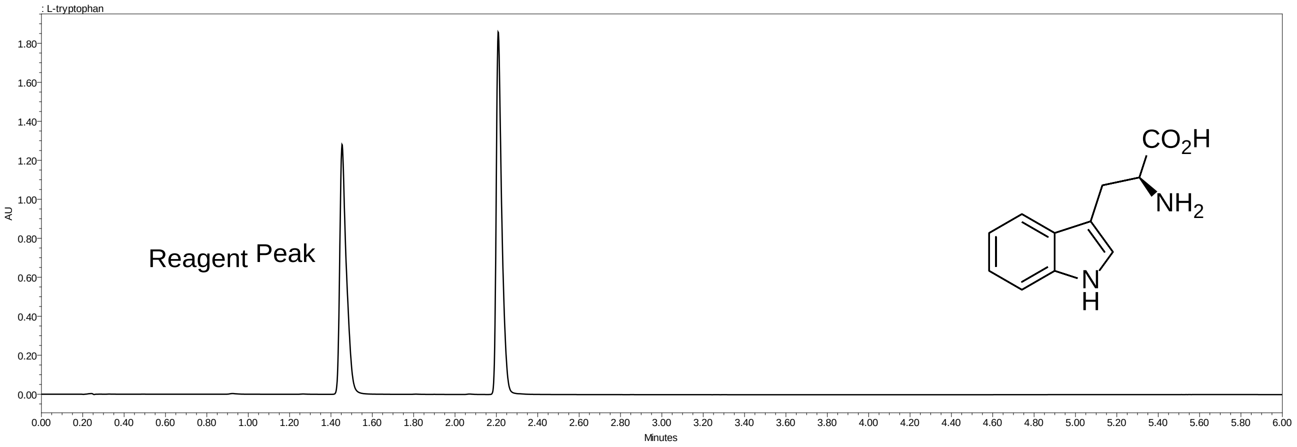

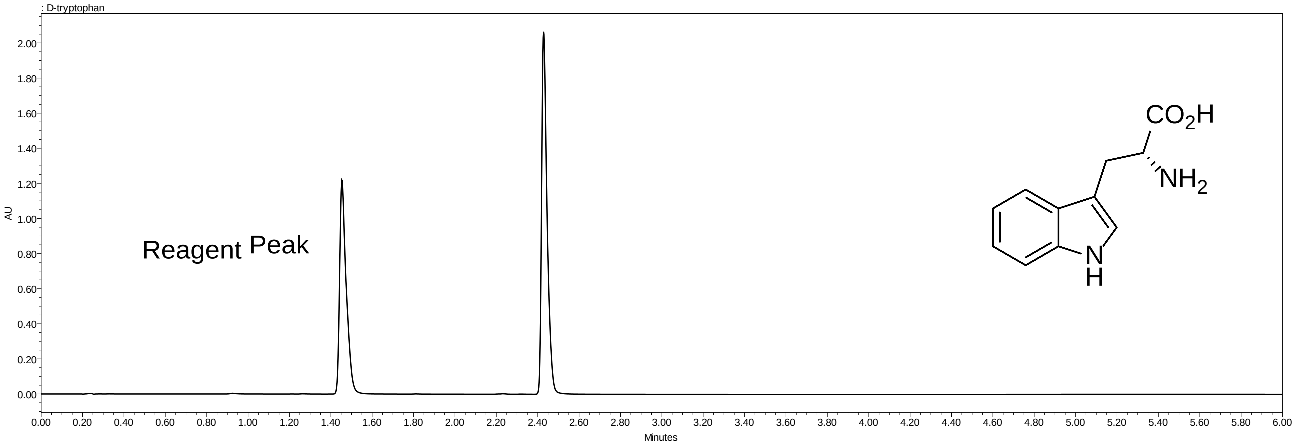

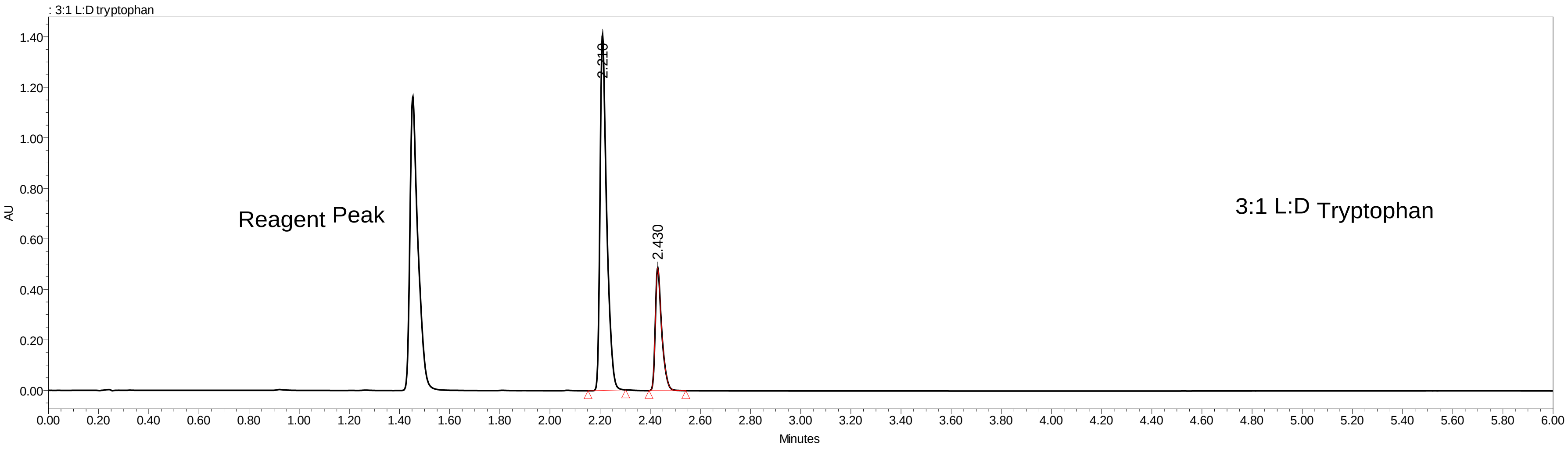

	Name	Retention Time	Area	% Area	Height	Int Type	Amount	Units	Peak Type	Peak Codes	Match_Criteria	Purity
1		2.210	2588715	74.91	1409209	bb			Unknown			

	Name	Retention Time	Area	% Area	Height	Int Type	Amount	Units	Peak Type	Peak Codes	Match_Criteria	Purity
2		2.430	867178	25.09	486339	bb			Unknown			

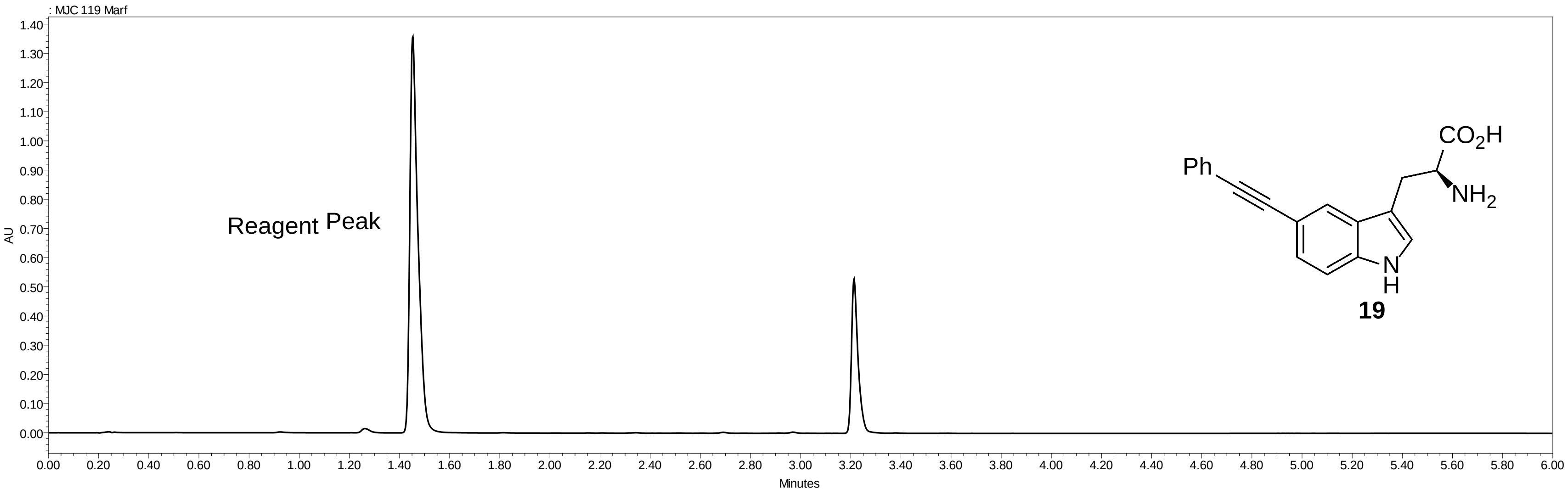
: MJC 119 Marf
AU
Minutes
Reagent Peak
Ph
CO2H
NH2
N
H
19

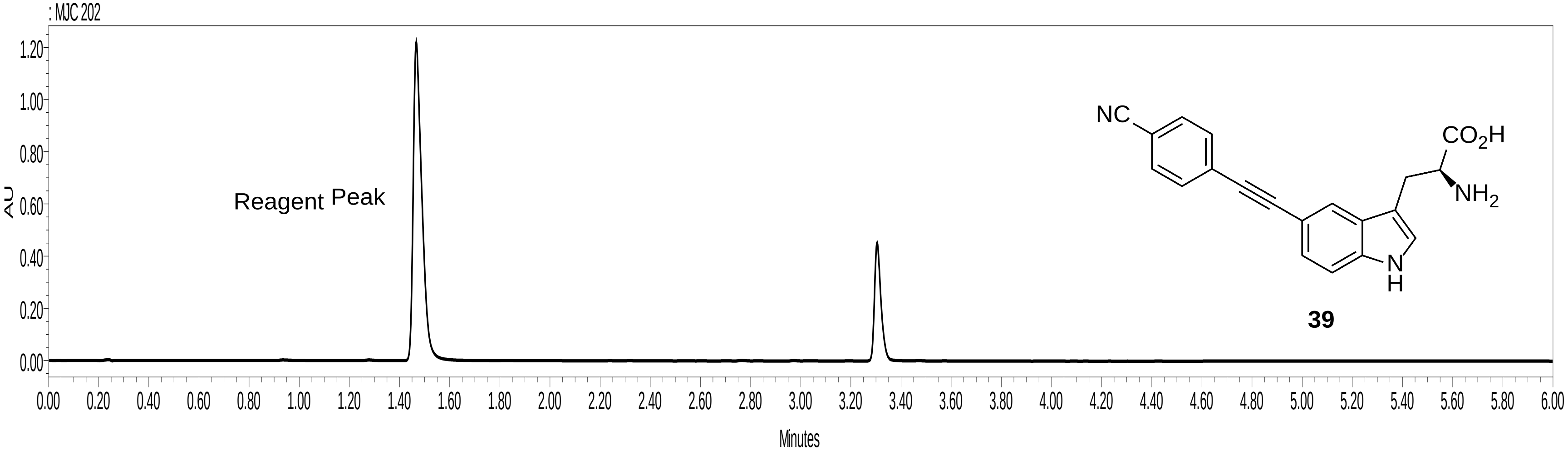
: MJC 202
AU
Minutes
Reagent Peak
NC
CO2H
NH2
N
H
39

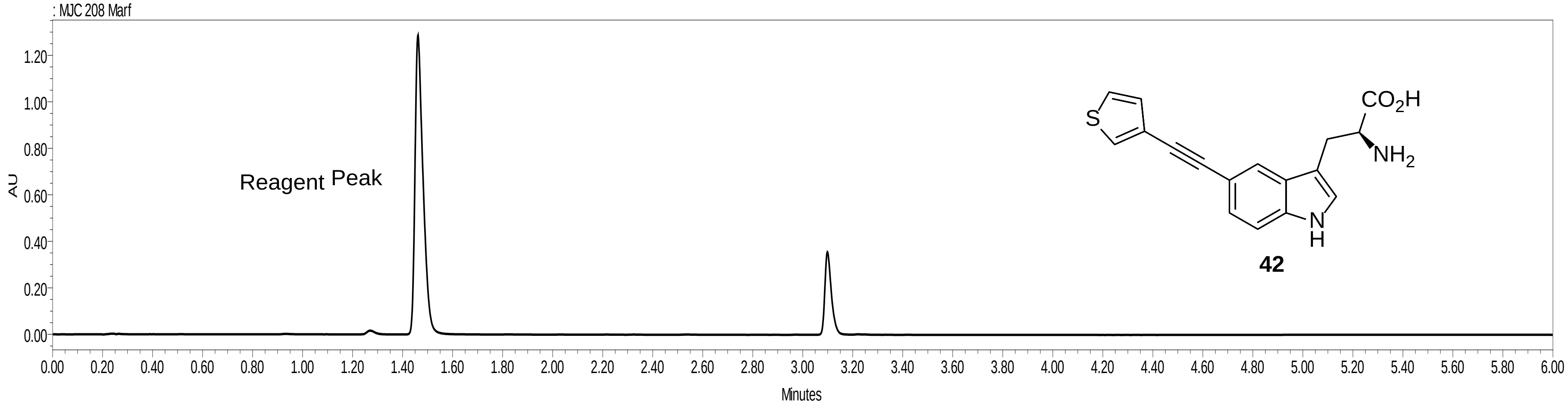
: MJC 208 Marf
AU
1.20
1.00
0.80
0.60
0.40
0.20
0.00
Reagent Peak
0.00 0.20 0.40 0.60 0.80 1.00 1.20 1.40 1.60 1.80 2.00 2.20 2.40 2.60 2.80 3.00 3.20 3.40 3.60 3.80 4.00 4.20 4.40 4.60 4.80 5.00 5.20 5.40 5.60 5.80 6.00
Minutes
S
CO_2H
NH_2
N
H
42

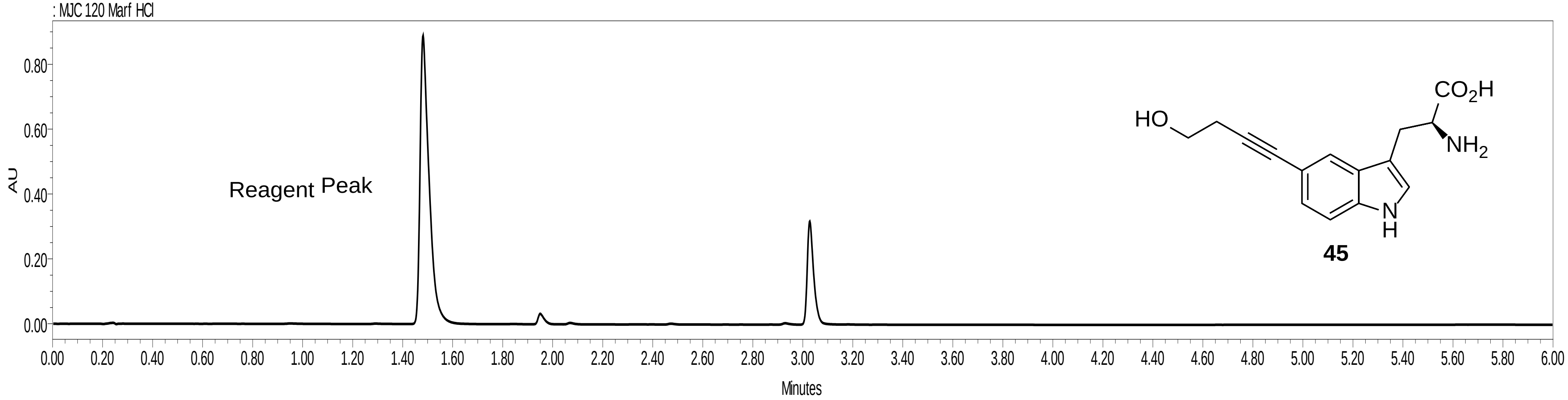
: MJC 120 Marf HCl
AU
0.80
0.60
0.40
0.20
0.00
Reagent Peak
0.00 0.20 0.40 0.60 0.80 1.00 1.20 1.40 1.60 1.80 2.00 2.20 2.40 2.60 2.80 3.00 3.20 3.40 3.60 3.80 4.00 4.20 4.40 4.60 4.80 5.00 5.20 5.40 5.60 5.80 6.00
Minutes
HO
CO_2H
NH_2
N
H
45

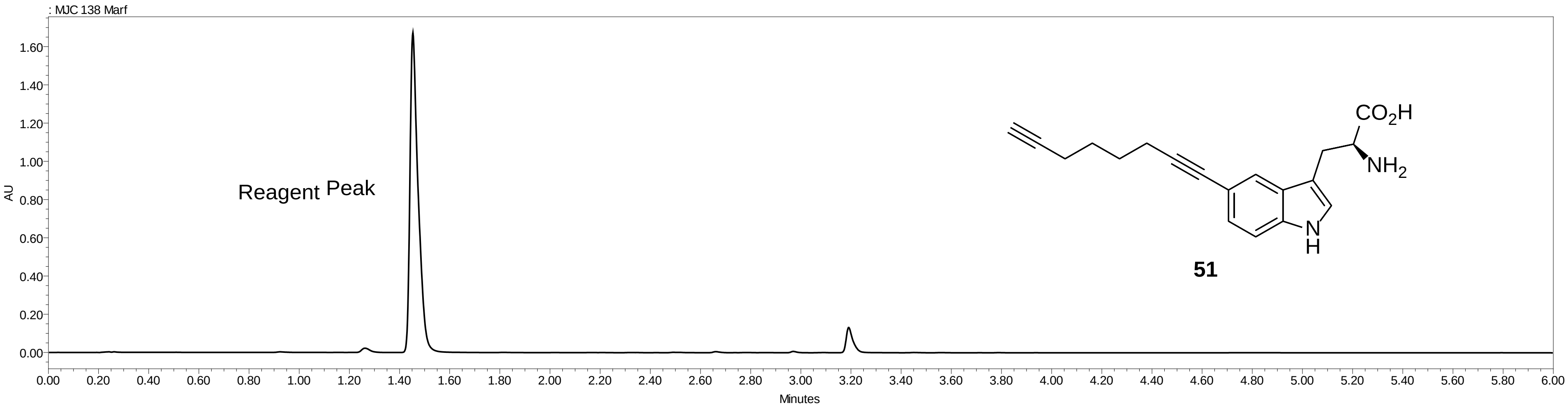
: MJC 138 Marf
AU
Minutes
Reagent Peak
CO2H
NH2
N
H
51
